# Supplementary material for: Altering microtubule dynamics is synergistically toxic with spindle assembly checkpoint inhibition
Source: Life Sci Alliance. 2020 Jan 24;3(2):e201900499. doi: 10.26508/lsa.201900499 (PMC6985455; doi:10.26508/lsa.201900499)
Supplement: Supplementary file 2 [file LSA-2019-00499_Supplemental_Data_1.pdf]

Supplementary data 1: Aneuploid screen growth curves

Aneuploid screen growth curves shown per cell line and per drug used. The top left corner lists the drug ID, the concentration and which cell line these growth curves belong to. RPE hTert are euploid control cells, and RPE1 Ts12 Ts5 are aneuploid double-trisomy cells.

One growth curve is displayed with the cell line DMSO control. If the drug was rescreened, a second cuve is also shown. All concentrations are in micromolar (uM).

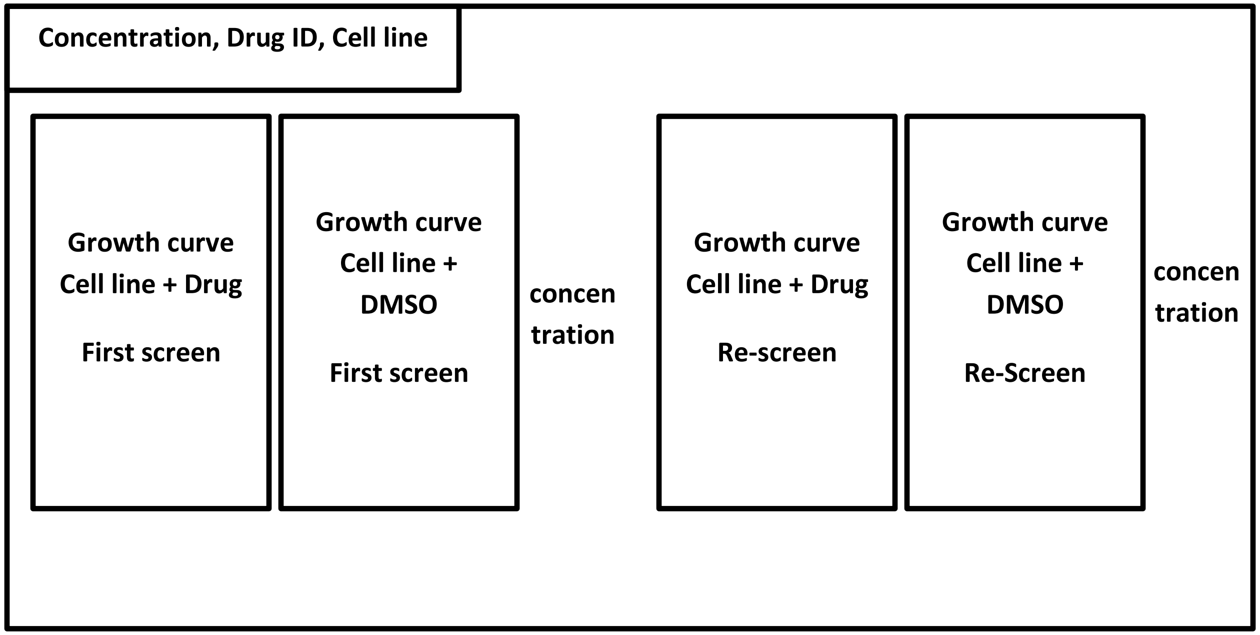

10 uM 1122 RPE1 hTert

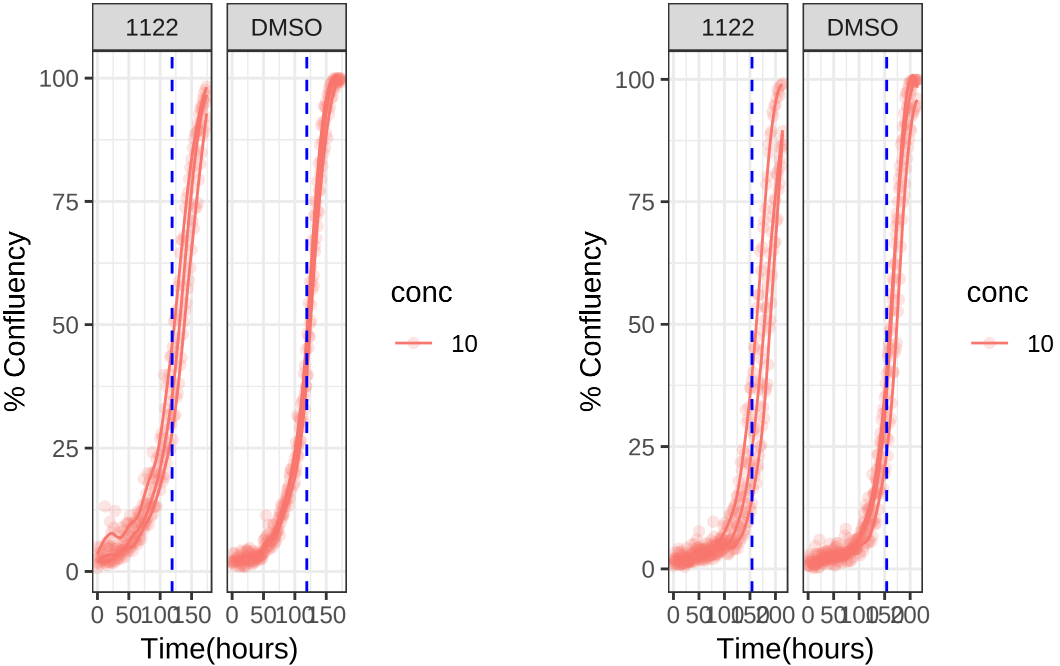

10 uM 1122 RPE1 Ts12 Ts5

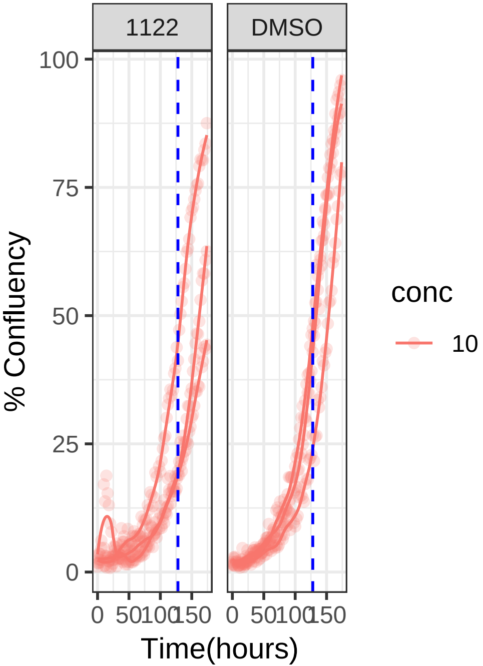

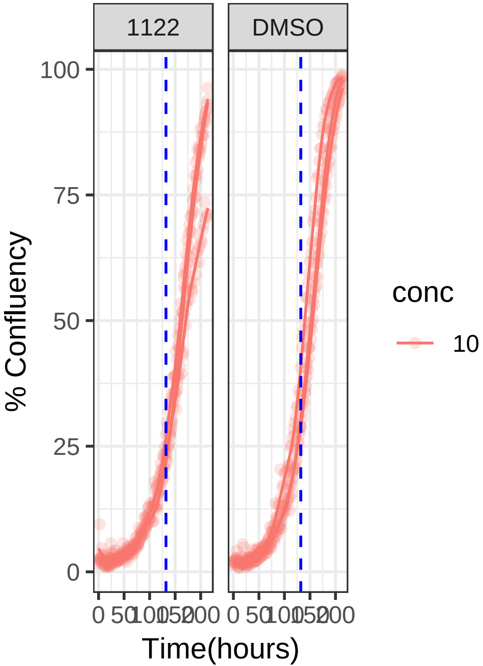

10 uM 1134 RPE1 hTert

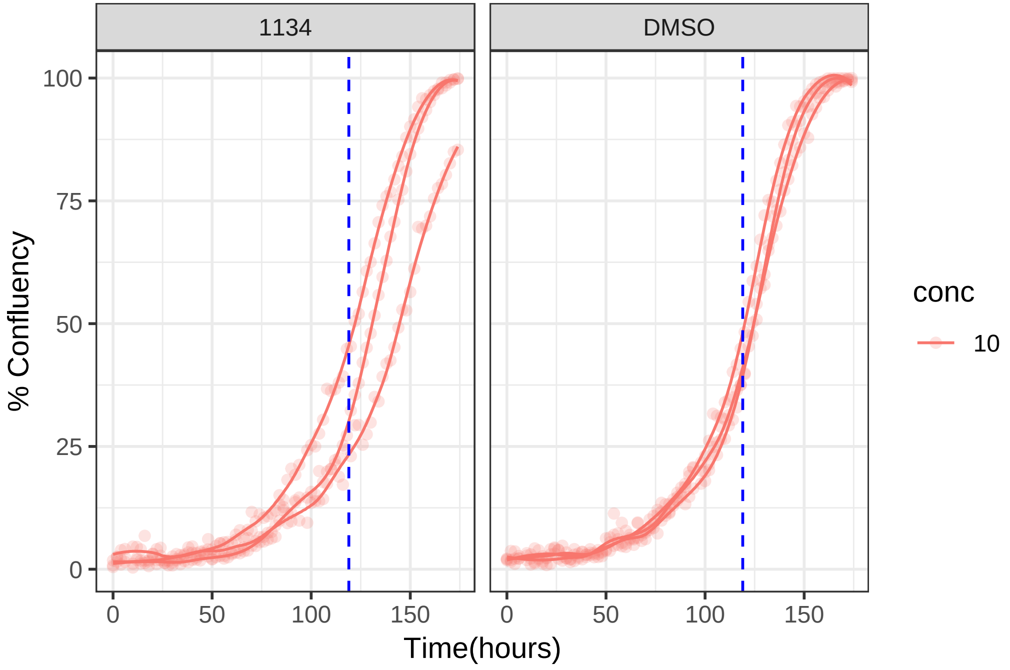

10 uM 1134 RPE1 Ts12 Ts5

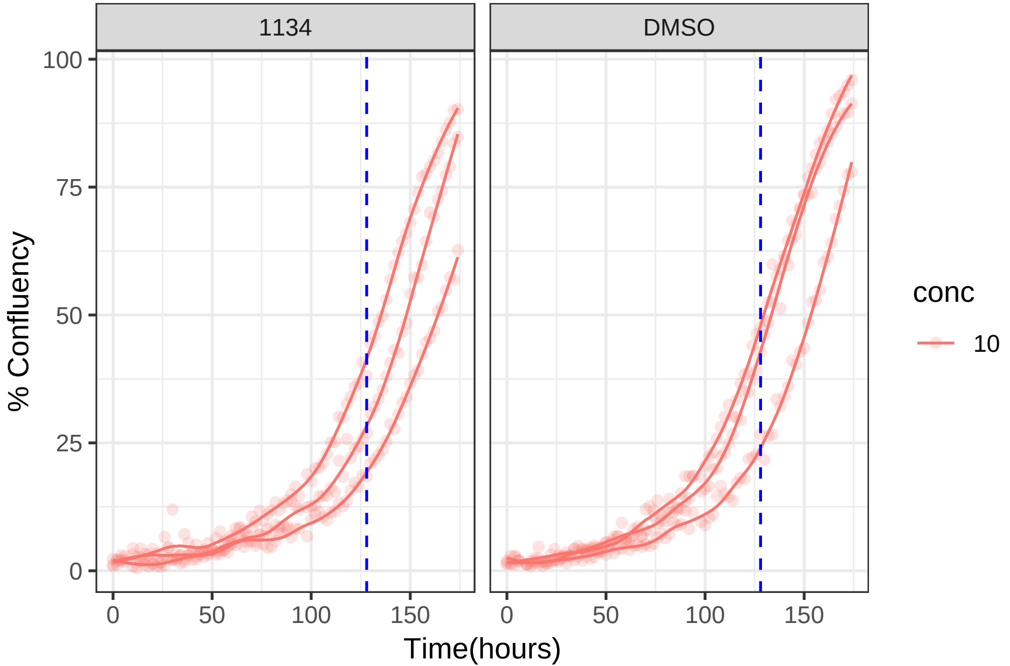

10 uM 1223 RPE1 hTert

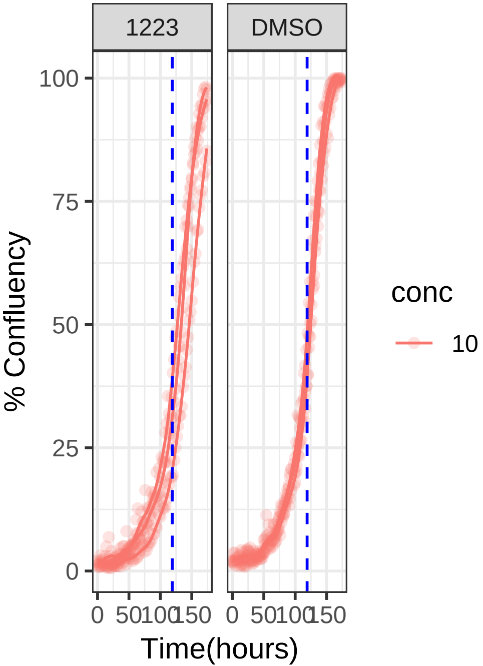

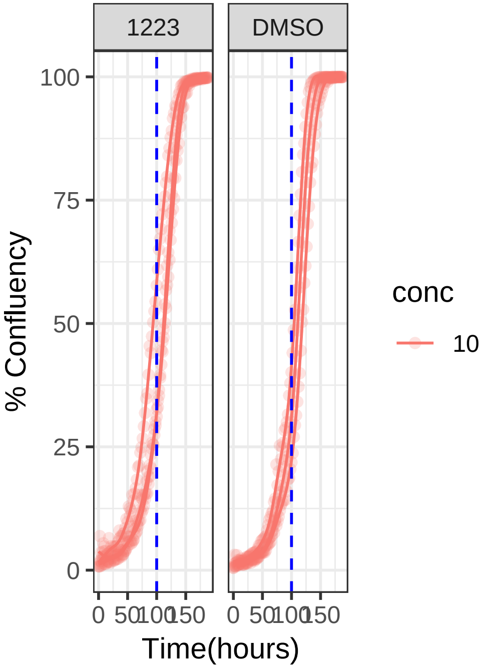

10 uM 1223 RPE1 Ts12 Ts5

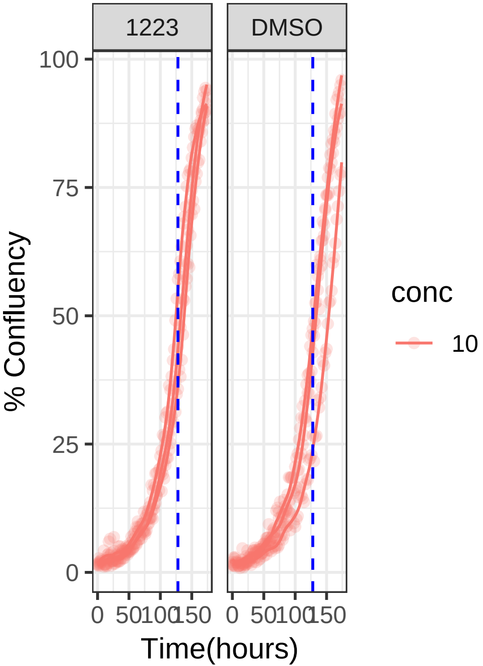

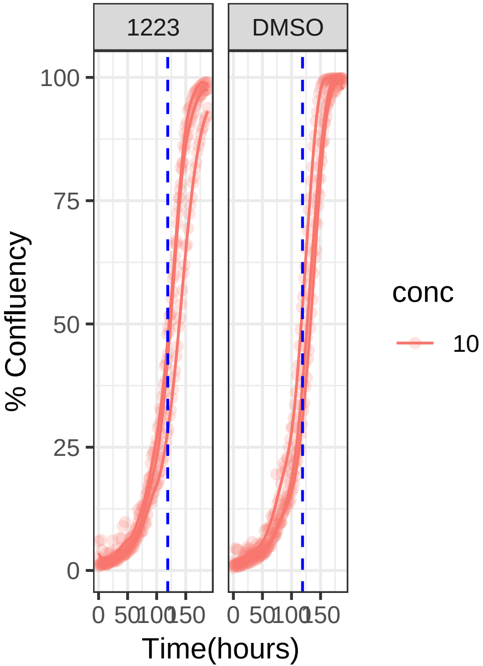

0.001 uM 1233 RPE1 hTert

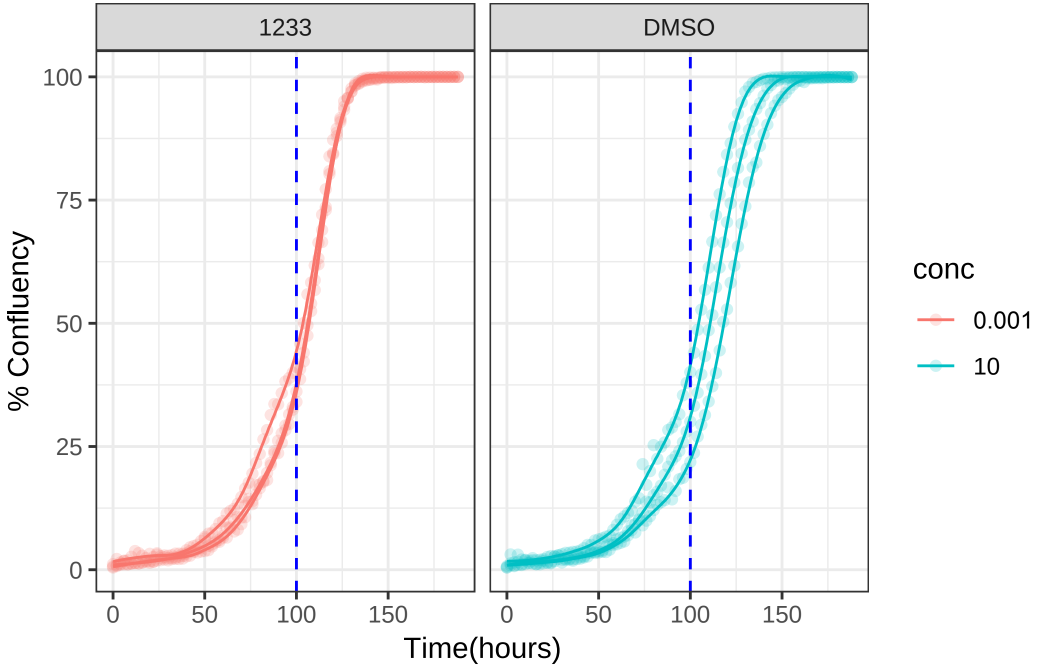

0.001 uM 1233 RPE1 Ts12 Ts5

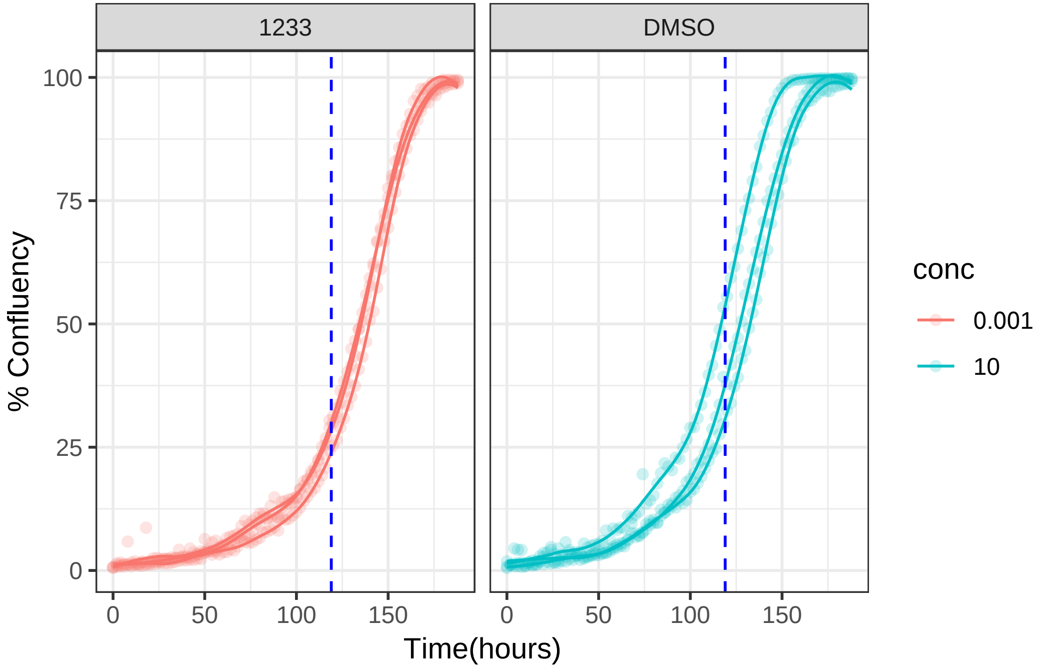

10 uM 1237 RPE1 hTert

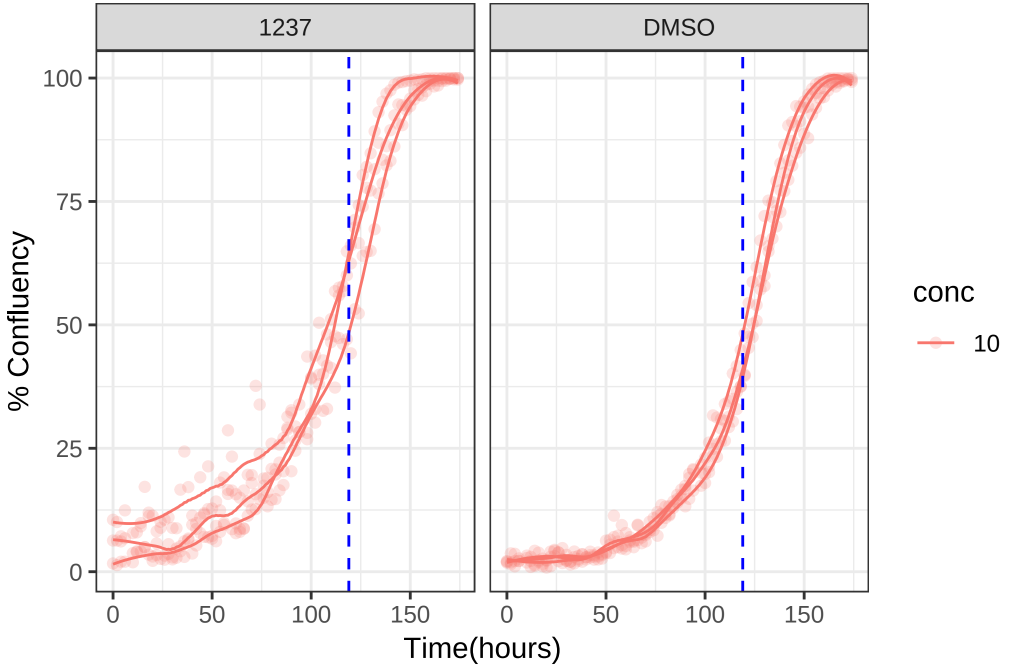

10 uM 1237 RPE1 Ts12 Ts5

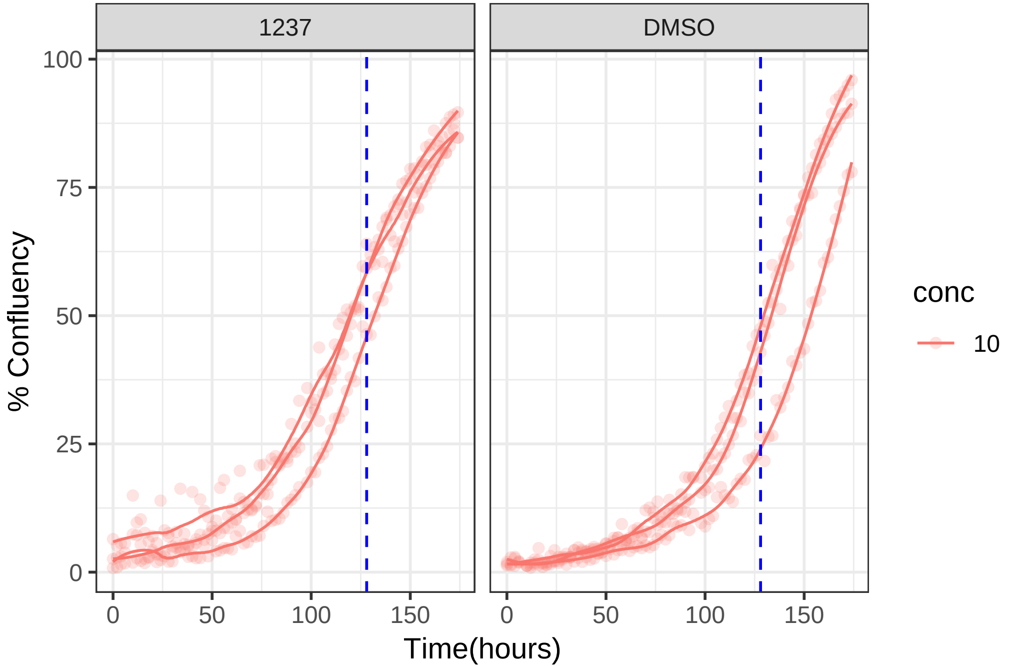

10 uM 1239 RPE1 hTert

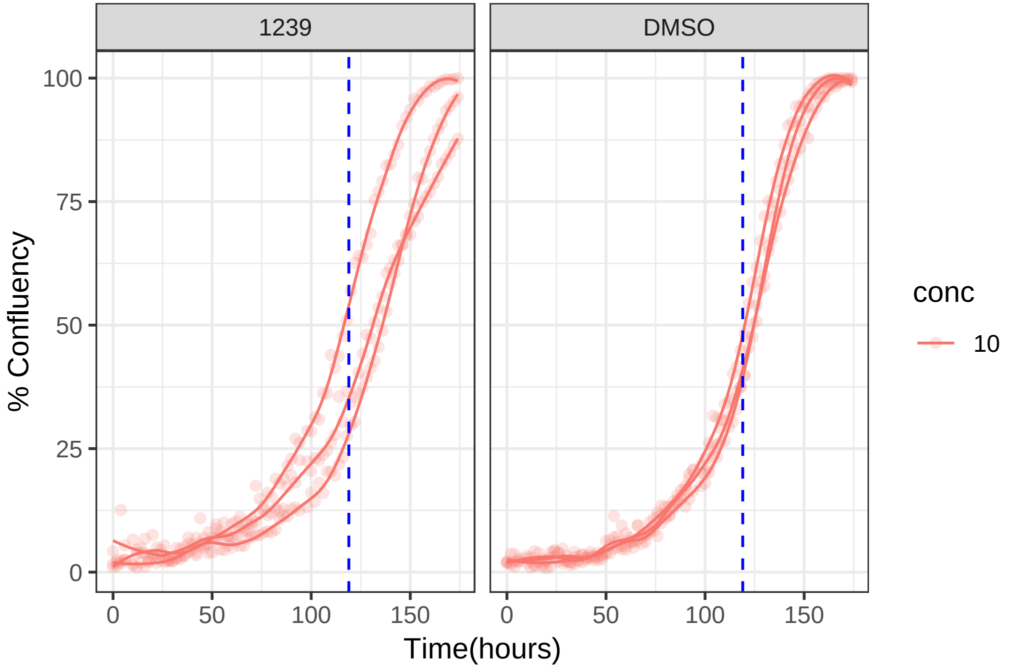

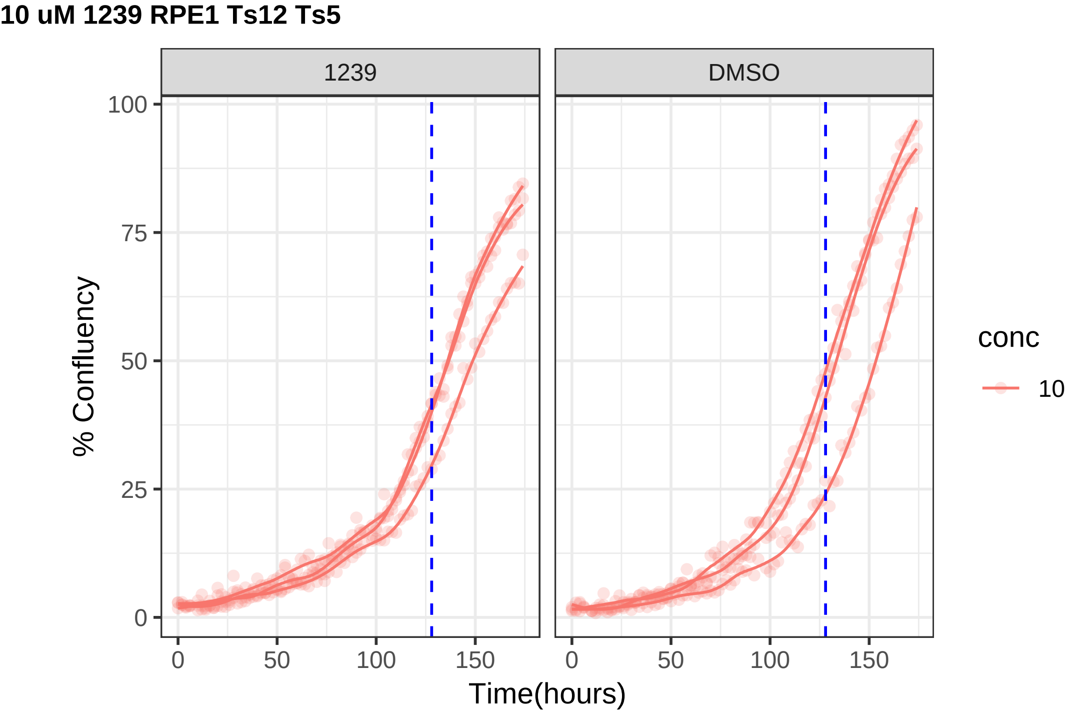
10 uM 1239 RPE1 Ts12 Ts5
1239
DMSO
% Confluency
Time(hours)
100
75
50
25
0
0
50
100
150
conc
10

10 uM 1243 RPE1 hTert

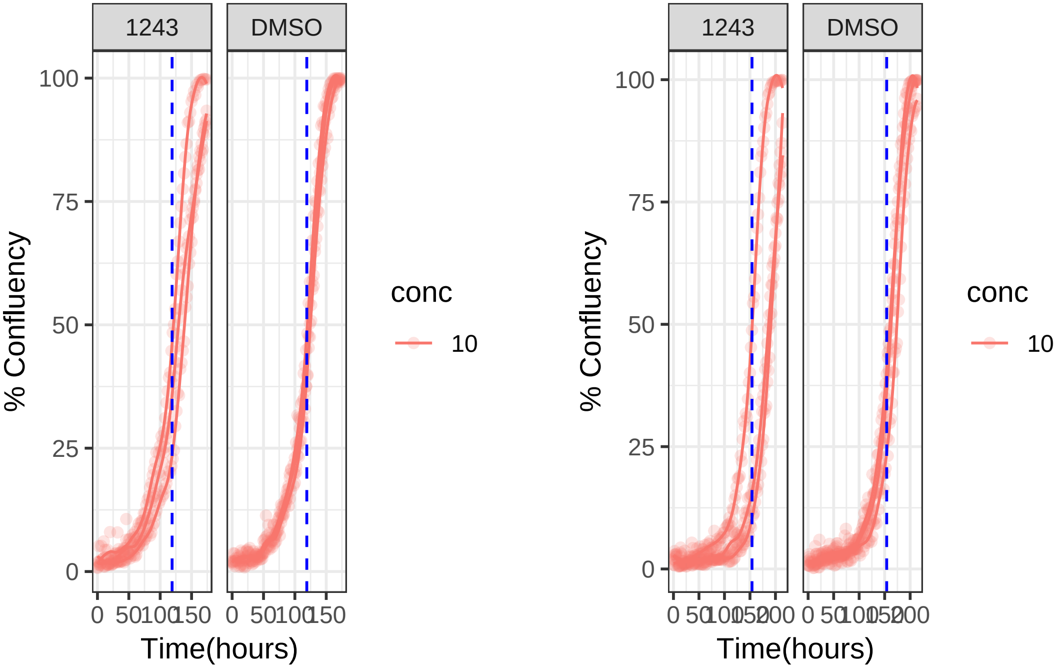

10 uM 1243 RPE1 Ts12 Ts5

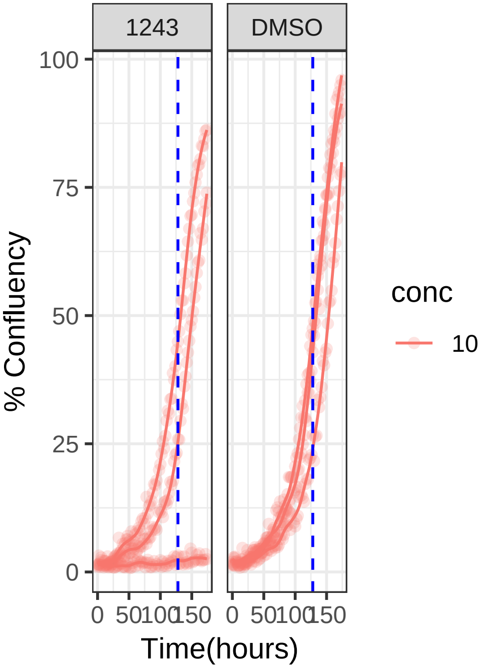

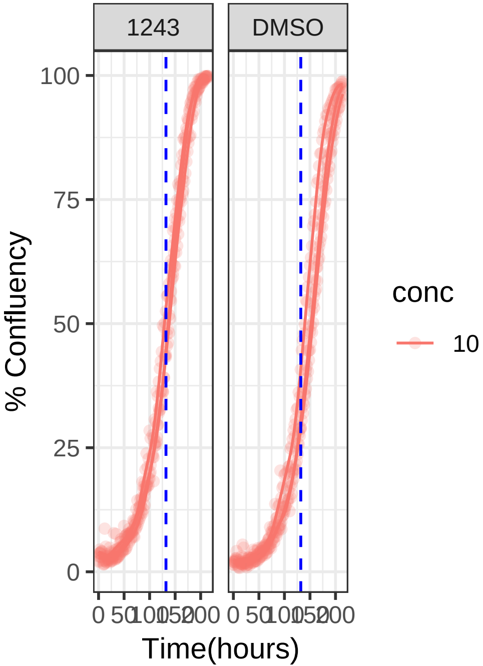

10 uM 1254 RPE1 hTert

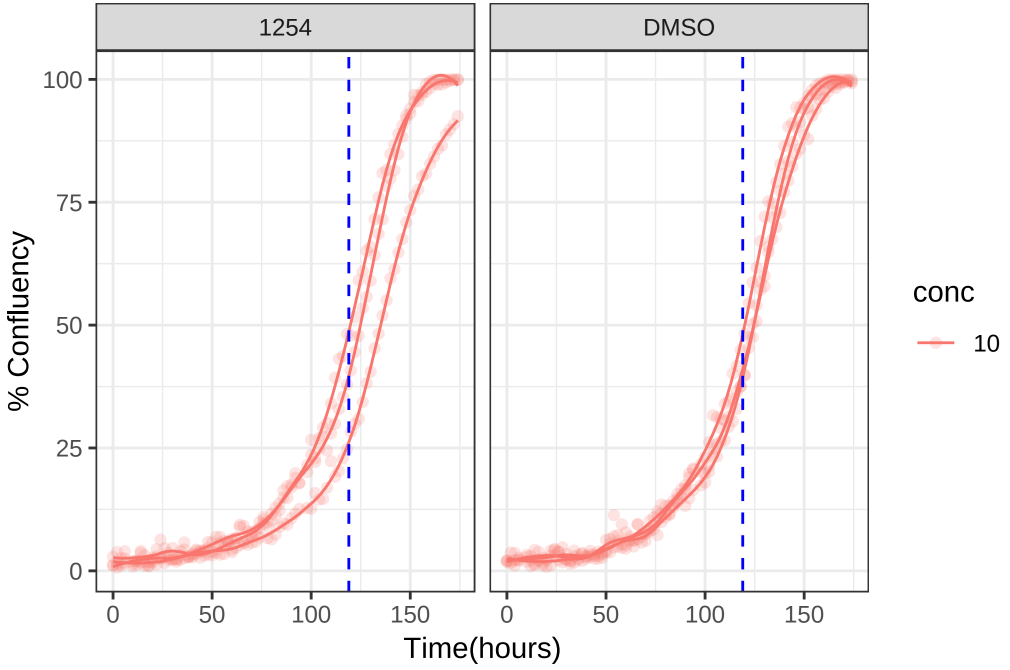

10 uM 1254 RPE1 Ts12 Ts5

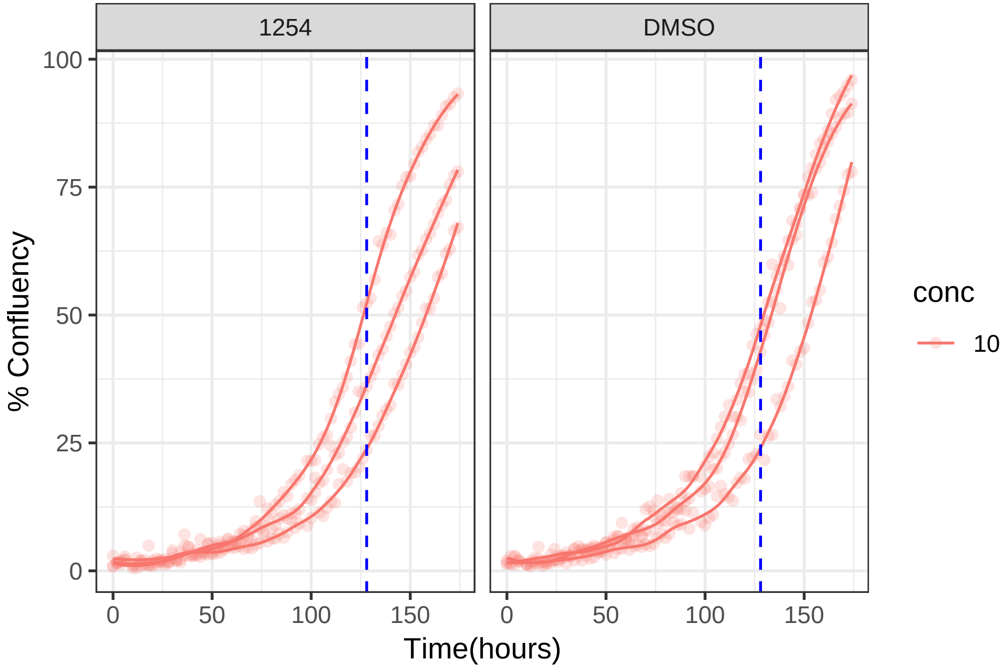

10 uM 1268 RPE1 hTert

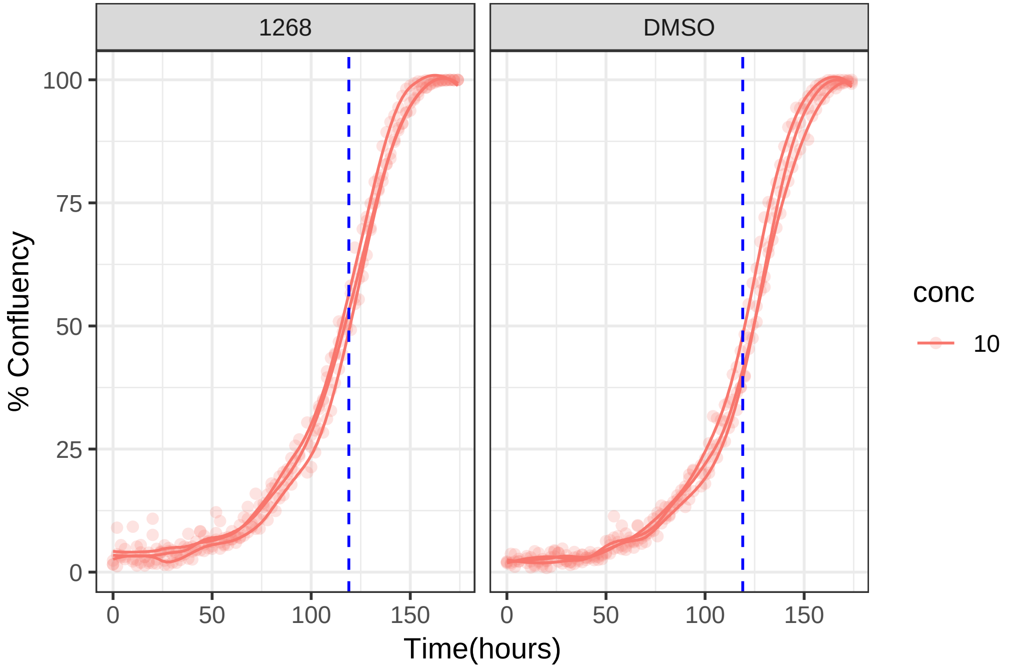

10 uM 1268 RPE1 Ts12 Ts5

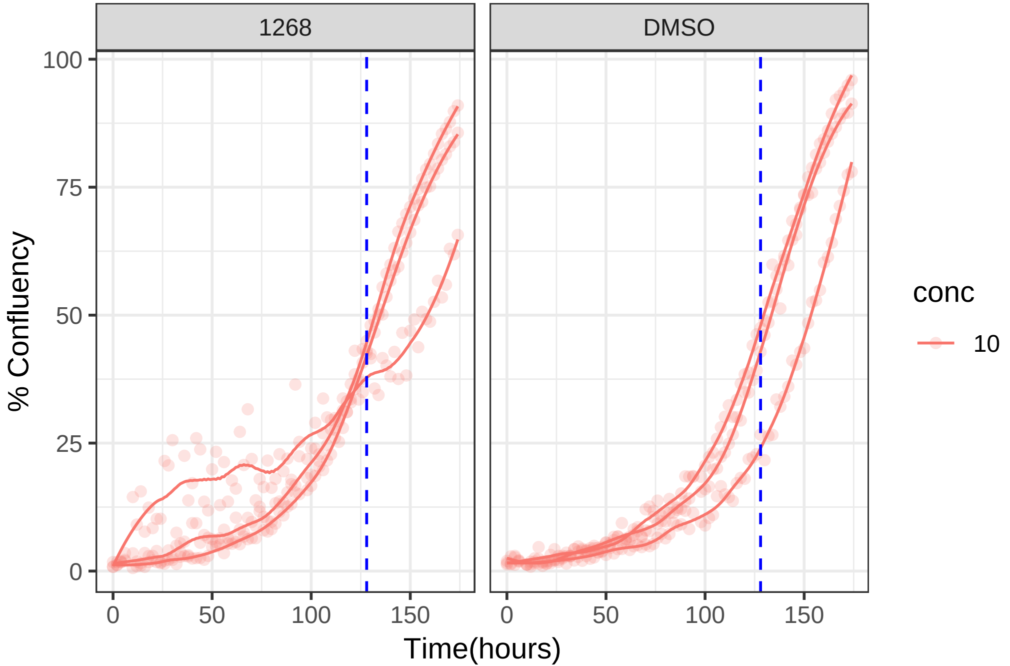

0.1 uM 1275 RPE1 hTert

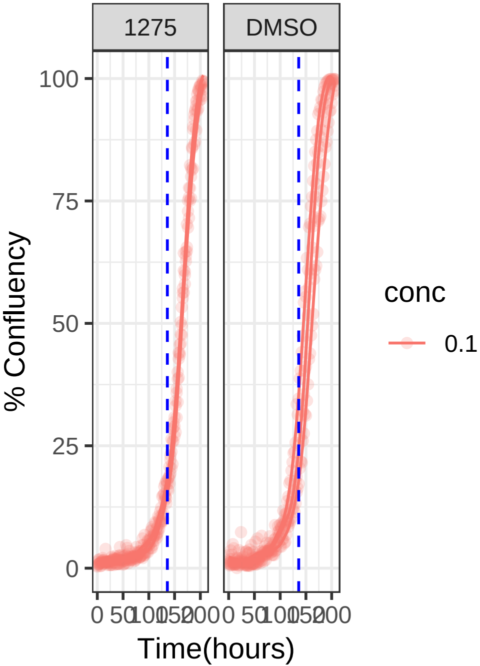

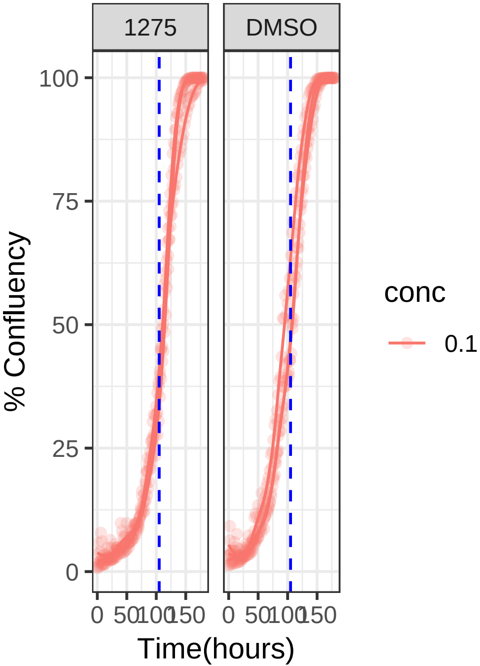

0.1 uM 1275 RPE1 Ts12 Ts5

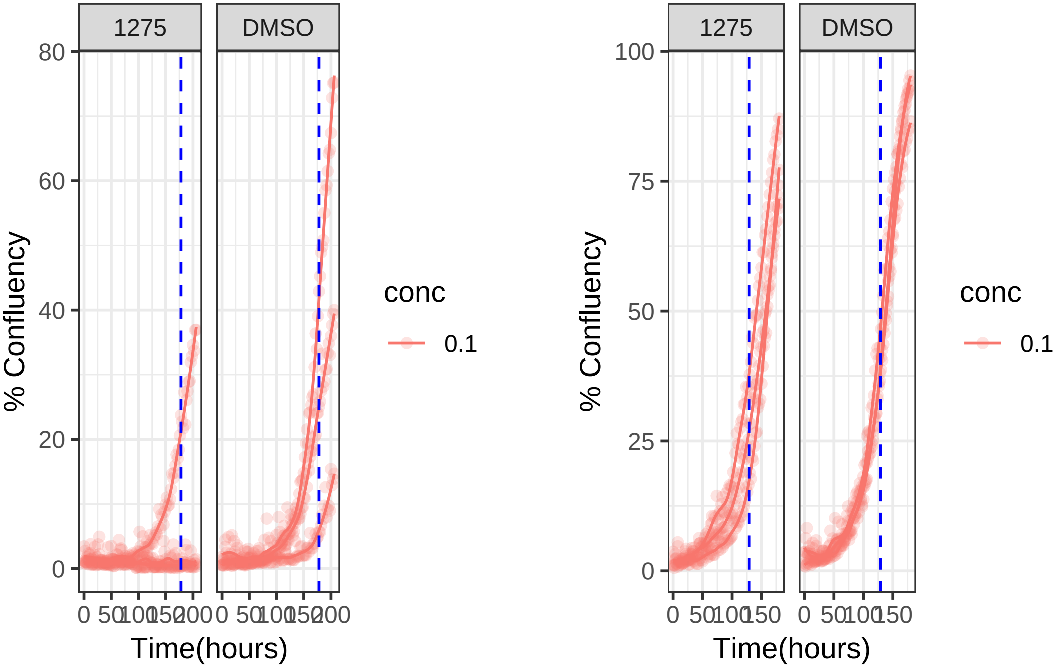

1 uM 1310 RPE1 hTert

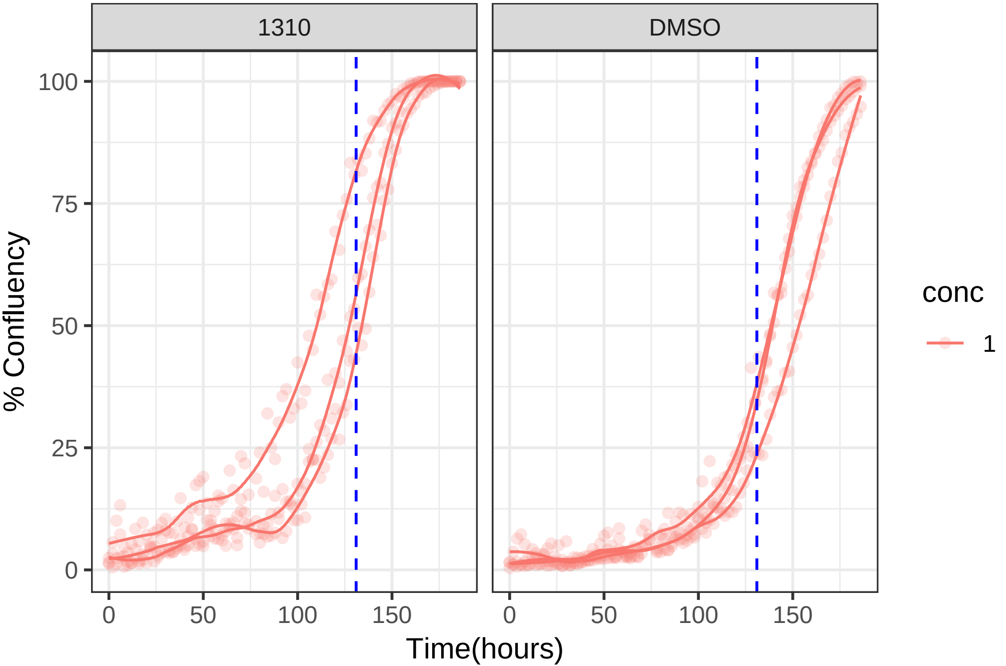

1 uM 1310 RPE1 Ts12 Ts5

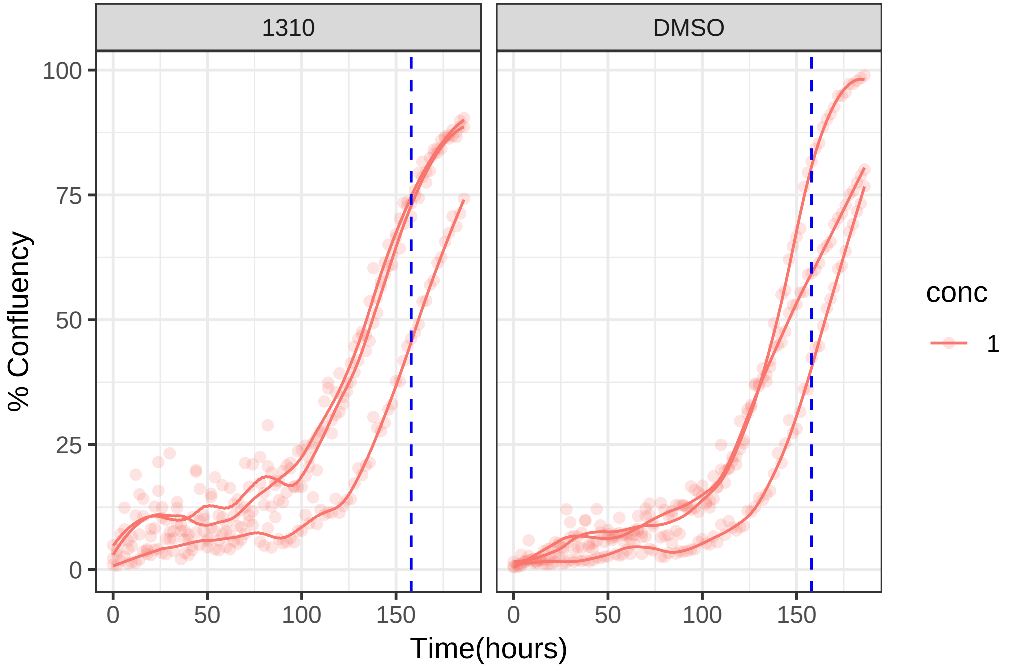

1 uM 1334 RPE1 hTert

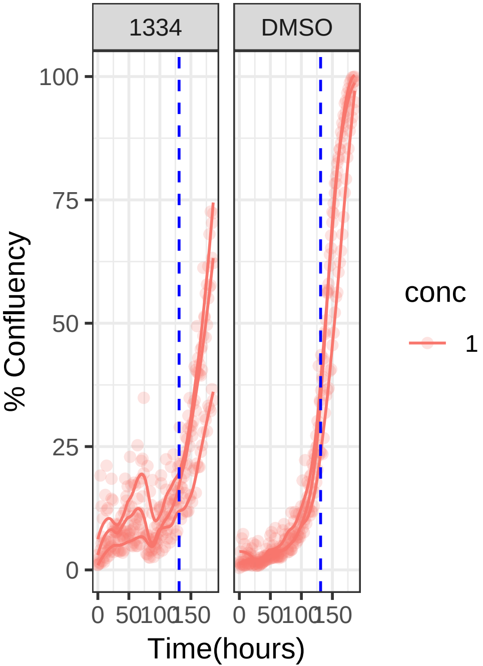

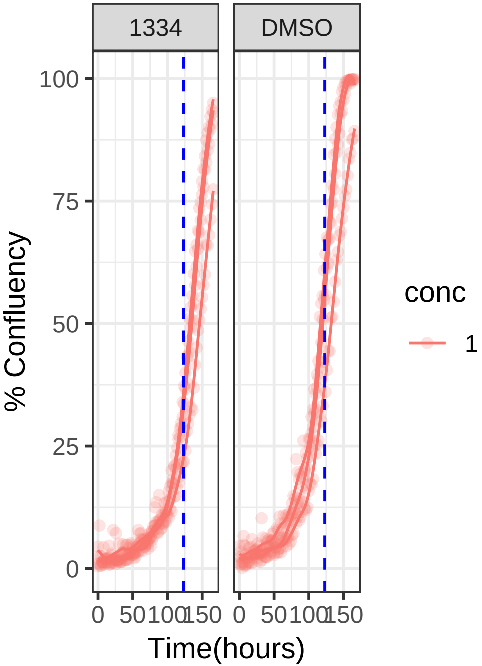

1 uM 1334 RPE1 Ts12 Ts5

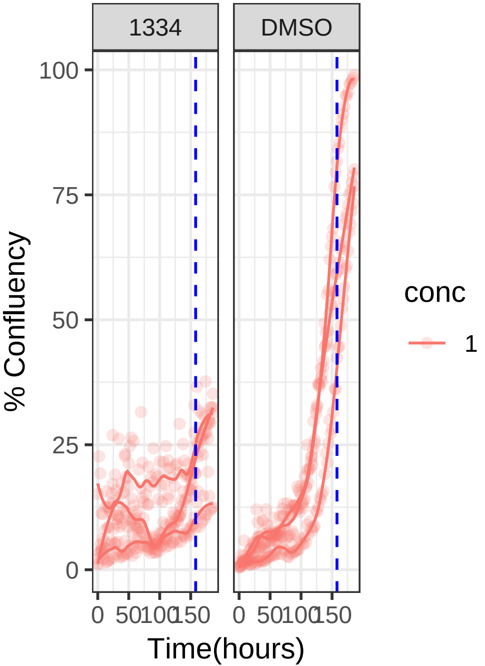

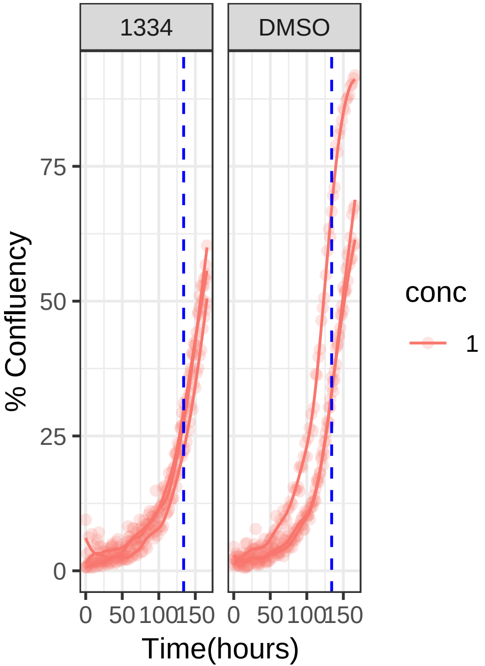

10 uM 1355 RPE1 hTert

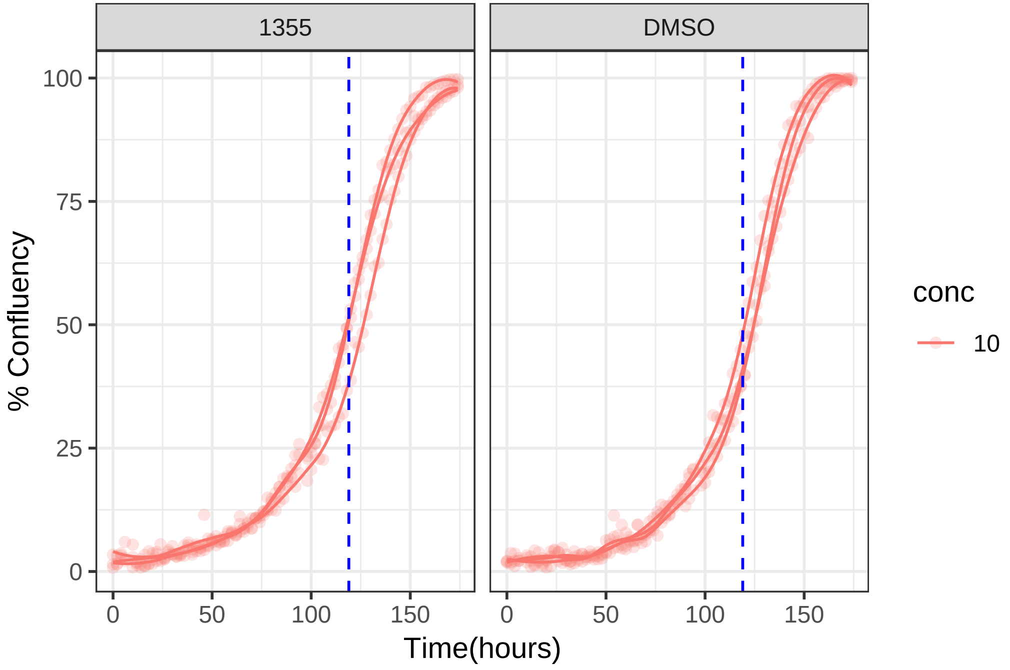

10 uM 1355 RPE1 Ts12 Ts5

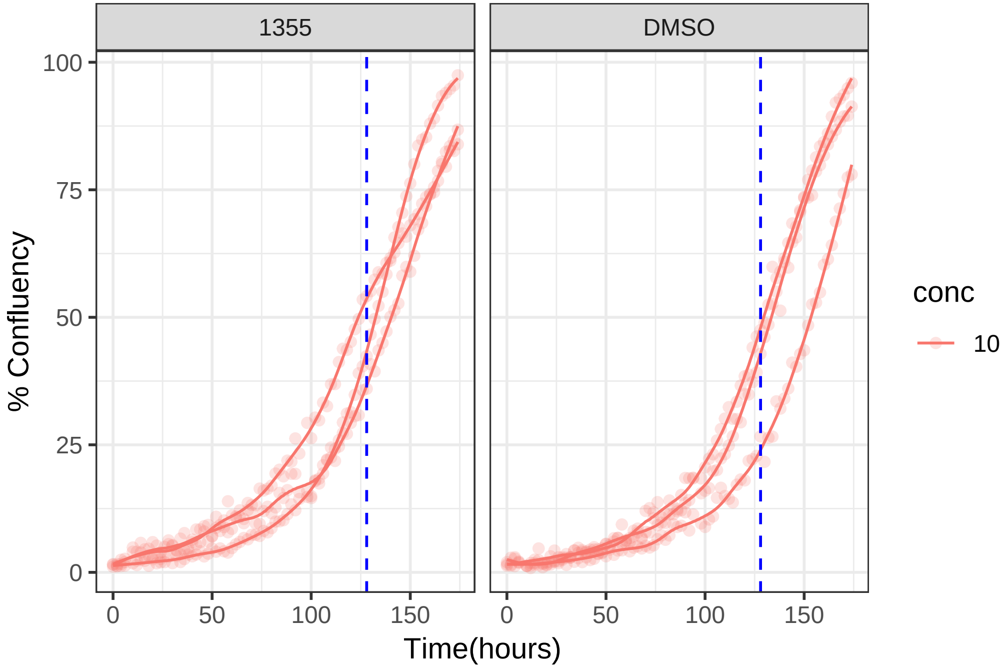

10 uM 1356 RPE1 hTert

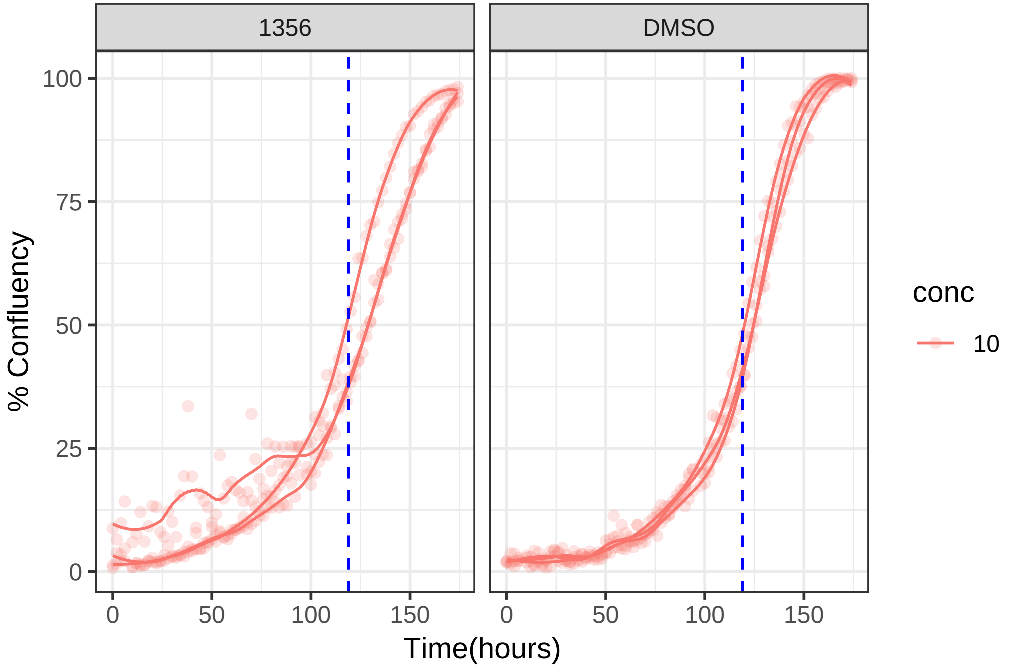

10 uM 1356 RPE1 Ts12 Ts5

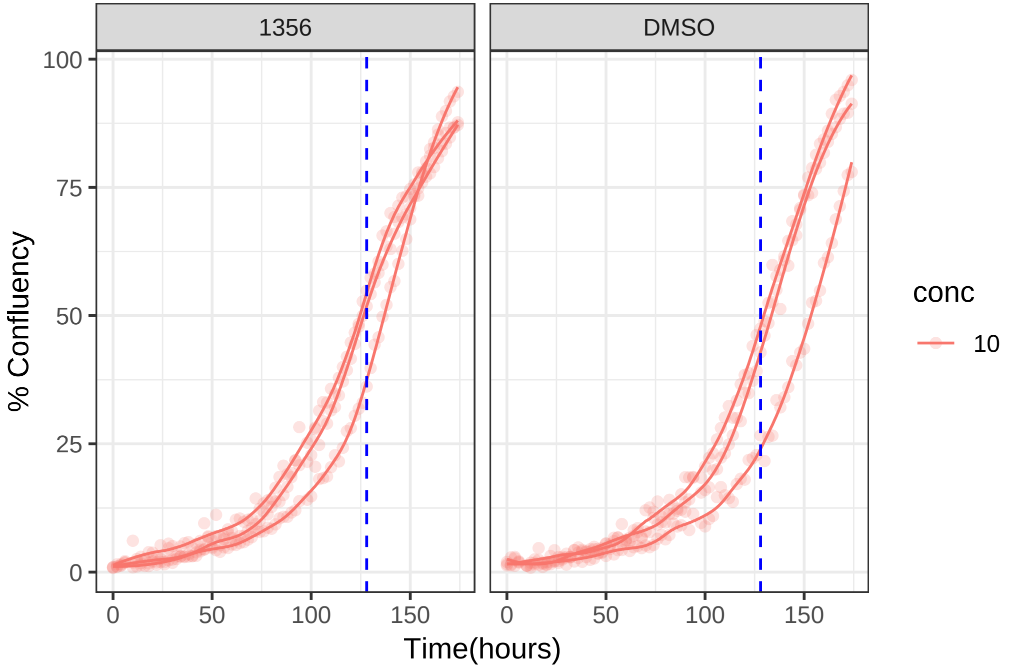

1 uM 1362 RPE1 hTert

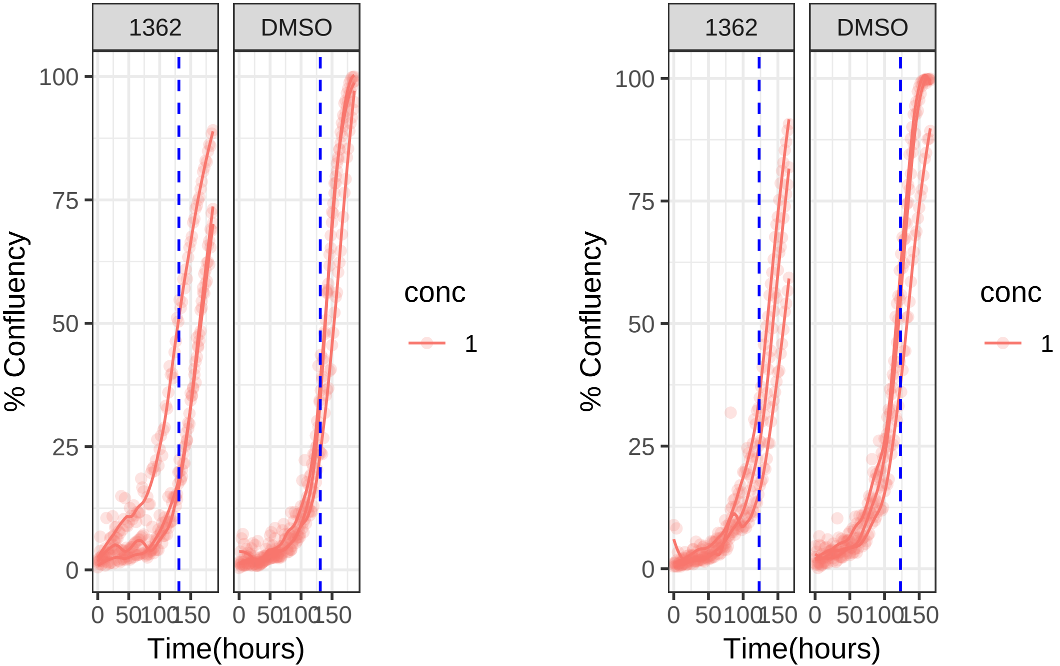

1 uM 1362 RPE1 Ts12 Ts5

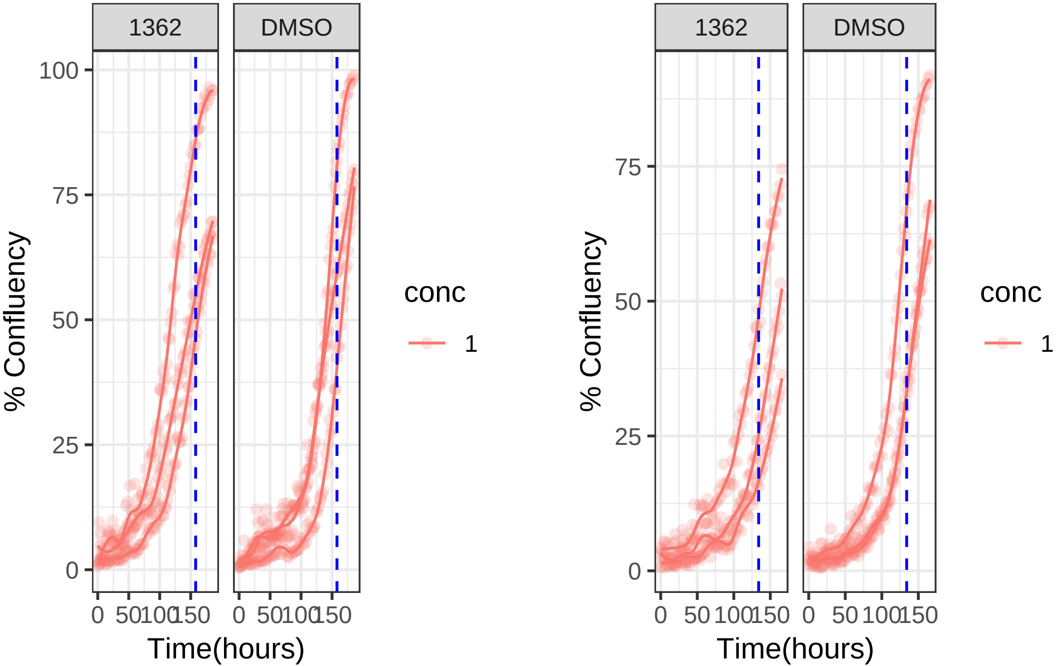

10 uM 1375 RPE1 hTert

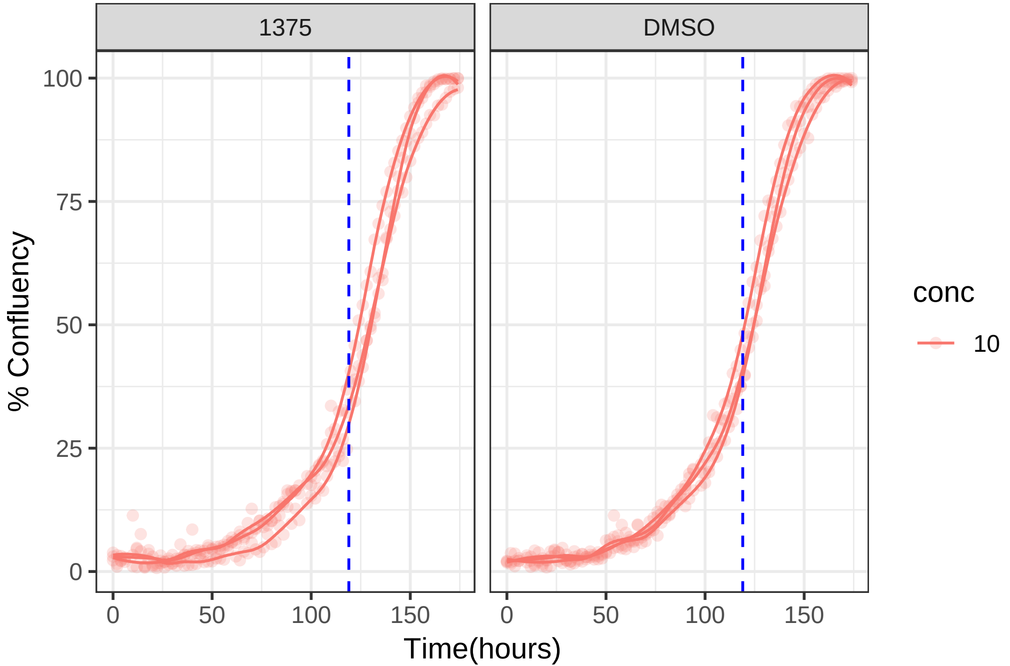

10 uM 1375 RPE1 Ts12 Ts5

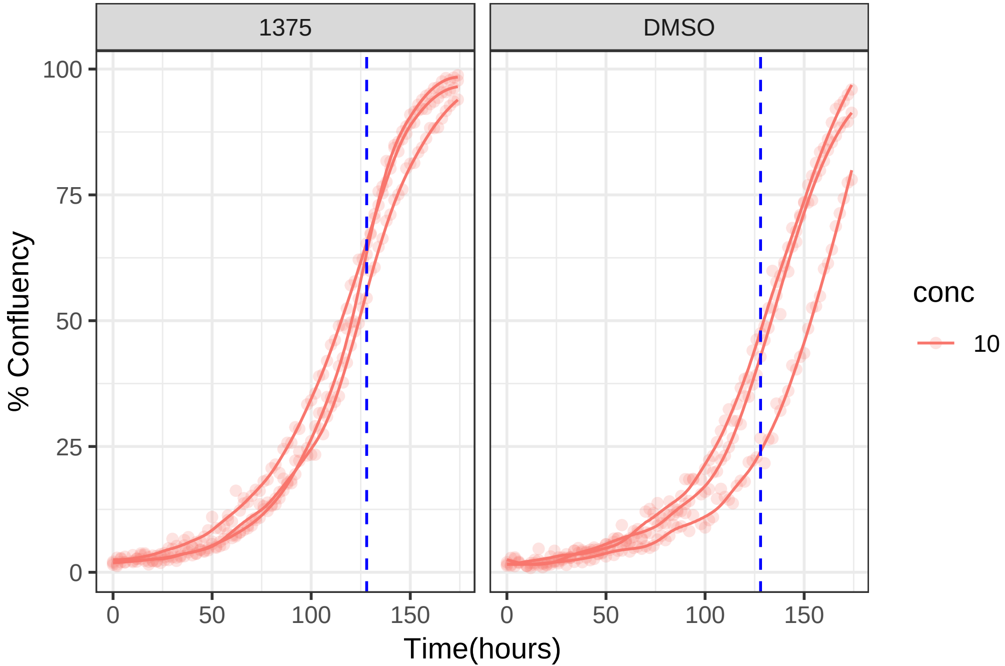

1 uM 1377 RPE1 hTert

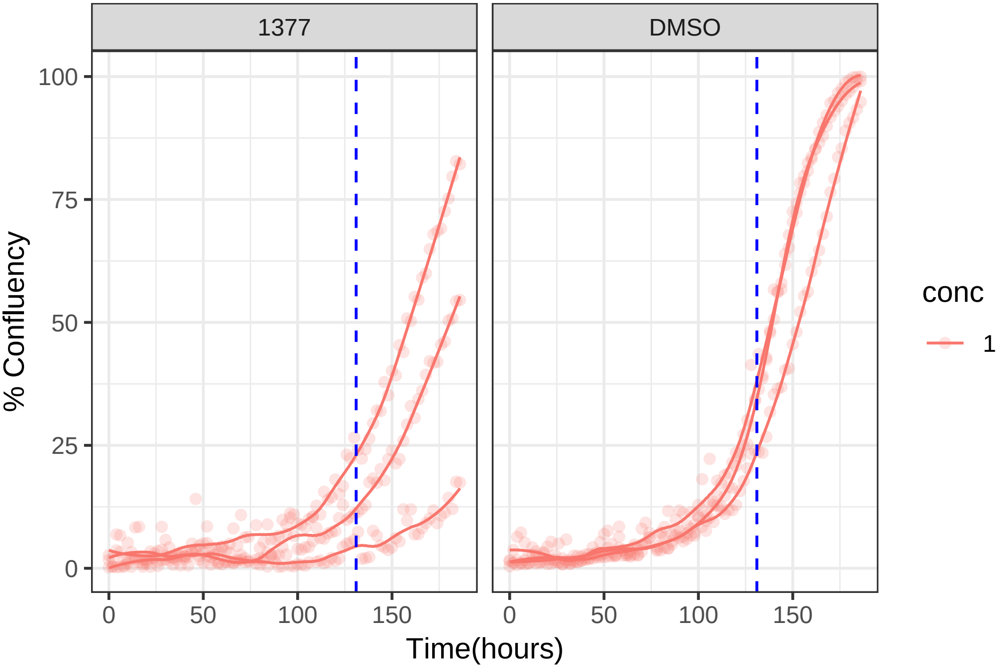

1 uM 1377 RPE1 Ts12 Ts5

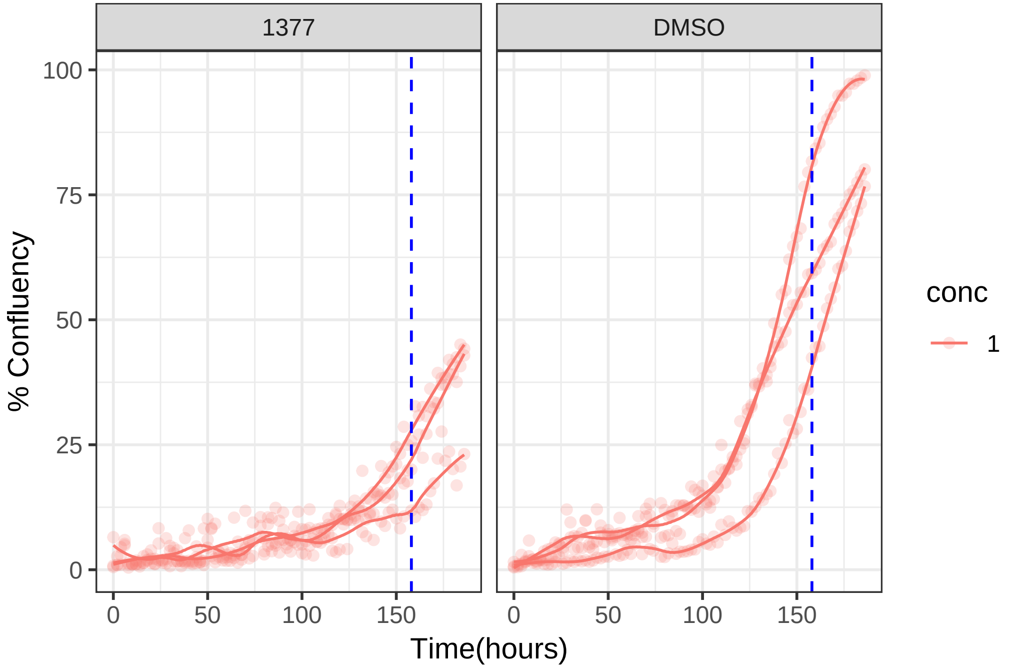

1 uM 1407 RPE1 hTert

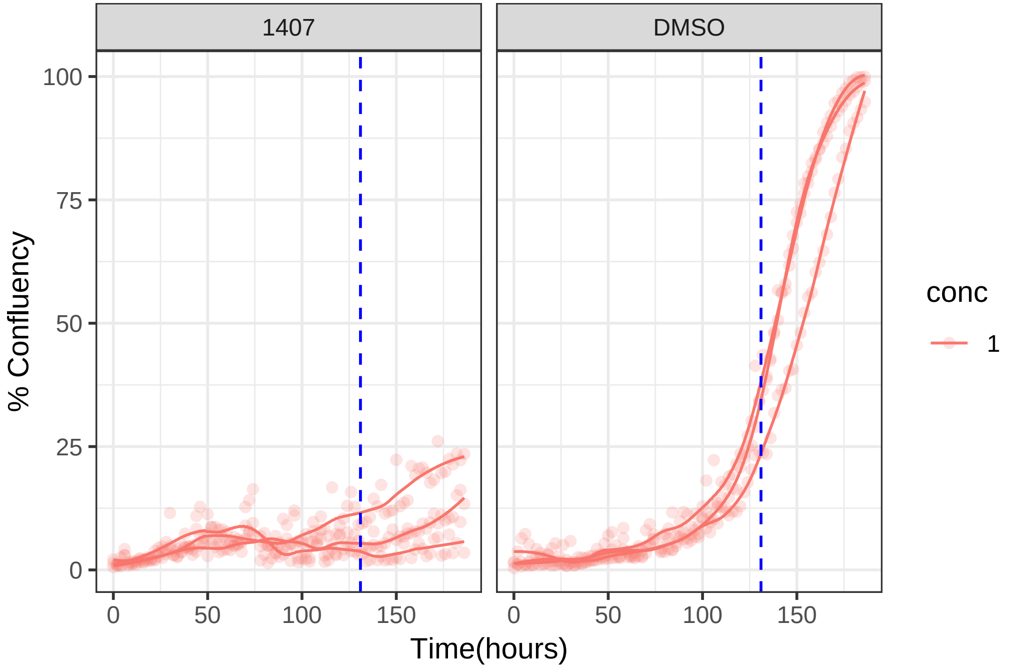

0.1 uM 1407 RPE1 hTert

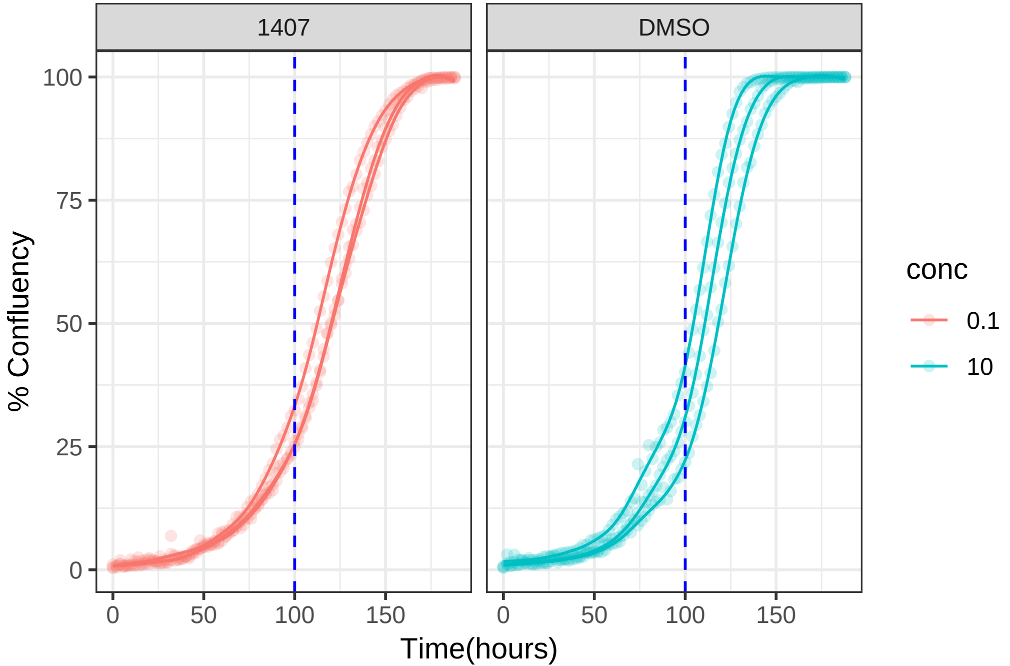

0.5 uM 1407 RPE1 hTert

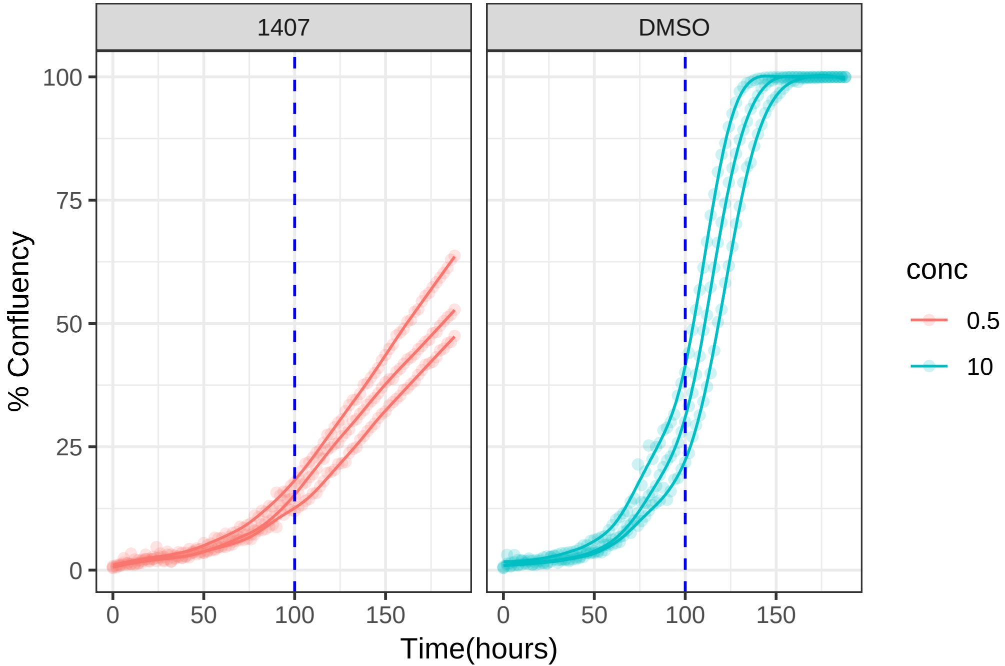

1 uM 1407 RPE1 Ts12 Ts5

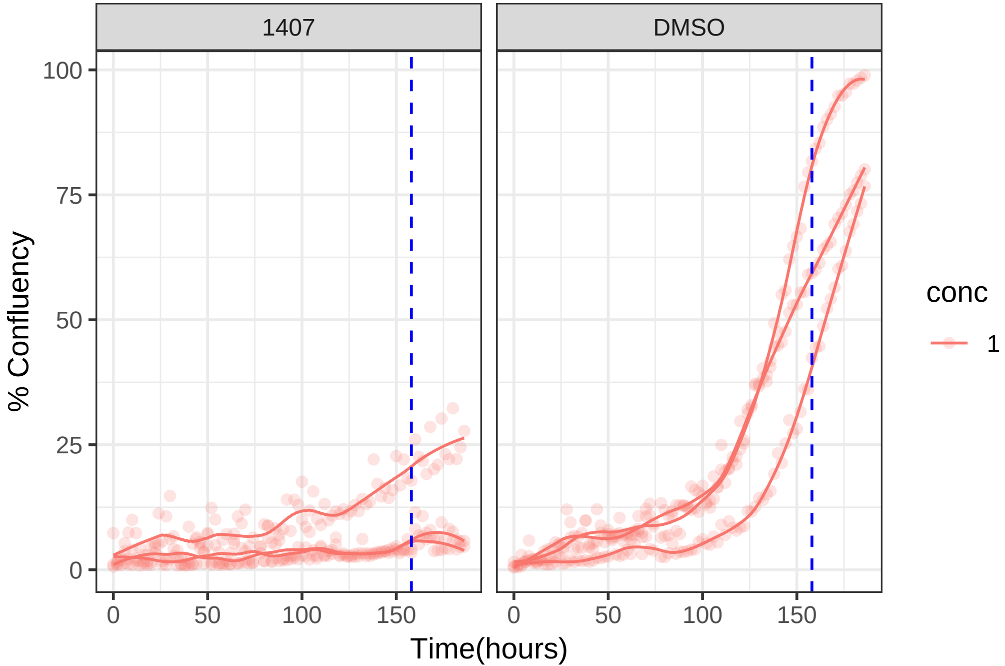

0.1 uM 1407 RPE1 Ts12 Ts5

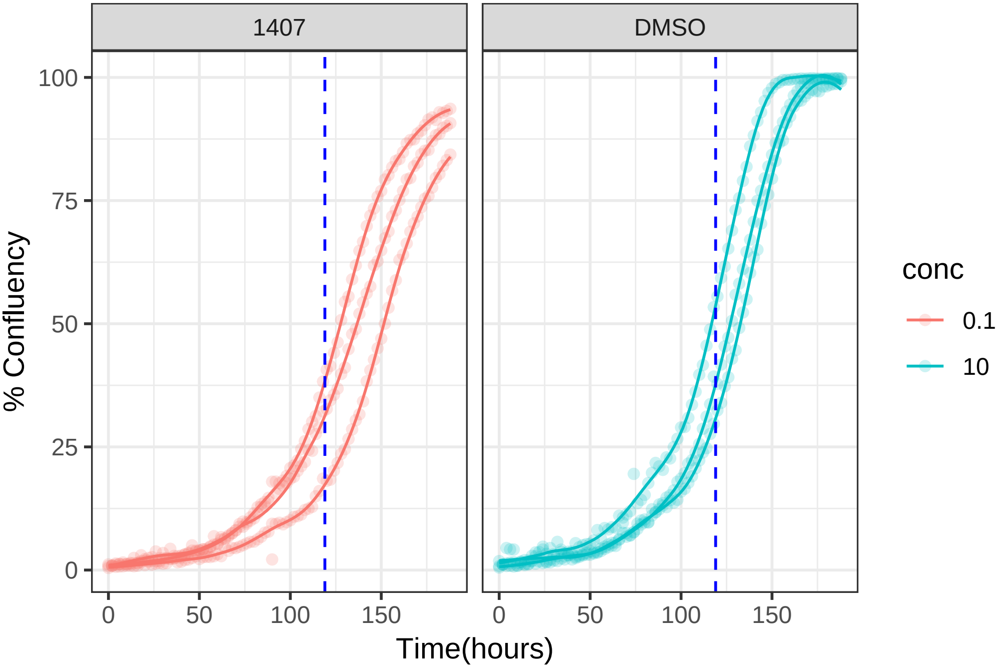

0.5 uM 1407 RPE1 Ts12 Ts5

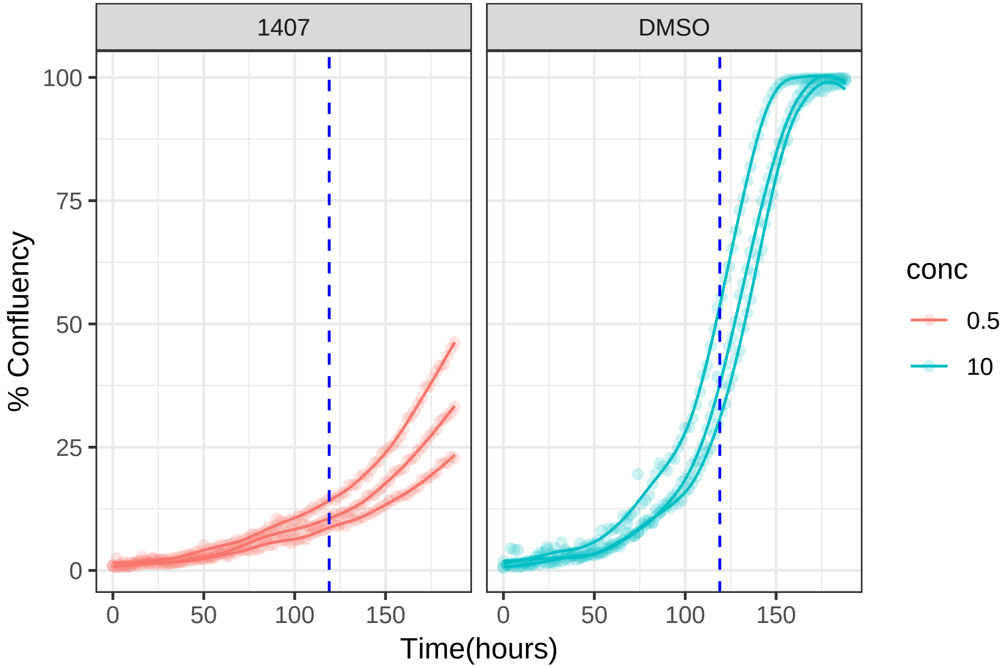

10 uM 1424 RPE1 hTert

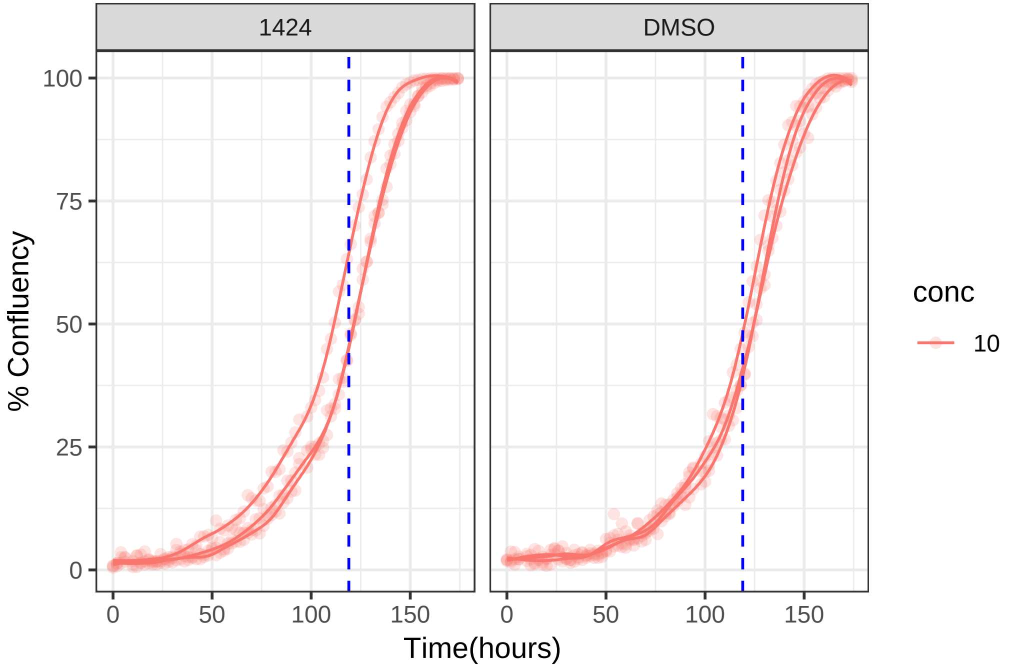

10 uM 1424 RPE1 Ts12 Ts5

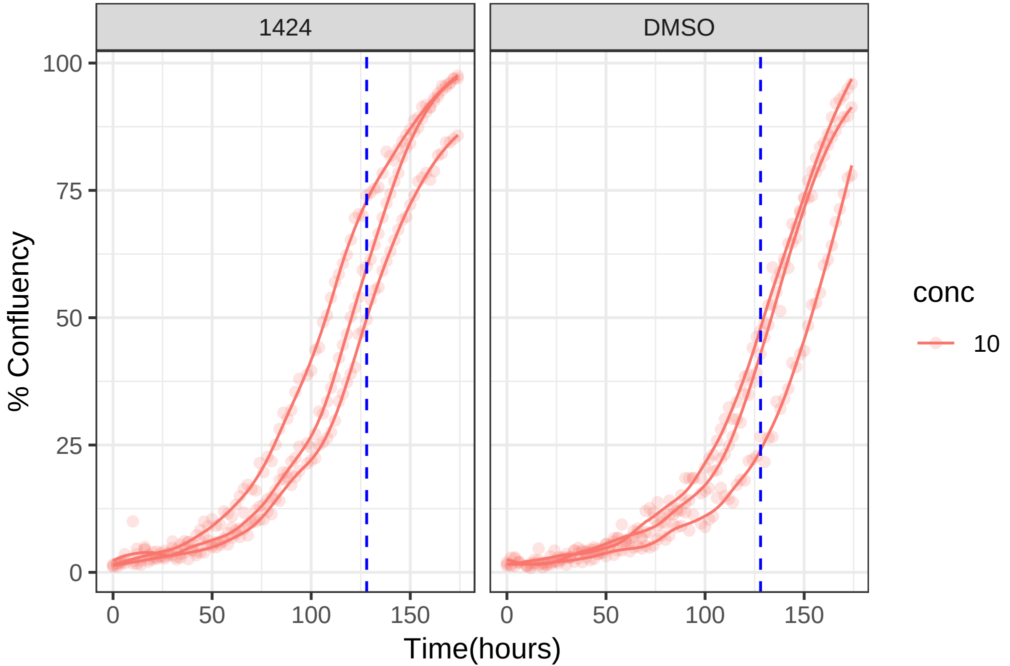

10 uM 1459 RPE1 hTert

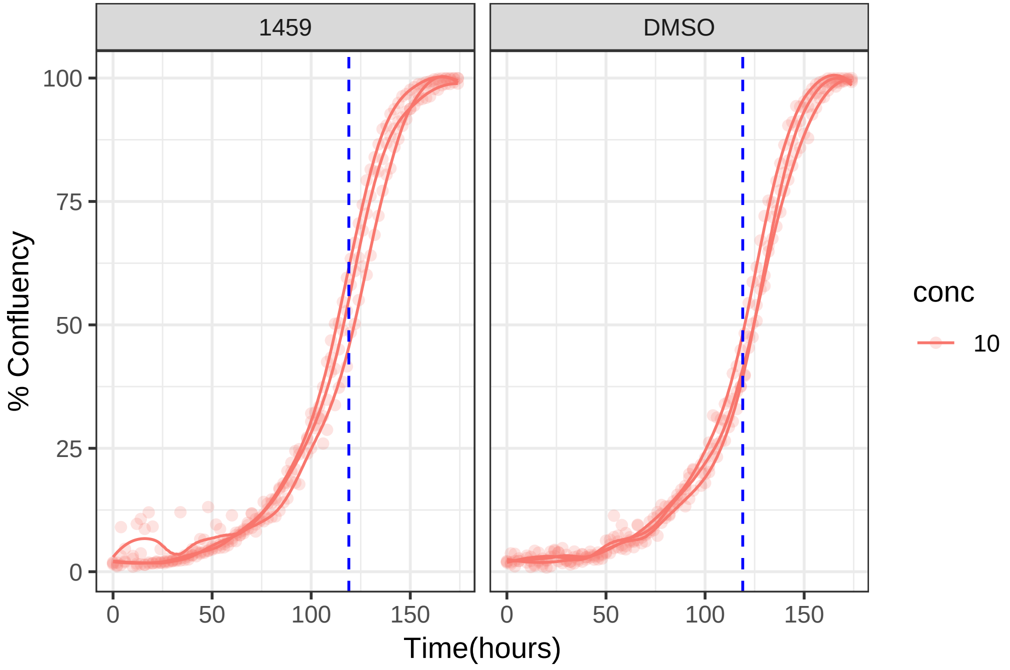

10 uM 1459 RPE1 Ts12 Ts5

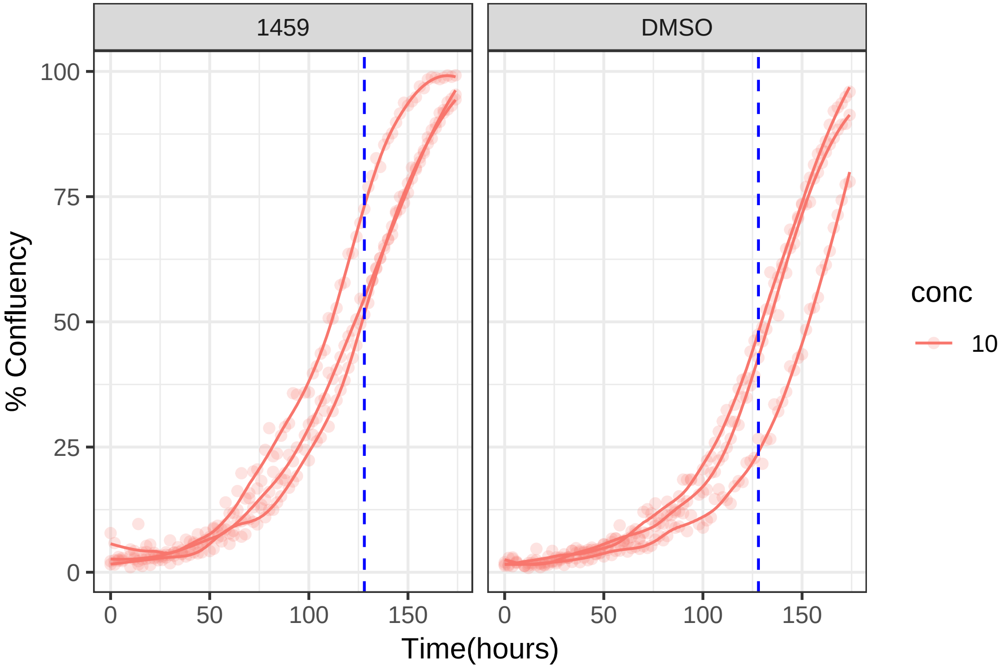

1 uM 1463 RPE1 hTert

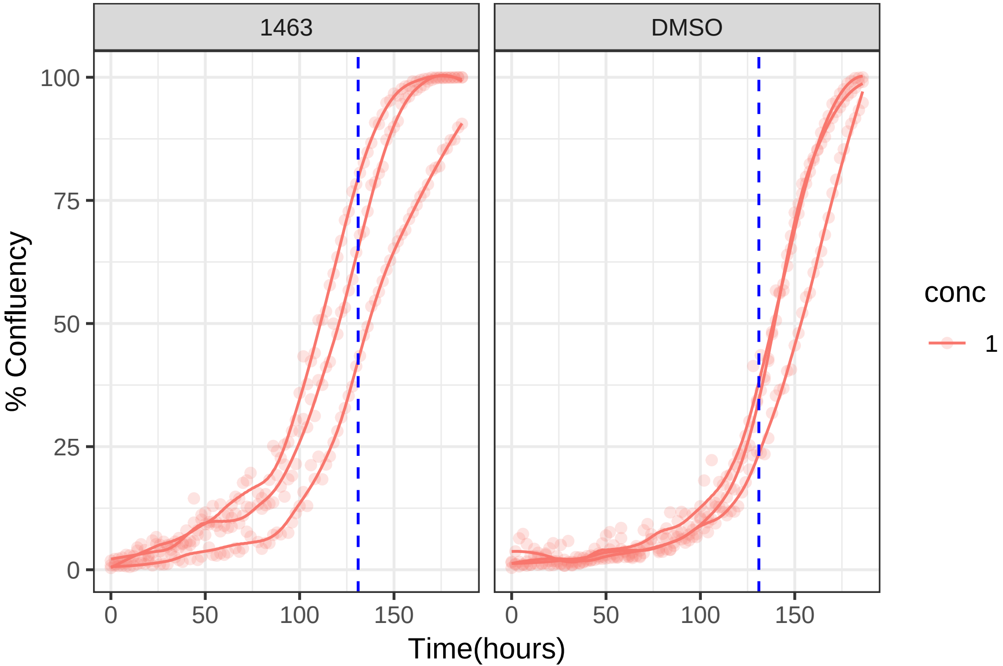

1 uM 1463 RPE1 Ts12 Ts5

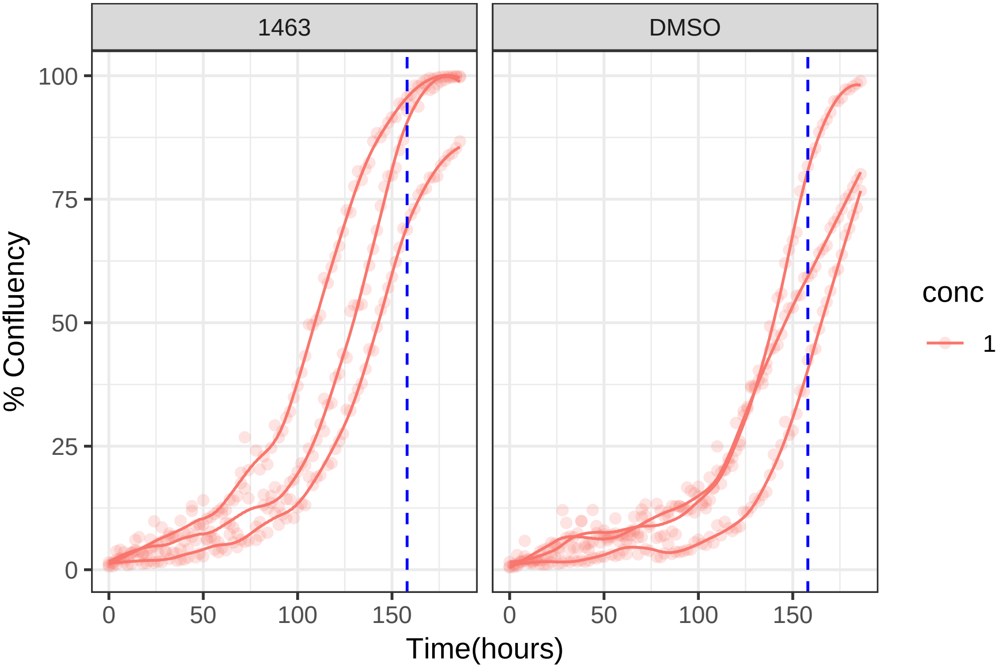

1 uM 1472 RPE1 hTert

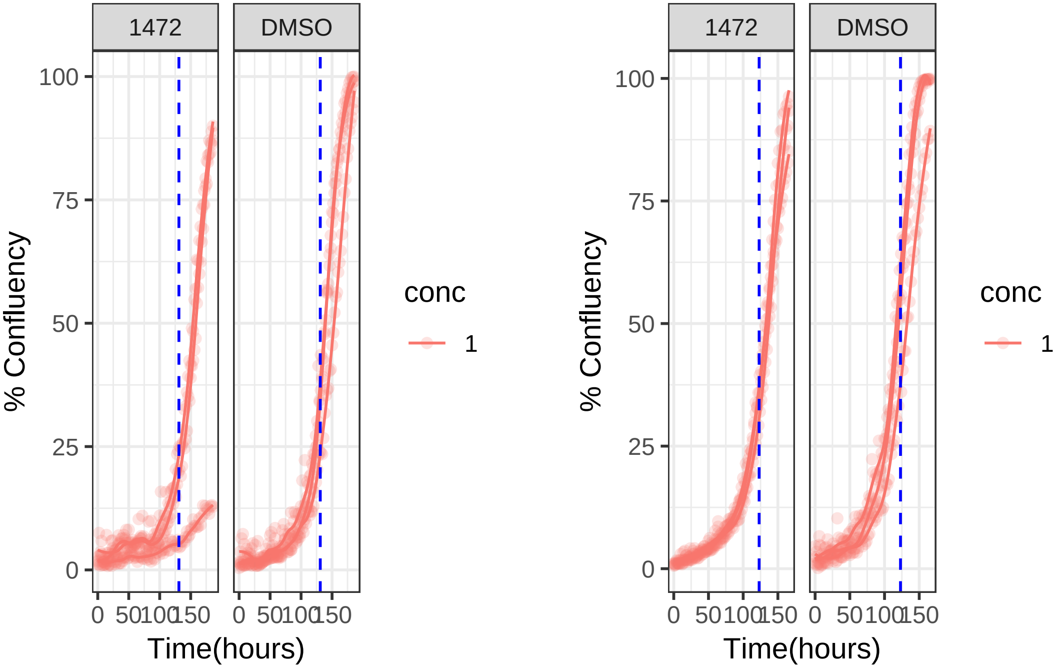

1 uM 1472 RPE1 Ts12 Ts5

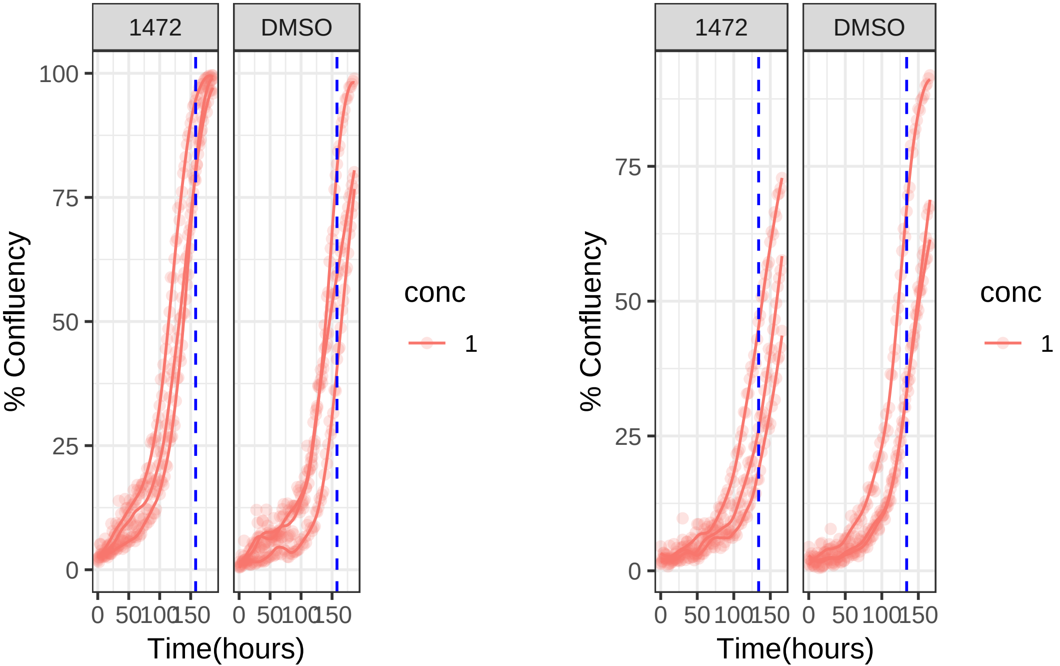

0.1 uM 1494 RPE1 hTert

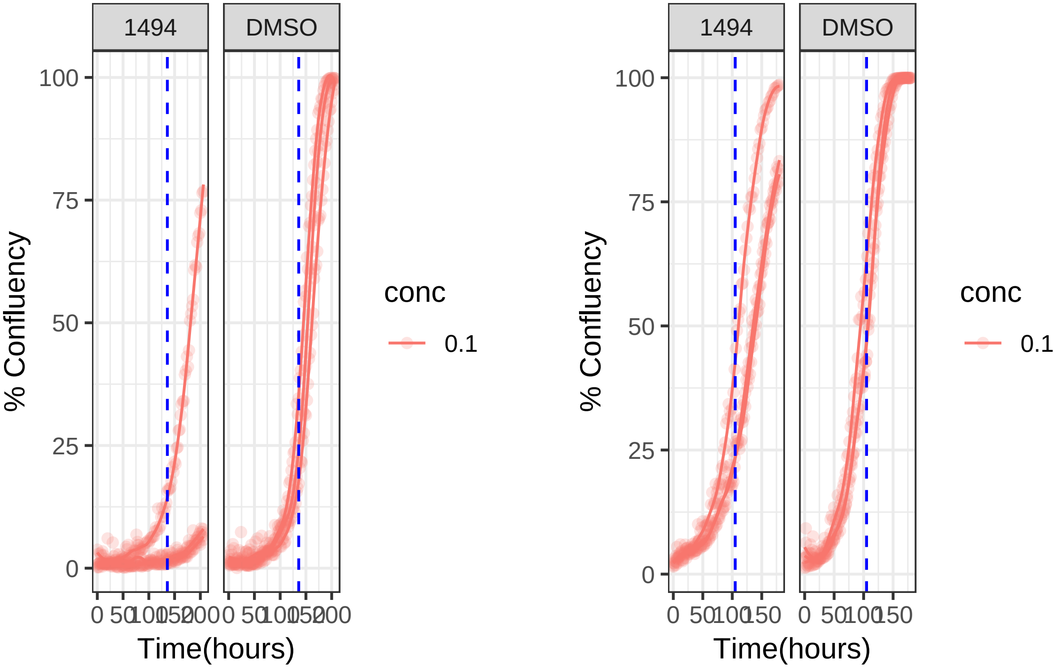

0.1 uM 1494 RPE1 Ts12 Ts5

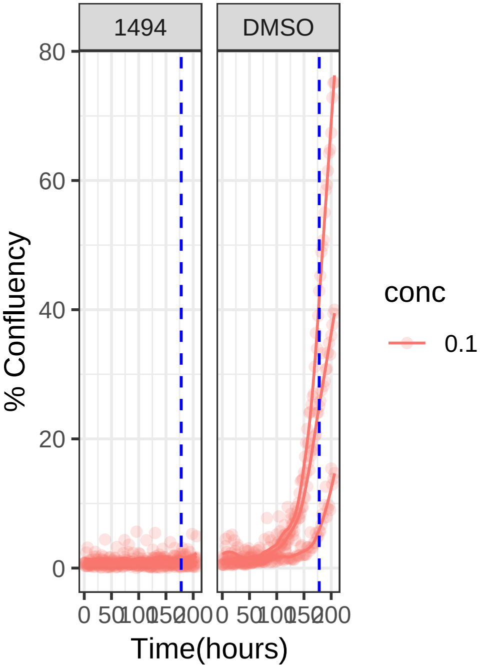

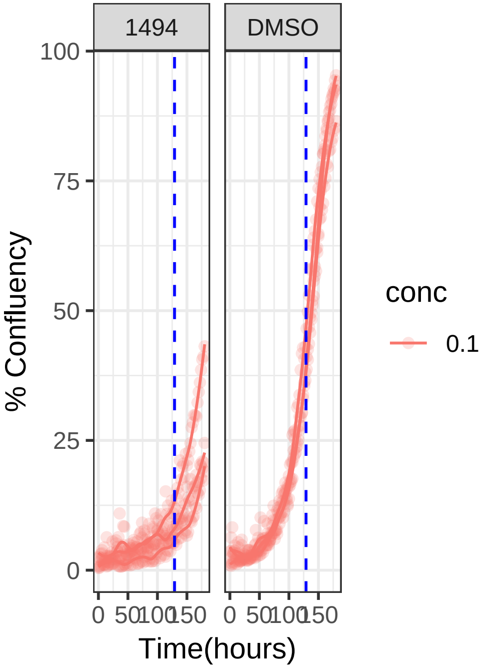

1 uM 1516 RPE1 hTert

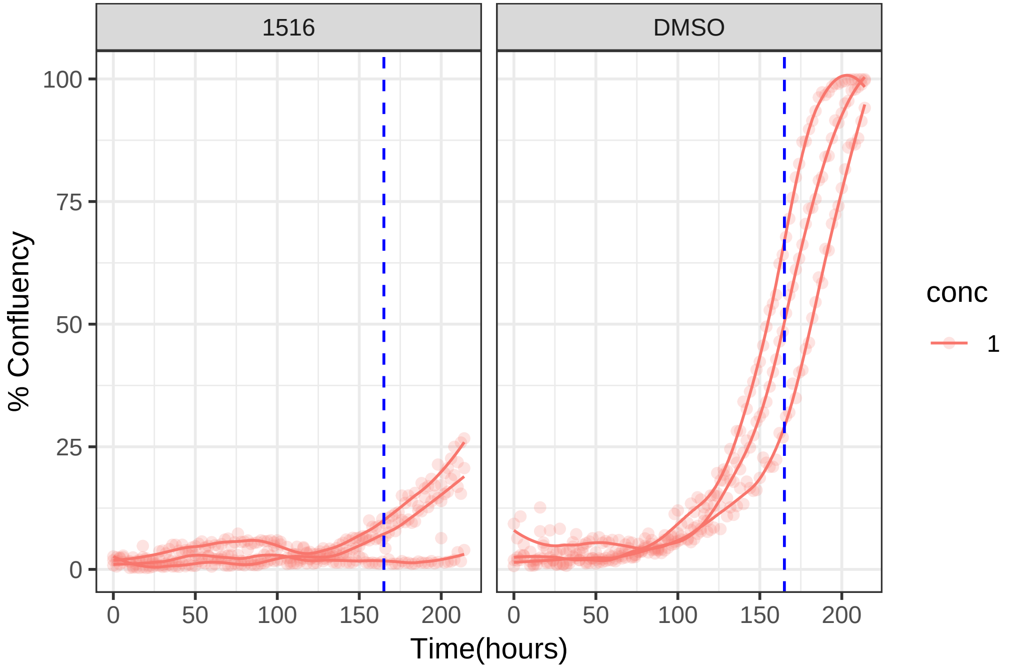

1 uM 1516 RPE1 Ts12 Ts5

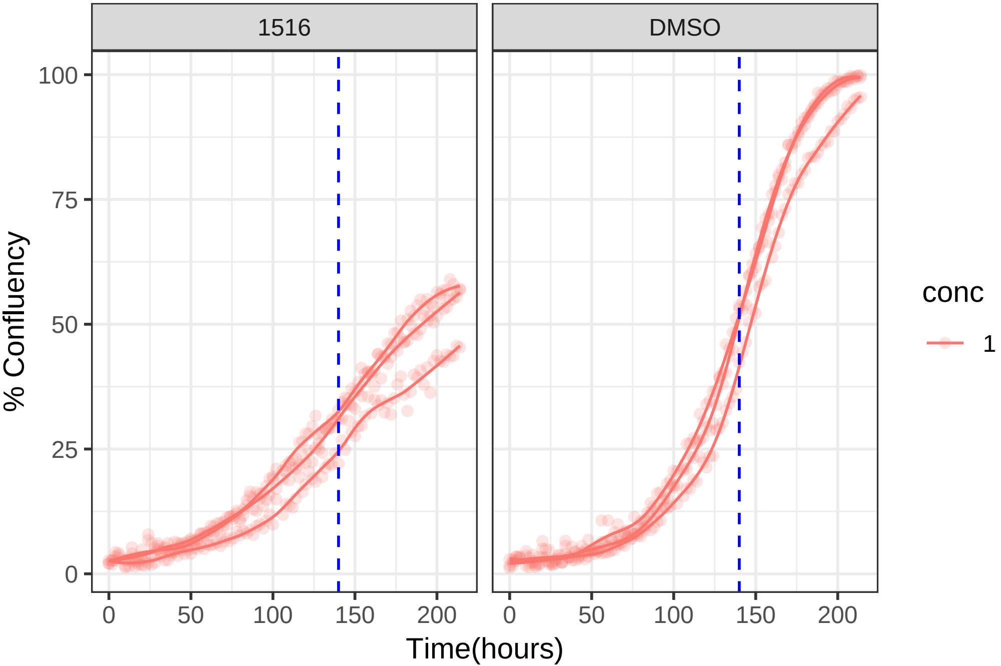

0.1 uM 1520 RPE1 hTert

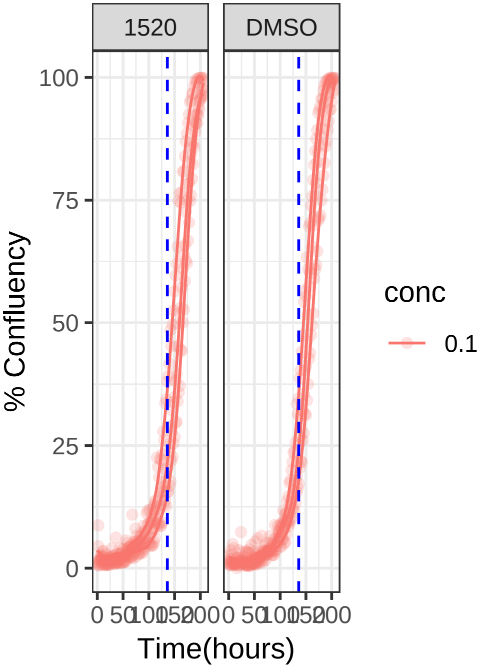

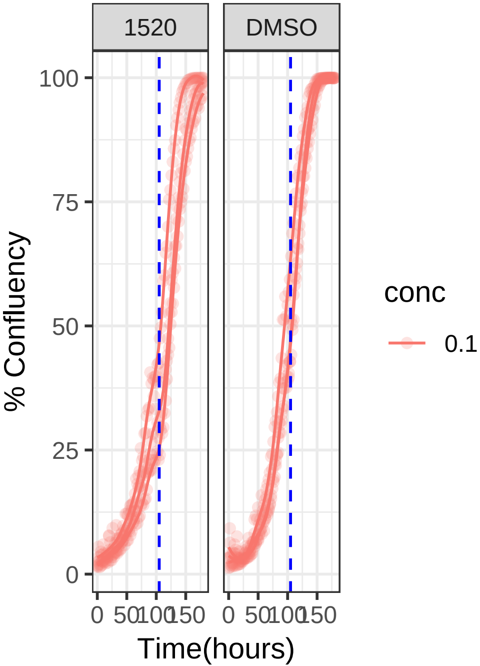

0.1 uM 1520 RPE1 Ts12 Ts5

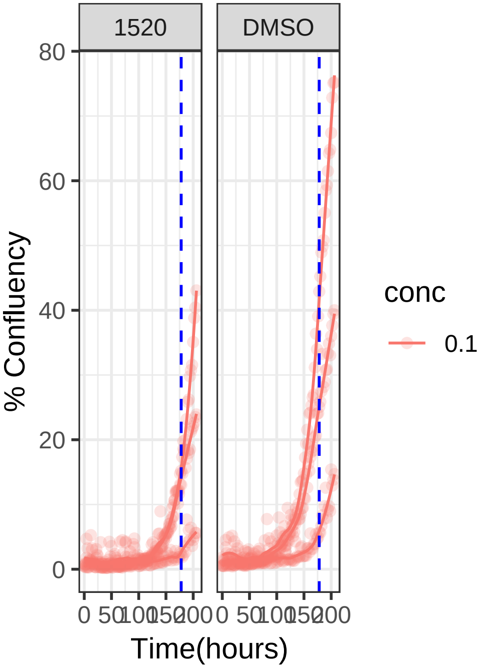

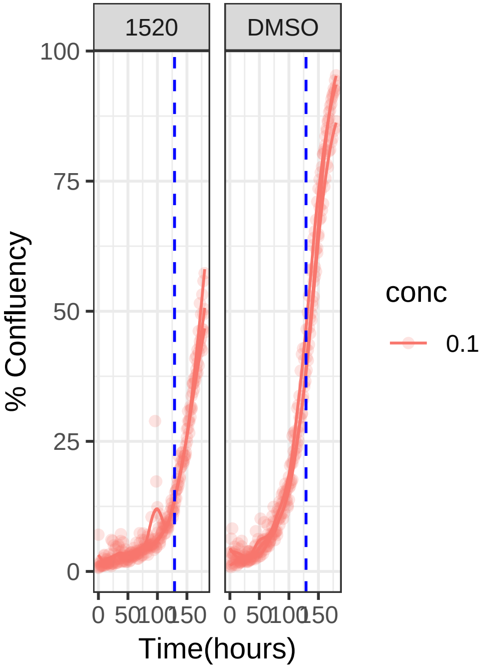

0.01 uM 1561 RPE1 hTert

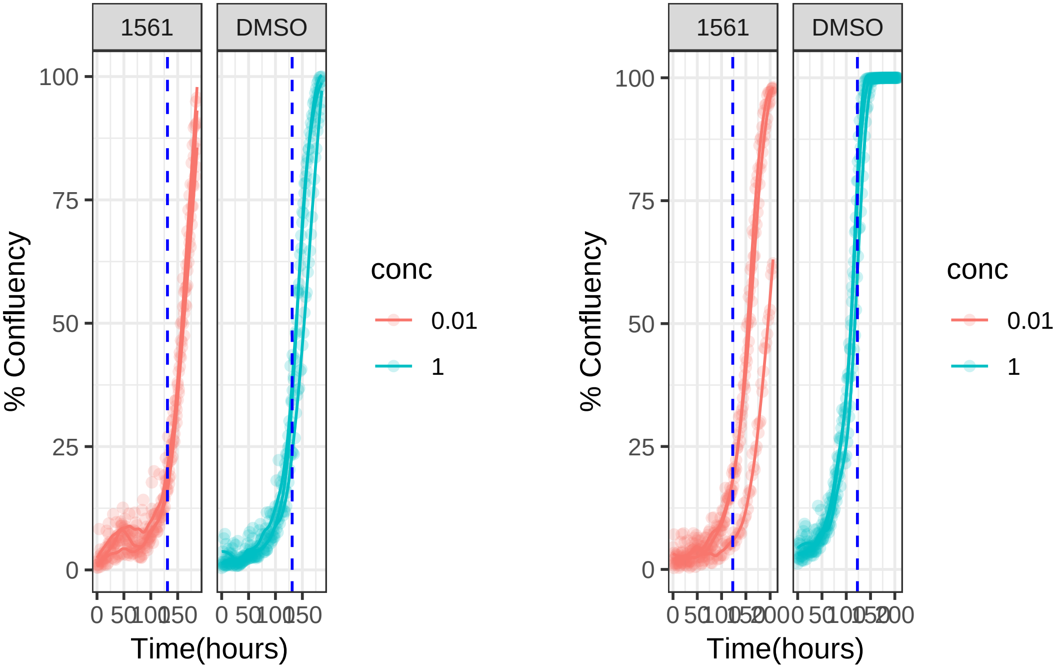

0.1 uM 1561 RPE1 hTert

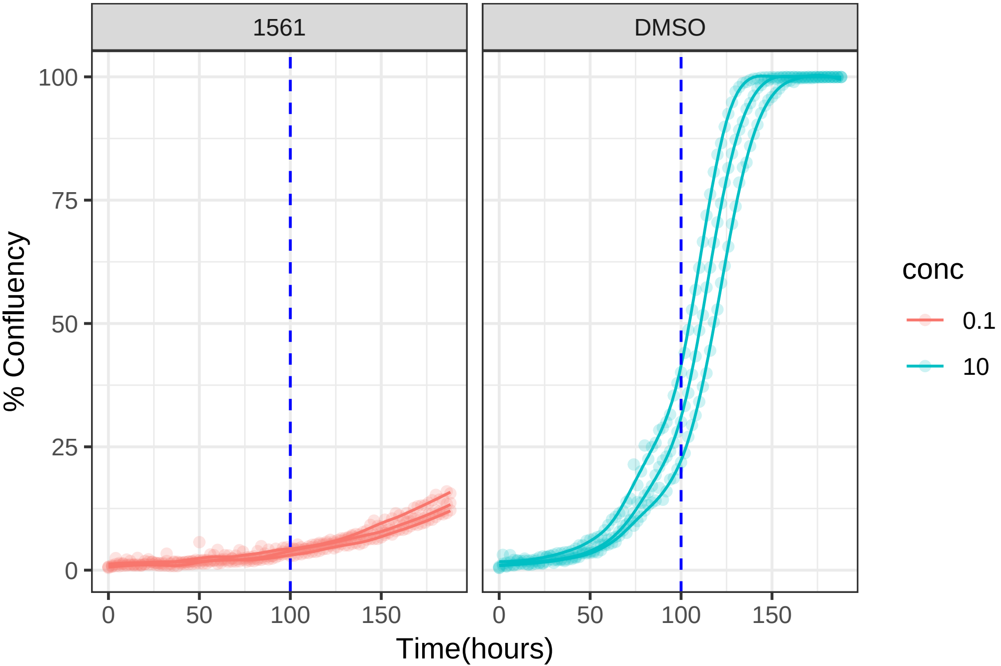

0.01 uM 1561 RPE1 Ts12 Ts5

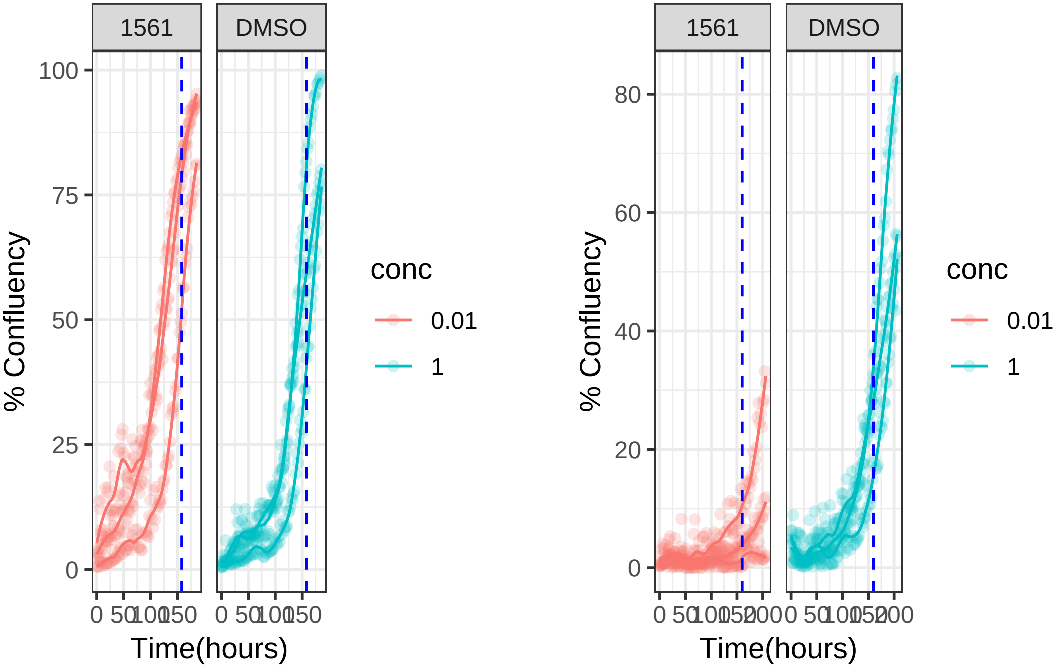

0.1 uM 1561 RPE1 Ts12 Ts5

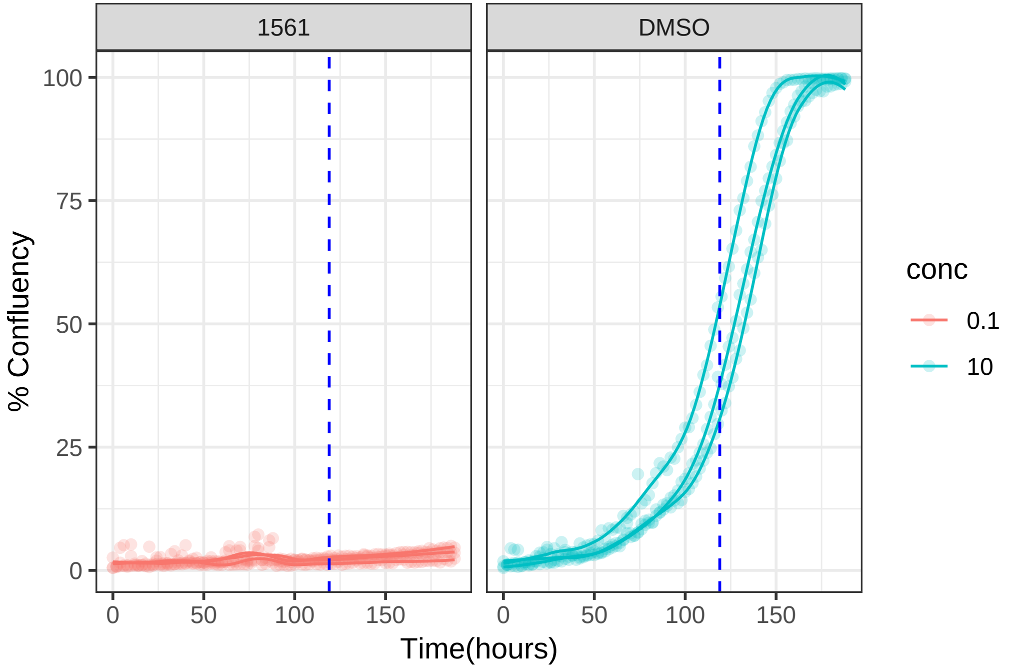

10 uM 1566 RPE1 hTert

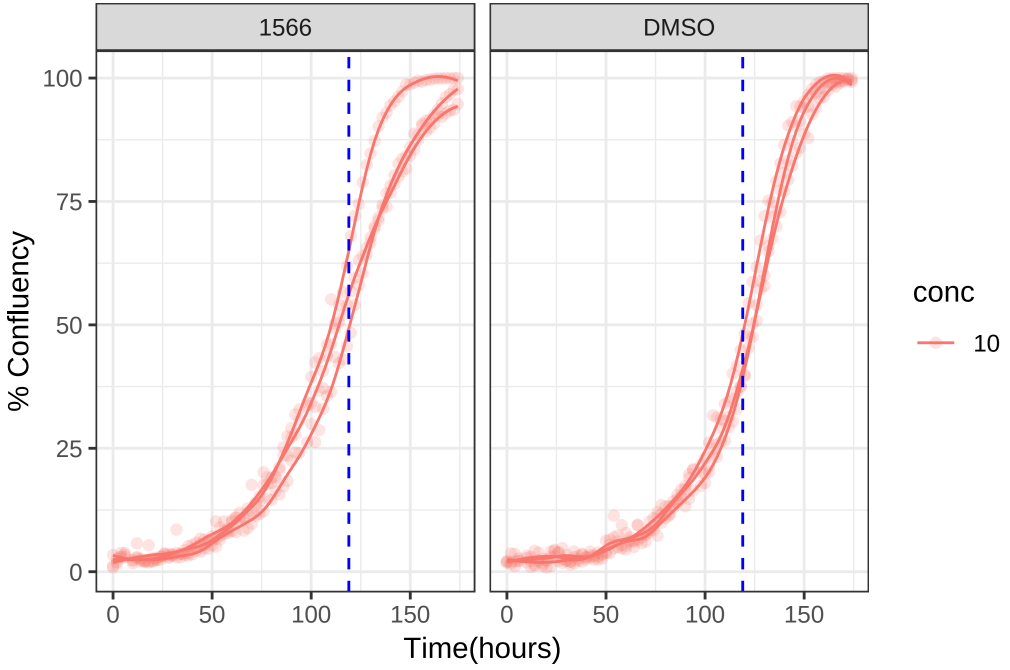

10 uM 1566 RPE1 Ts12 Ts5

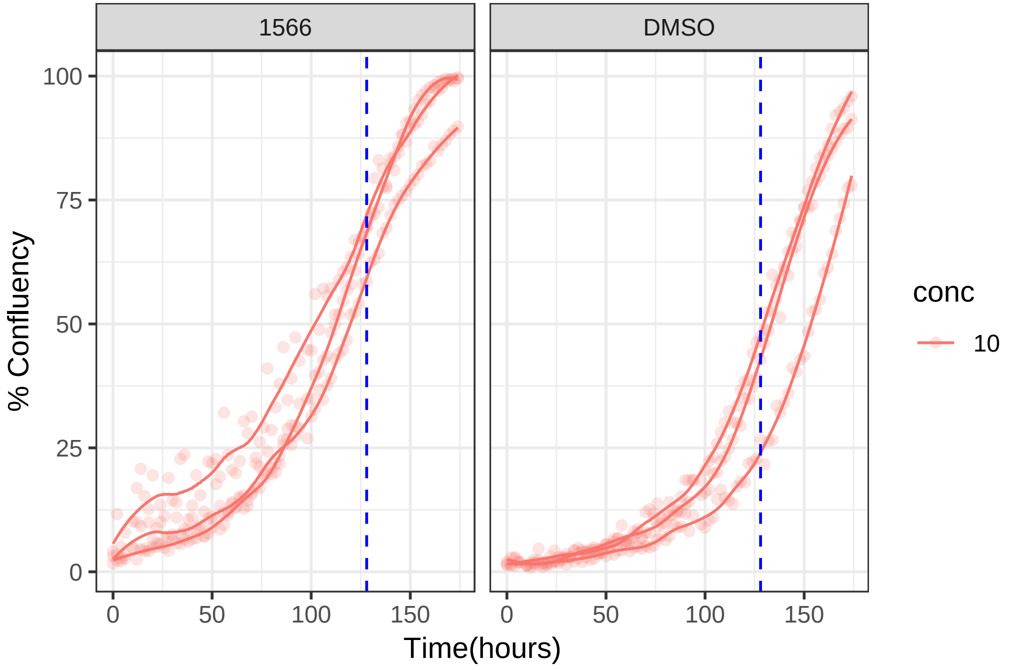

0.1 uM 1596 RPE1 hTert

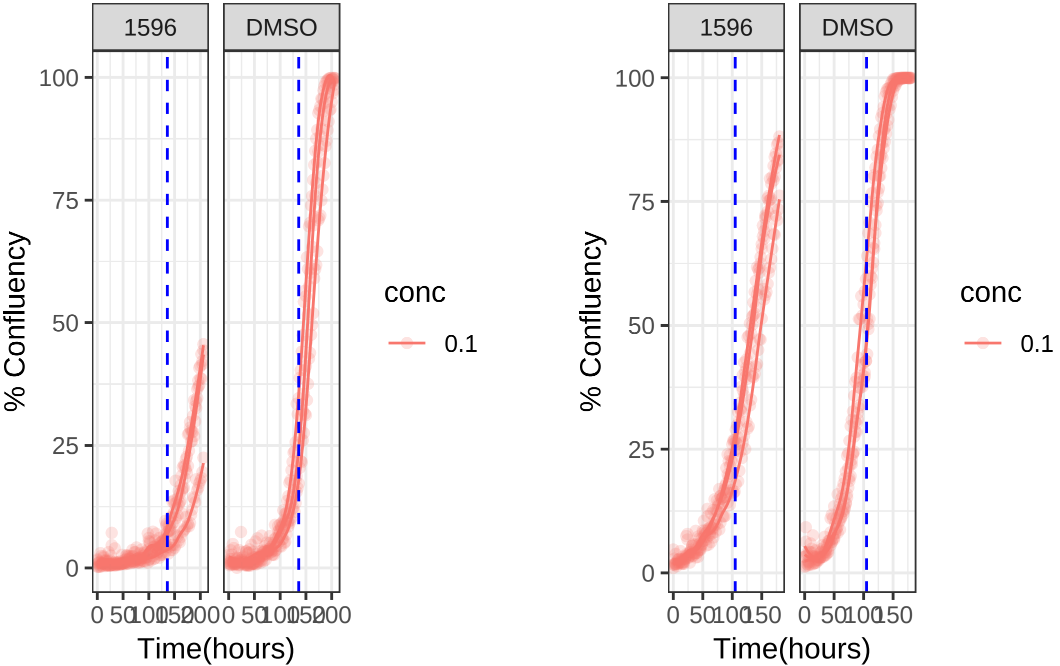

0.1 uM 1596 RPE1 Ts12 Ts5

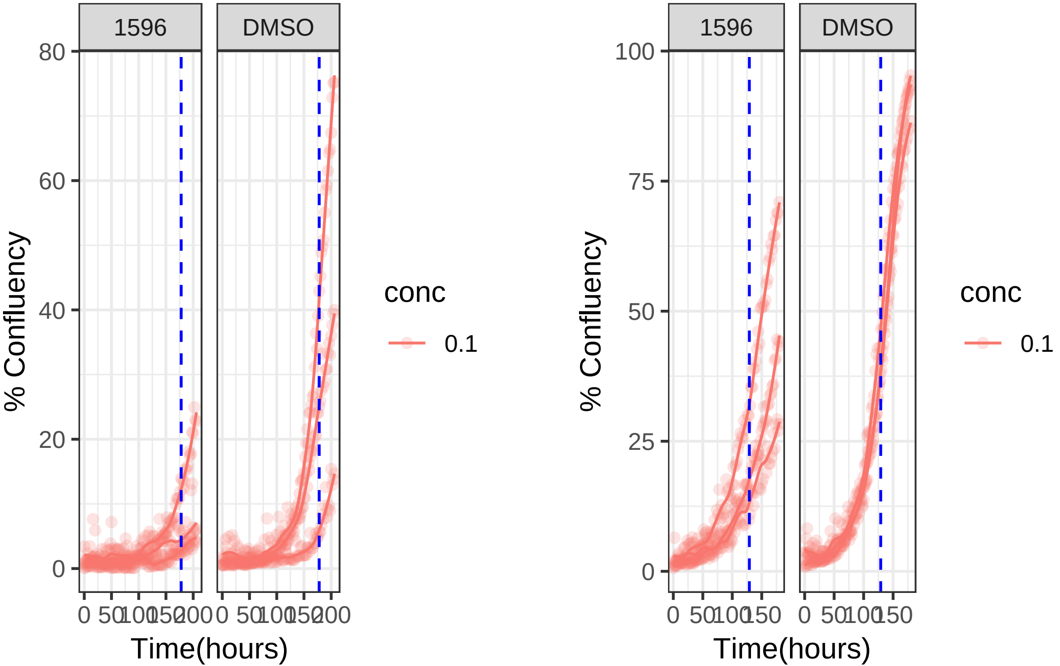

0.1 uM 1629 RPE1 hTert

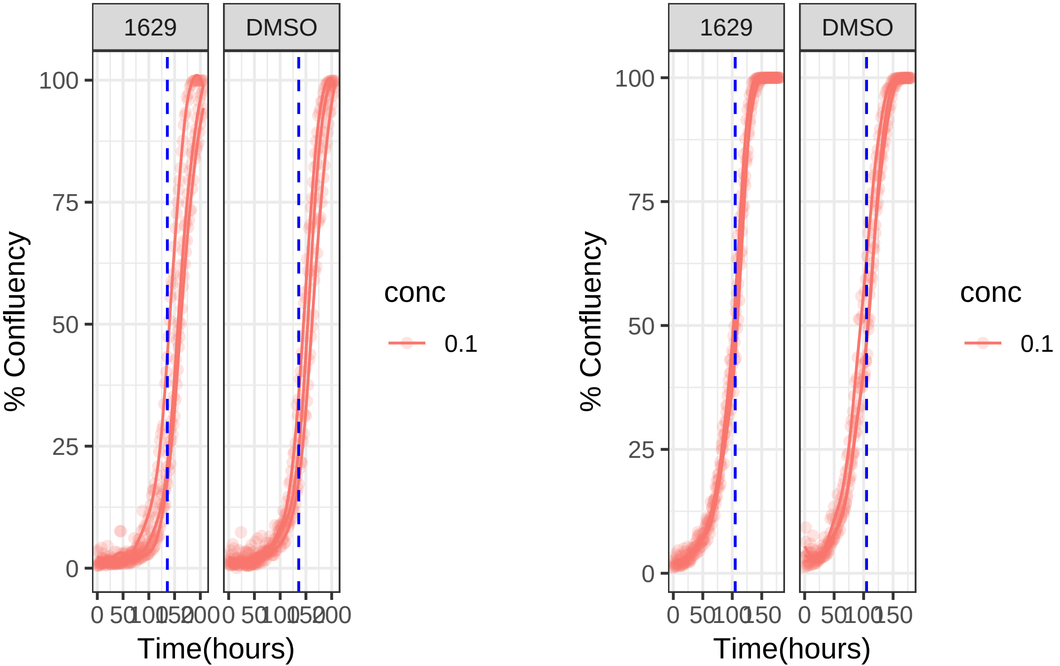

0.1 uM 1629 RPE1 Ts12 Ts5

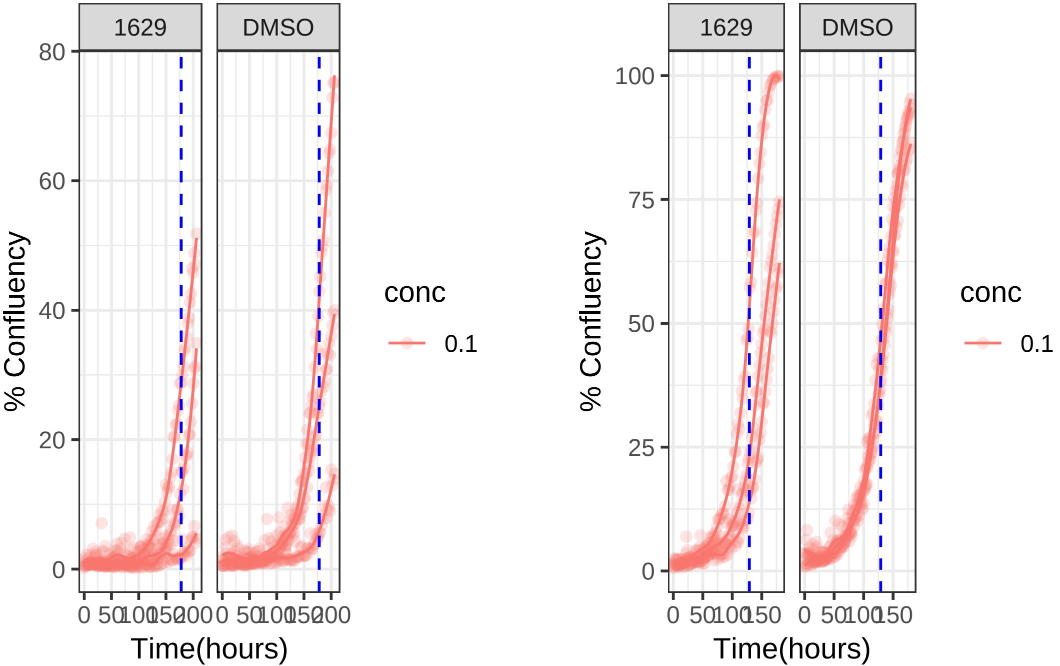

1 uM 1645 RPE1 hTert

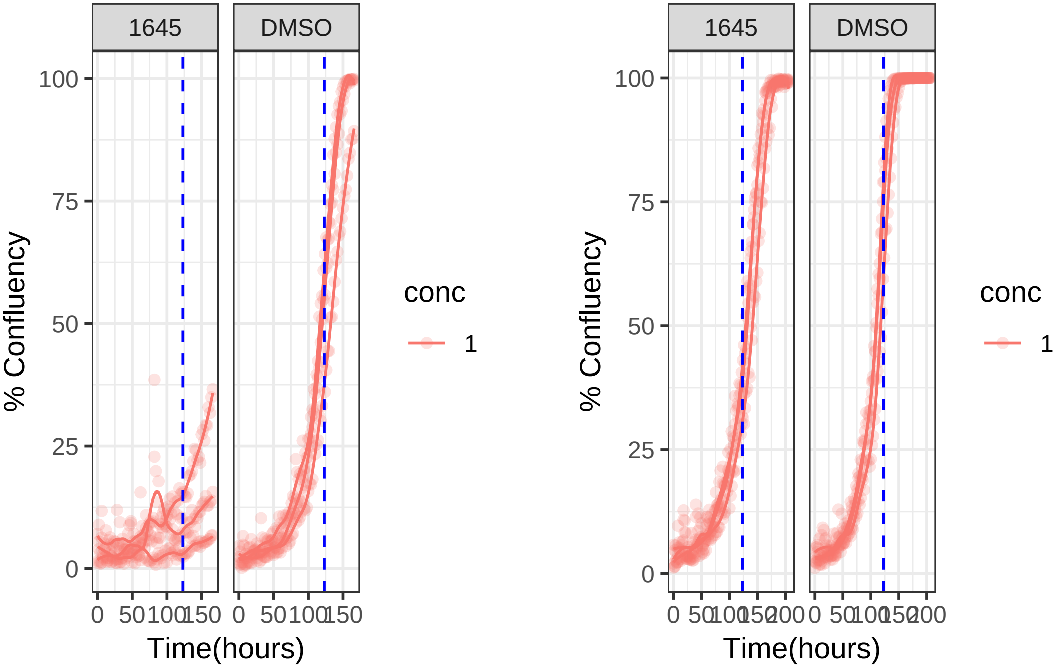

0.1 uM 1645 RPE1 hTert

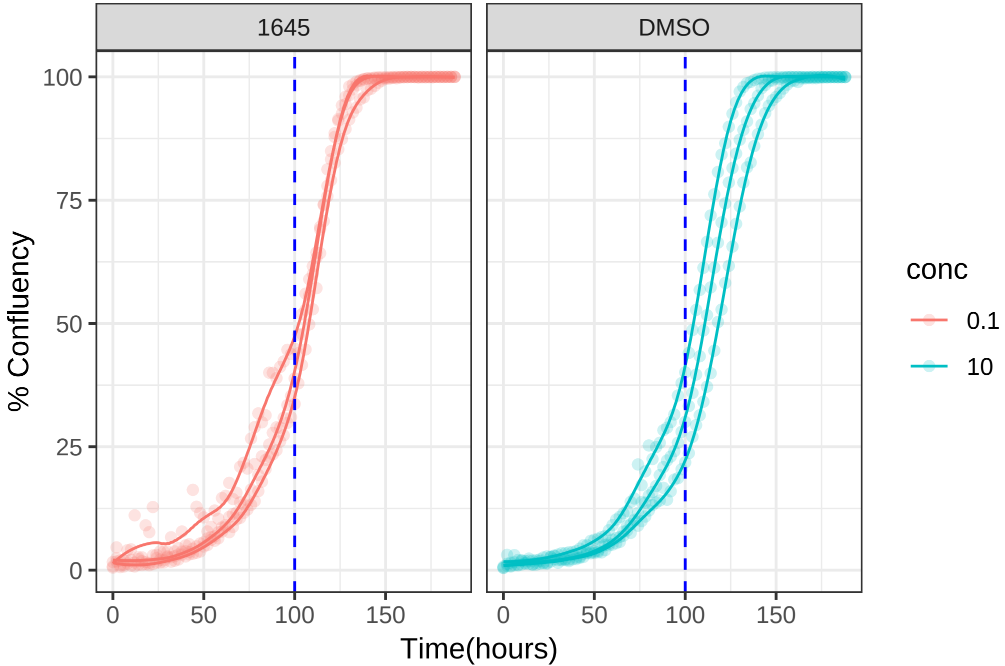

1 uM 1645 RPE1 Ts12 Ts5

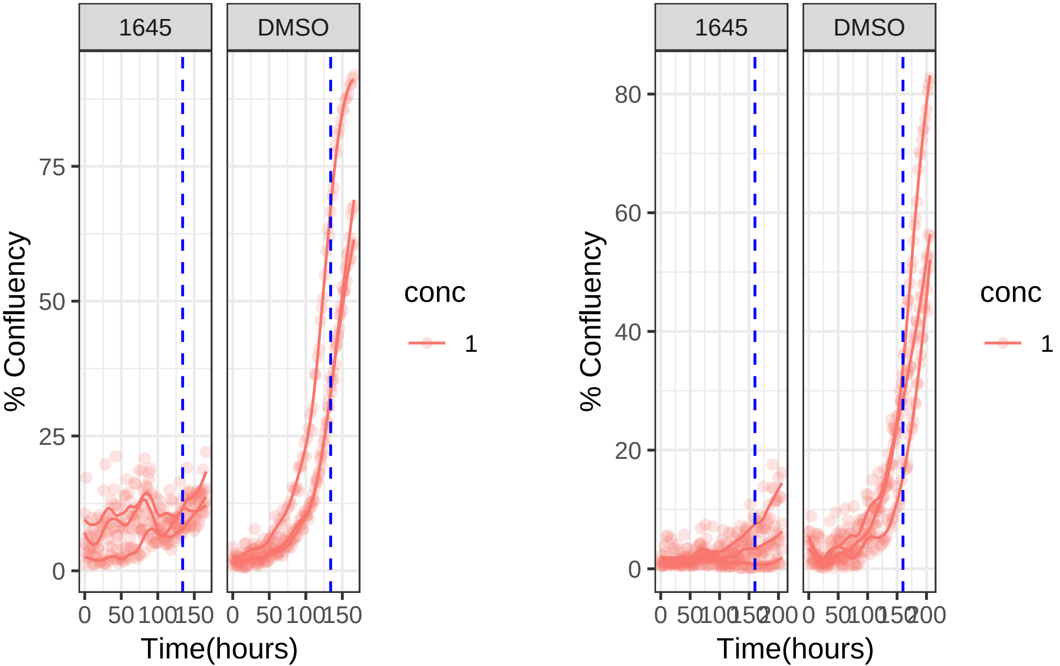

0.1 uM 1645 RPE1 Ts12 Ts5

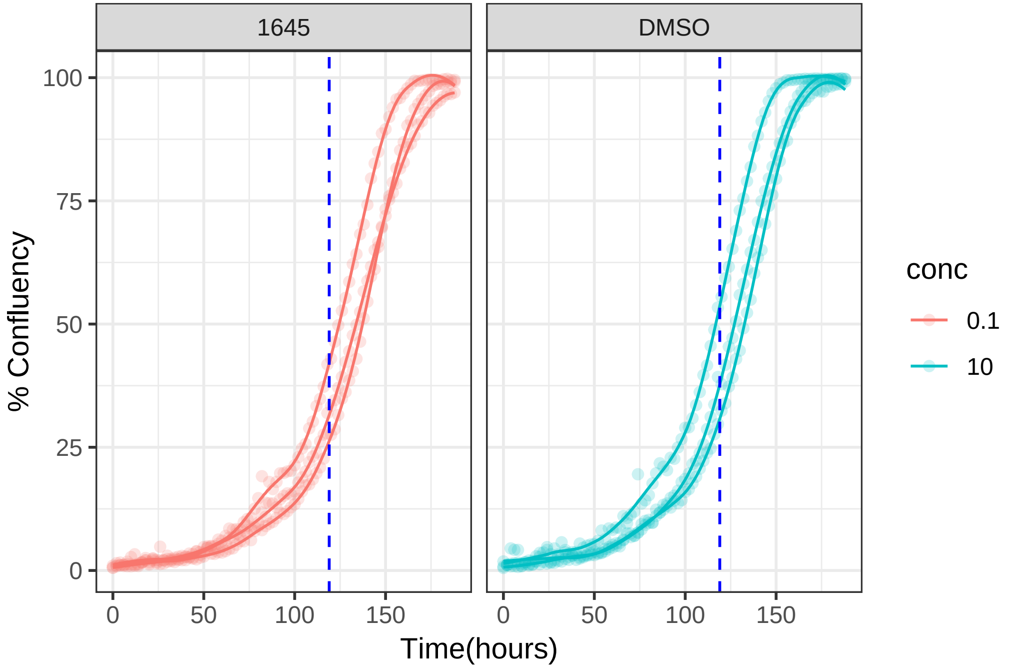

0.1 uM 1687 RPE1 hTert

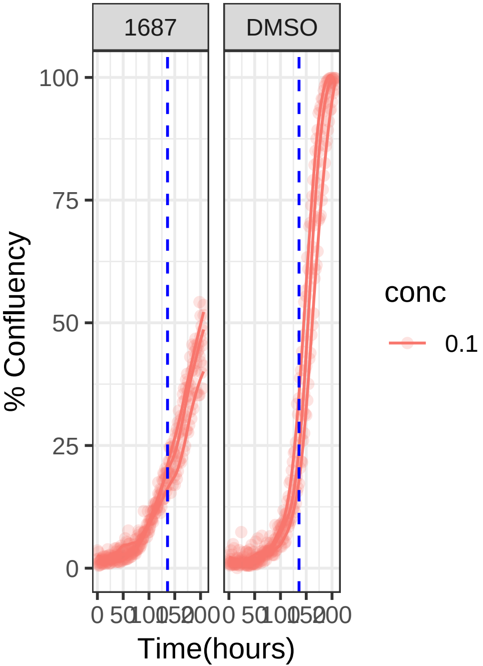

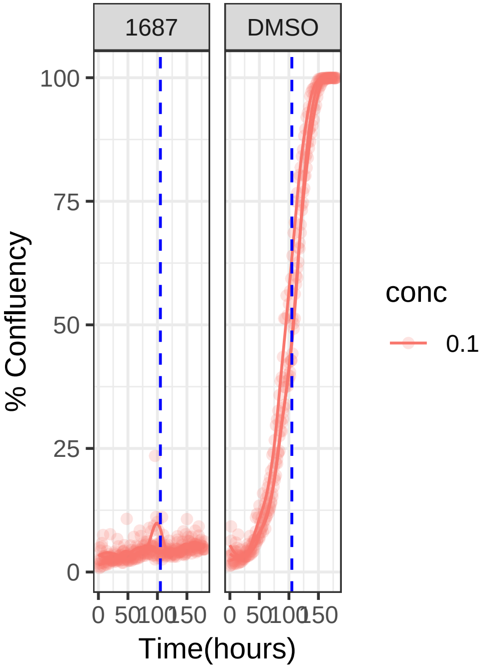

0.01 uM 1687 RPE1 hTert

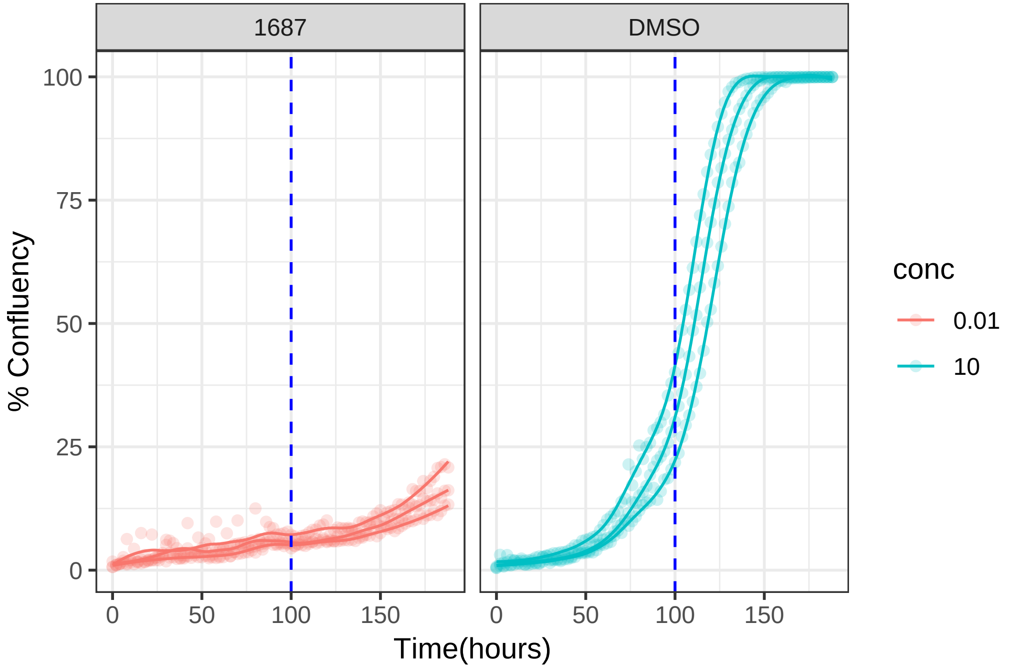

0.1 uM 1687 RPE1 Ts12 Ts5

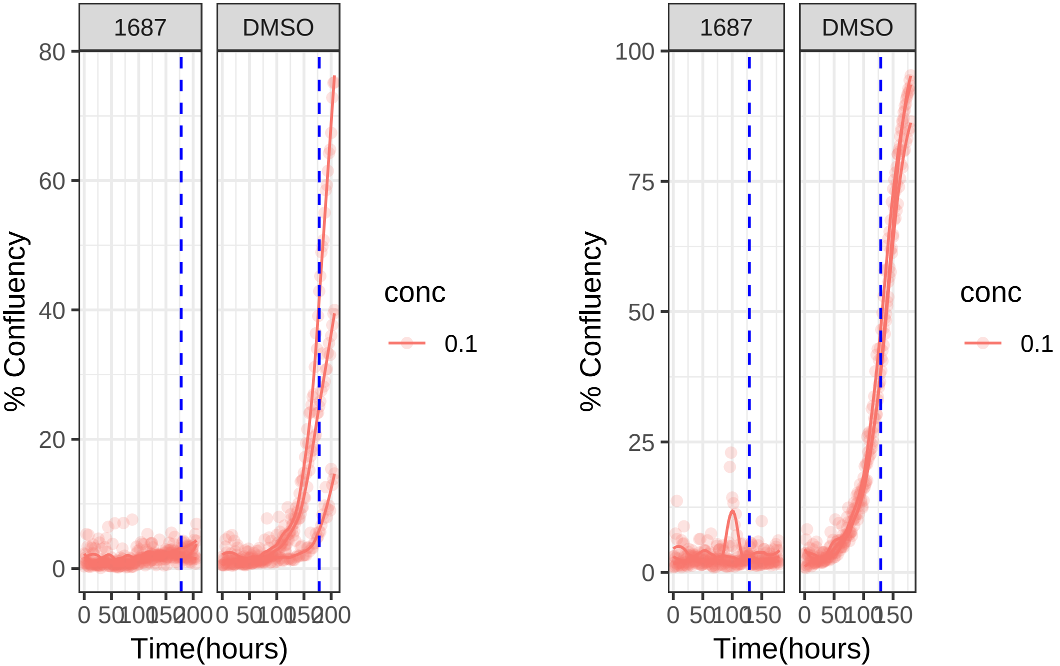

0.01 uM 1687 RPE1 Ts12 Ts5

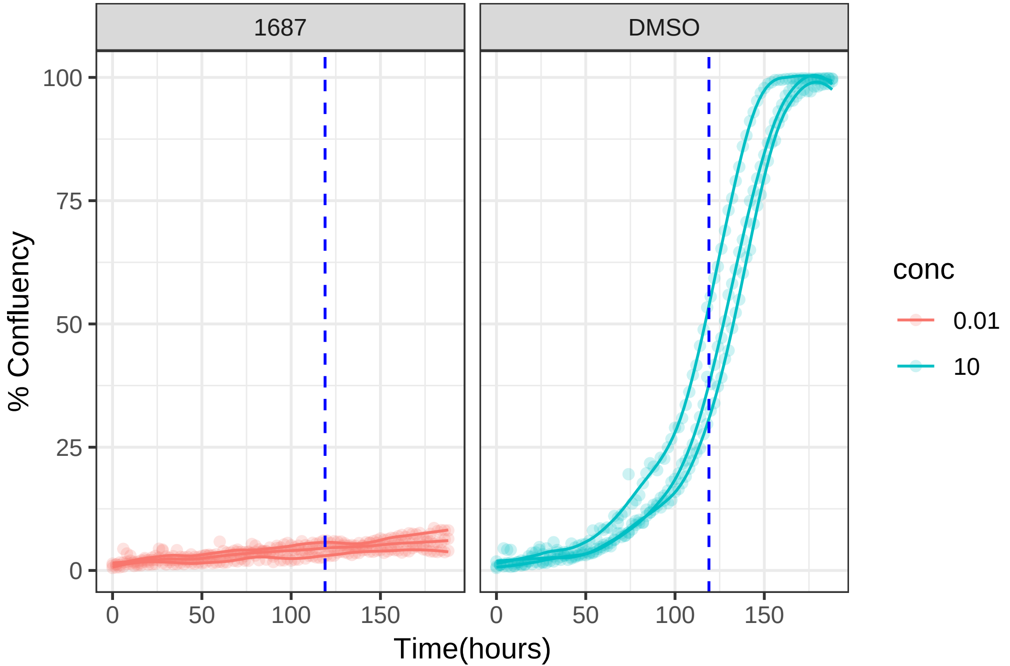

10 uM 1691 RPE1 hTert

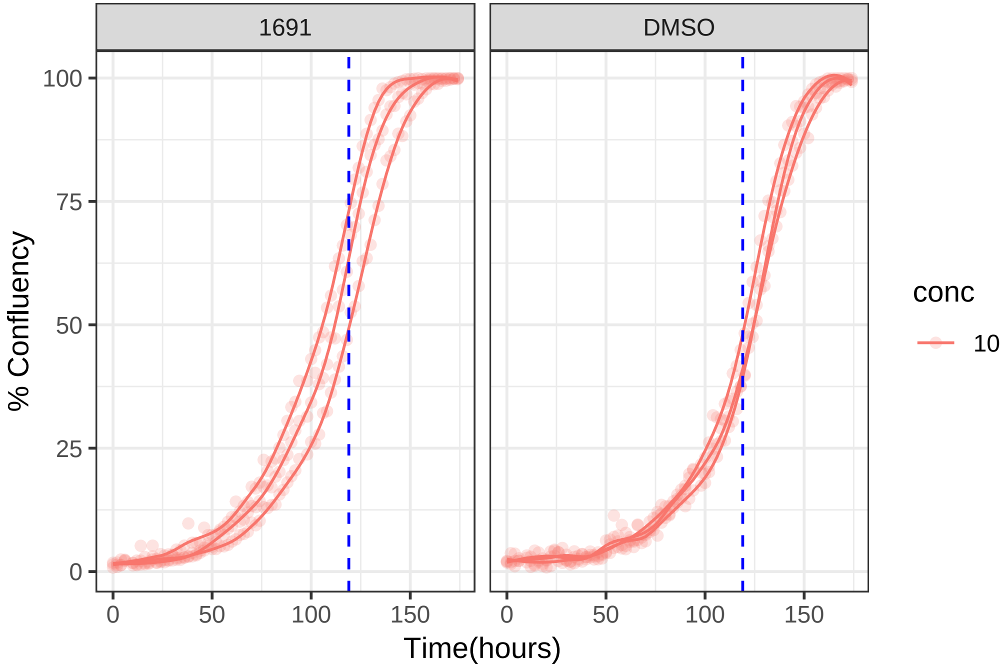

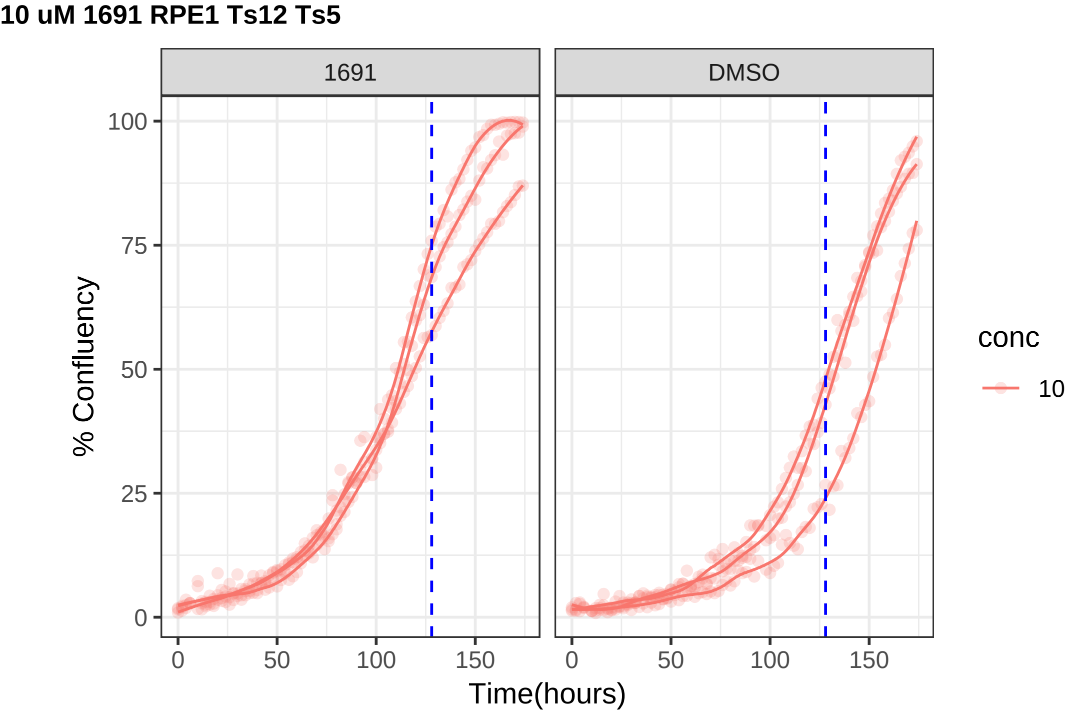

10 uM 1691 RPE1 Ts12 Ts5
1691
DMSO
% Confluency
0
25
50
75
100
0
50
100
150
Time(hours)
conc
10

1 uM 1729 RPE1 hTert

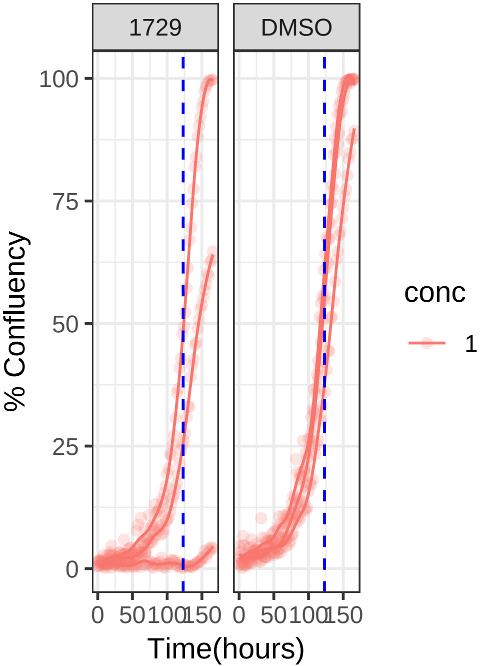

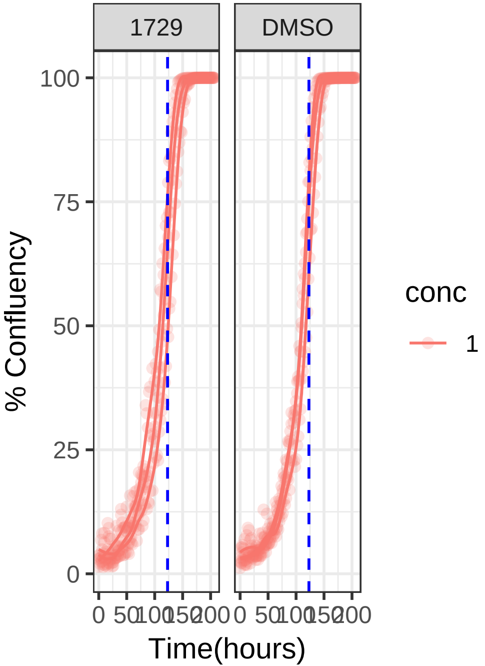

1 uM 1729 RPE1 Ts12 Ts5

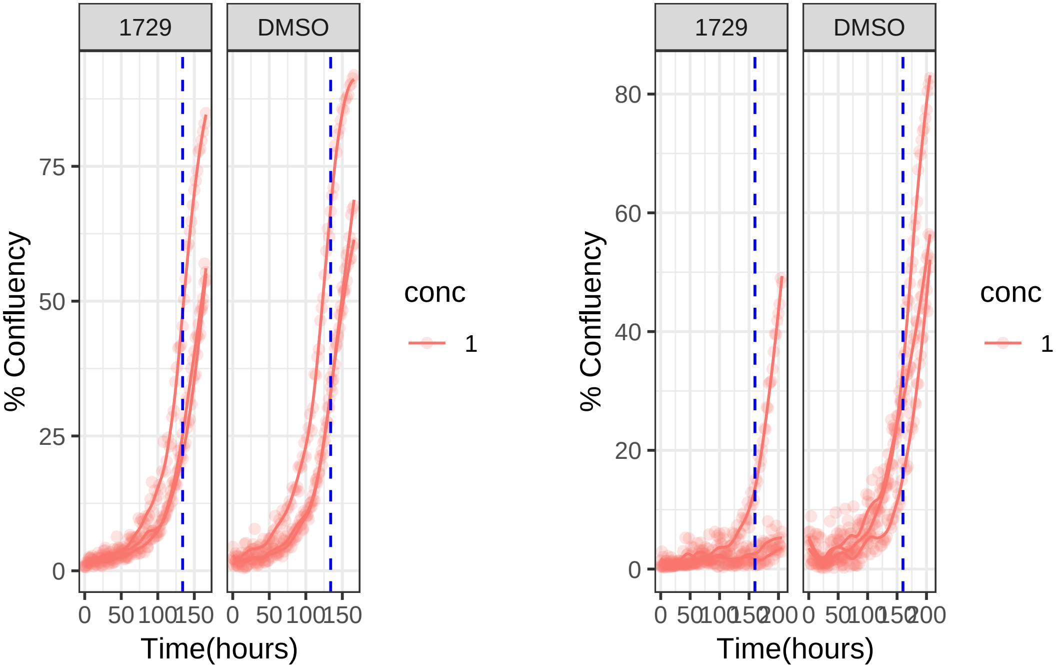

1 uM 1743 RPE1 hTert

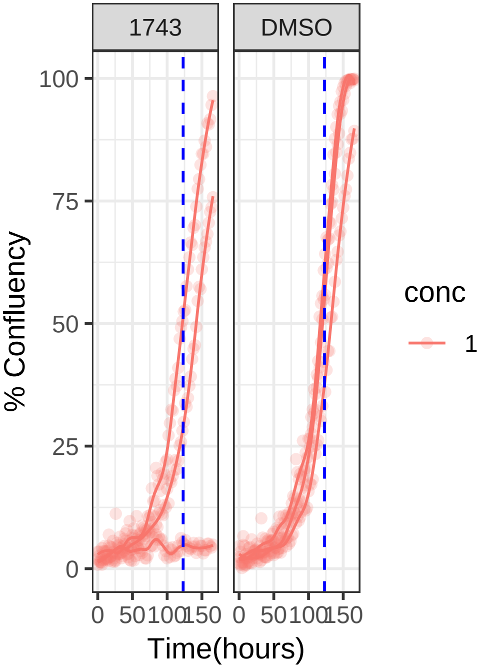

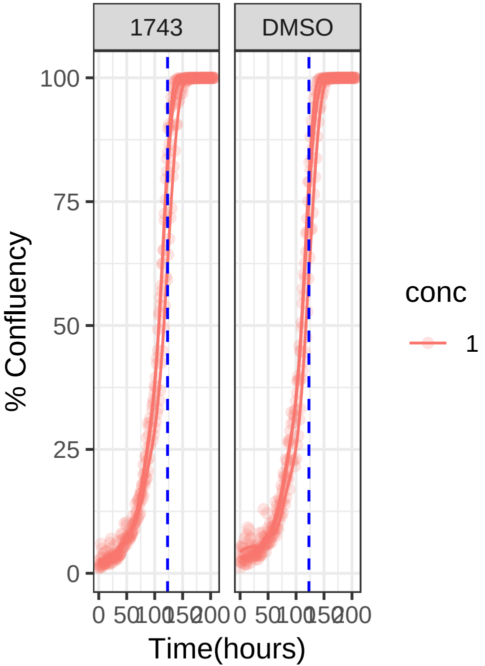

1 uM 1743 RPE1 Ts12 Ts5

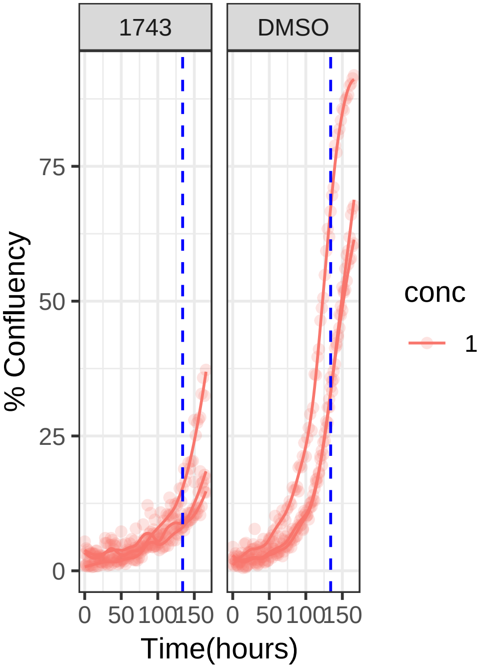

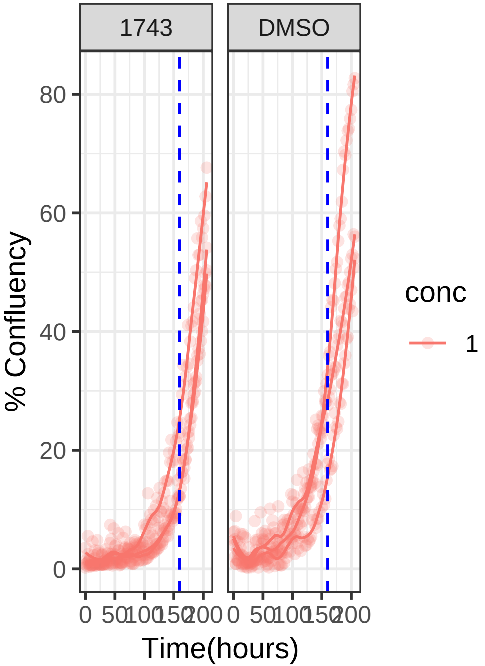

10 uM 1765 RPE1 hTert

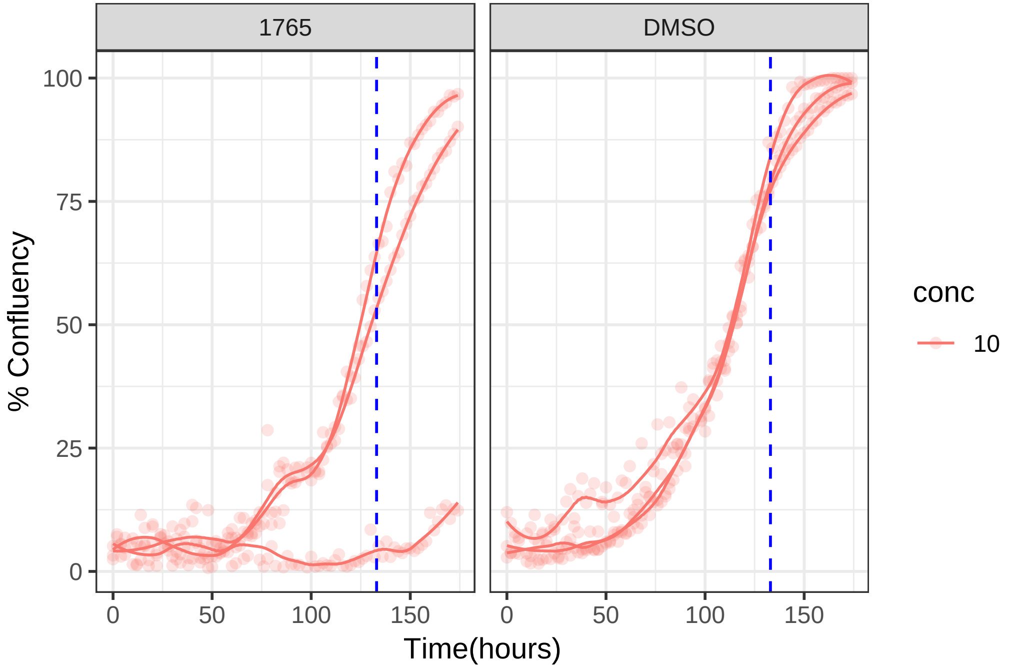

10 uM 1765 RPE1 Ts12 Ts5

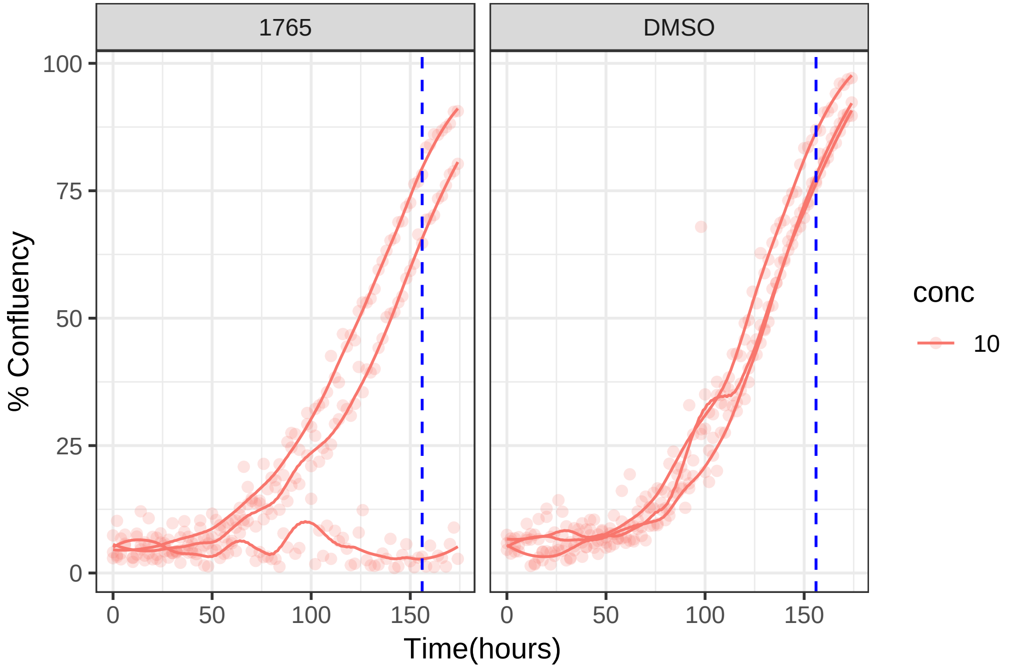

10 uM 1789 RPE1 hTert

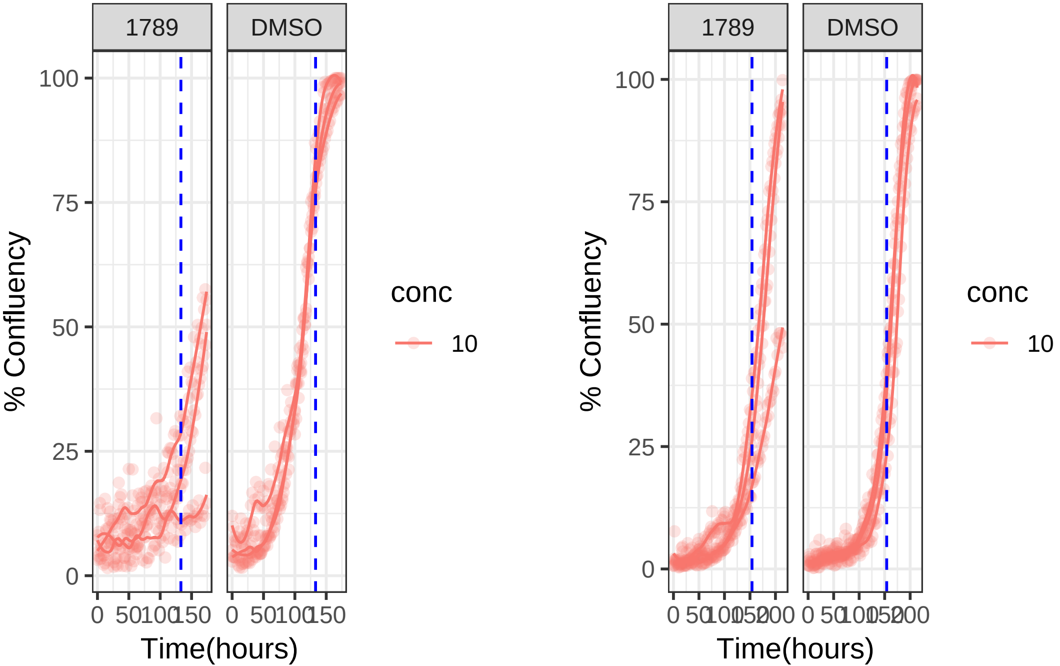

10 uM 1789 RPE1 Ts12 Ts5

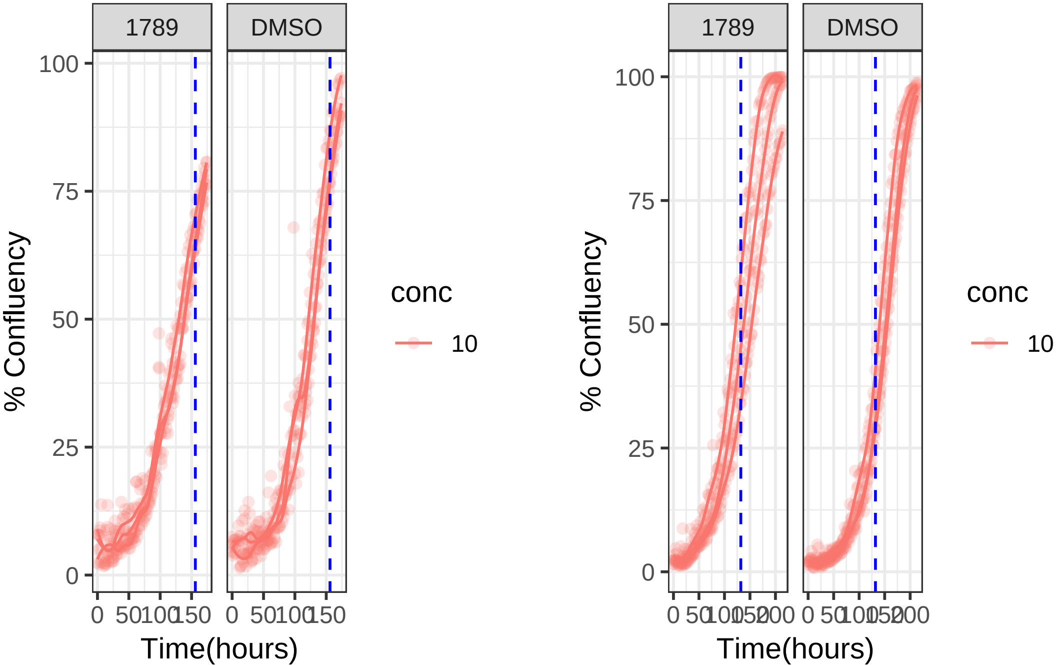

1 uM 1797 RPE1 hTert

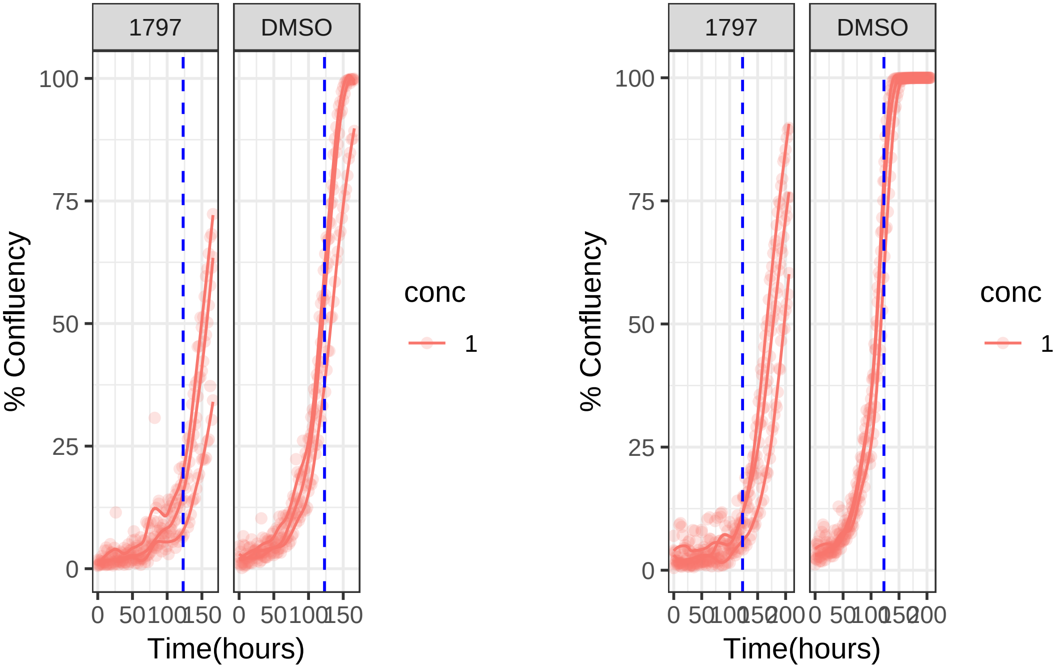

1 uM 1797 RPE1 Ts12 Ts5

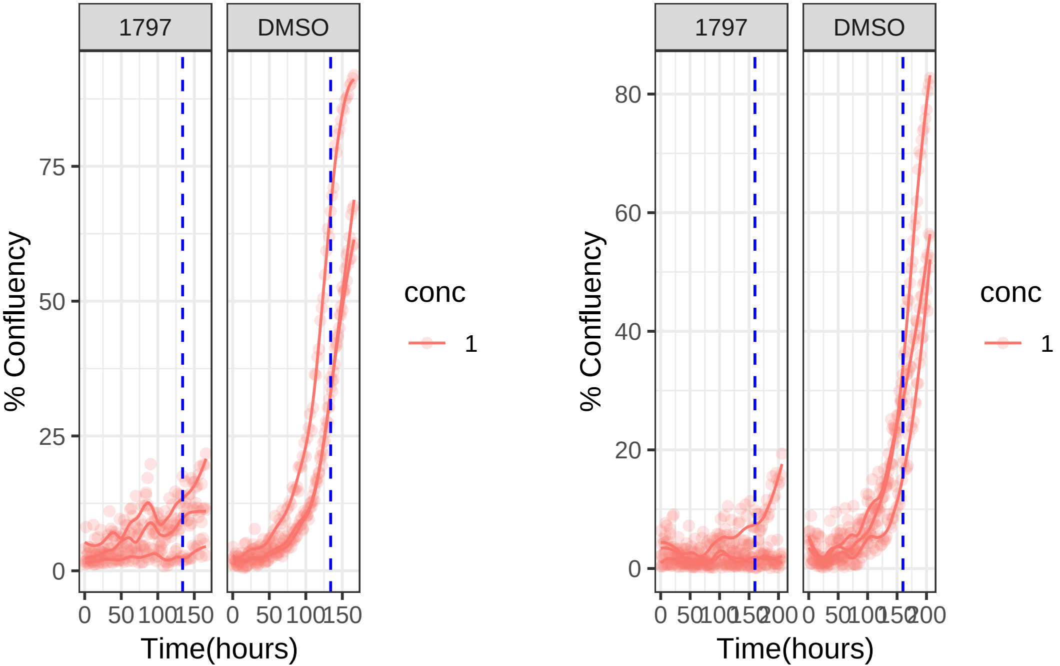

1 uM 1801 RPE1 hTert

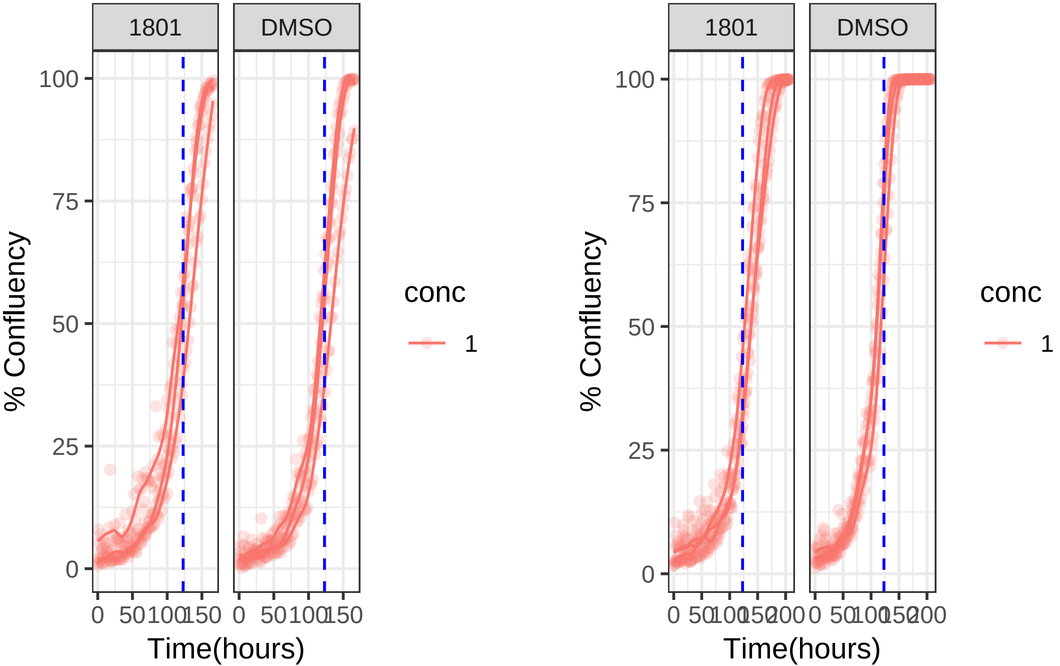

1 uM 1801 RPE1 Ts12 Ts5

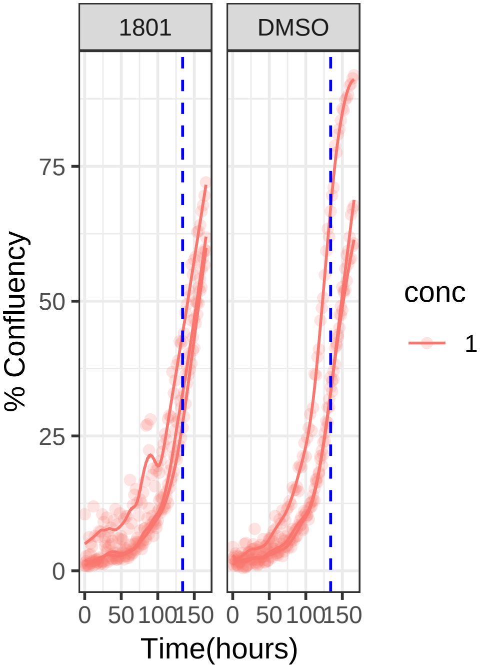

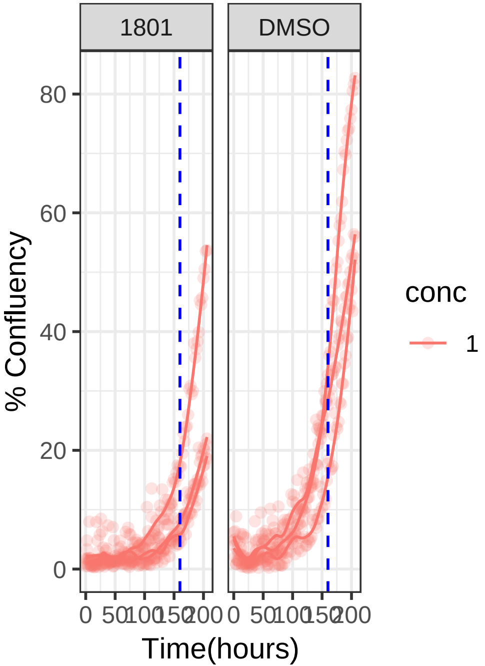

1 uM 1803 RPE1 hTert

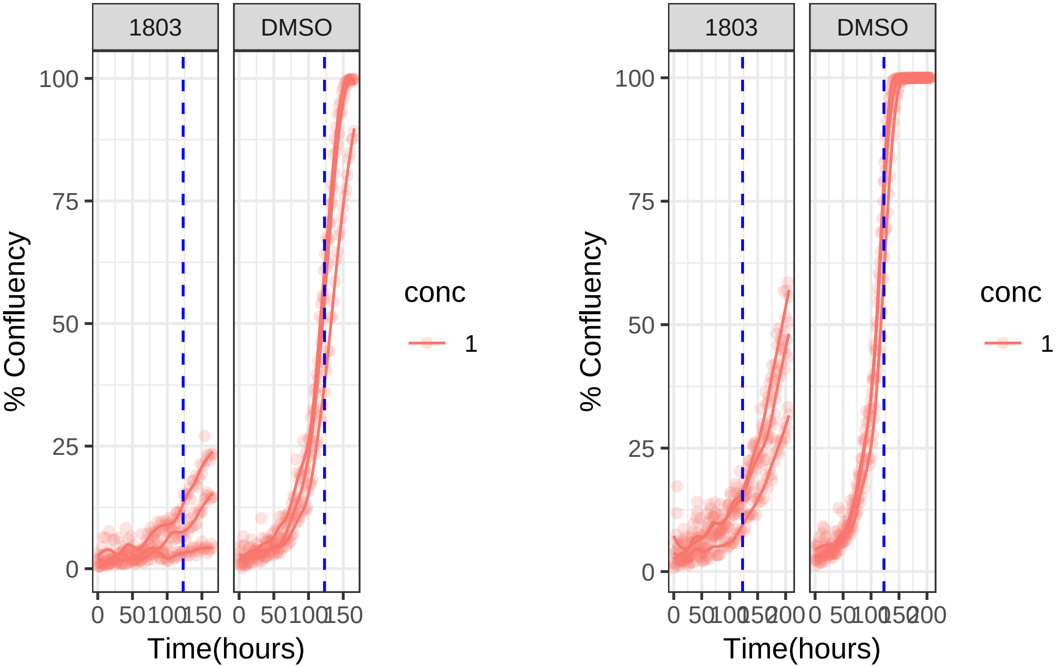

1 uM 1803 RPE1 Ts12 Ts5

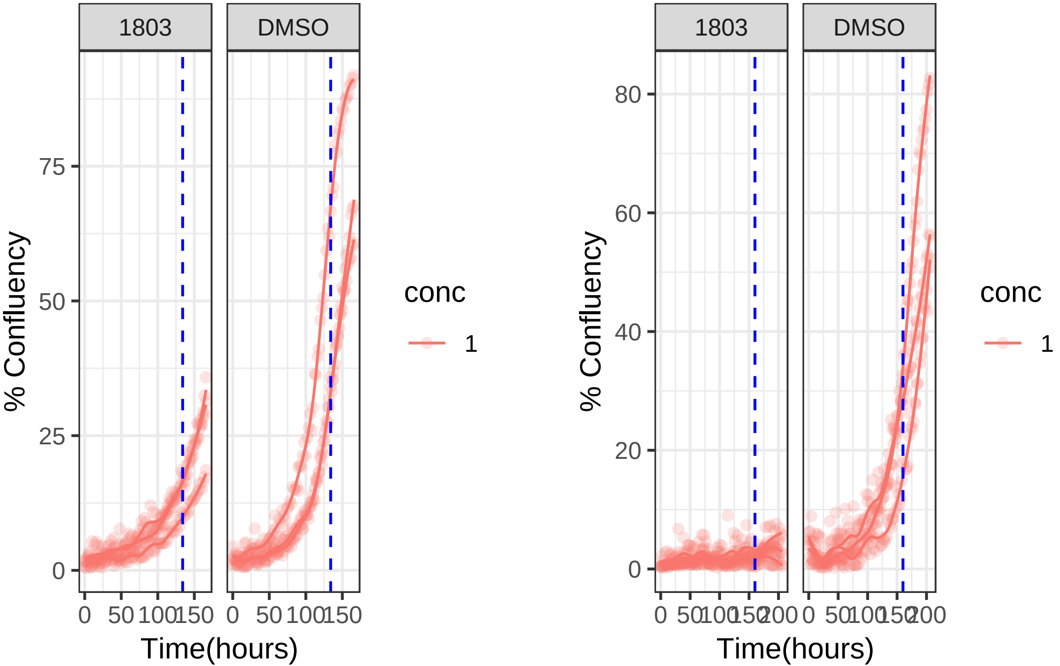

0.01 uM 1807 RPE1 hTert

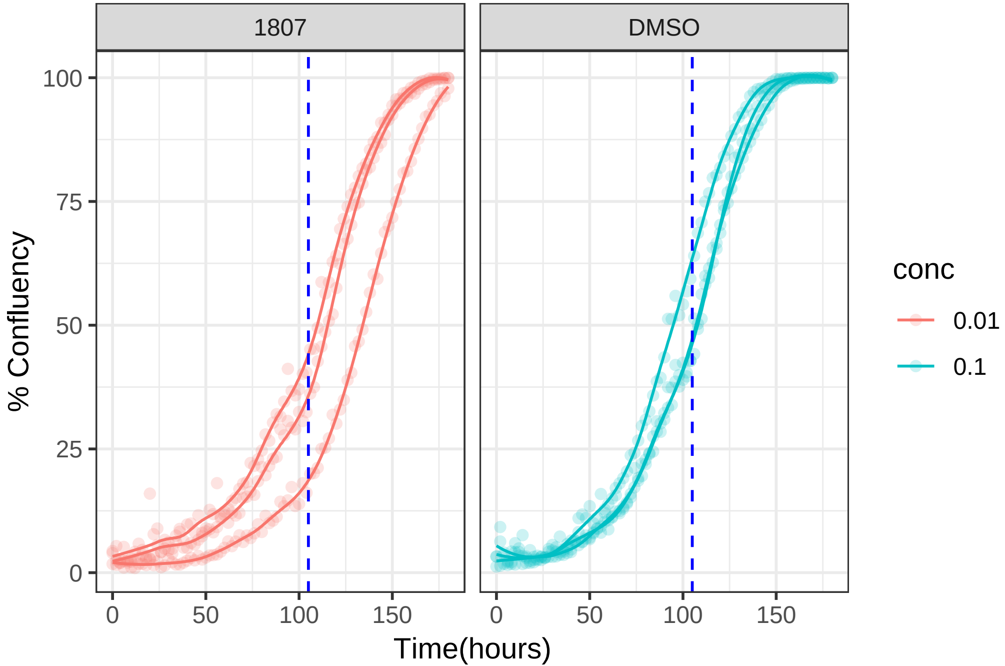

0.01 uM 1807 RPE1 Ts12 Ts5

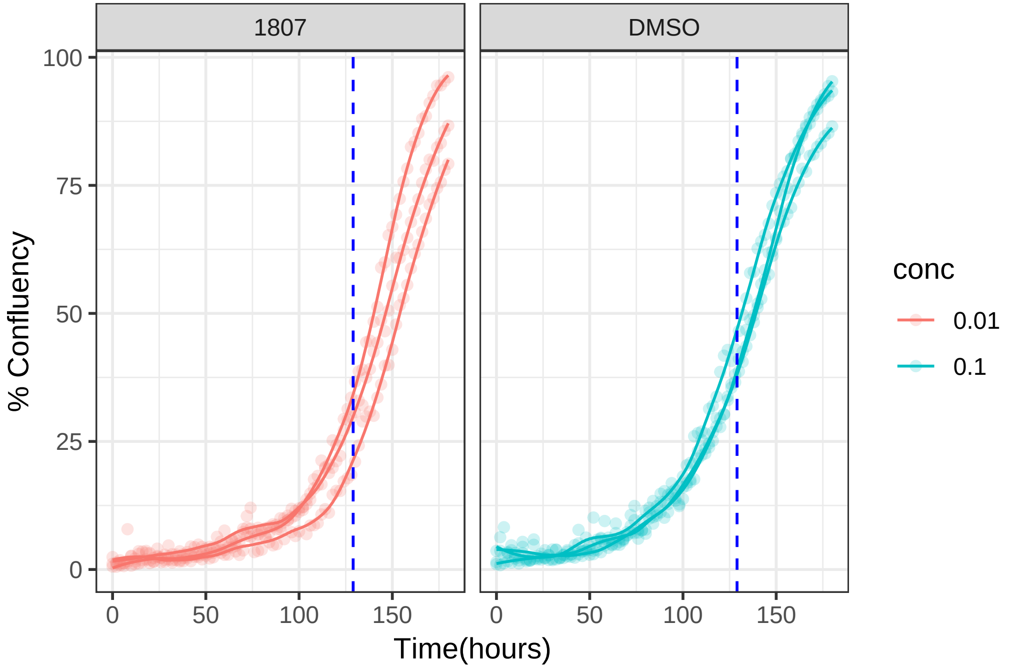

10 uM 1814 RPE1 hTert

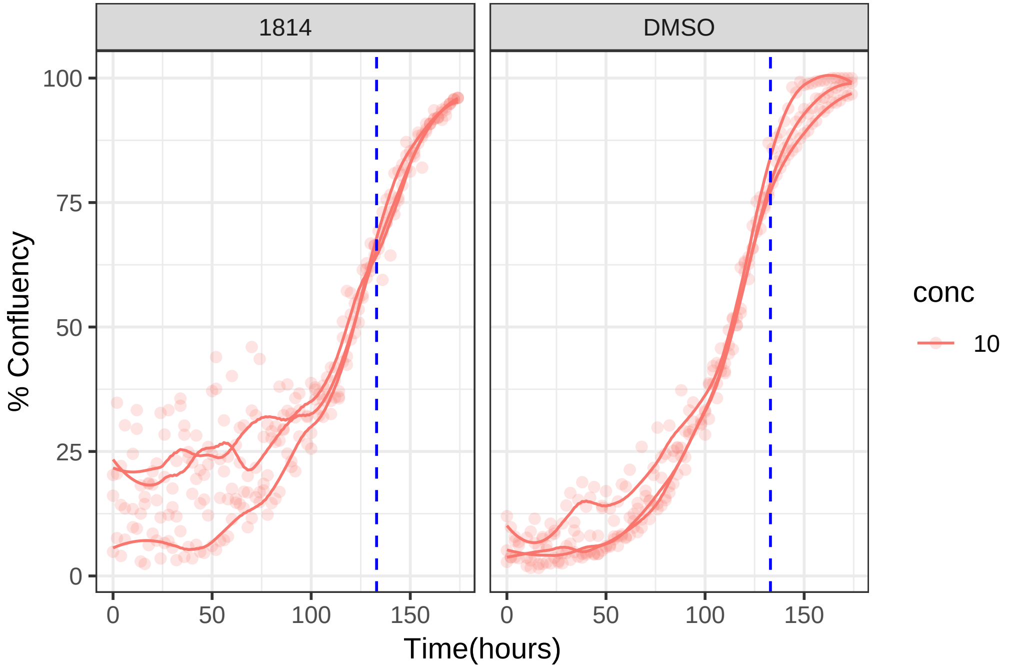

10 uM 1814 RPE1 Ts12 Ts5

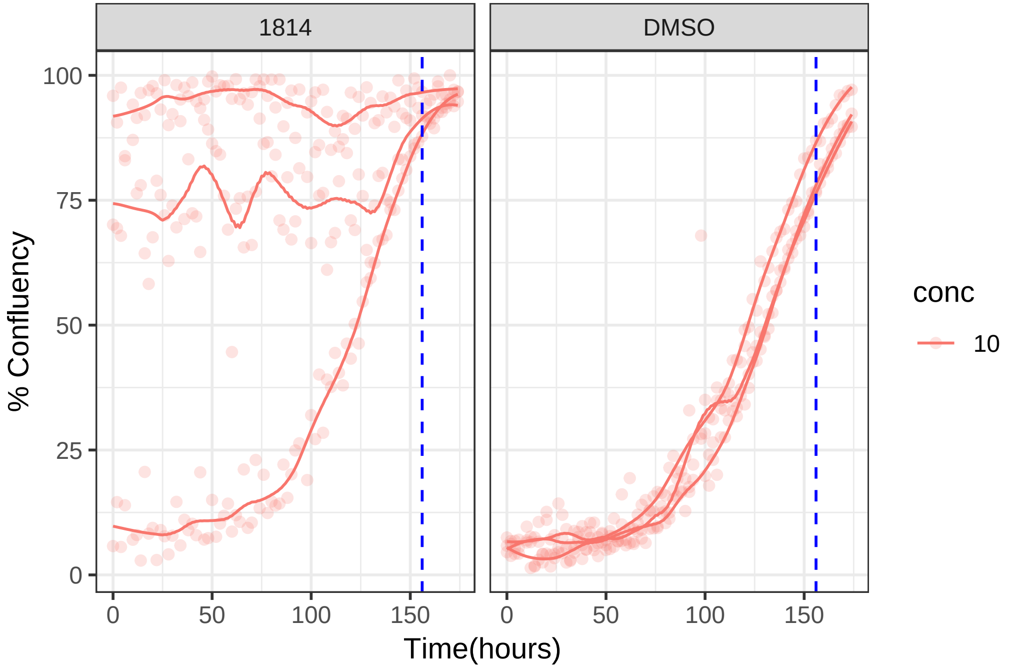

10 uM 1831 RPE1 hTert

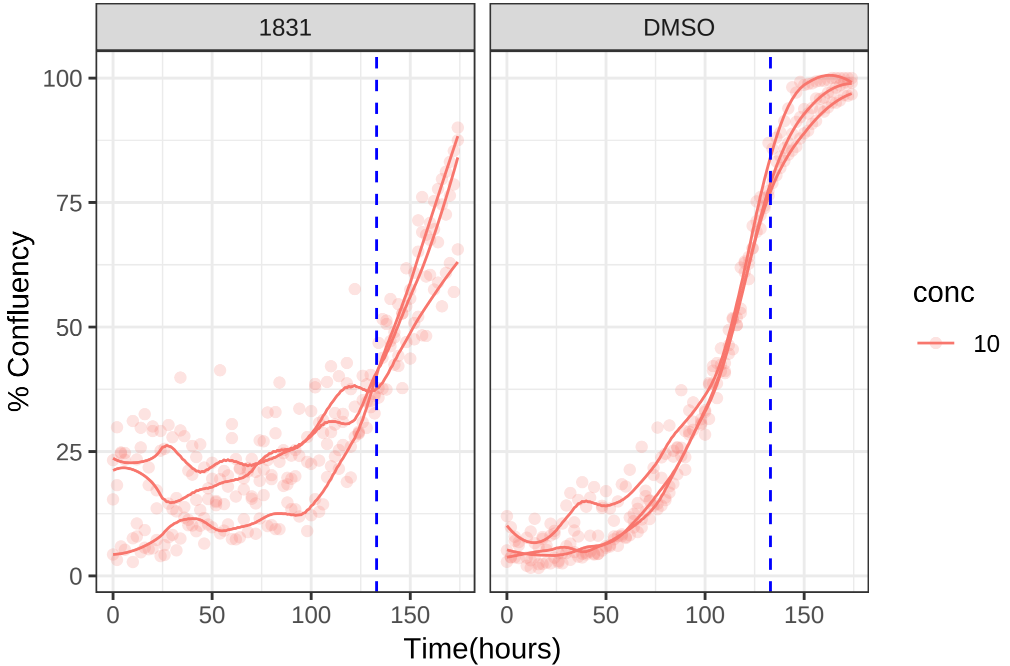

10 uM 1831 RPE1 Ts12 Ts5

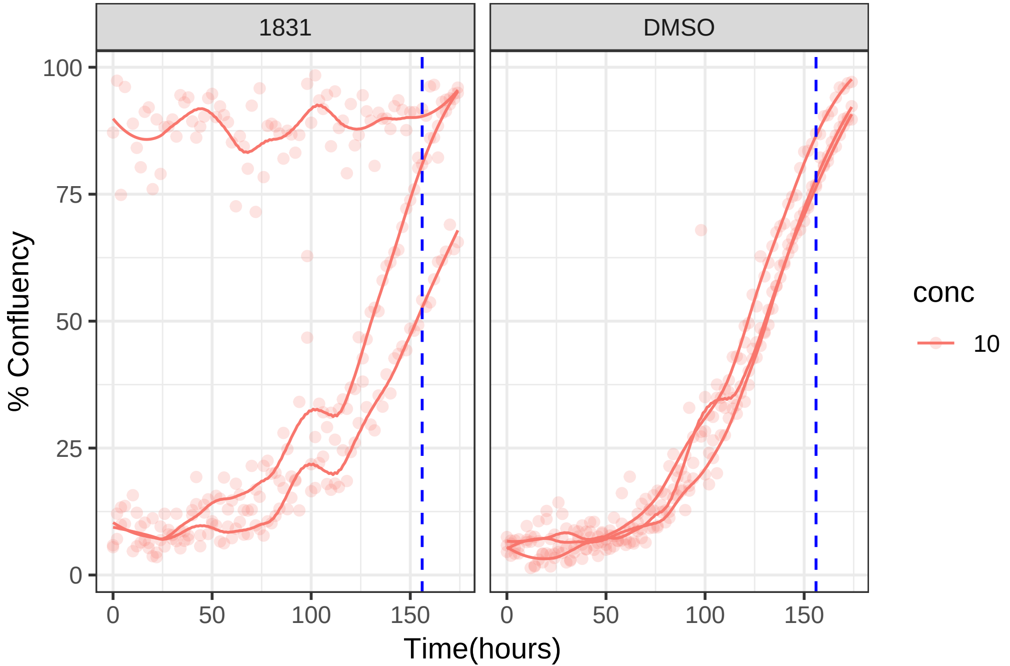

10 uM 1853 RPE1 hTert

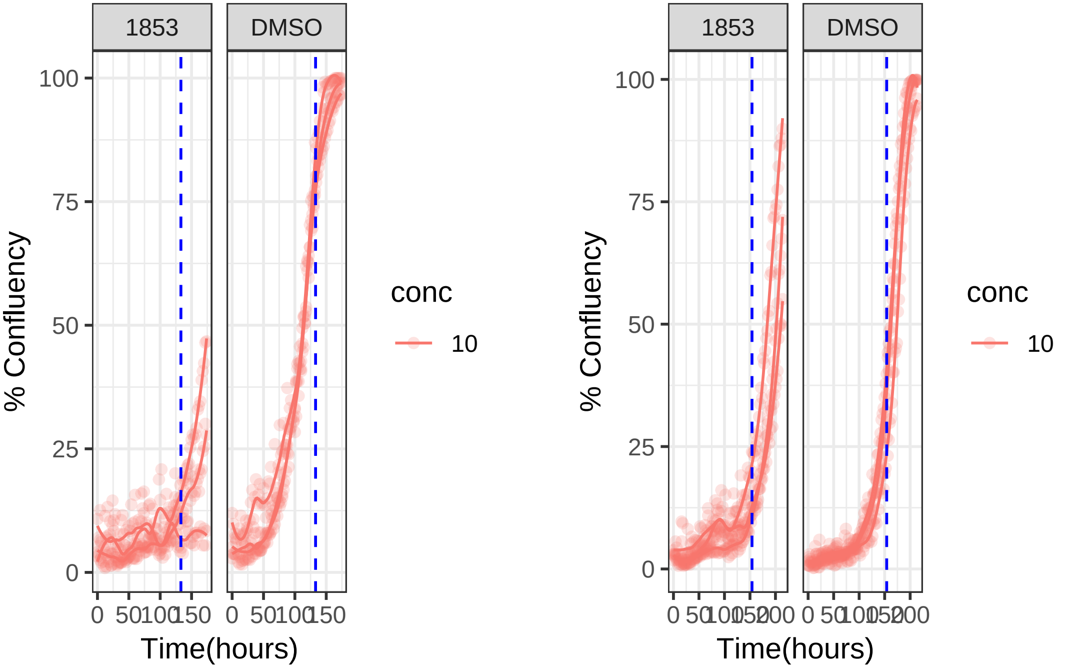

10 uM 1853 RPE1 Ts12 Ts5

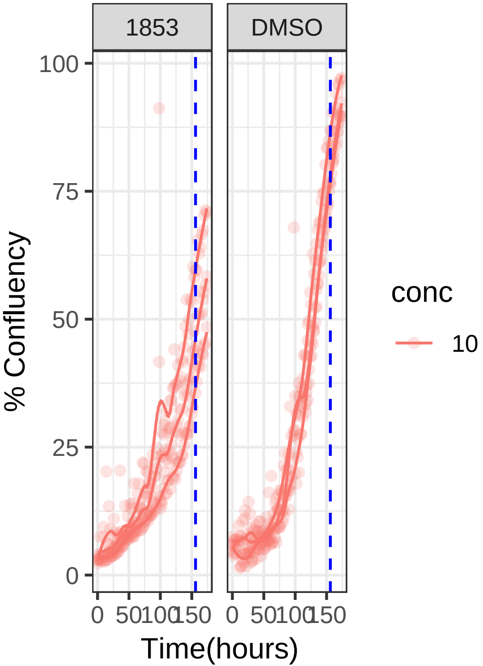

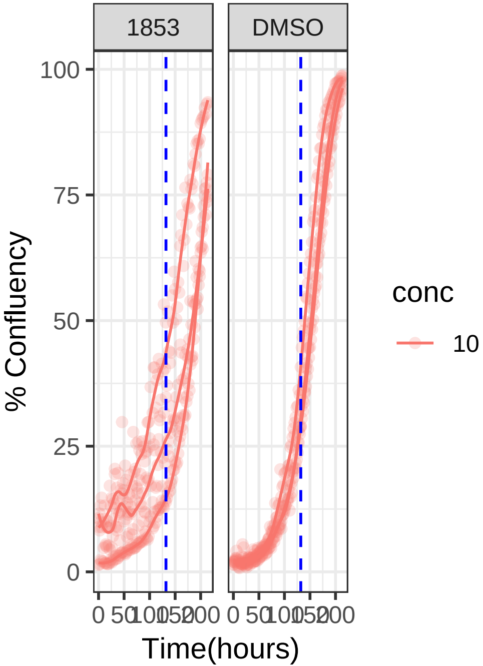

10 uM 1895 RPE1 hTert

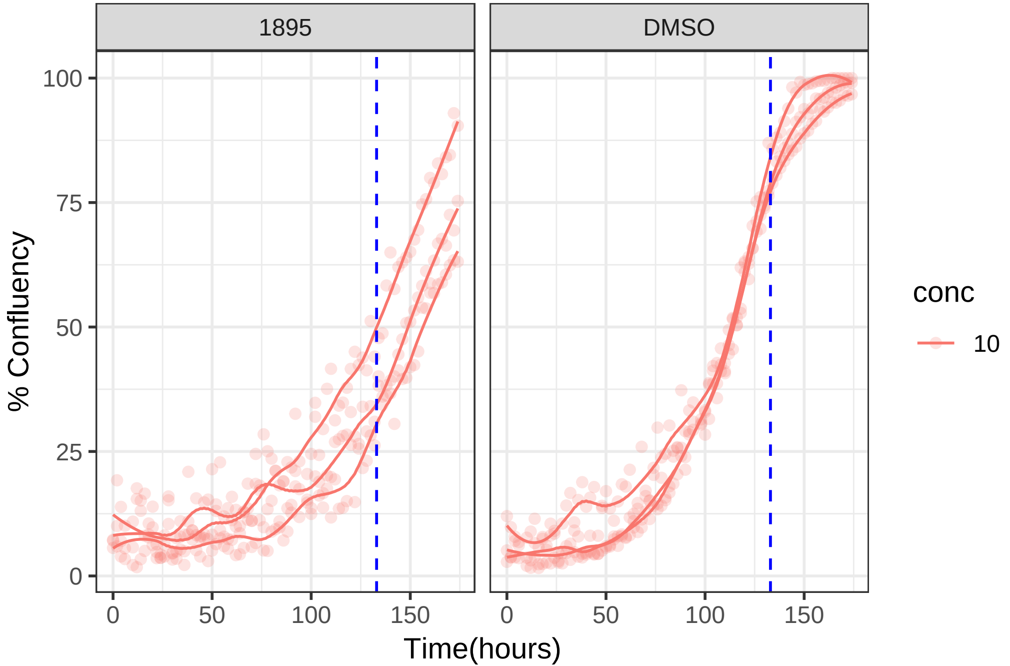

10 uM 1895 RPE1 Ts12 Ts5

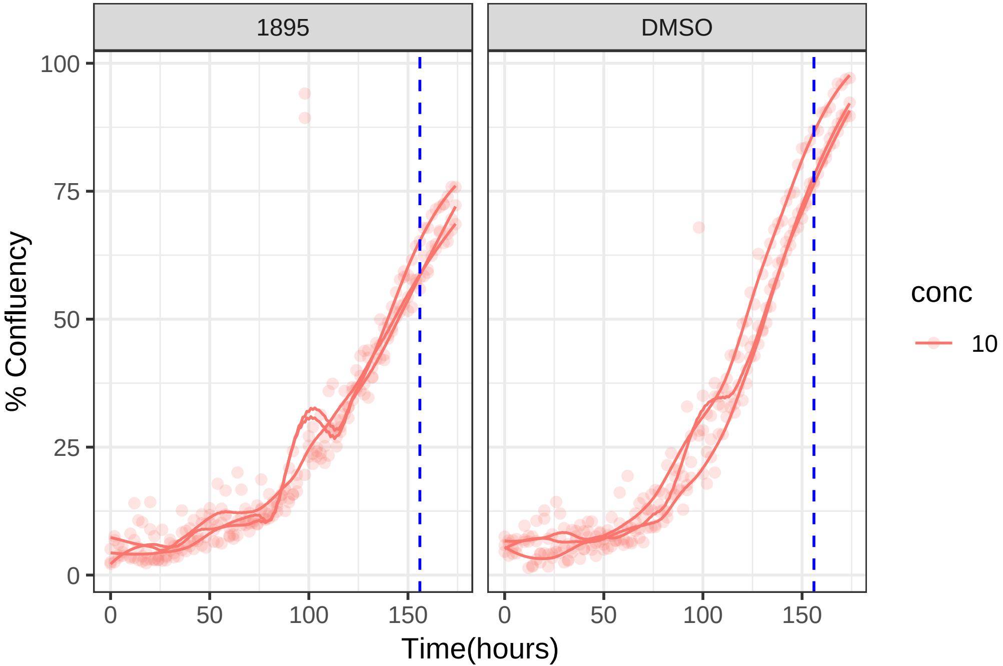

10 uM 1928 RPE1 hTert

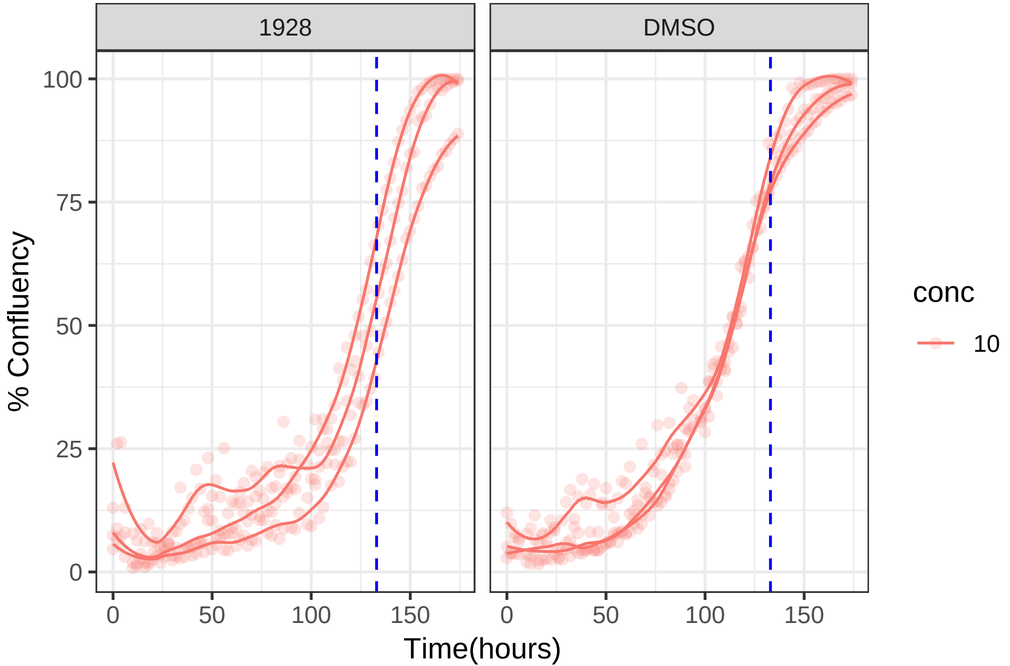

10 uM 1928 RPE1 Ts12 Ts5

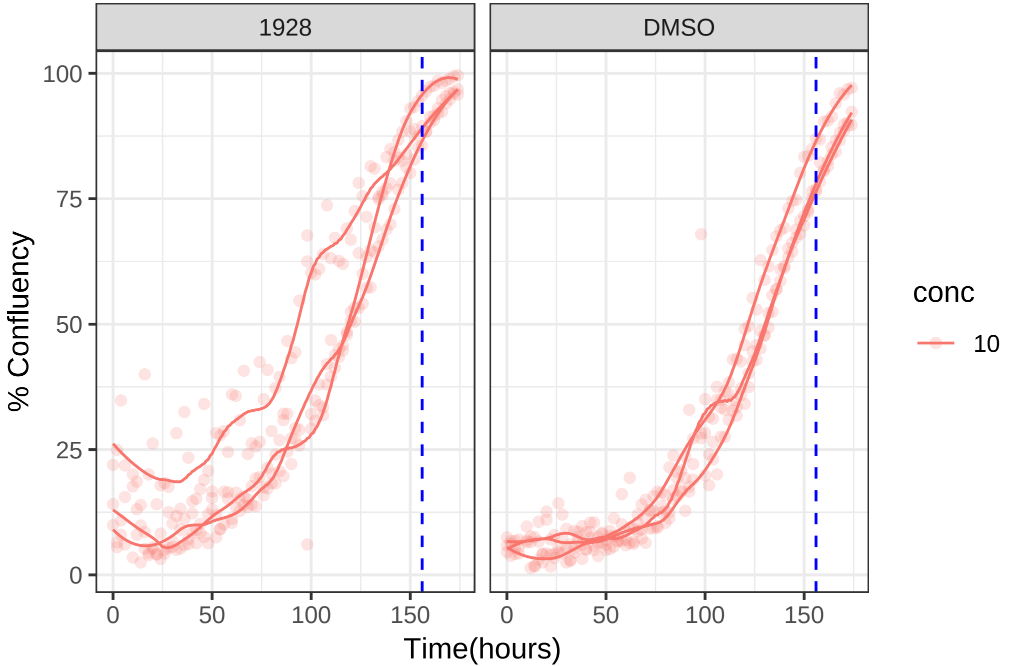

0.01 uM 1999 RPE1 hTert

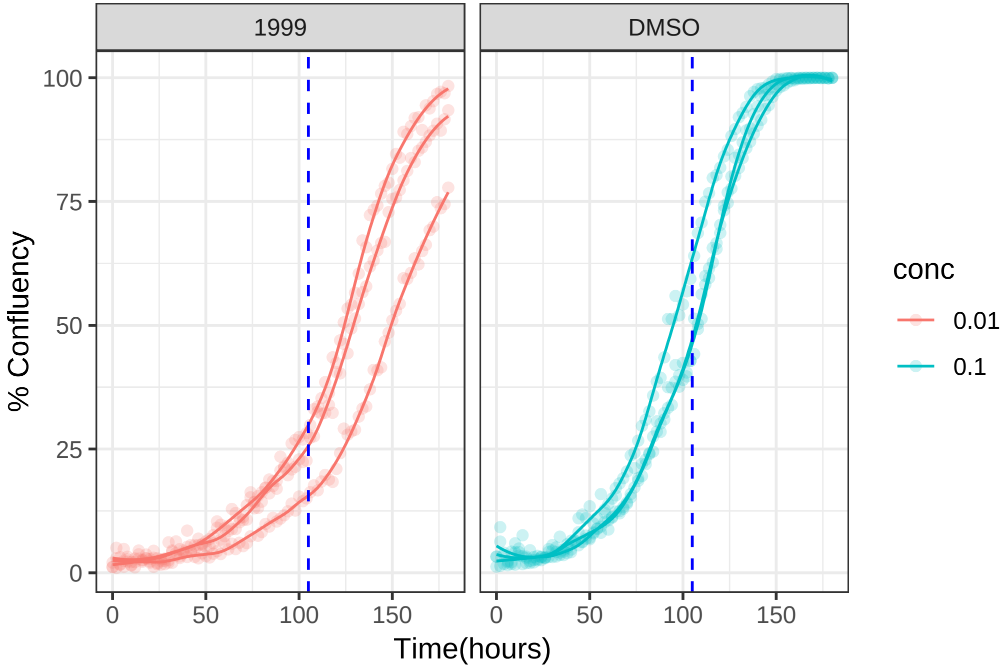

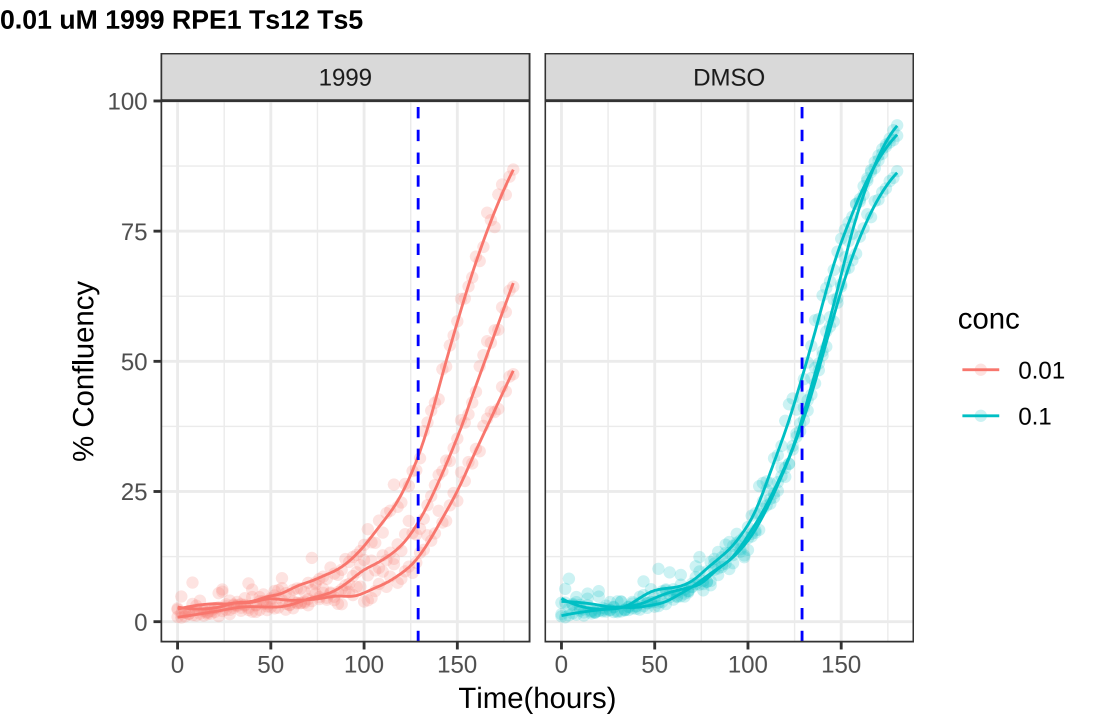

0.01 uM 1999 RPE1 Ts12 Ts5
1999
DMSO
100
75
50
25
0
% Confluency
0
50
100
150
Time(hours)
conc
0.01
0.1

1 uM 2008 RPE1 hTert

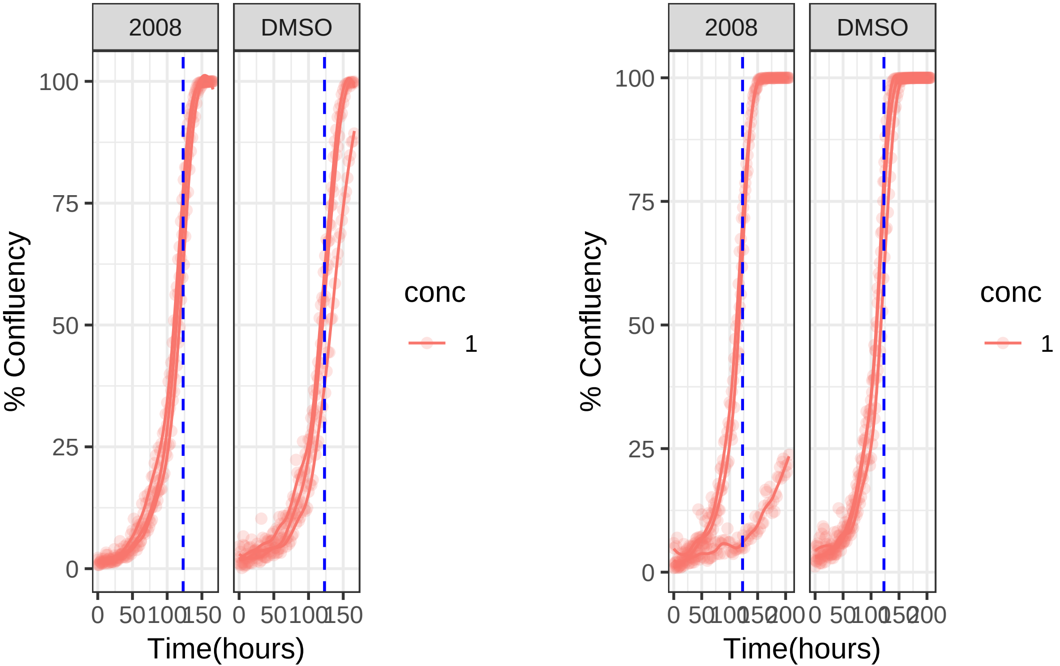

1 uM 2008 RPE1 Ts12 Ts5

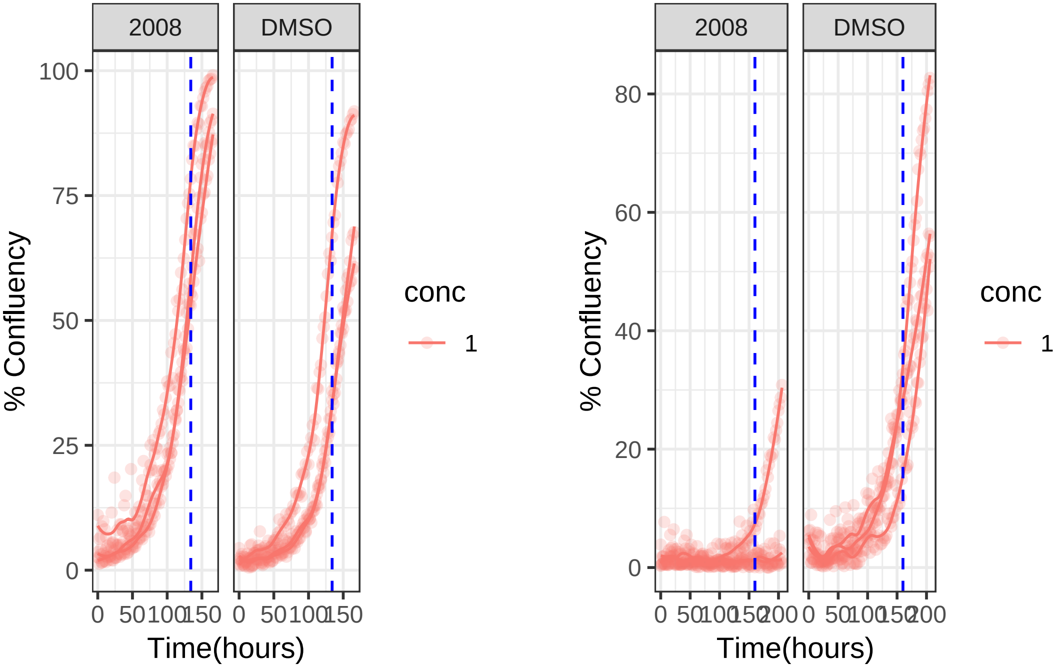

1 uM 2014 RPE1 hTert

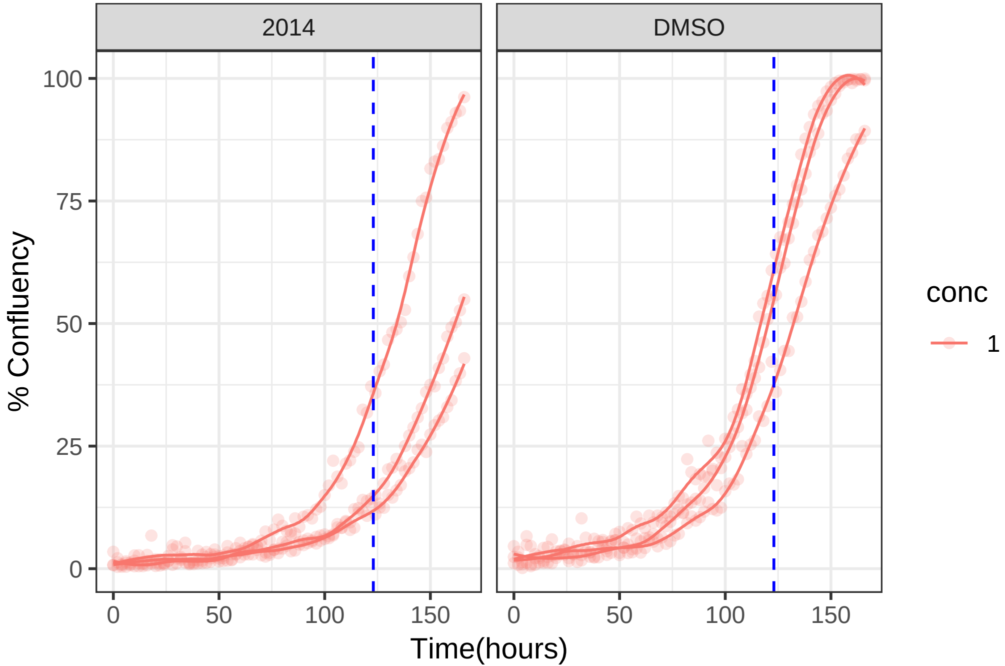

1 uM 2014 RPE1 Ts12 Ts5

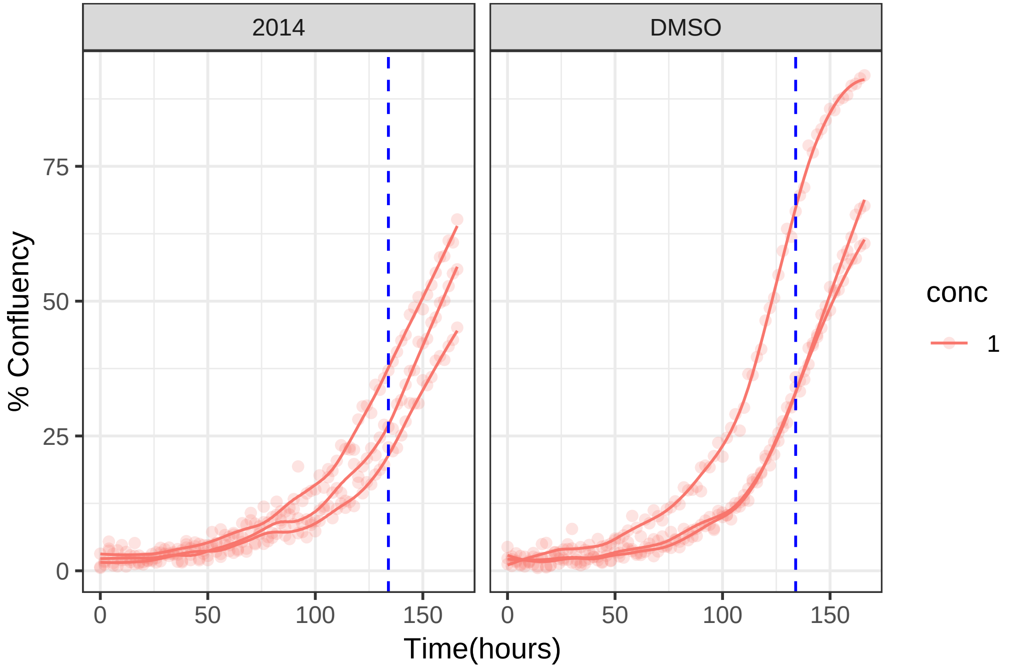

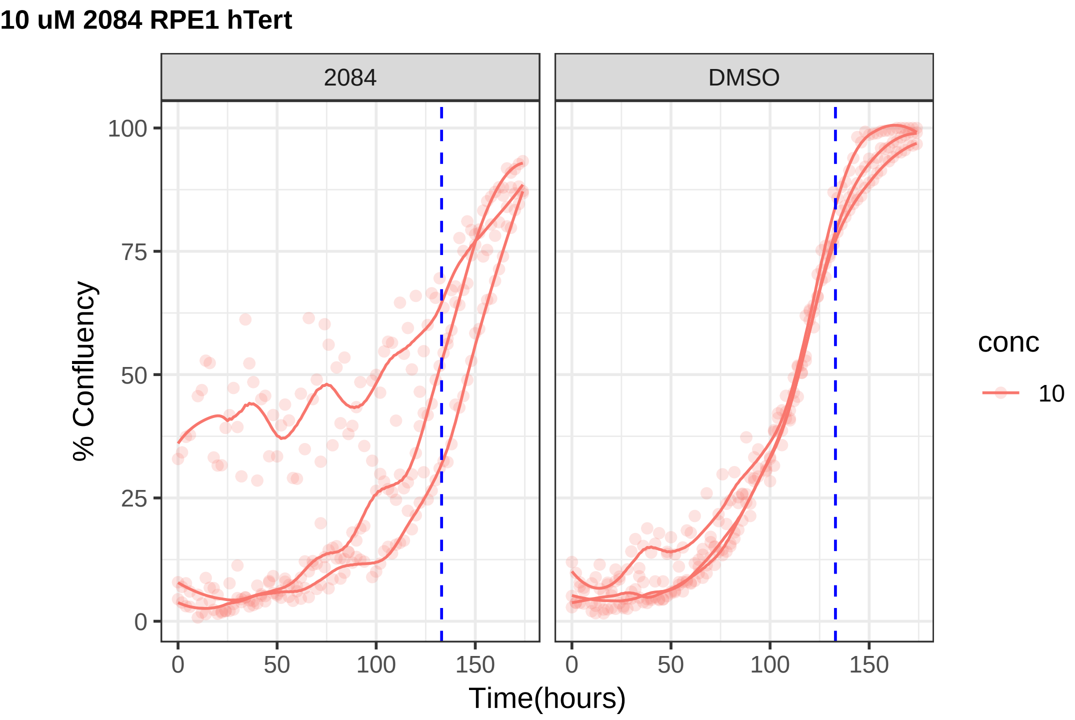

10 uM 2084 RPE1 hTert
2084
DMSO
% Confluency
0
25
50
75
100
0
50
100
150
Time(hours)
conc
10

10 uM 2084 RPE1 Ts12 Ts5

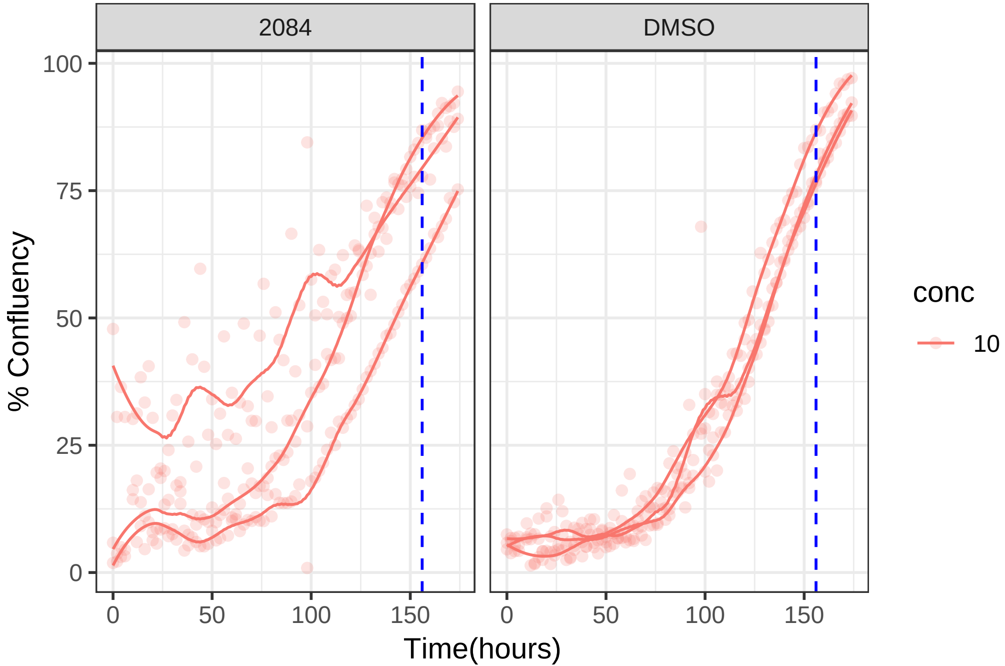

10 uM 2100 RPE1 hTert

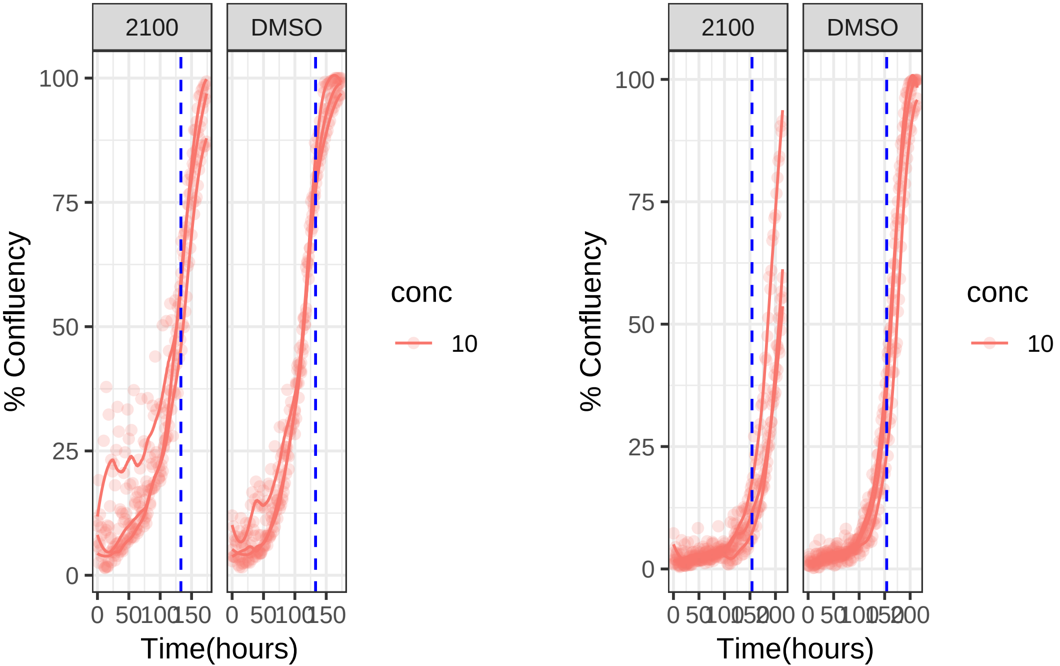

10 uM 2100 RPE1 Ts12 Ts5

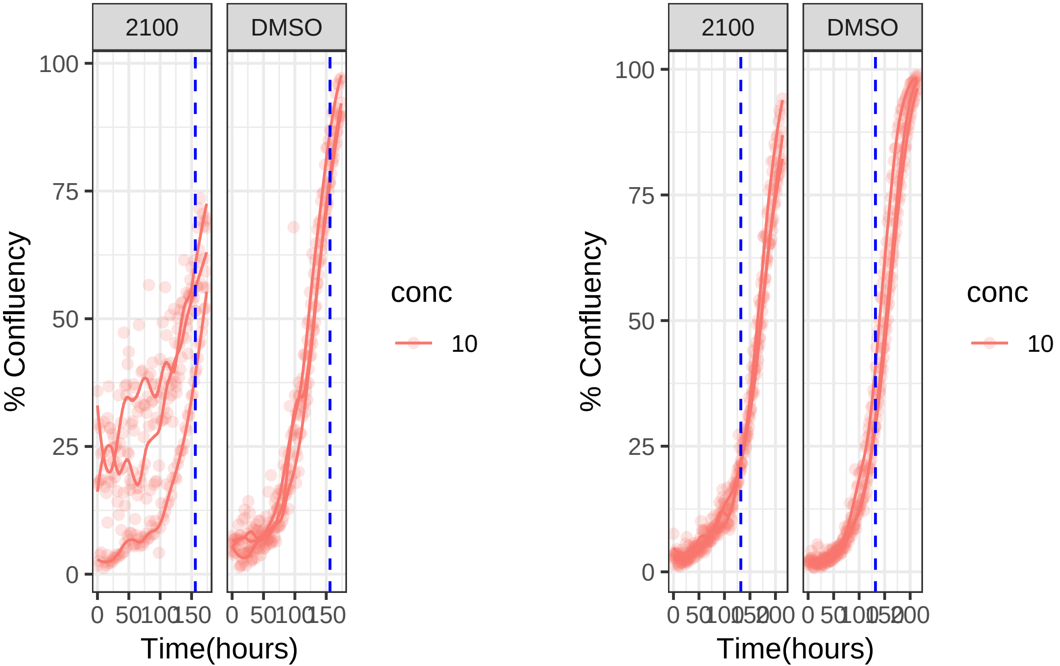

10 uM 2114 RPE1 hTert

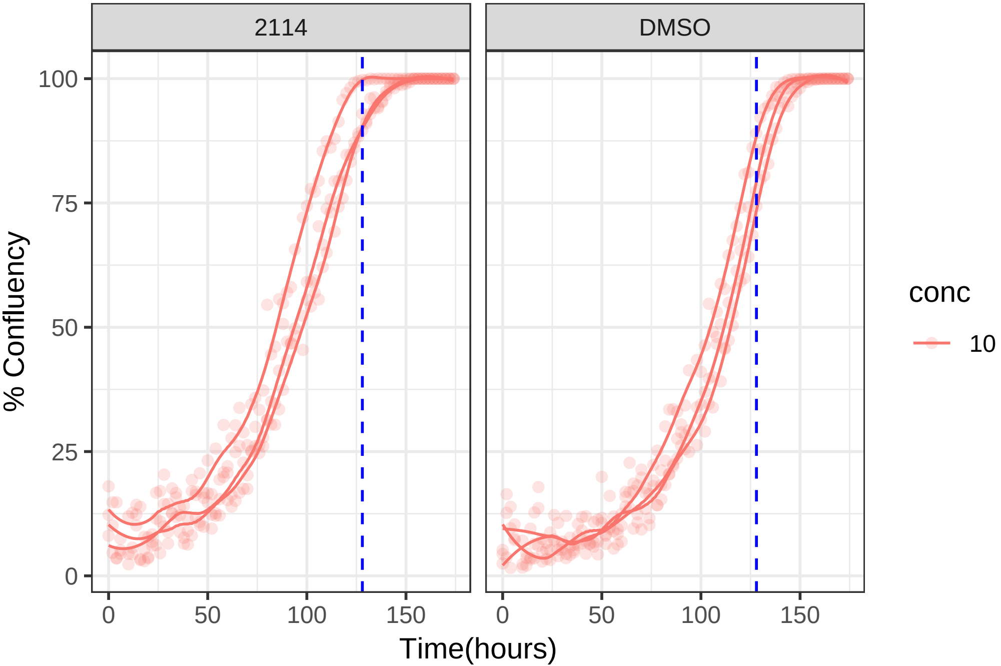

10 uM 2114 RPE1 Ts12 Ts5

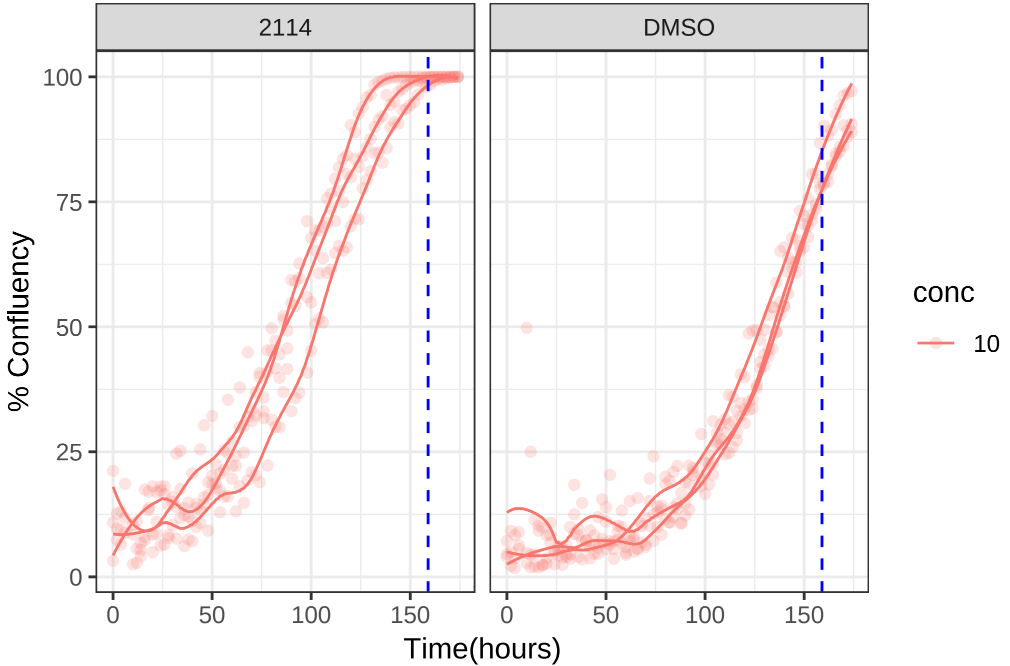

0.01 uM 2120 RPE1 hTert

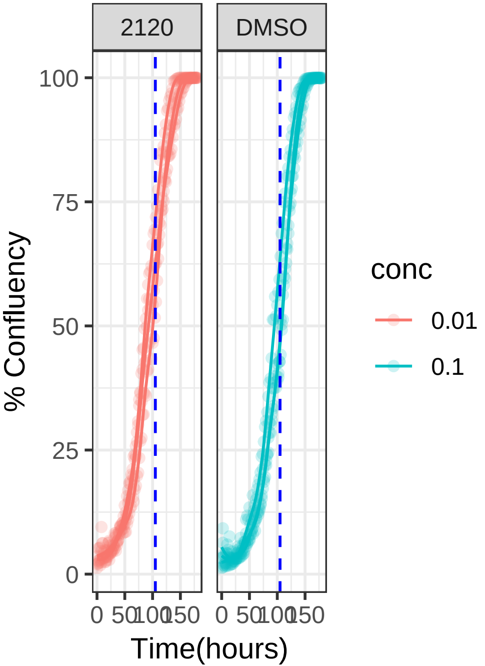

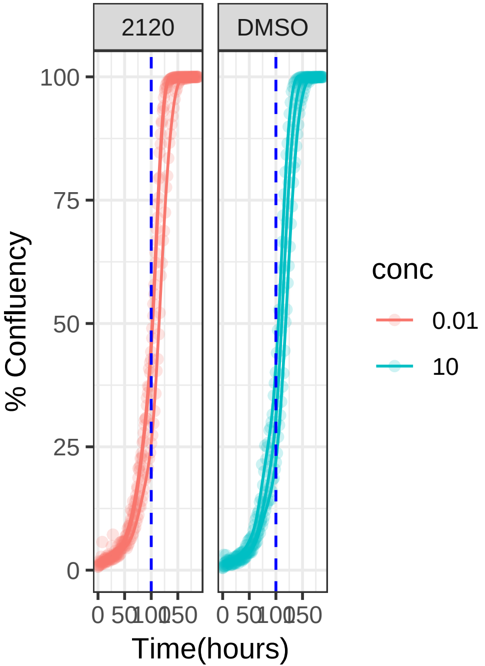

0.01 uM 2120 RPE1 Ts12 Ts5

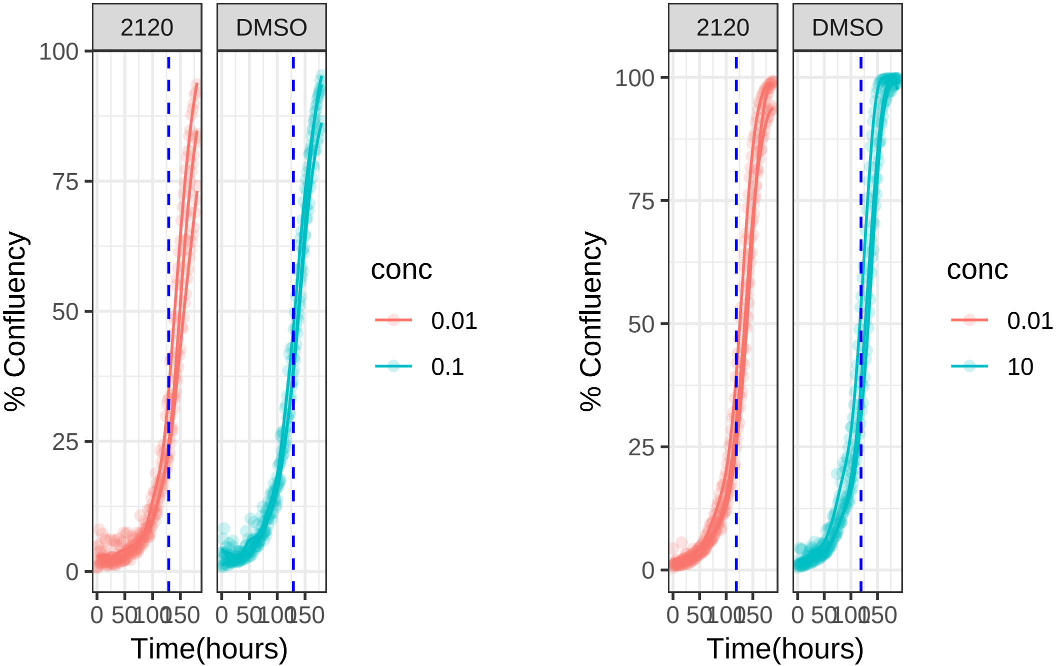

10 uM 2138 RPE1 hTert

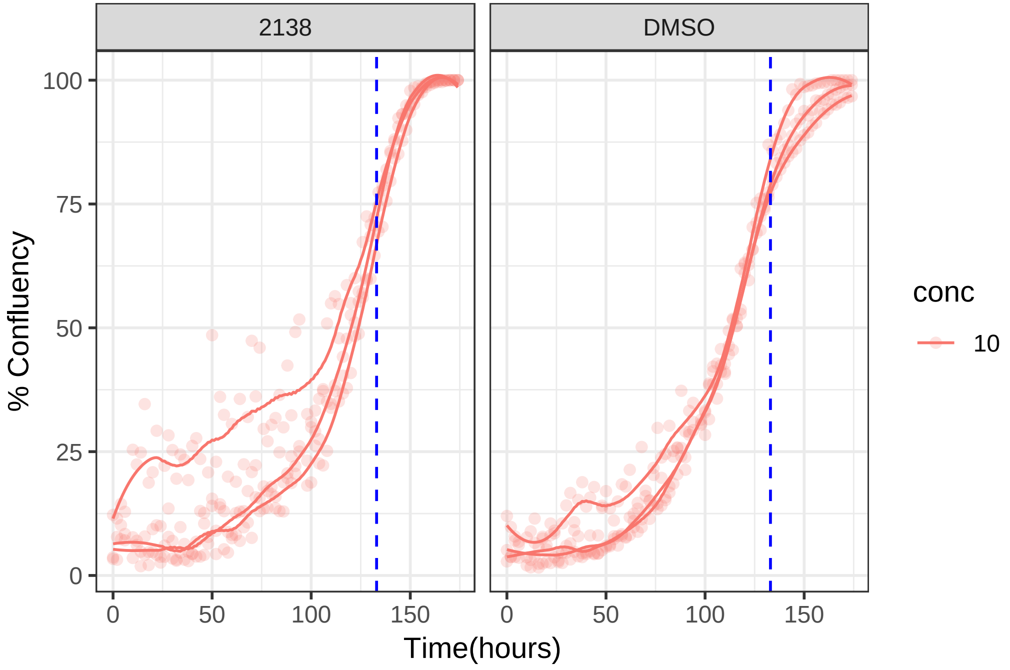

10 uM 2138 RPE1 Ts12 Ts5

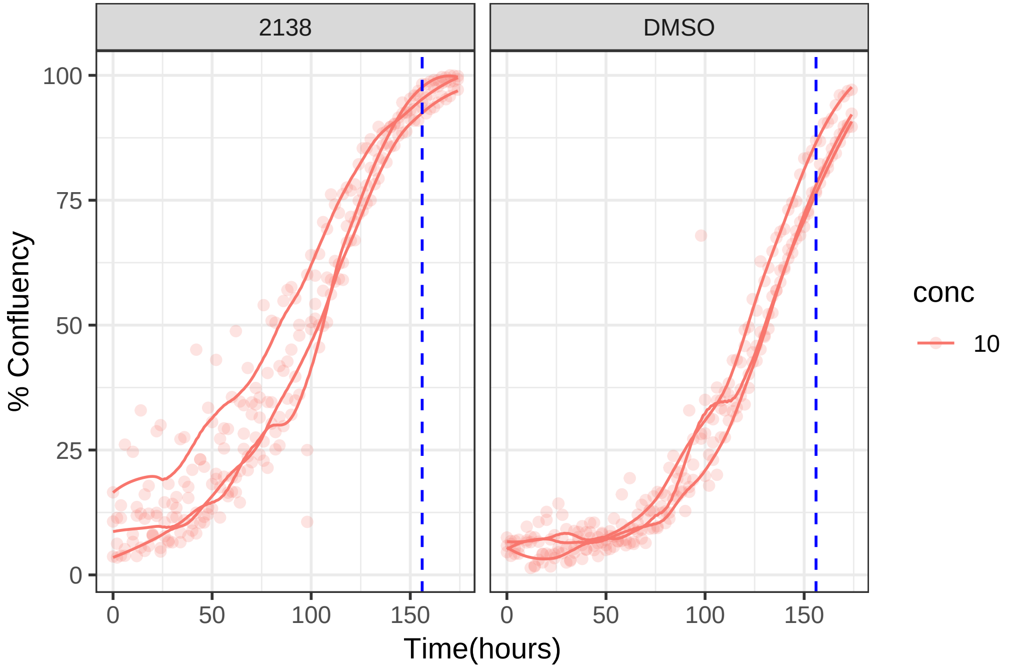

10 uM 2140 RPE1 hTert

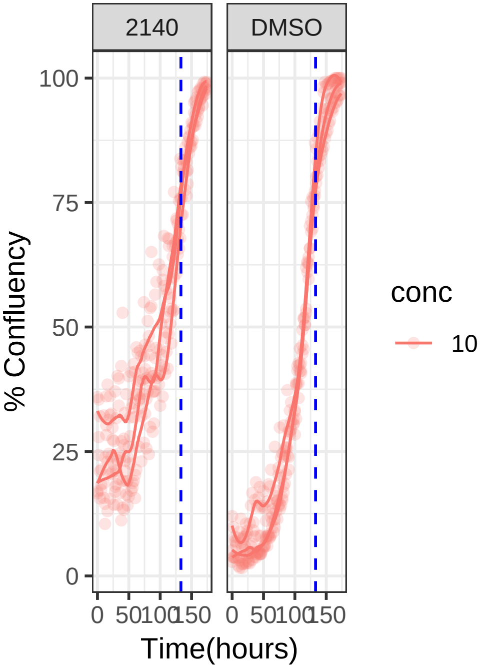

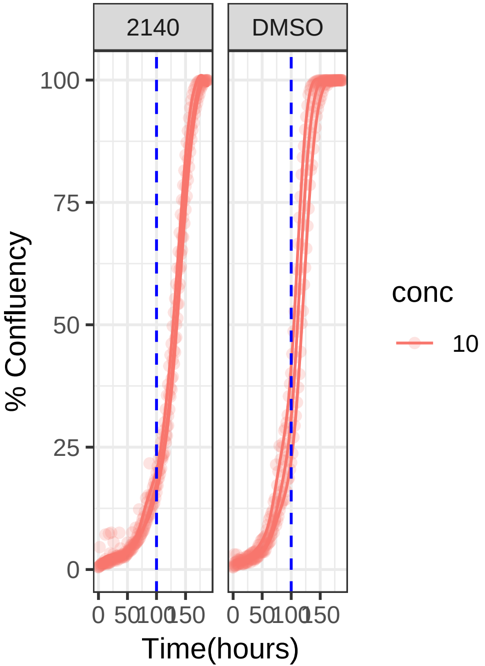

10 uM 2140 RPE1 Ts12 Ts5

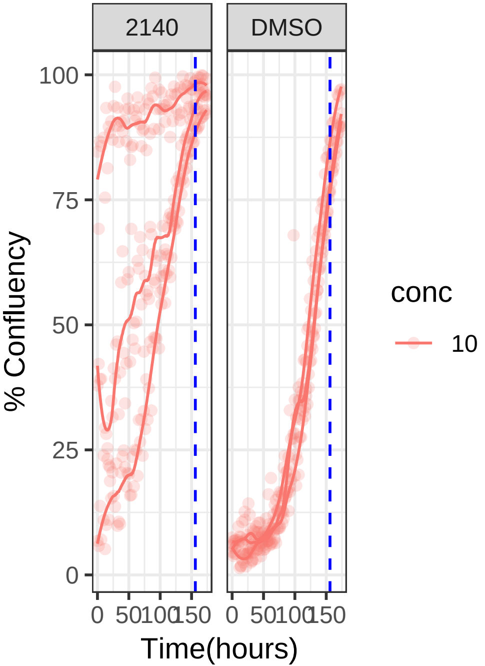

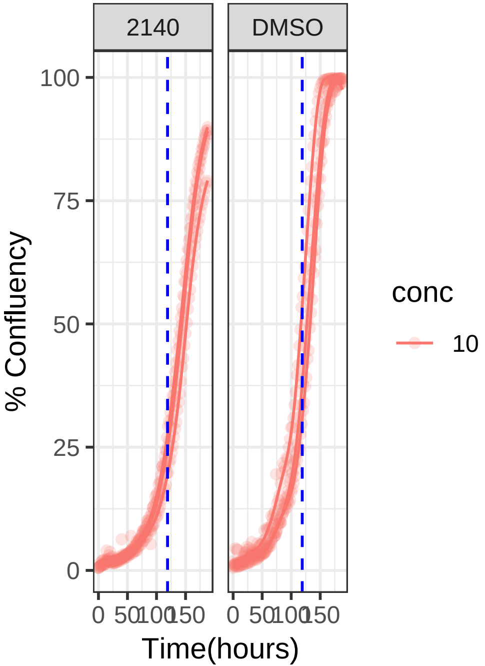

0.01 uM 2142 RPE1 hTert

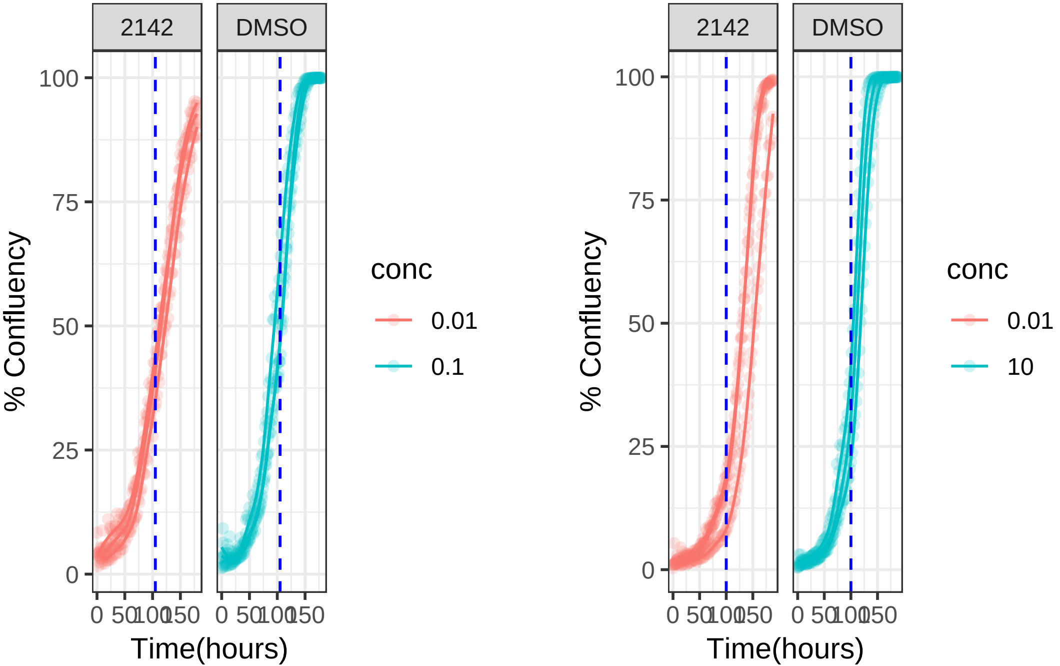

0.01 uM 2142 RPE1 Ts12 Ts5

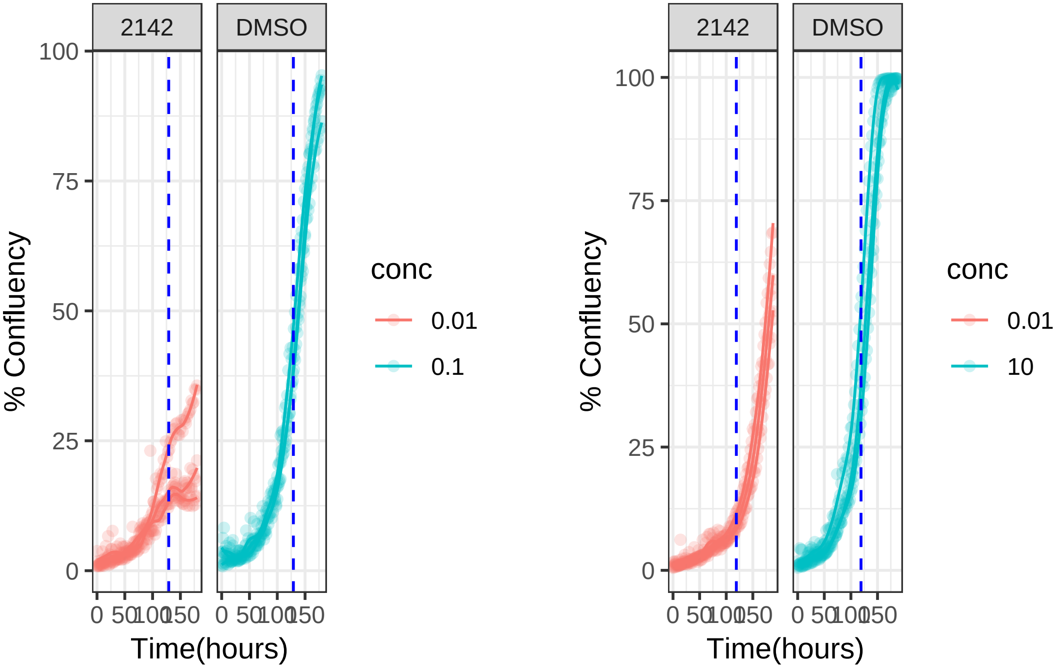

10 uM 2158 RPE1 hTert

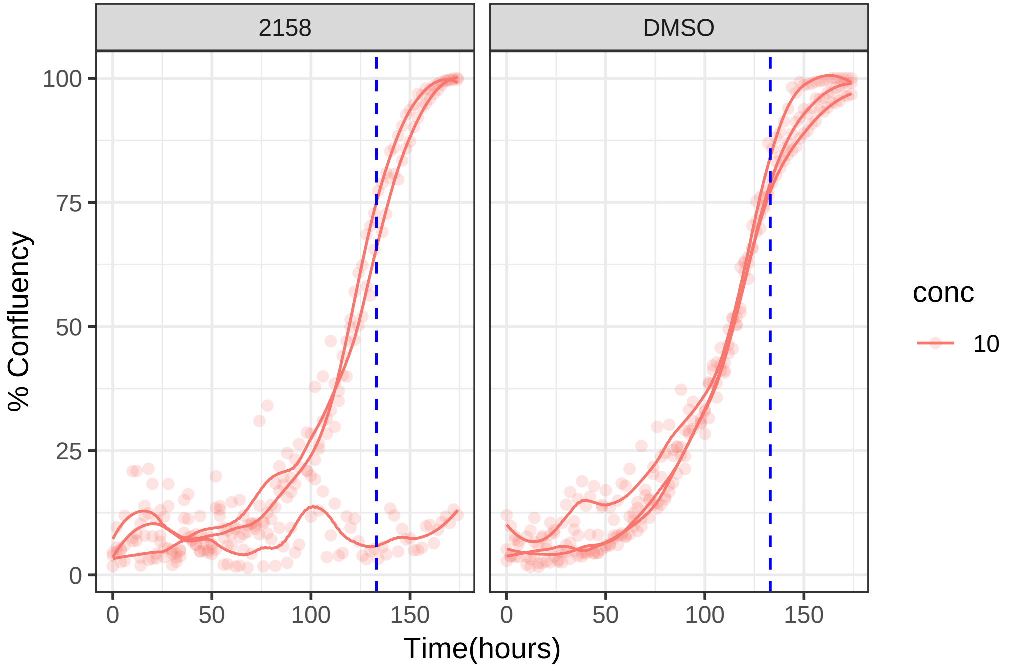

10 uM 2158 RPE1 Ts12 Ts5

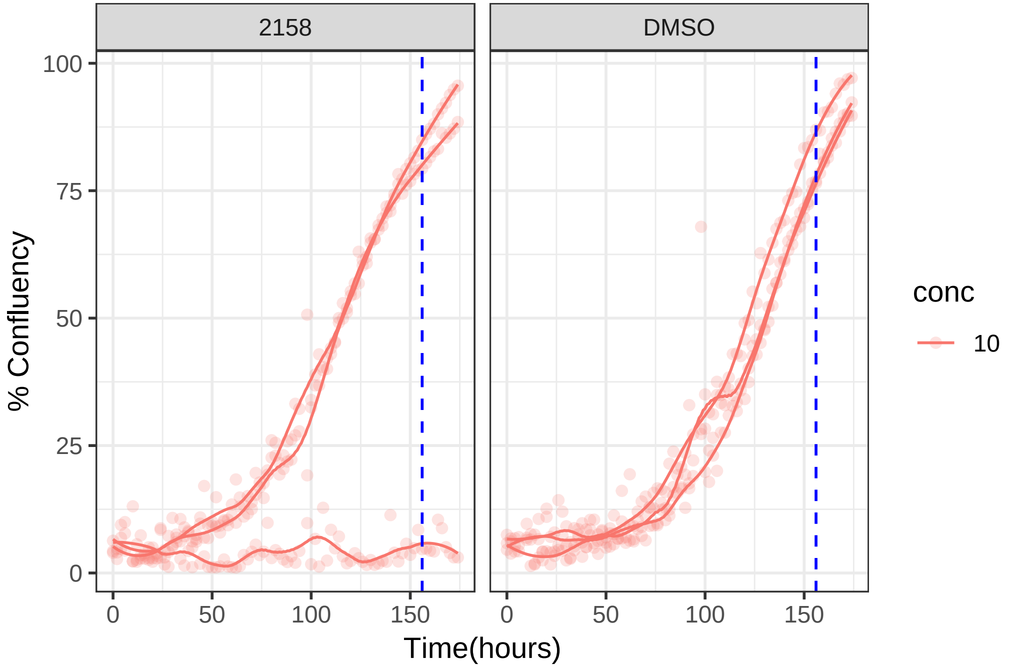

10 uM 2159 RPE1 hTert

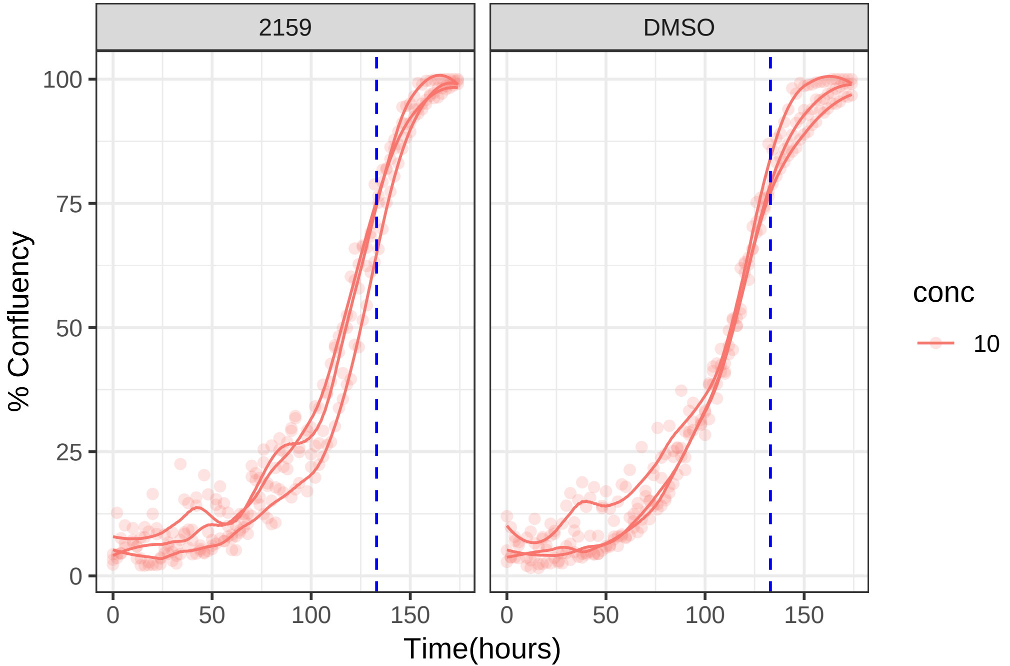

10 uM 2159 RPE1 Ts12 Ts5

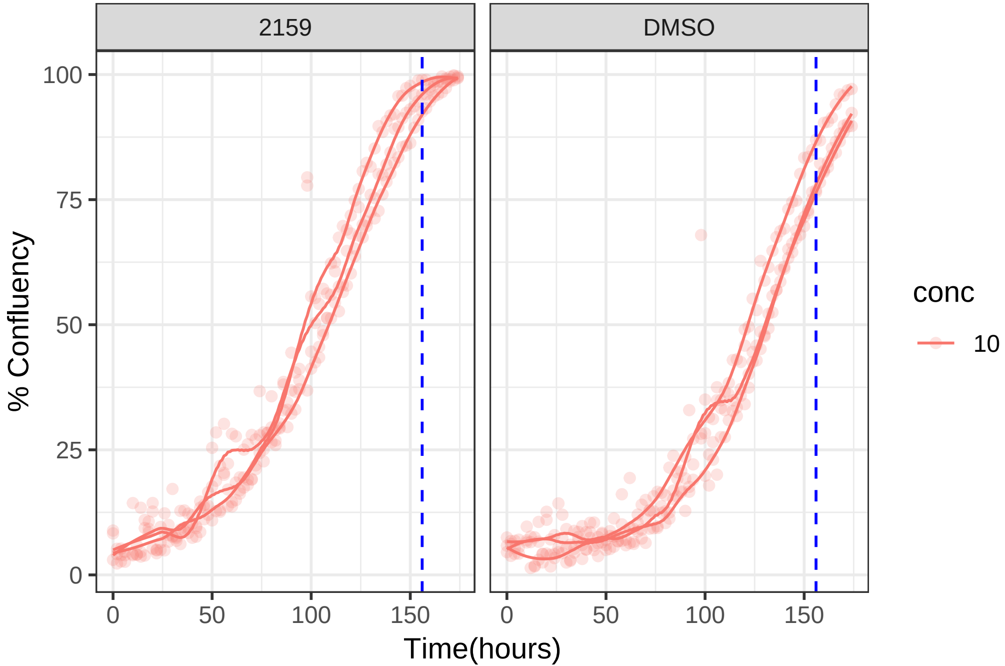

0.1 uM 2160 RPE1 hTert

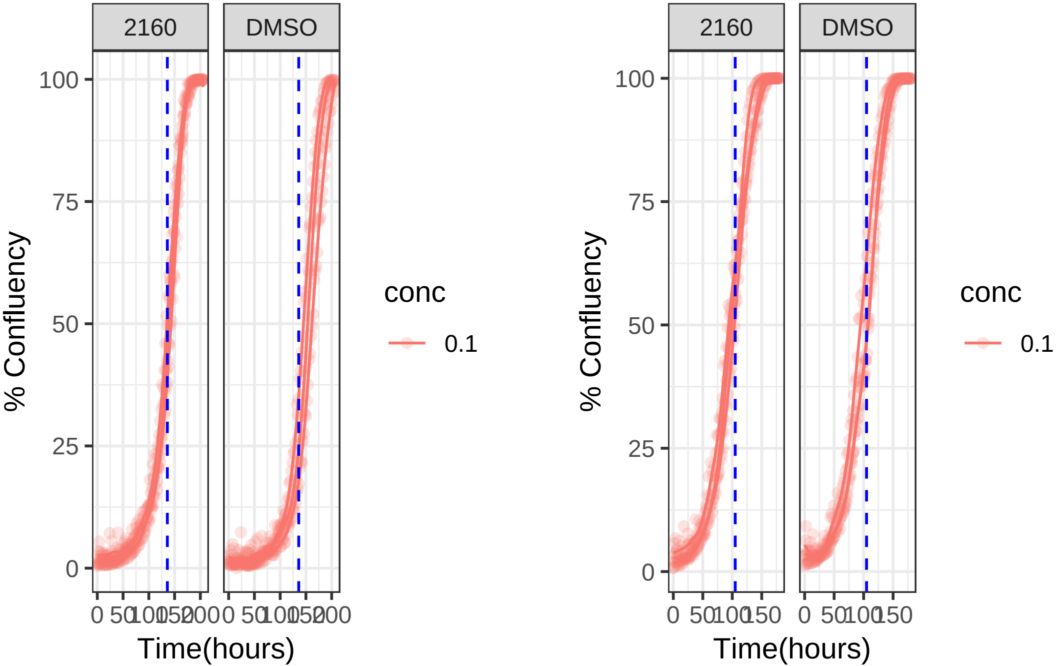

0.1 uM 2160 RPE1 Ts12 Ts5

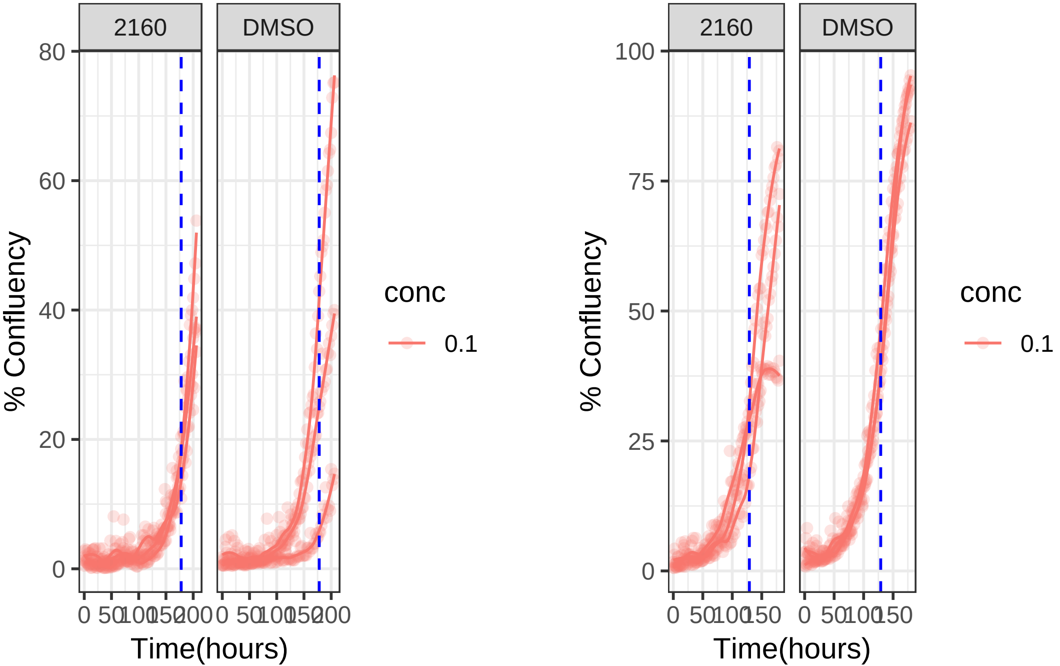

10 uM 2165 RPE1 hTert

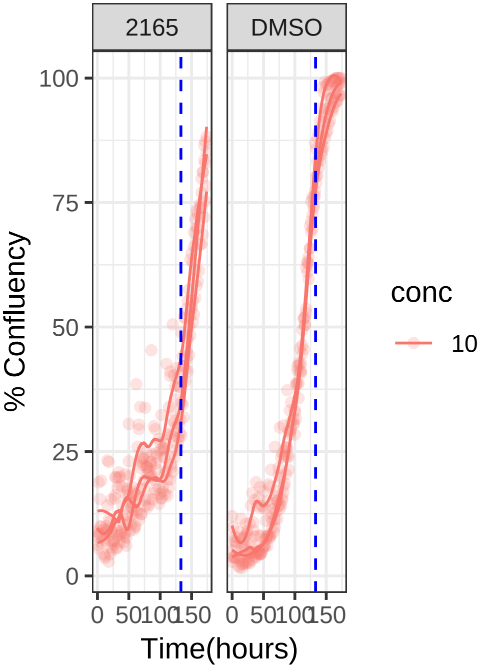

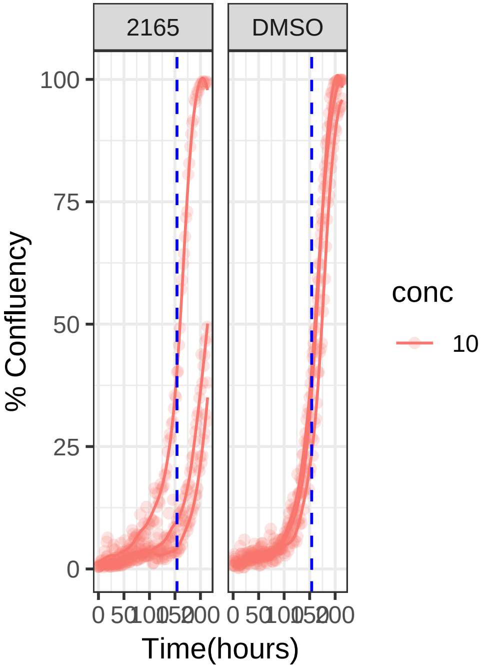

10 uM 2165 RPE1 Ts12 Ts5

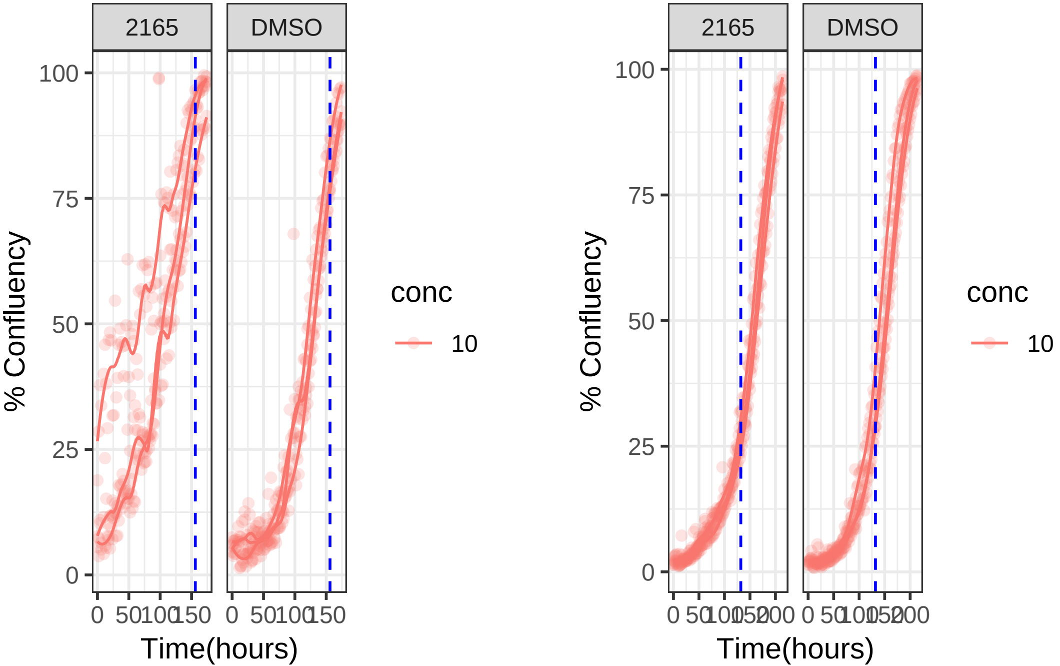

10 uM 2168 RPE1 hTert

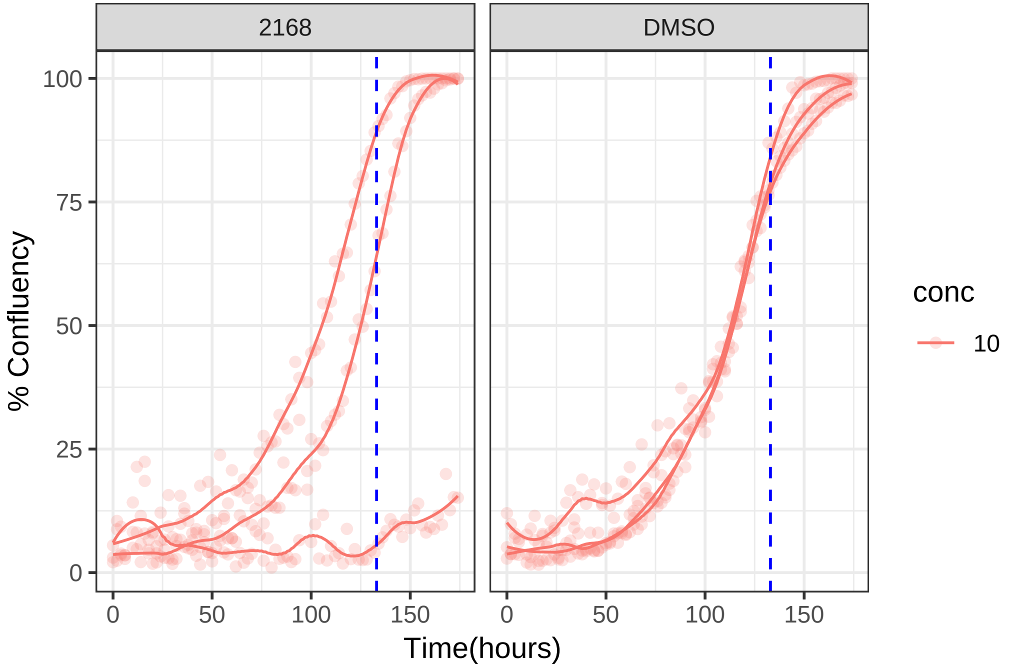

10 uM 2168 RPE1 Ts12 Ts5

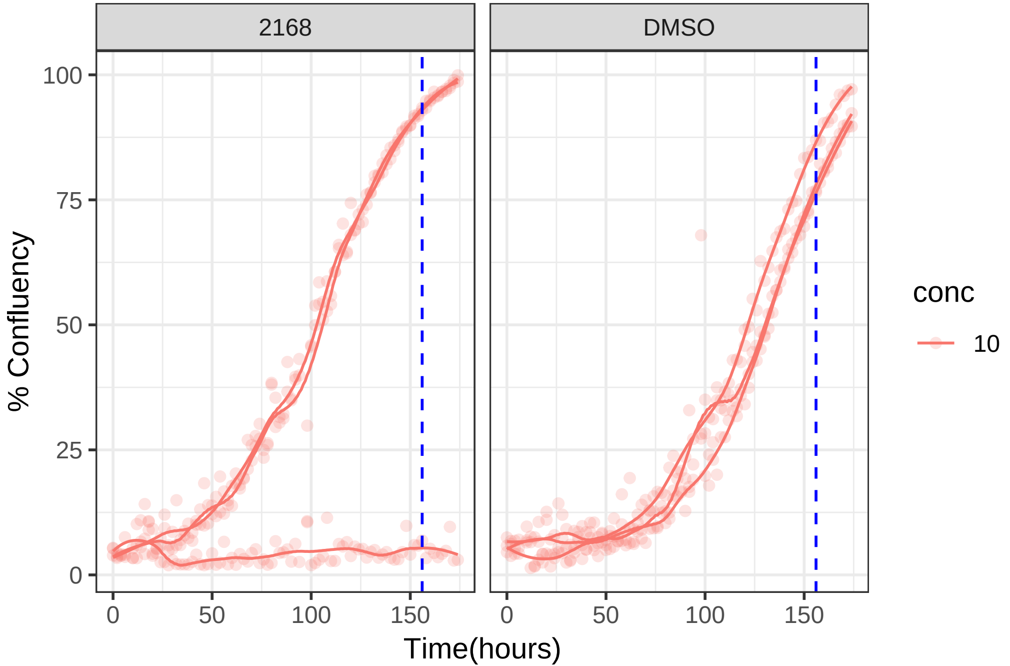

10 uM 2170 RPE1 hTert

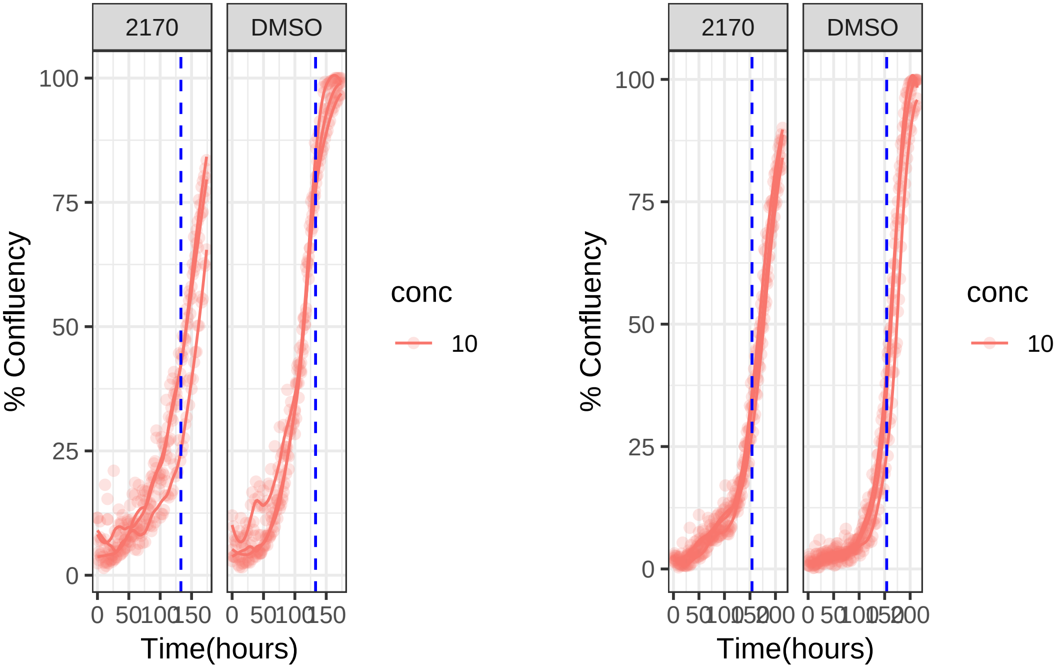

10 uM 2170 RPE1 Ts12 Ts5

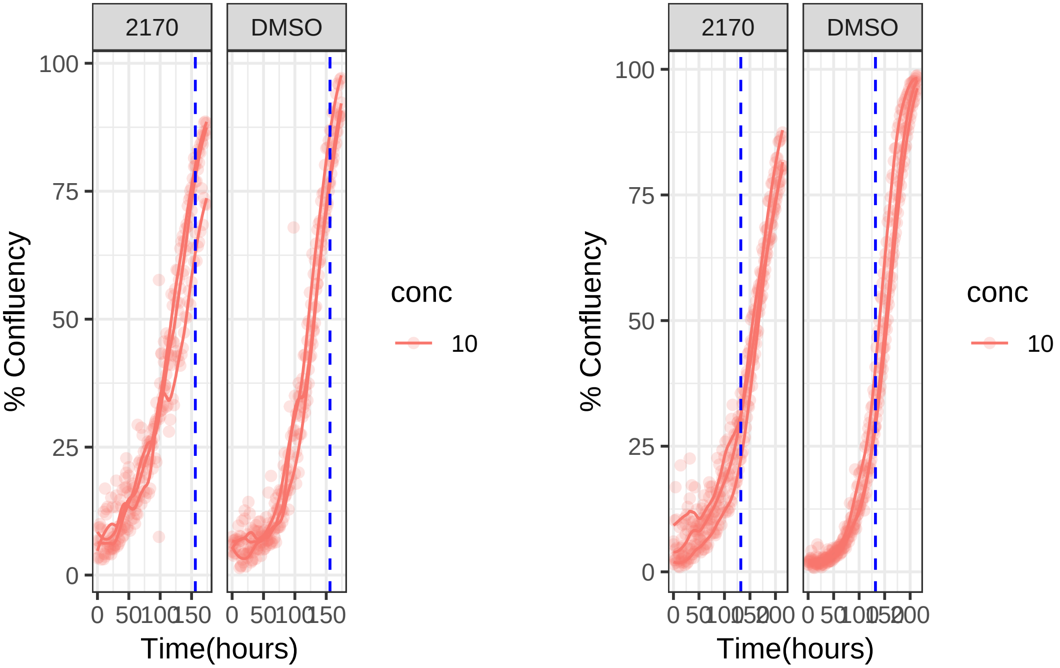

10 uM 2180 RPE1 hTert

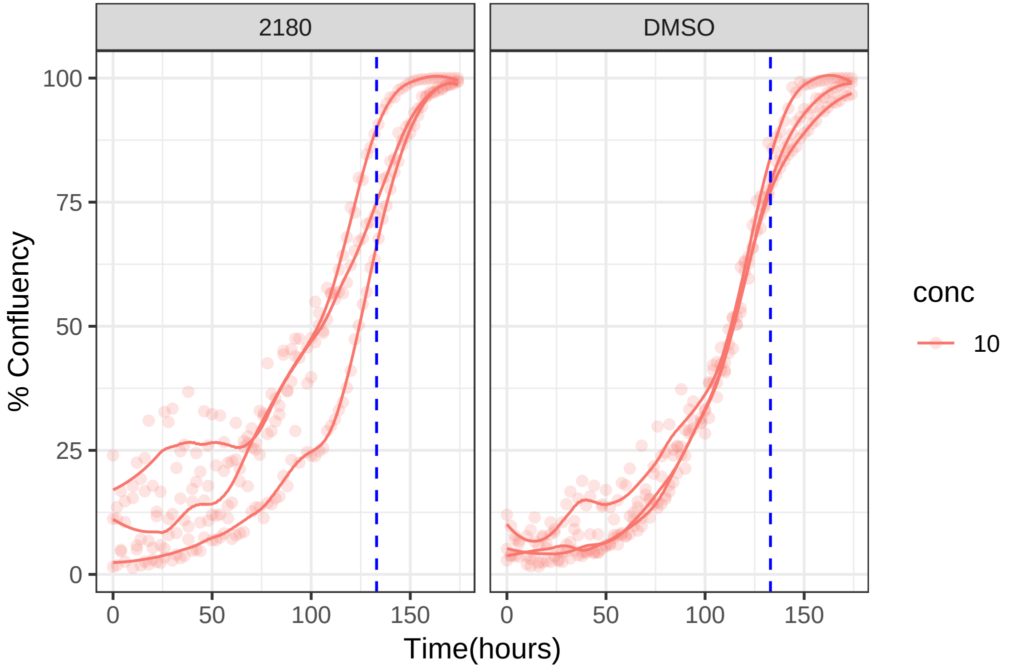

10 uM 2180 RPE1 Ts12 Ts5

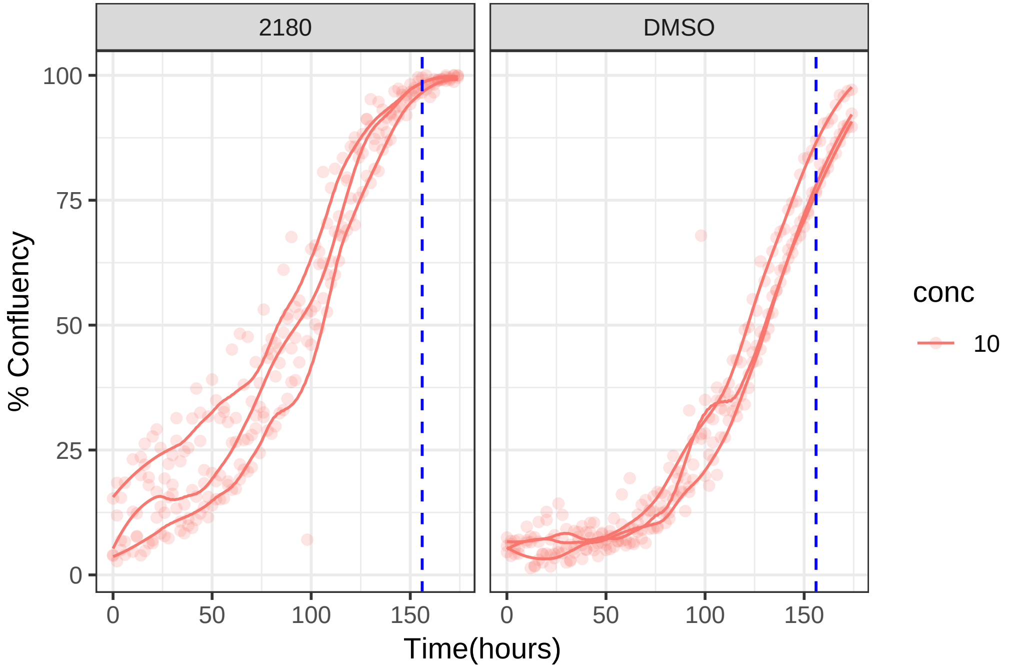

10 uM 2185 RPE1 hTert

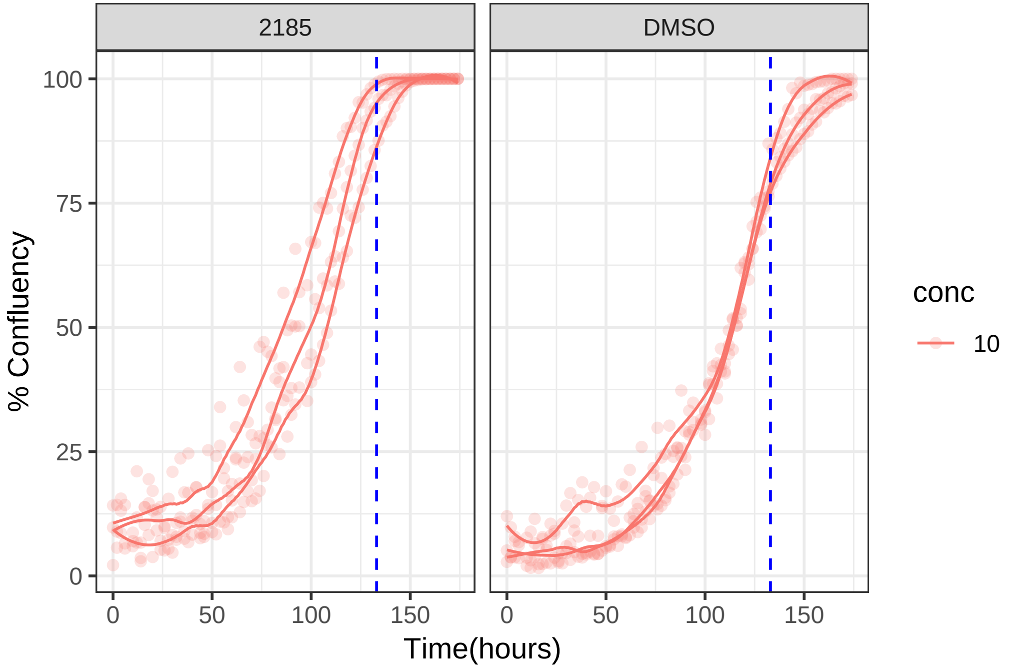

10 uM 2185 RPE1 Ts12 Ts5

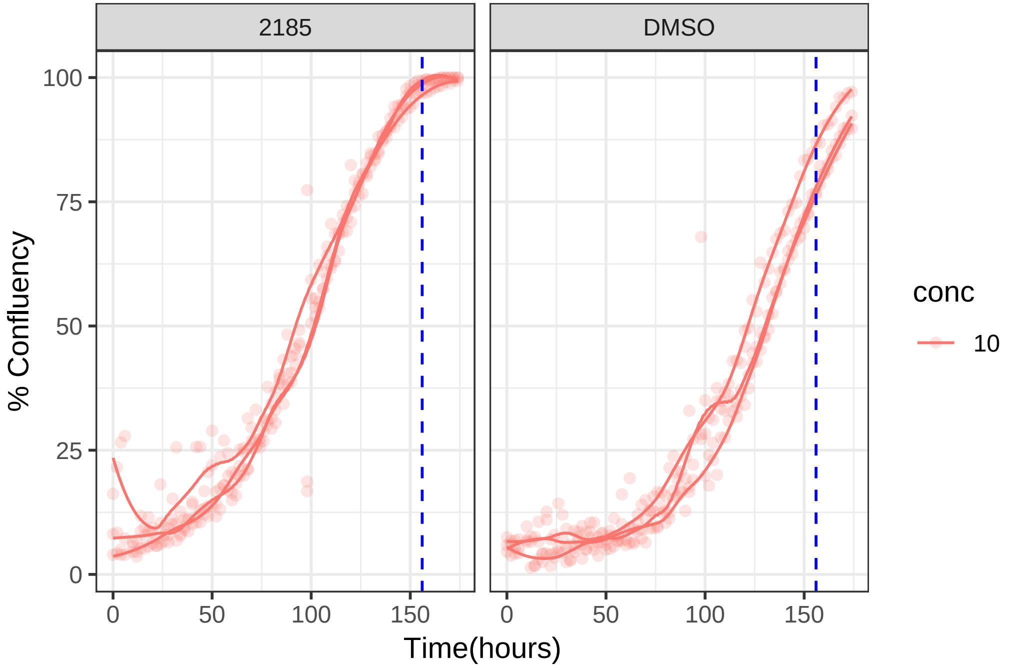

1 uM 2188 RPE1 hTert

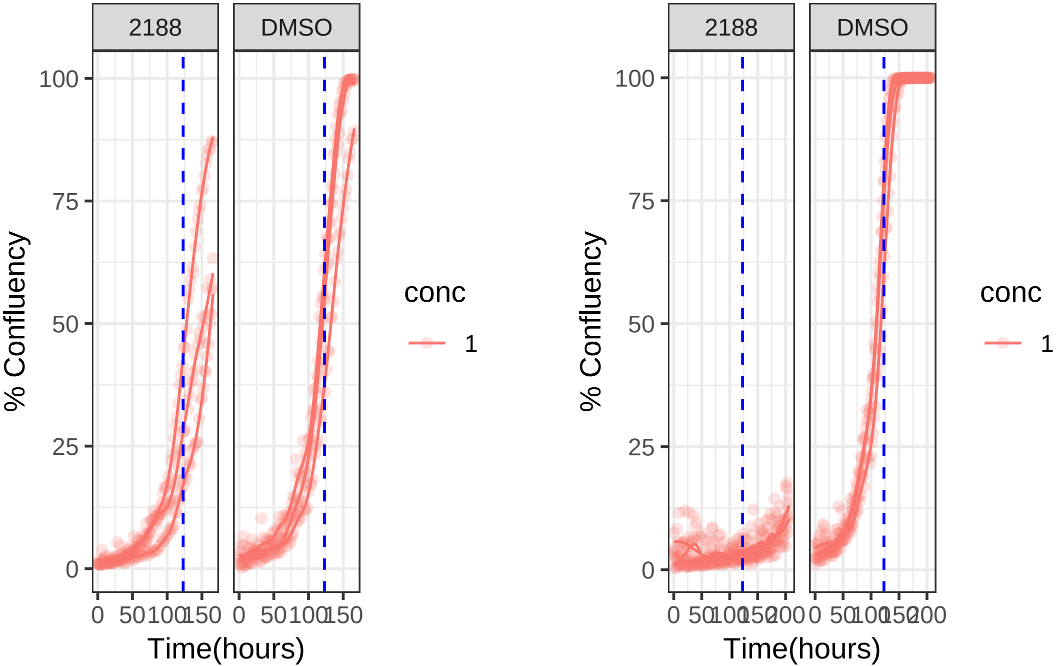

1 uM 2188 RPE1 Ts12 Ts5

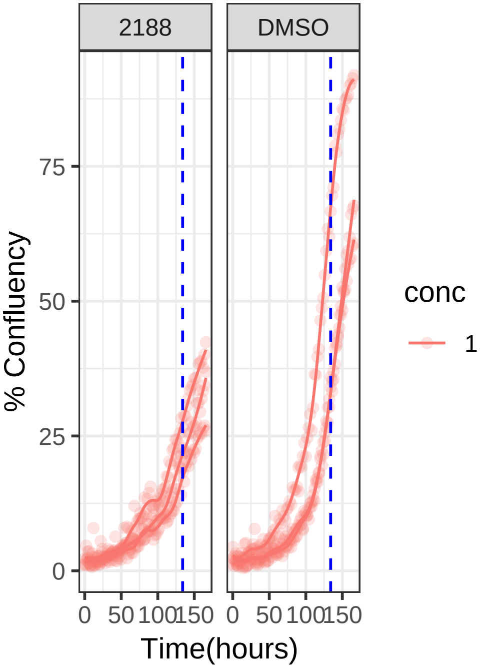

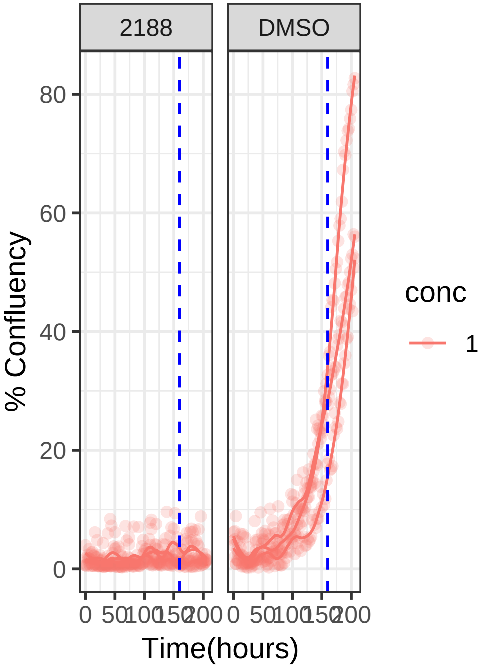

1 uM 2195 RPE1 hTert

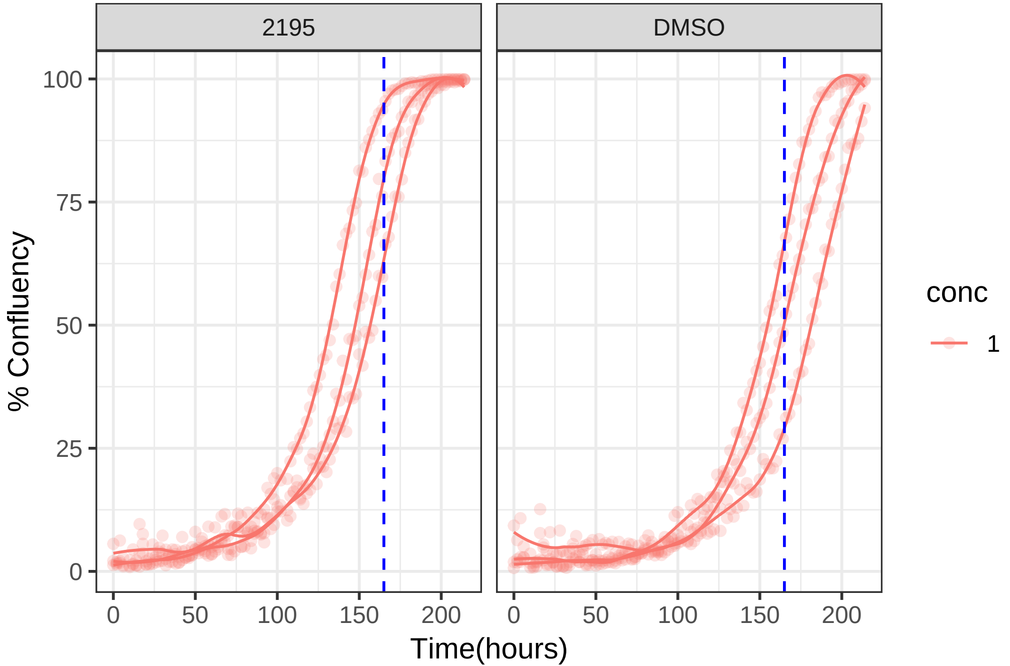

1 uM 2195 RPE1 Ts12 Ts5

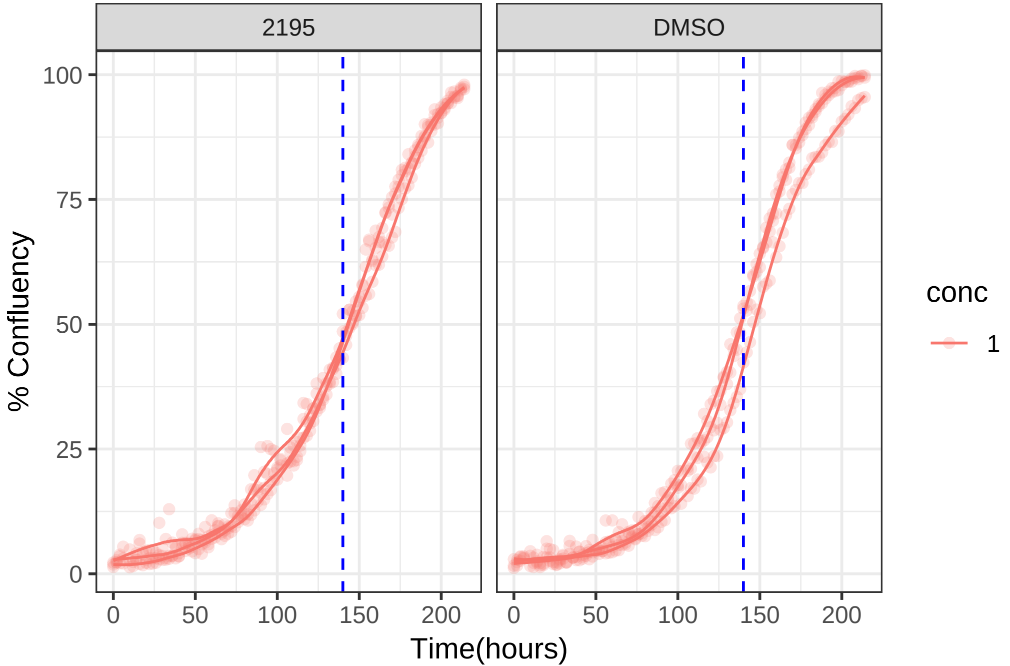

0.001 uM 2198 RPE1 hTert

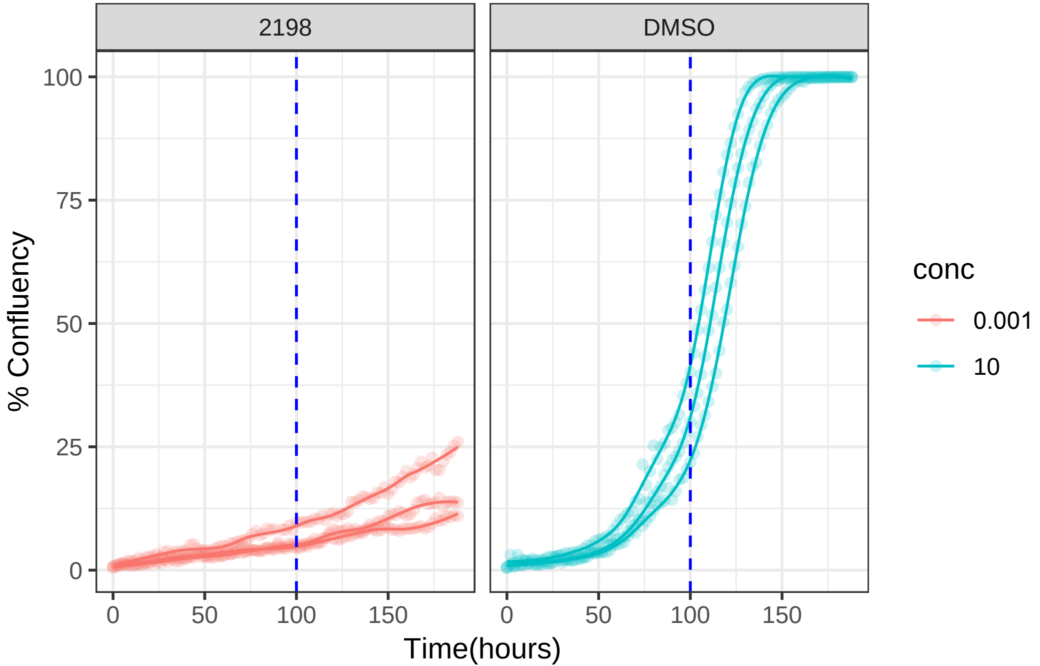

0.001 uM 2198 RPE1 Ts12 Ts5

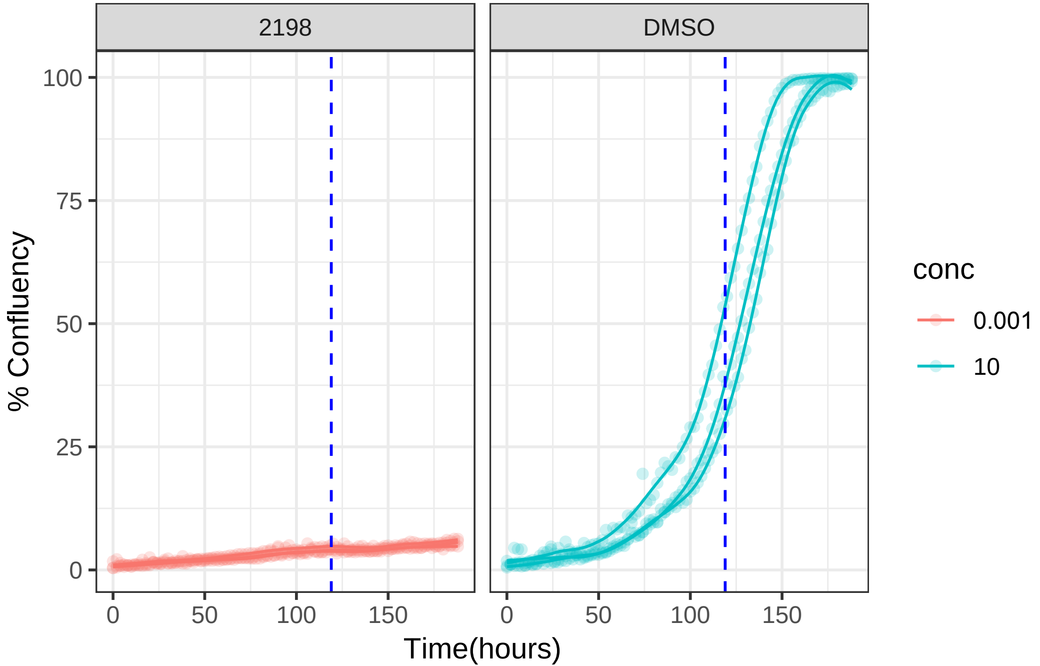

10 uM 2209 RPE1 hTert

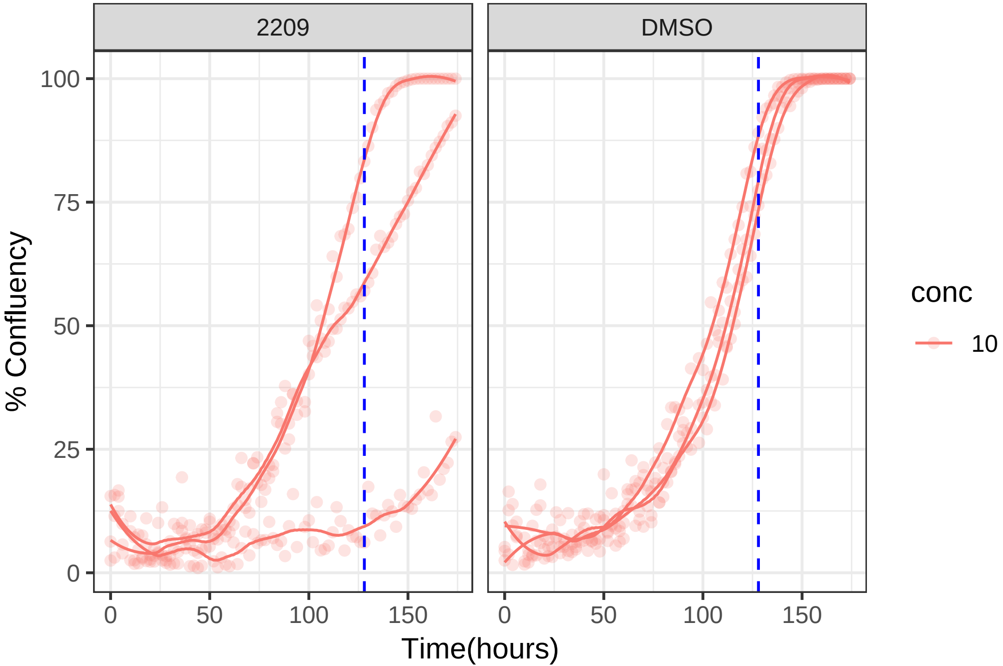

10 uM 2209 RPE1 Ts12 Ts5

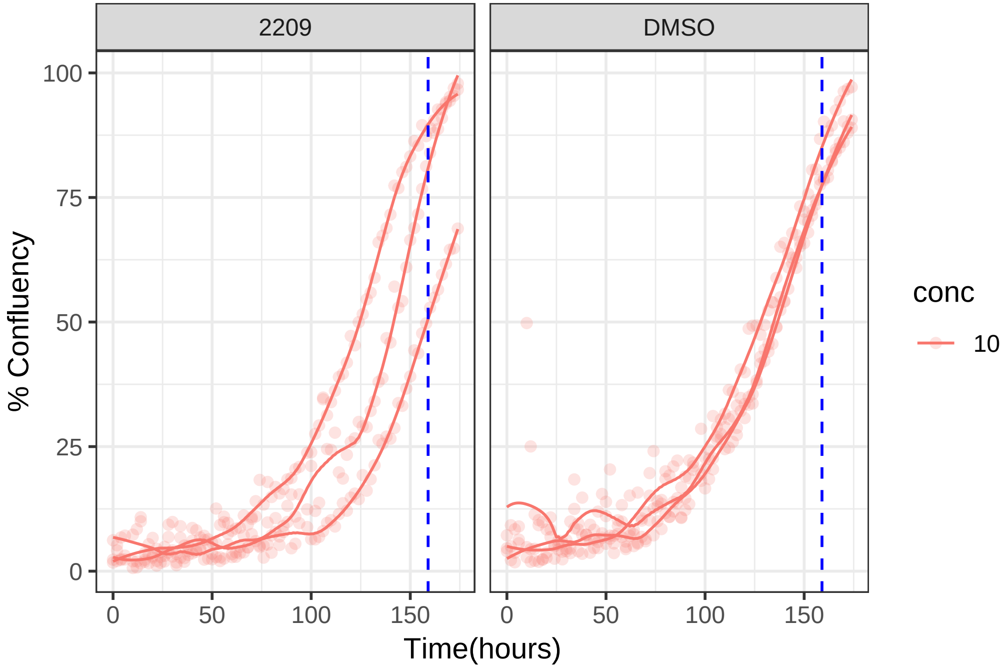

10 uM 2210 RPE1 hTert

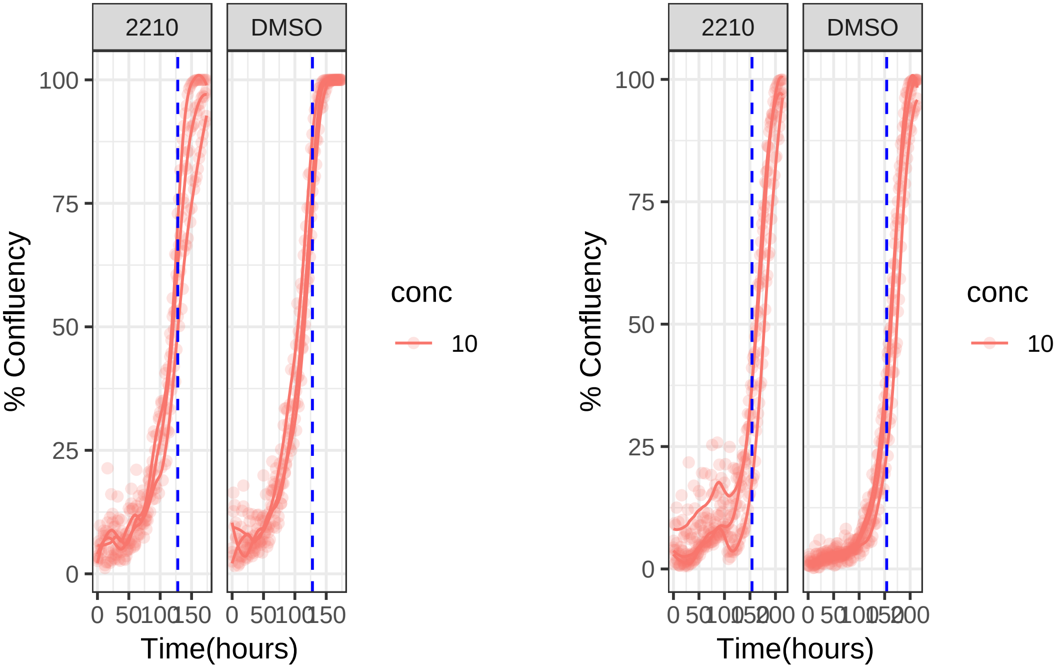

10 uM 2210 RPE1 Ts12 Ts5

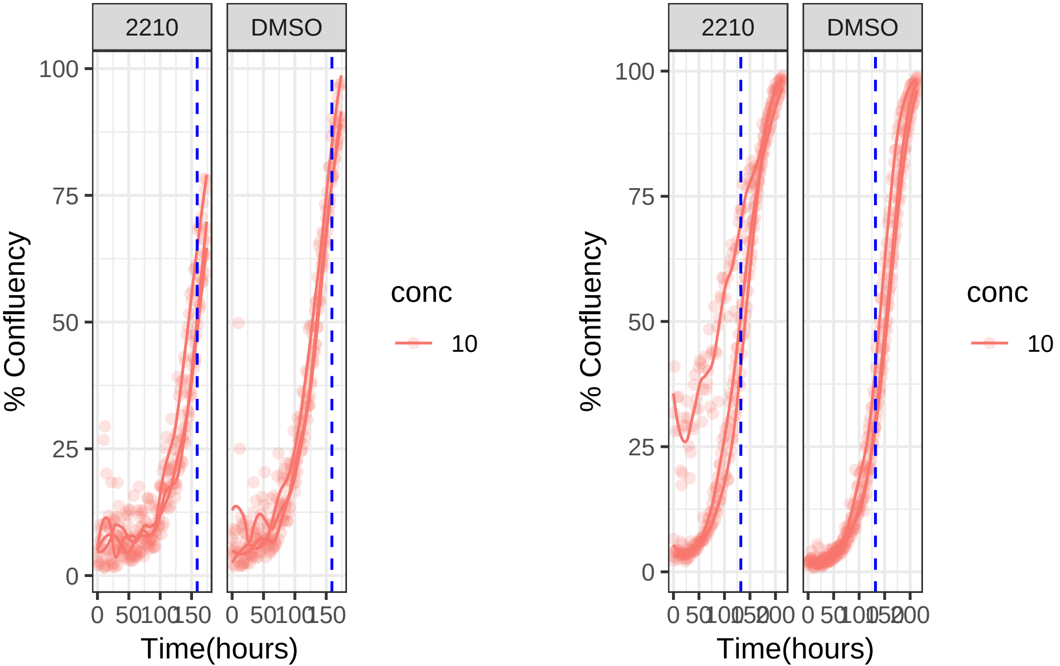

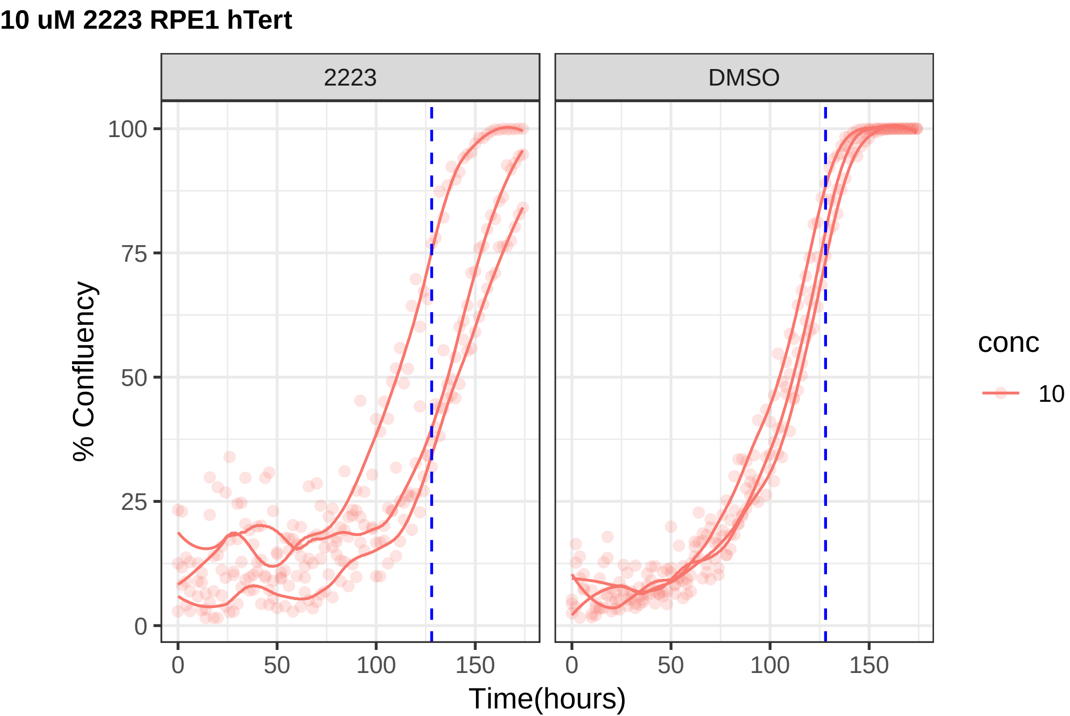

10 uM 2223 RPE1 hTert
2223
DMSO
100
75
50
25
0
0
50
100
150
% Confluency
Time(hours)
conc
10

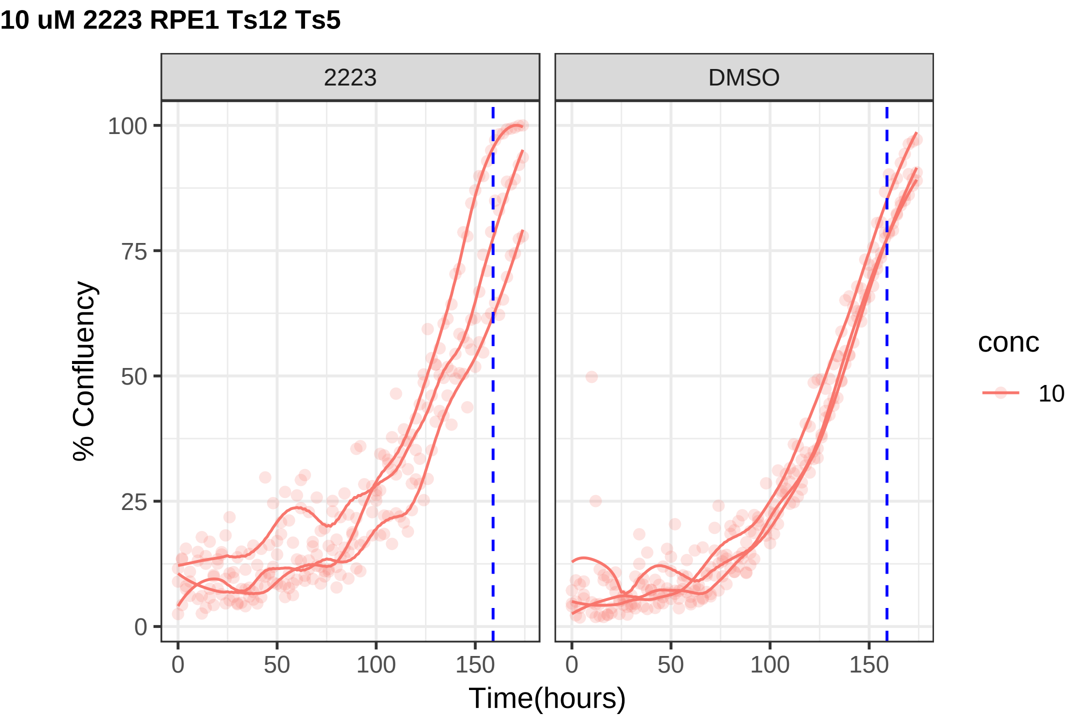

10 uM 2223 RPE1 Ts12 Ts5
2223
DMSO
% Confluency
100
75
50
25
0
0
50
100
150
Time(hours)
conc
10

10 uM 2227 RPE1 hTert

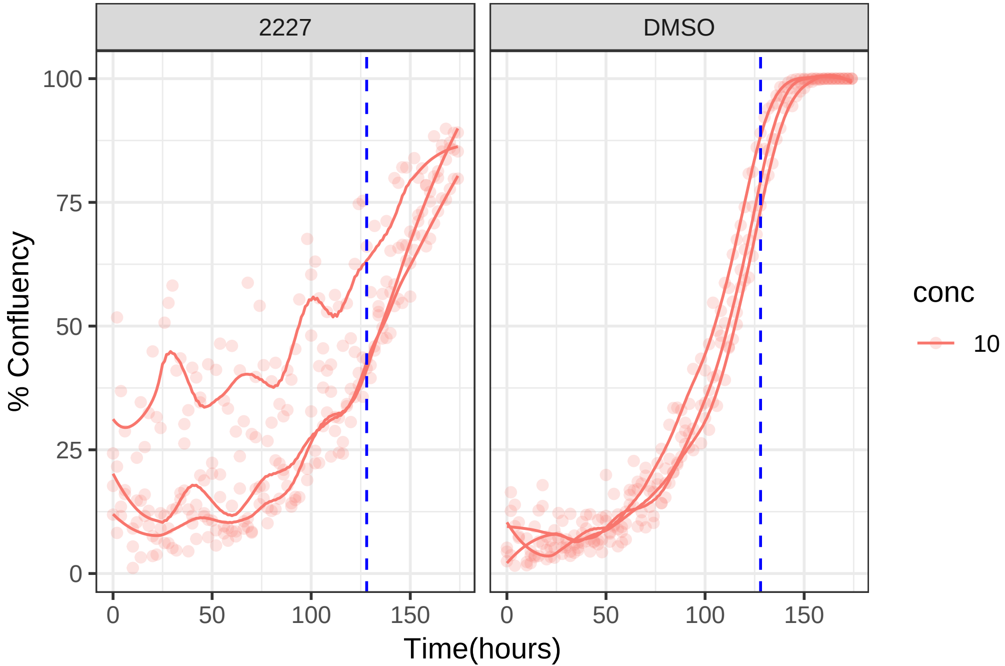

10 uM 2227 RPE1 Ts12 Ts5

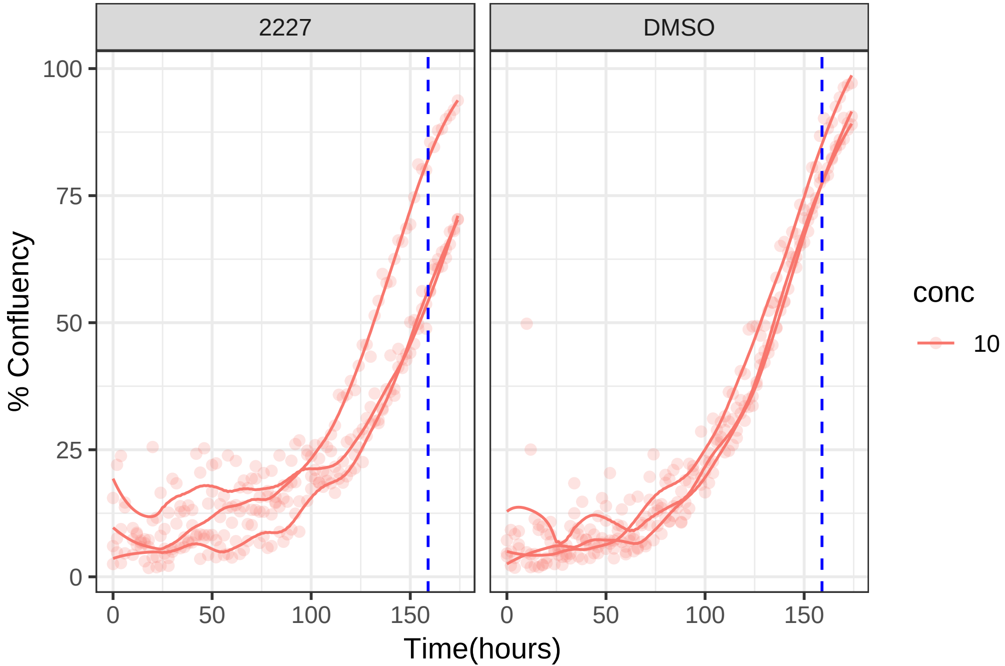

1 uM 2241 RPE1 hTert

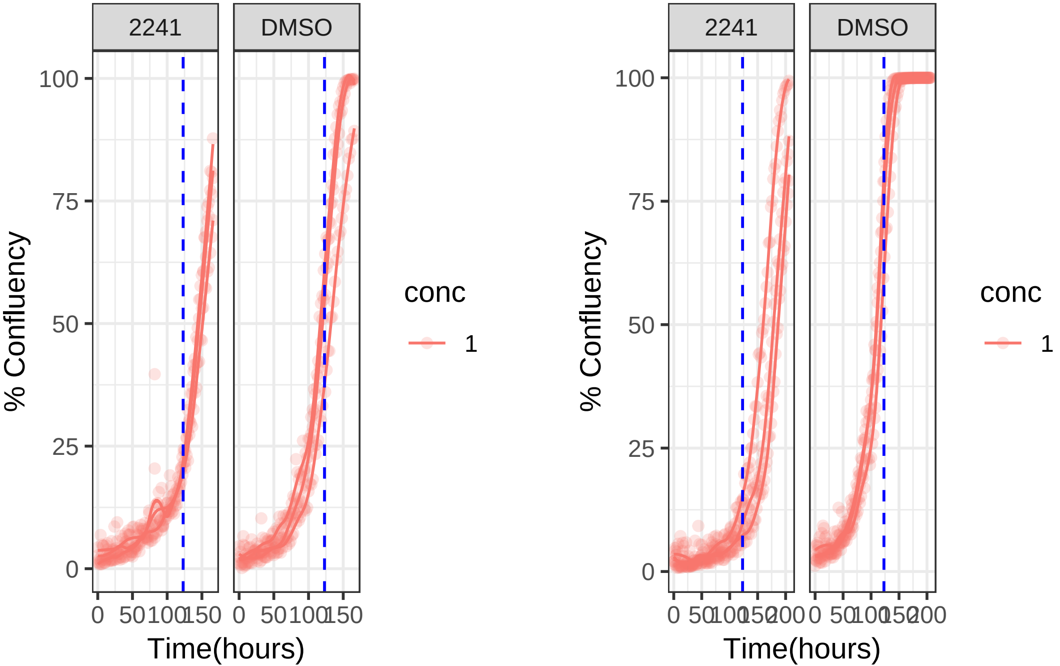

1 uM 2241 RPE1 Ts12 Ts5

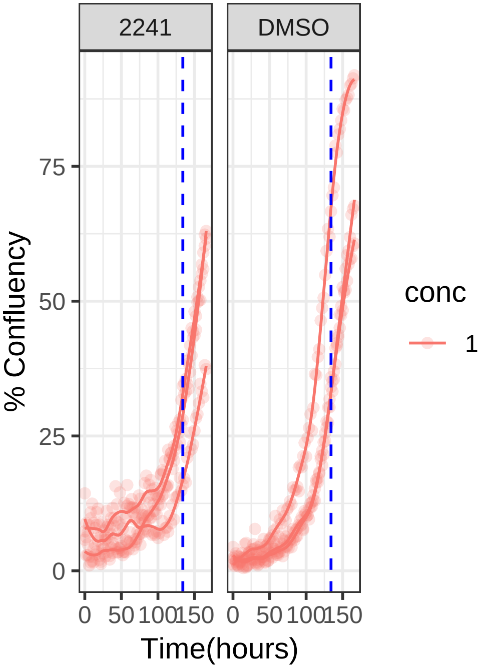

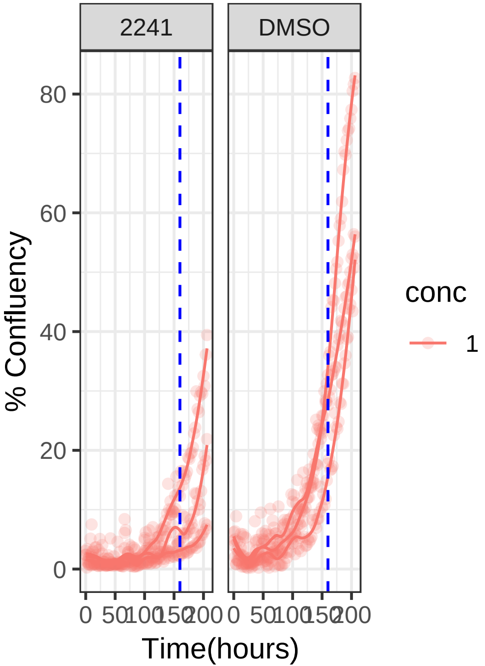

10 uM 2242 RPE1 hTert

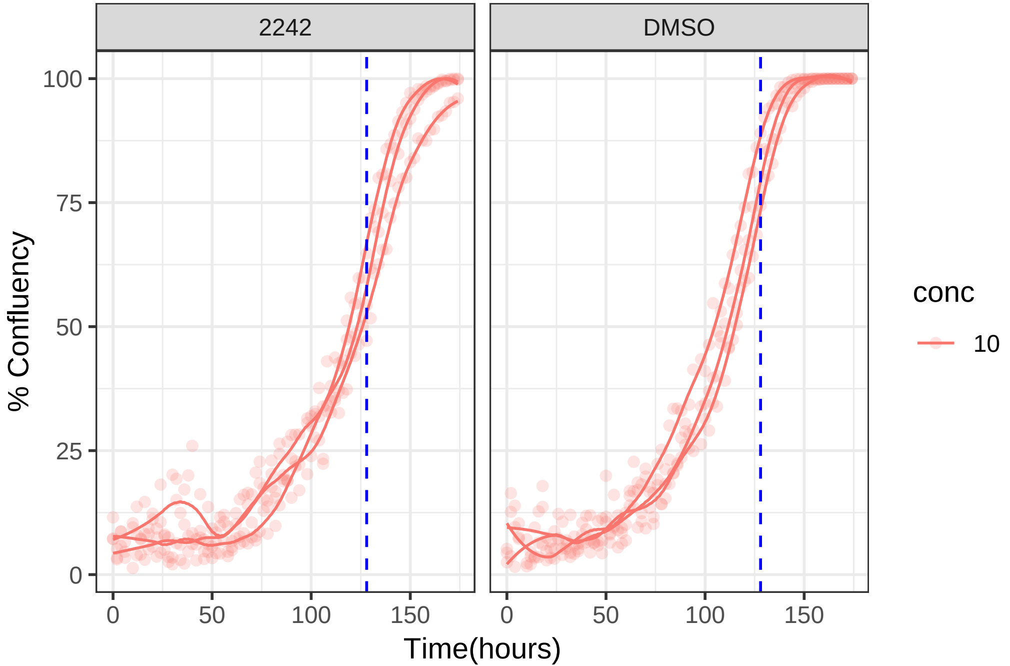

10 uM 2242 RPE1 Ts12 Ts5

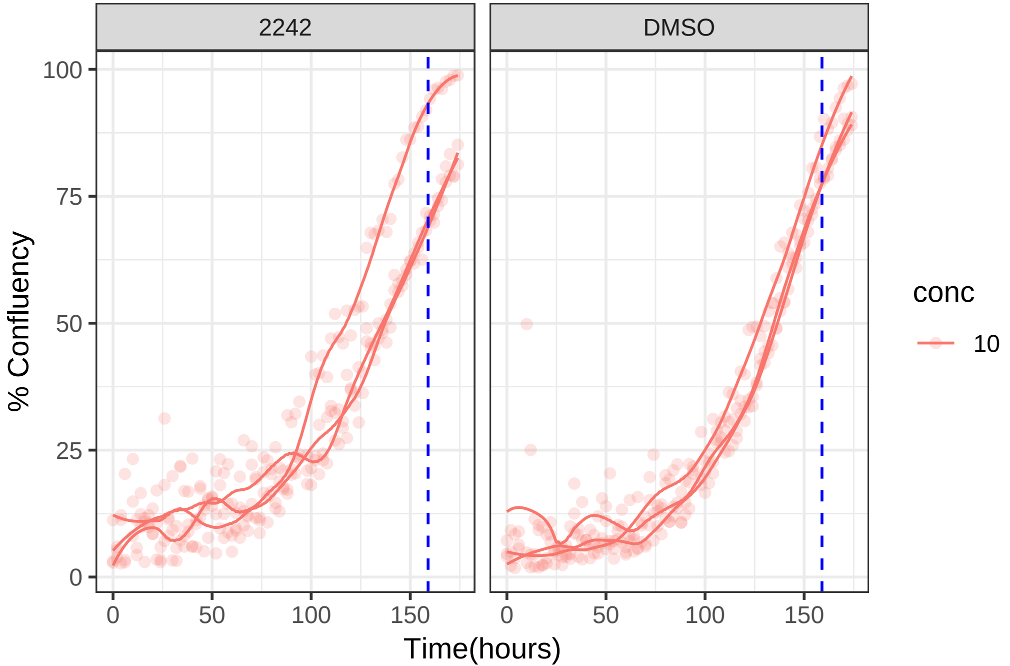

1 uM 2244 RPE1 hTert

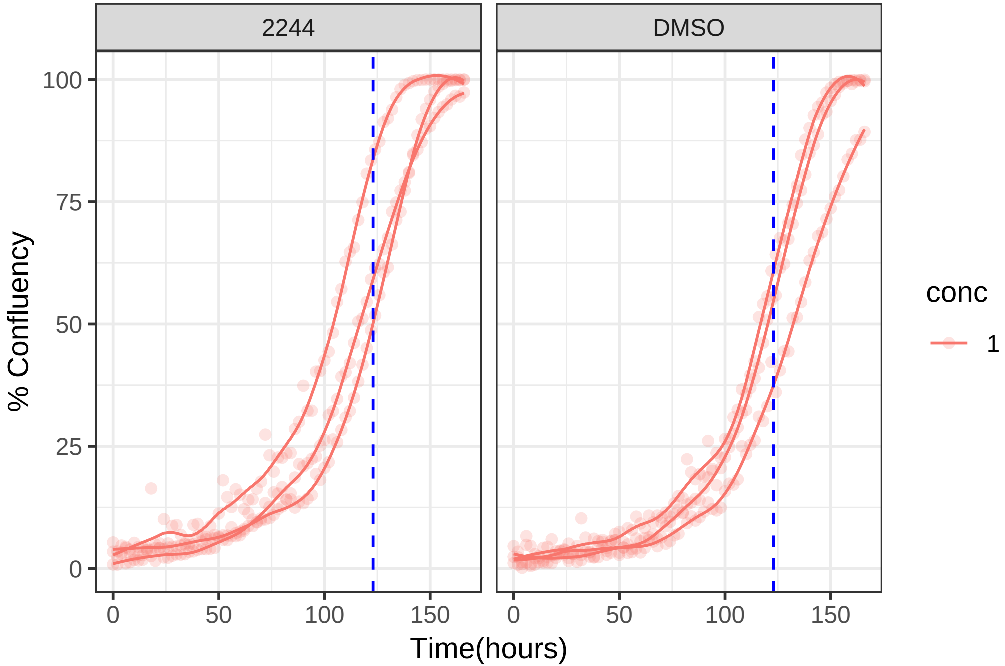

1 uM 2244 RPE1 Ts12 Ts5

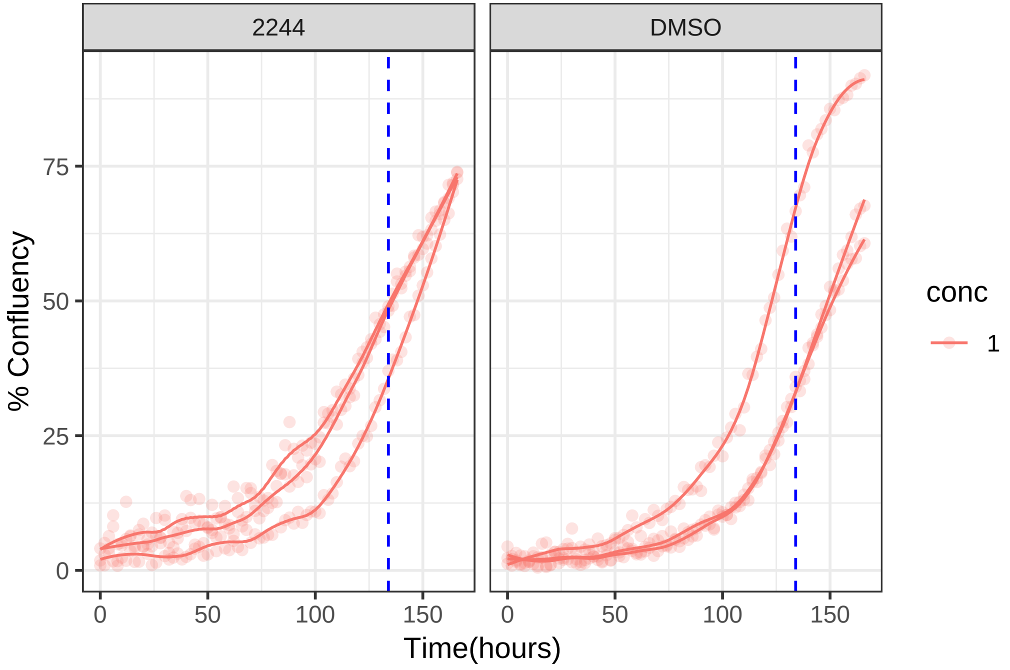

1 uM 2249 RPE1 hTert

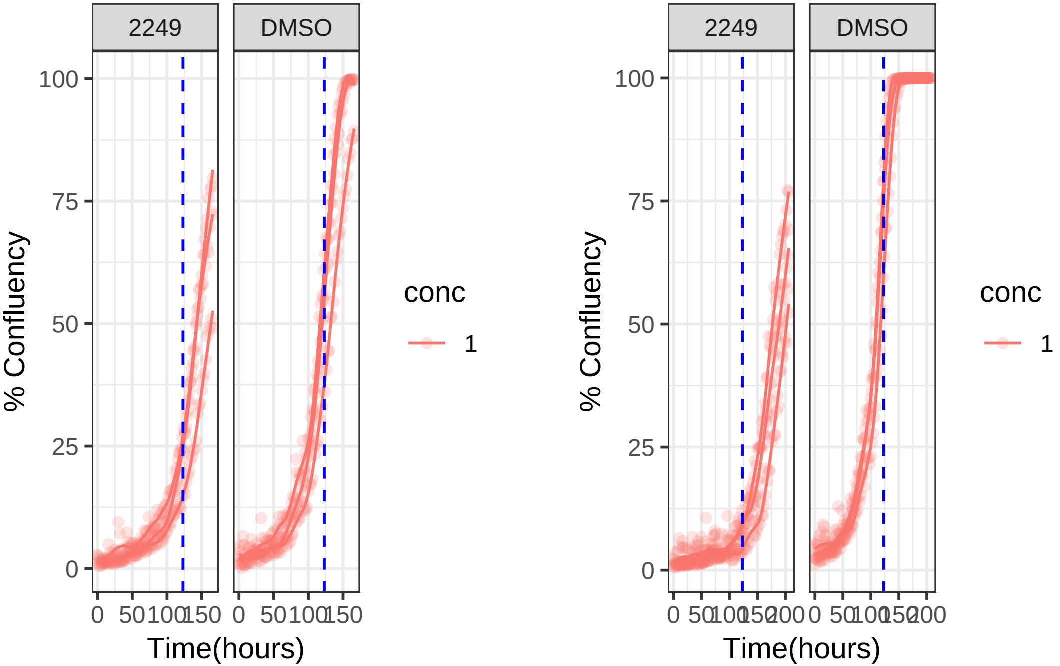

1 uM 2249 RPE1 Ts12 Ts5

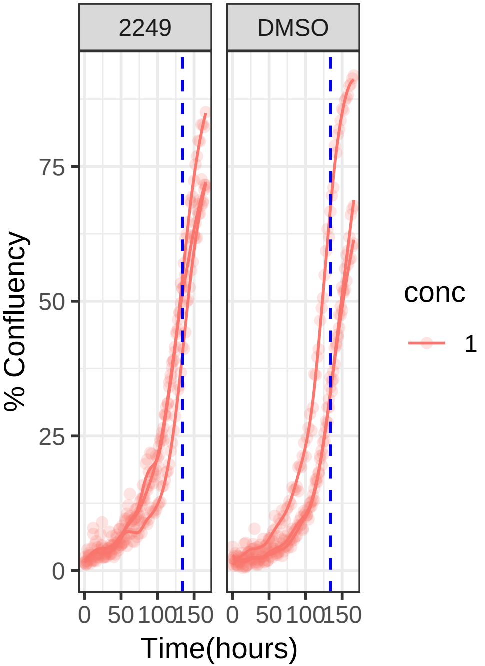

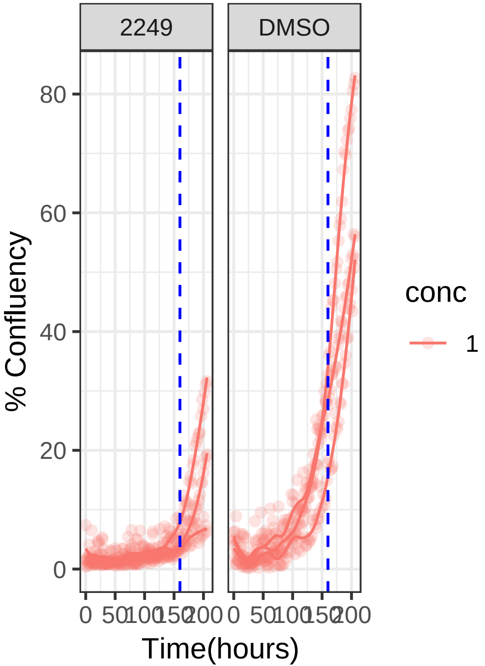

0.1 uM 2250 RPE1 hTert

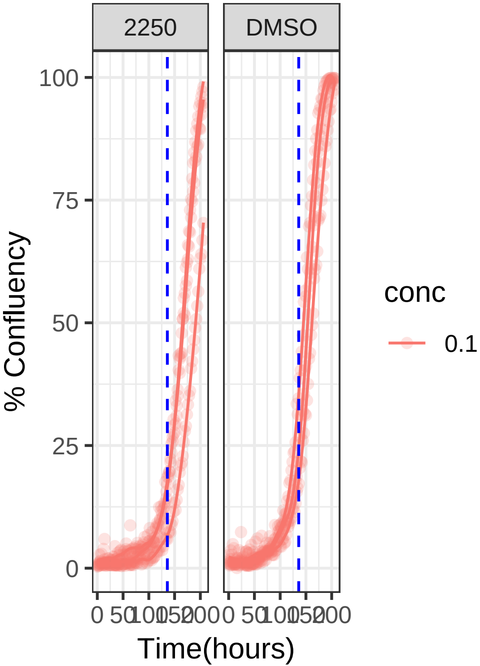

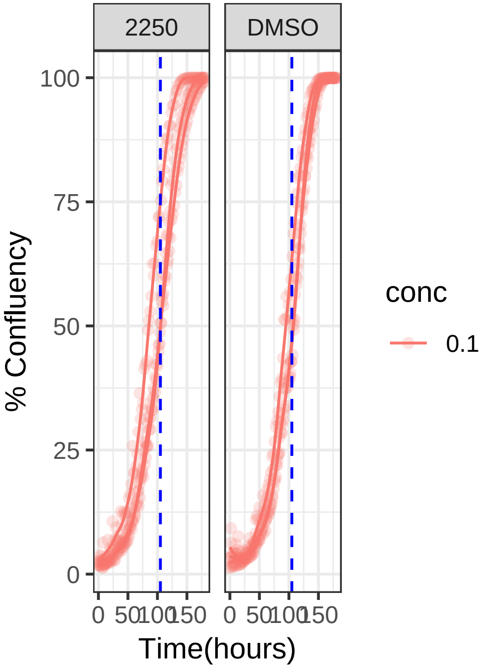

0.1 uM 2250 RPE1 Ts12 Ts5

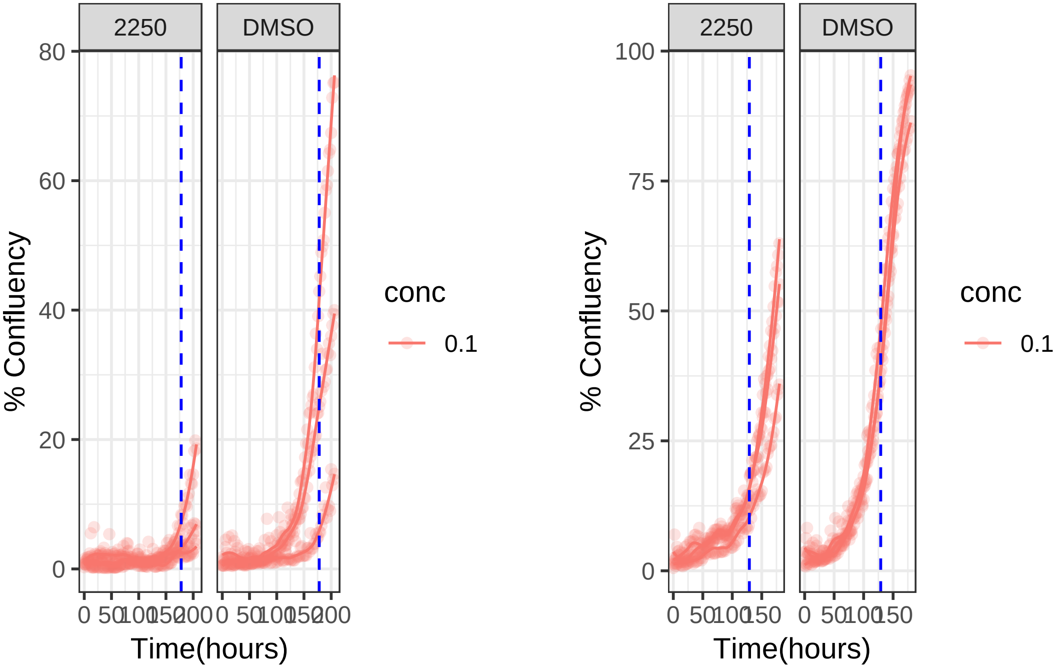

10 uM 2262 RPE1 hTert

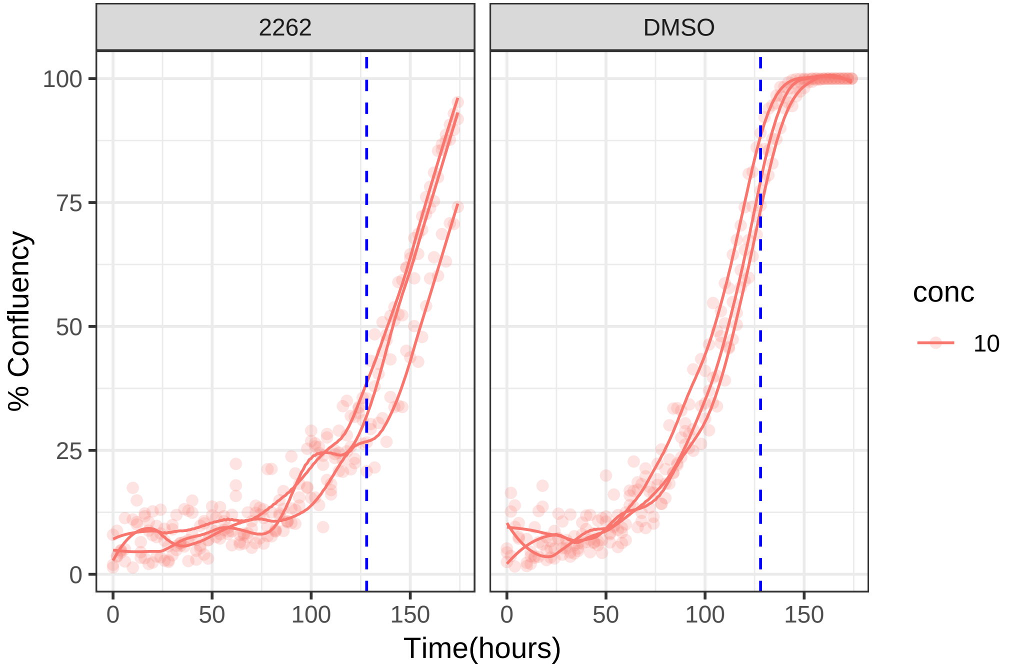

10 uM 2262 RPE1 Ts12 Ts5

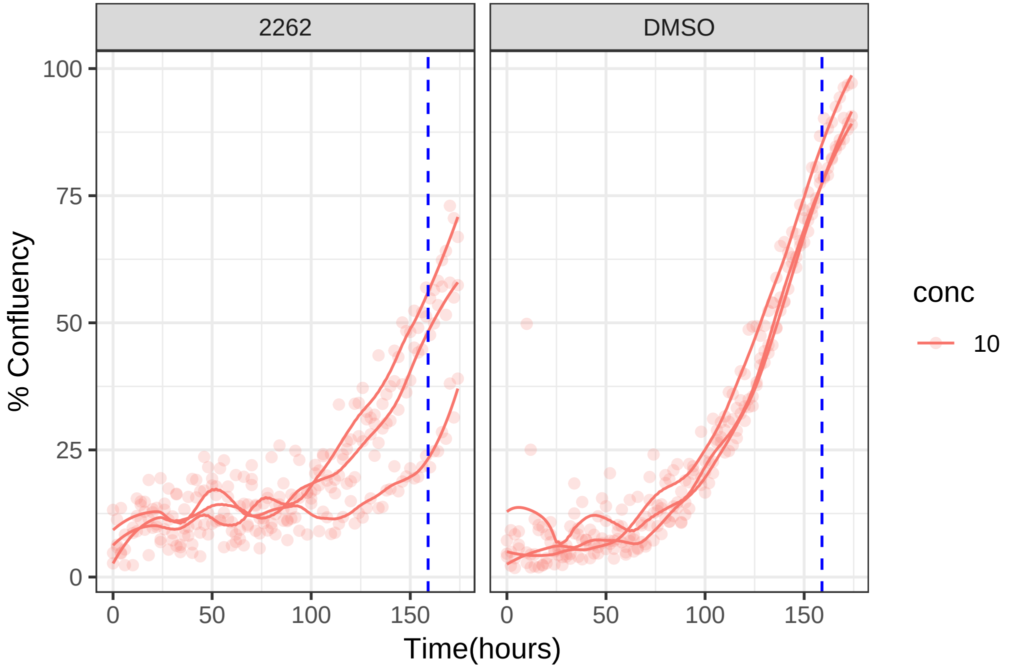

10 uM 2268 RPE1 hTert

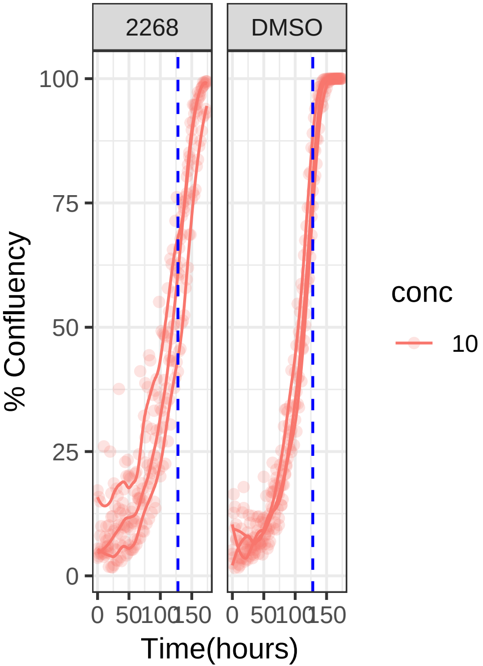

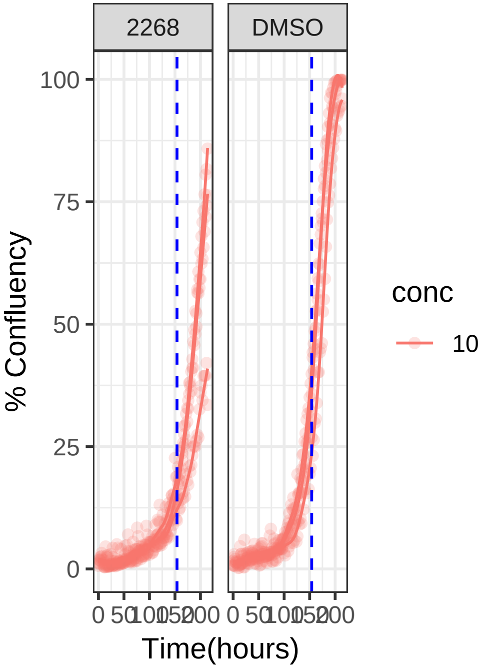

10 uM 2268 RPE1 Ts12 Ts5

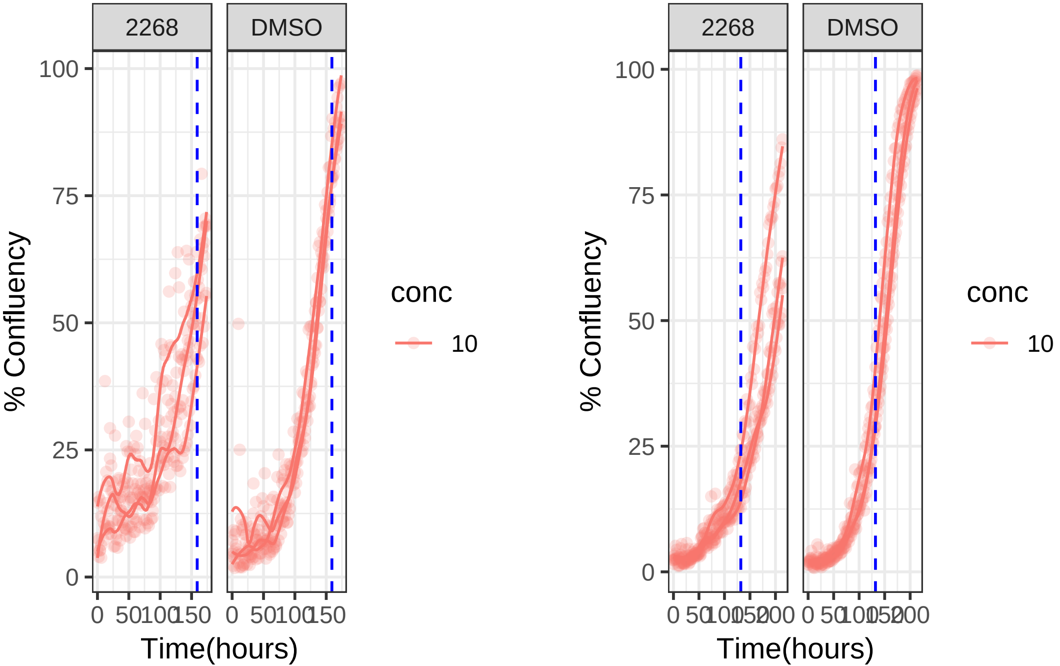

10 uM 2278 RPE1 hTert

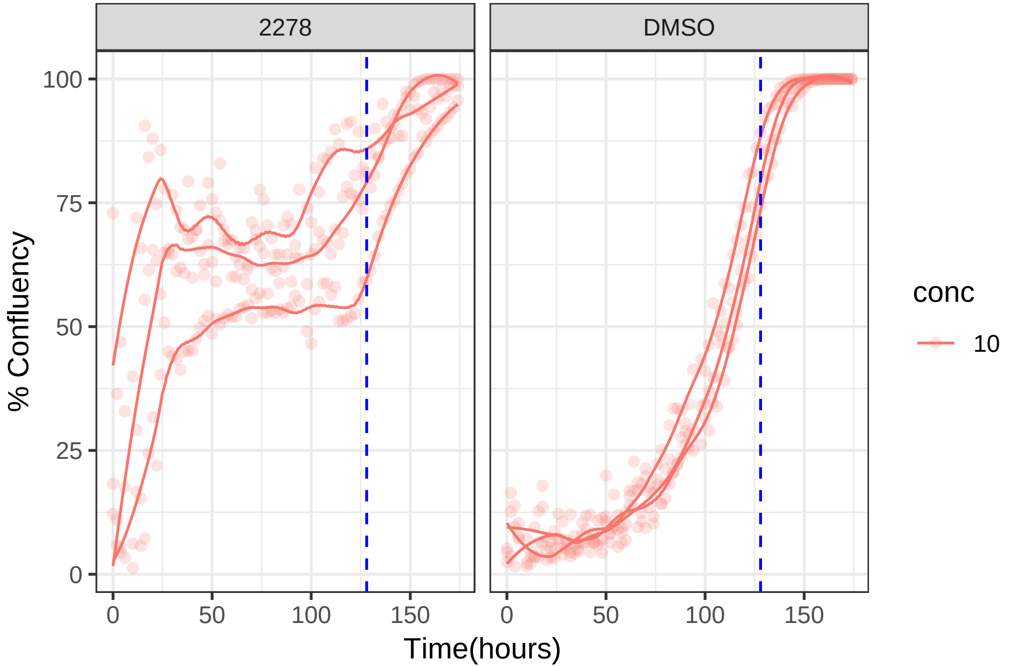

10 uM 2278 RPE1 Ts12 Ts5

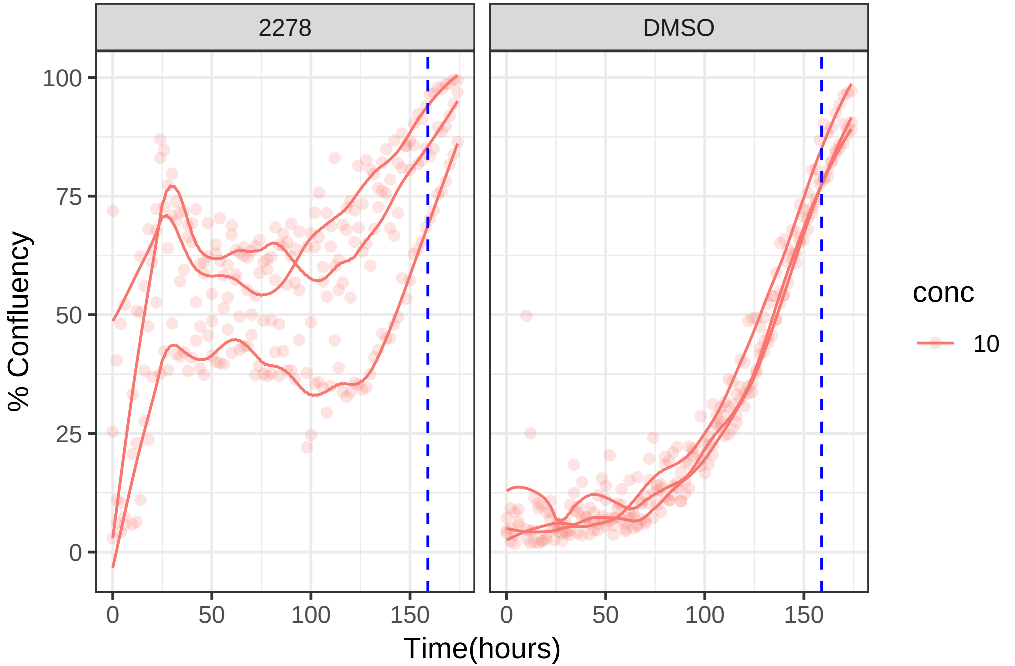

10 uM 2293 RPE1 hTert

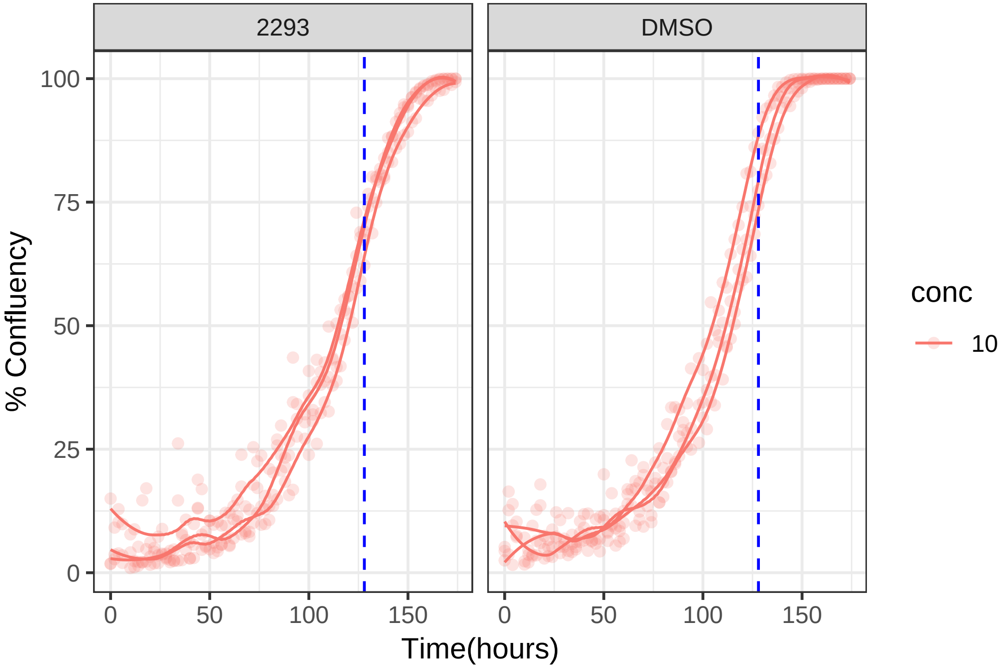

10 uM 2293 RPE1 Ts12 Ts5

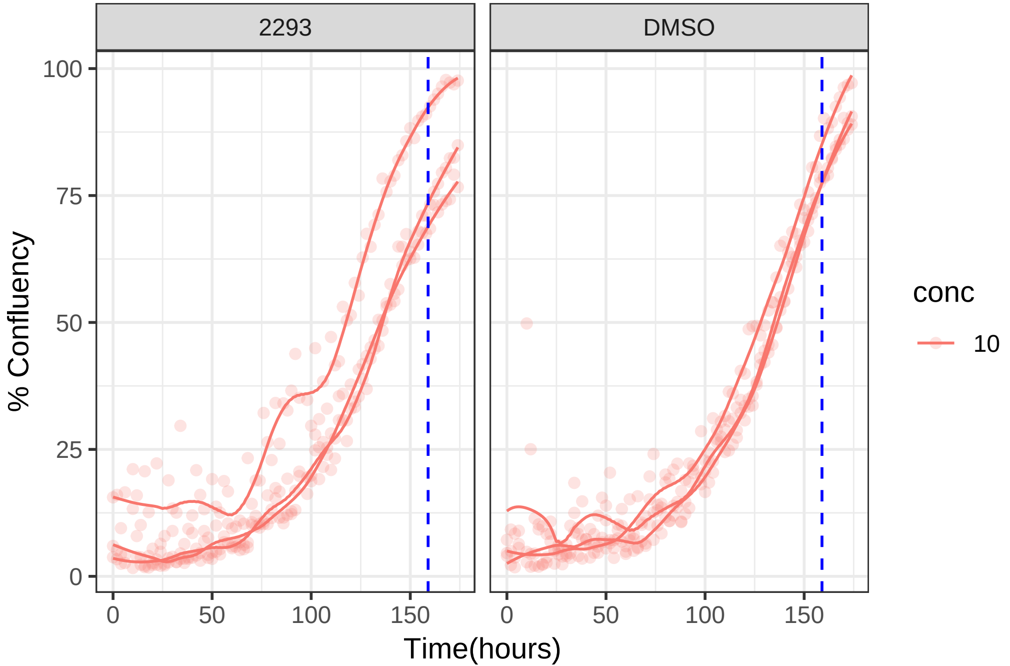

1 uM 2300 RPE1 hTert

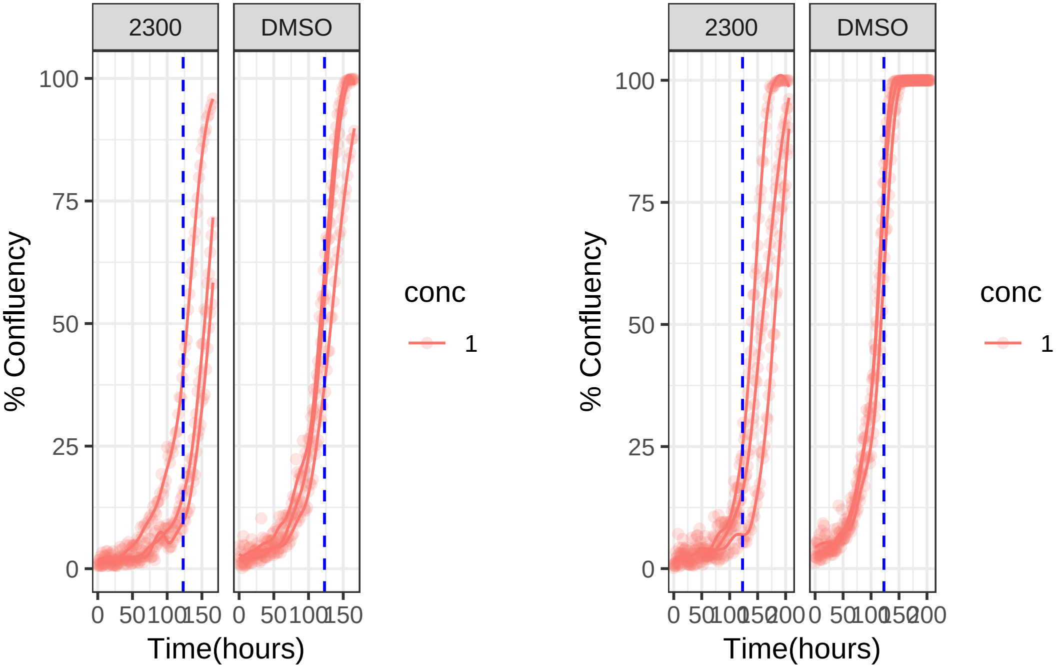

1 uM 2300 RPE1 Ts12 Ts5

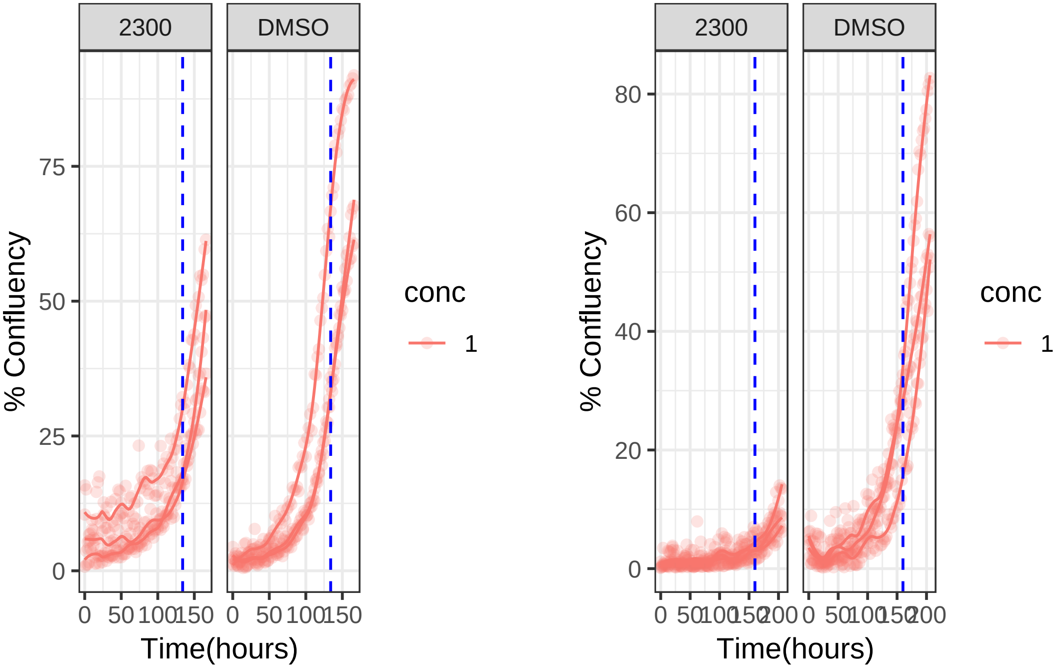

10 uM 2319 RPE1 hTert

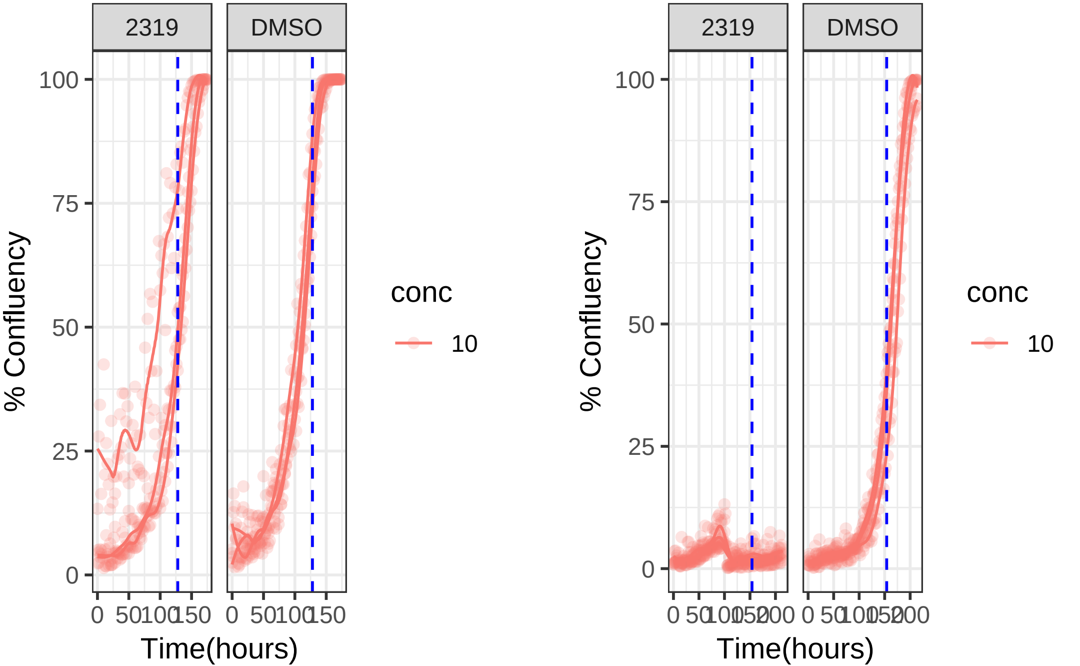

1 uM 2319 RPE1 hTert

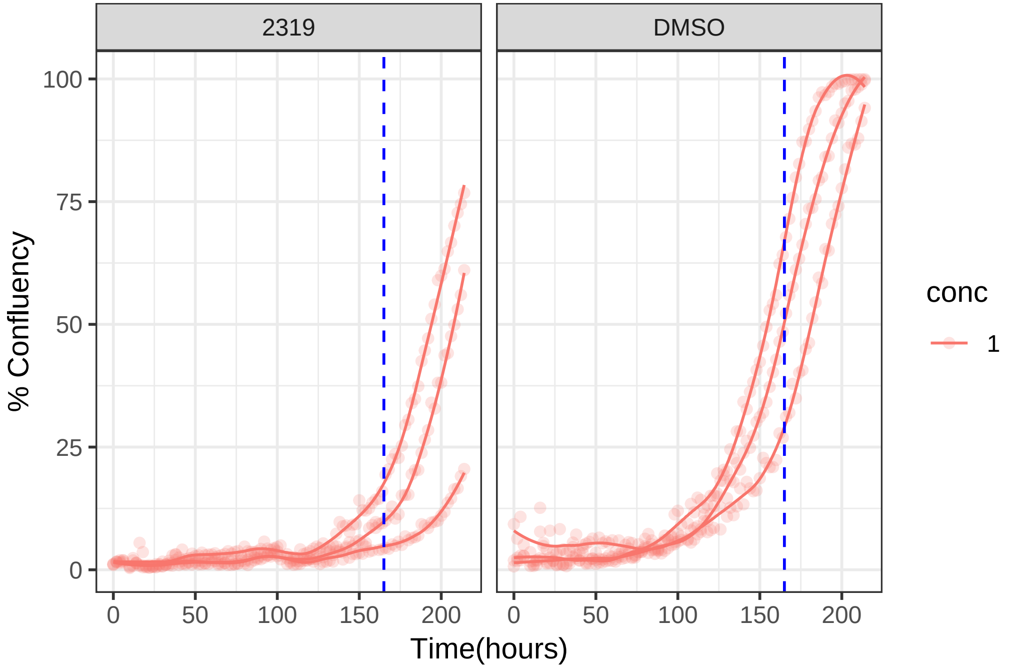

10 uM 2319 RPE1 Ts12 Ts5

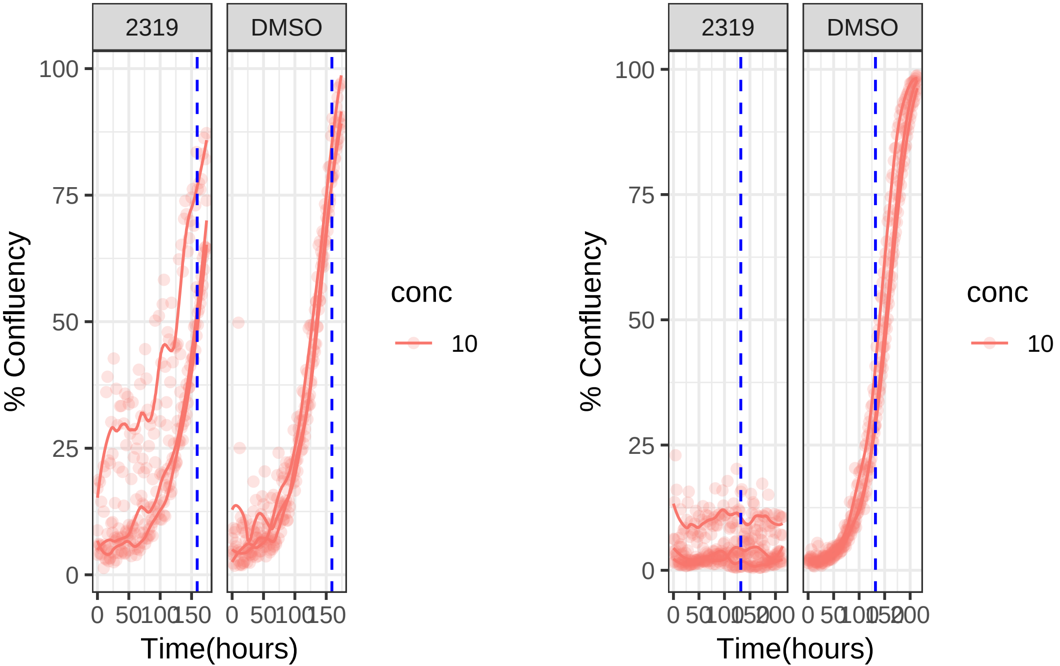

1 uM 2319 RPE1 Ts12 Ts5

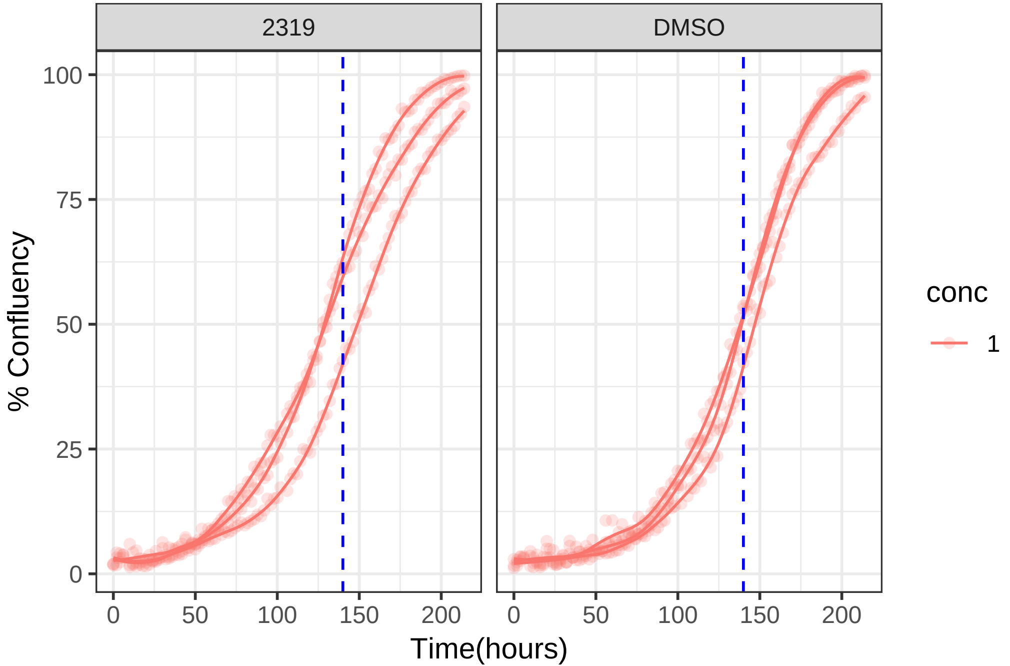

1 uM 2323 RPE1 hTert

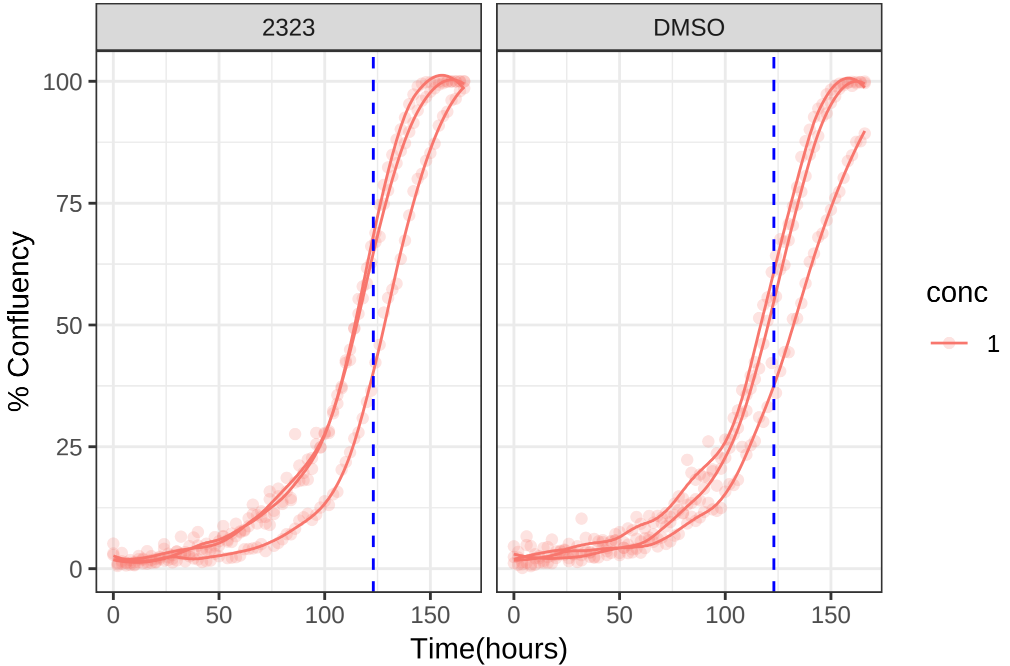

1 uM 2323 RPE1 Ts12 Ts5

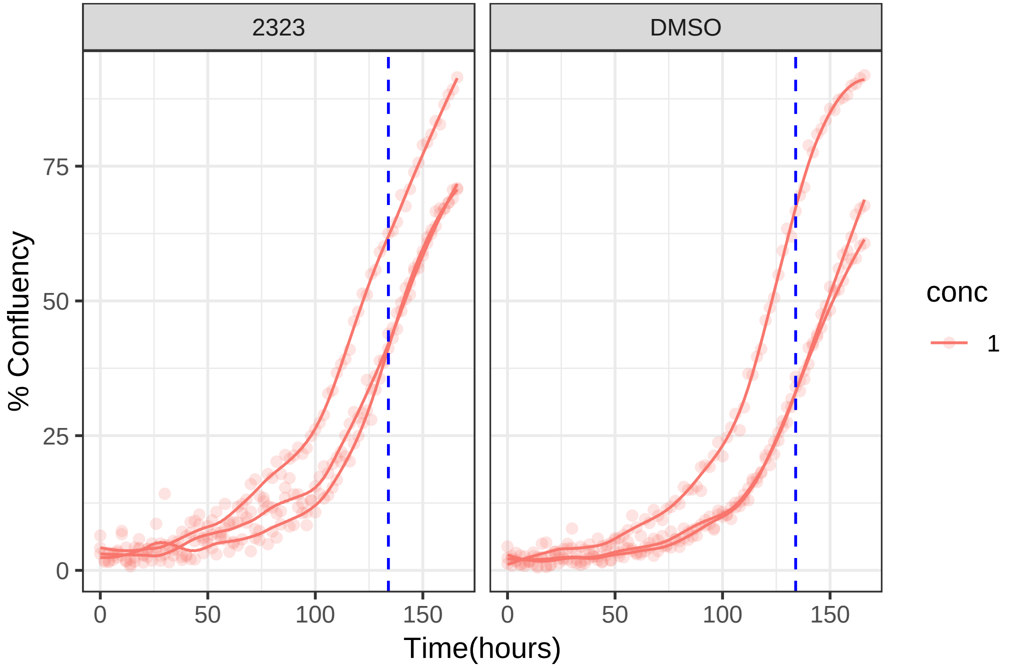

10 uM 2325 RPE1 hTert

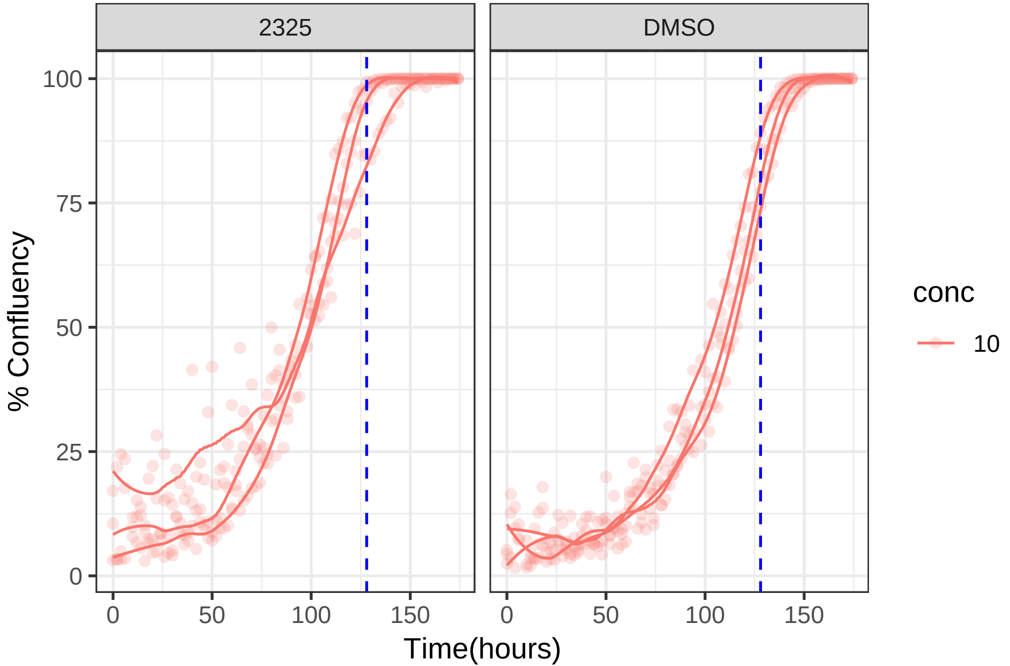

10 uM 2325 RPE1 Ts12 Ts5

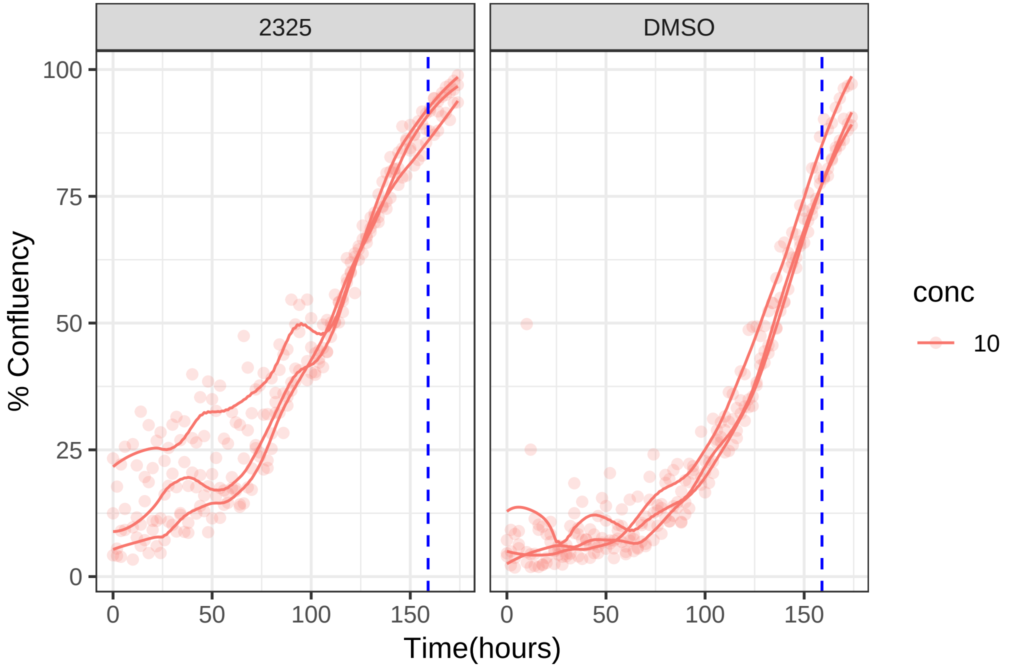

10 uM 2326 RPE1 hTert

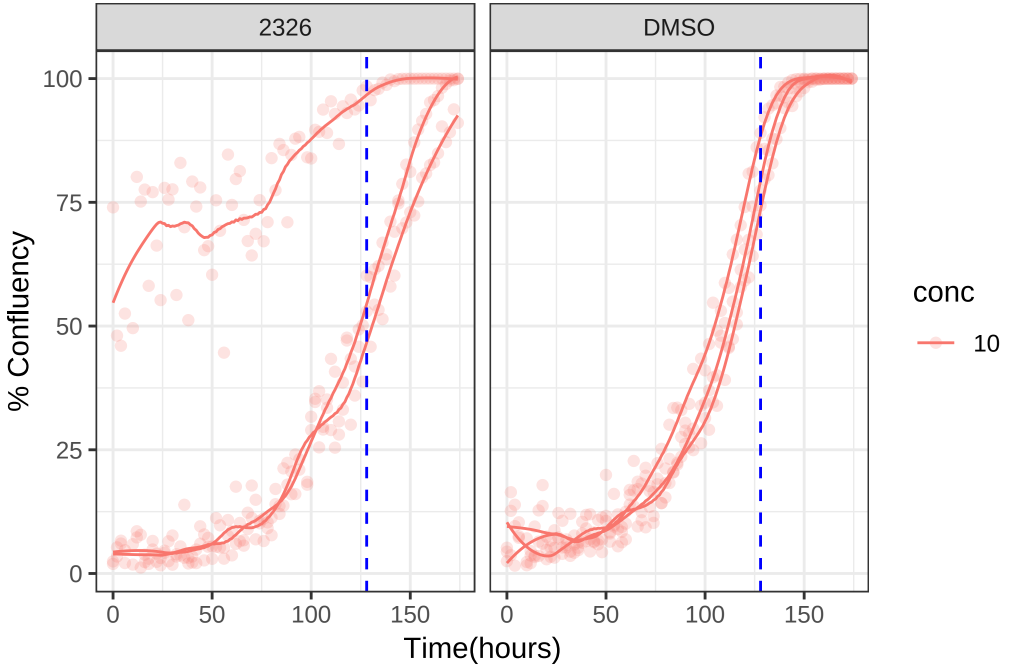

10 uM 2326 RPE1 Ts12 Ts5

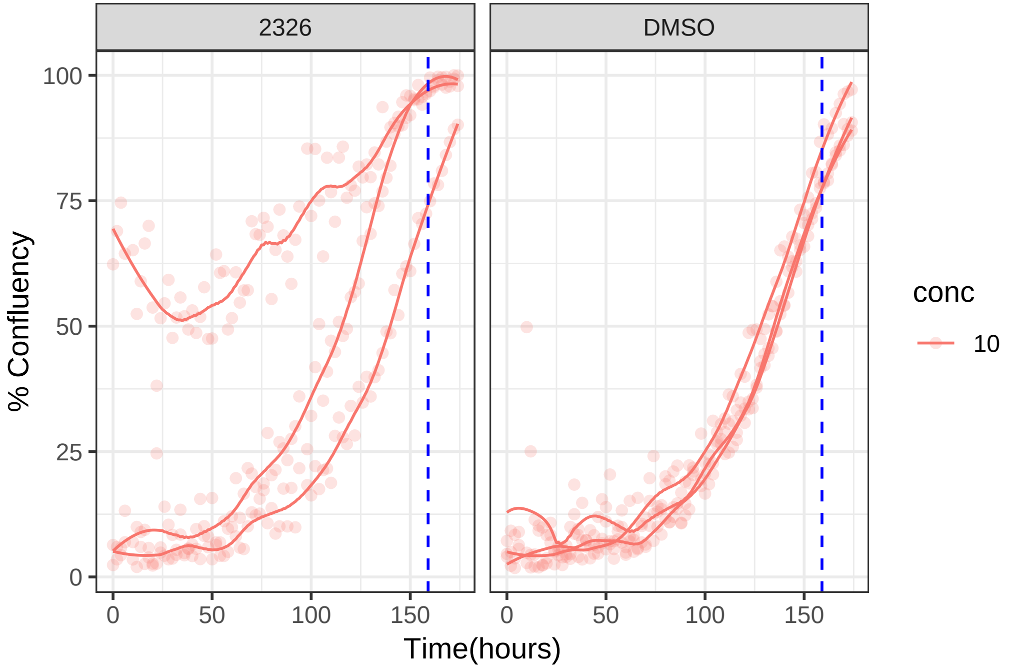

10 uM 2327 RPE1 hTert

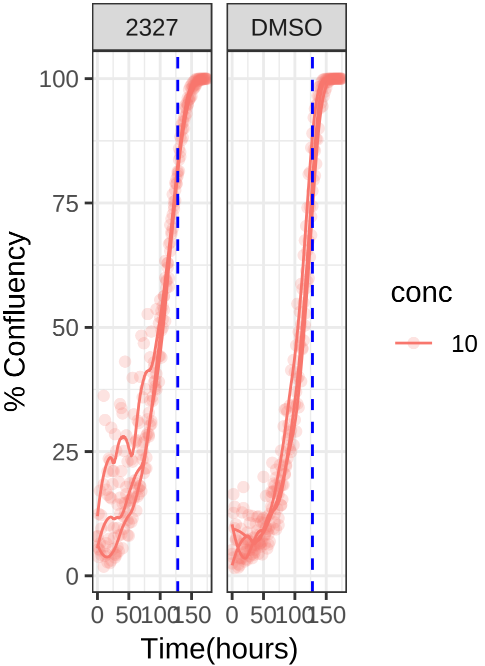

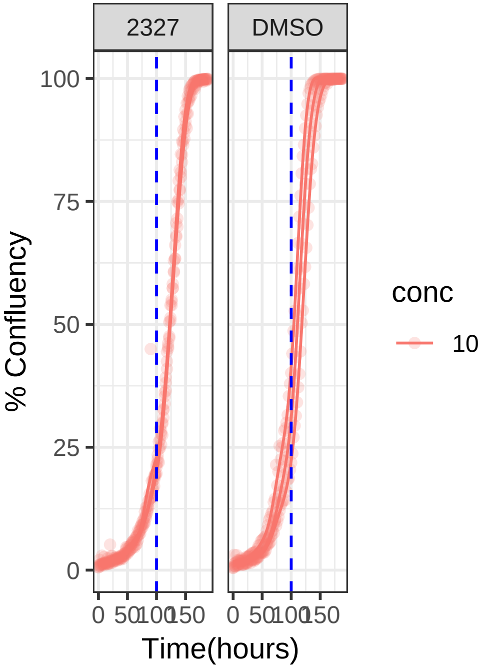

10 uM 2327 RPE1 Ts12 Ts5

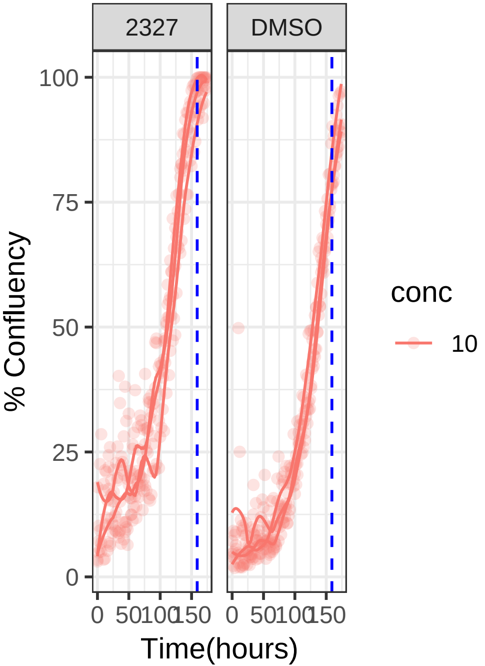

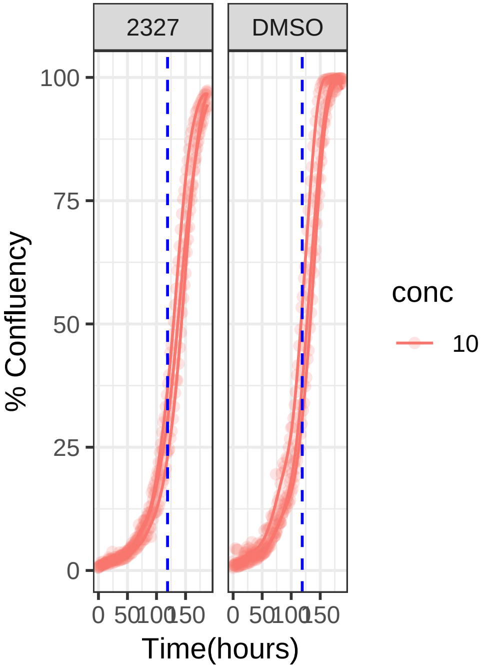

10 uM 2339 RPE1 hTert

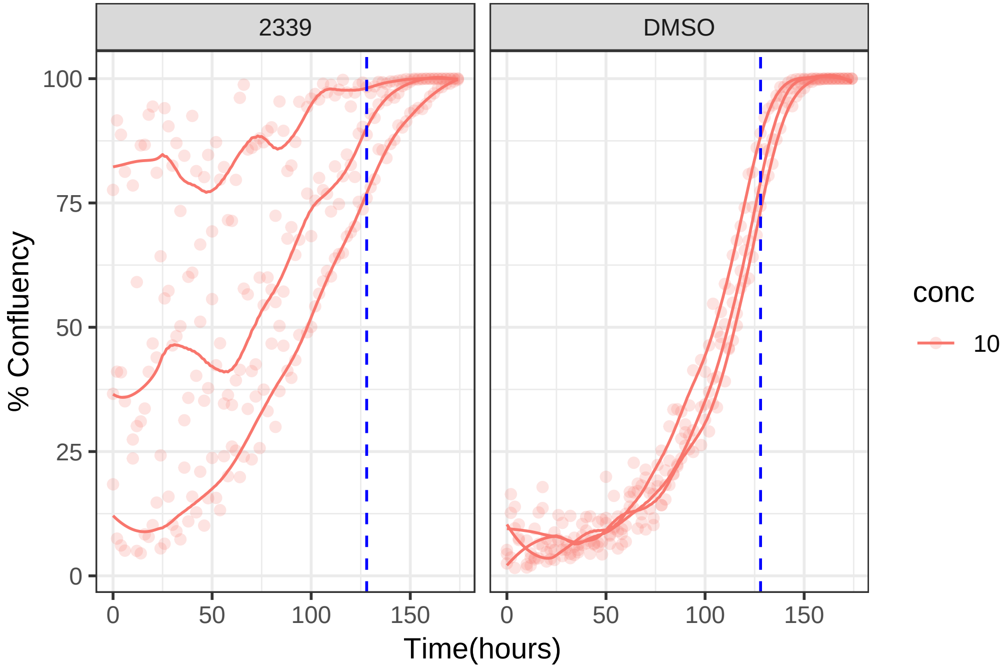

10 uM 2339 RPE1 Ts12 Ts5

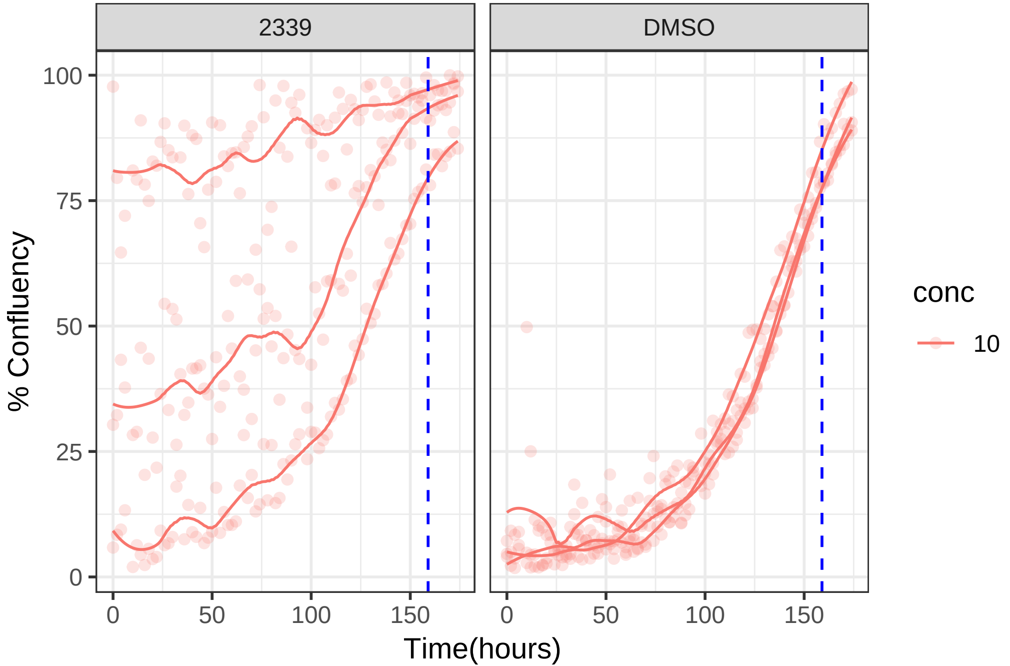

0.1 uM 2347 RPE1 hTert

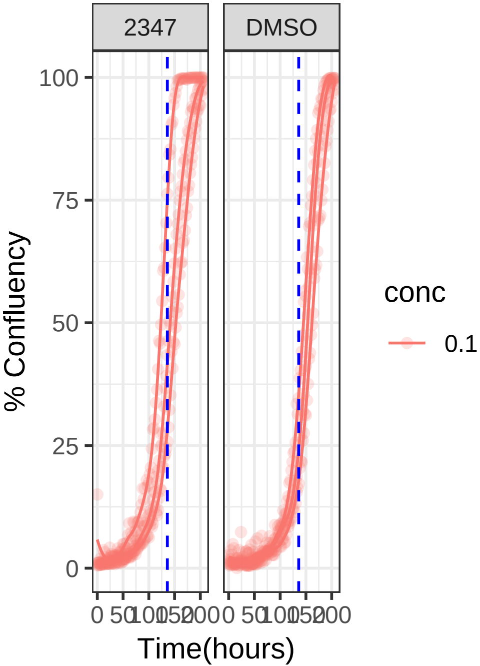

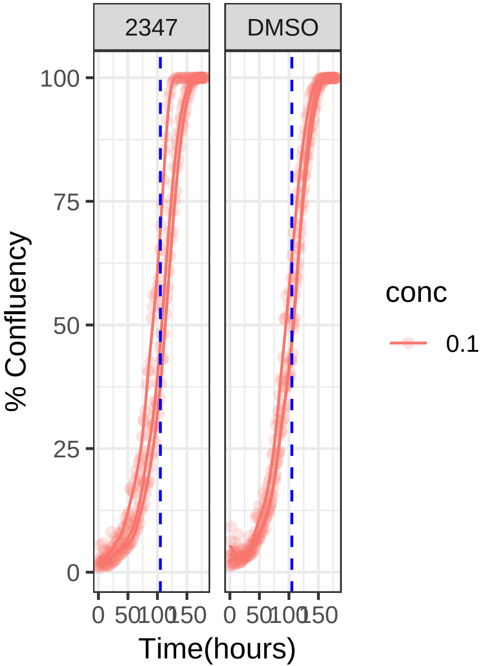

0.1 uM 2347 RPE1 Ts12 Ts5

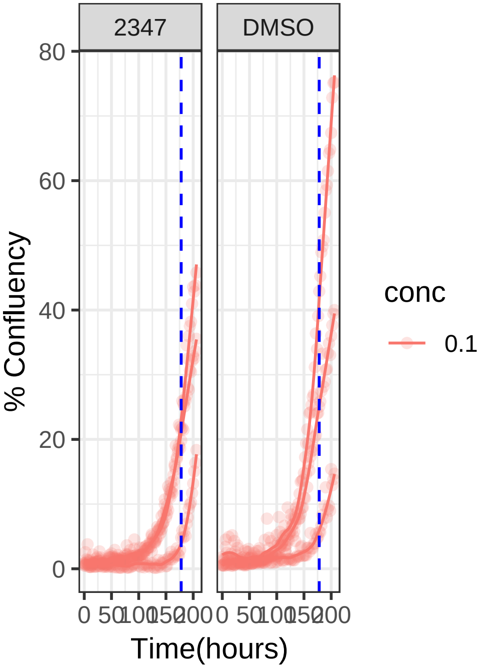

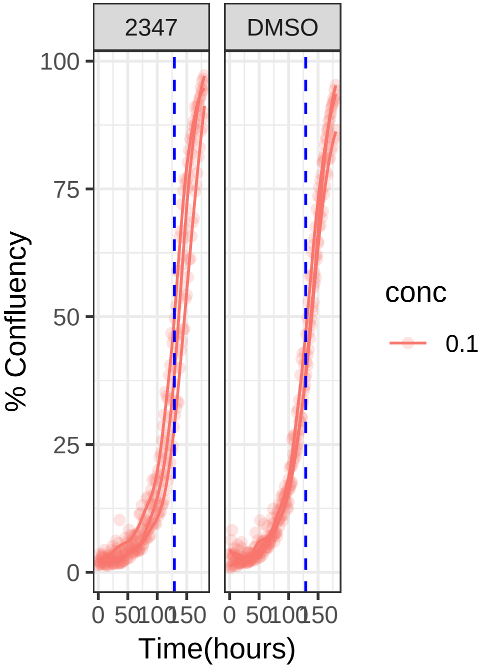

10 uM 2358 RPE1 hTert

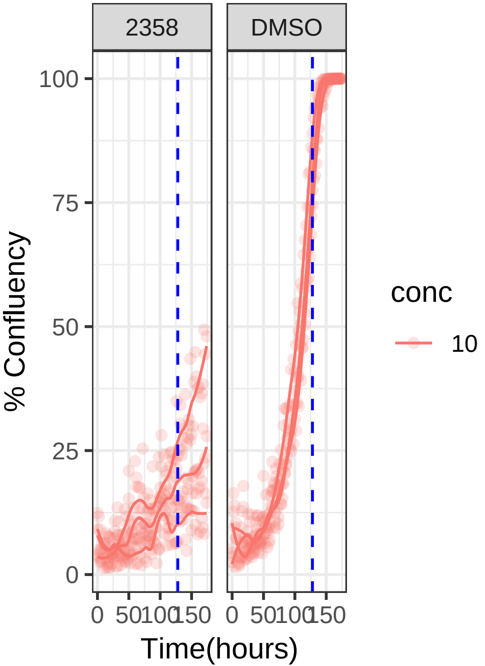

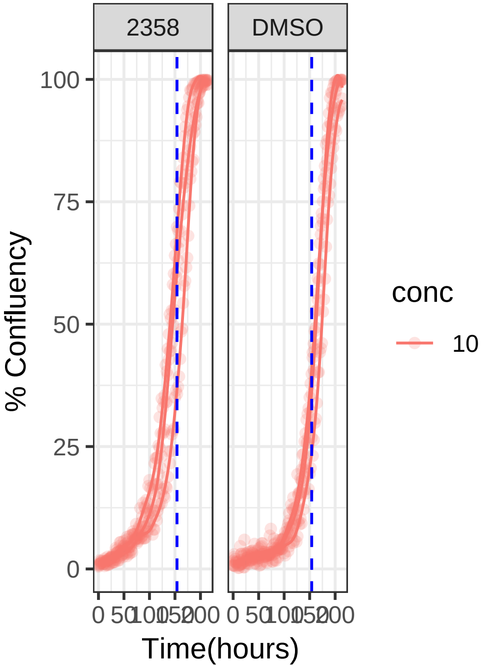

10 uM 2358 RPE1 Ts12 Ts5

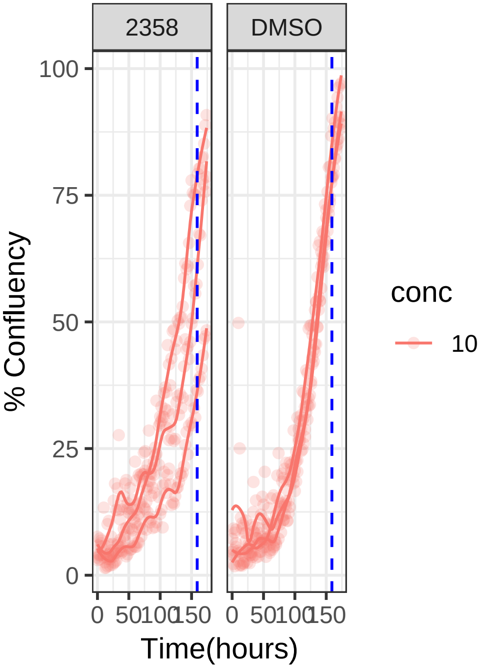

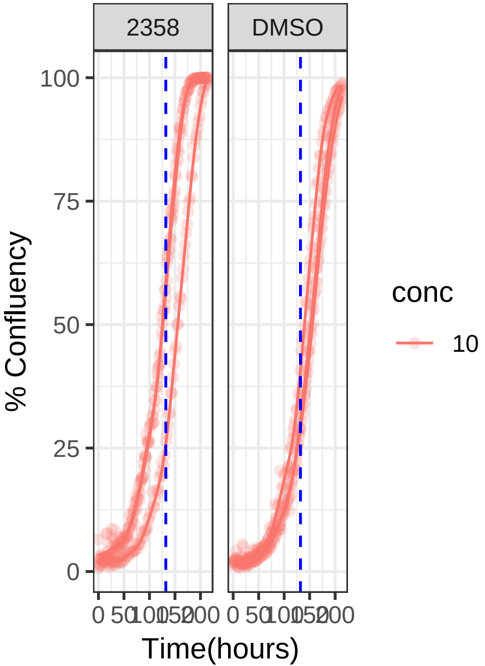

1 uM 2359 RPE1 hTert

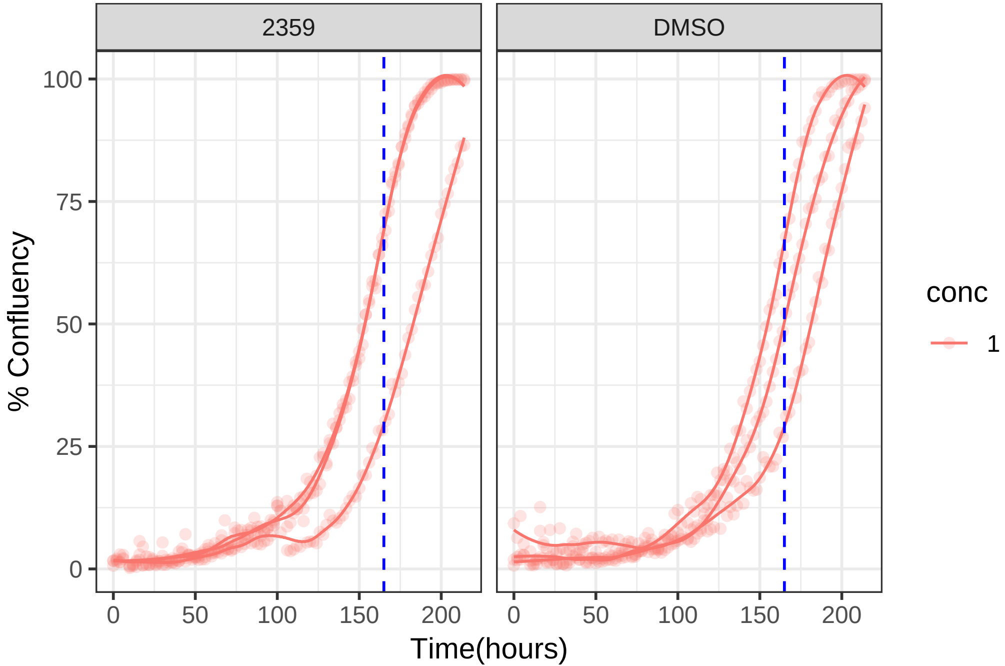

1 uM 2359 RPE1 Ts12 Ts5

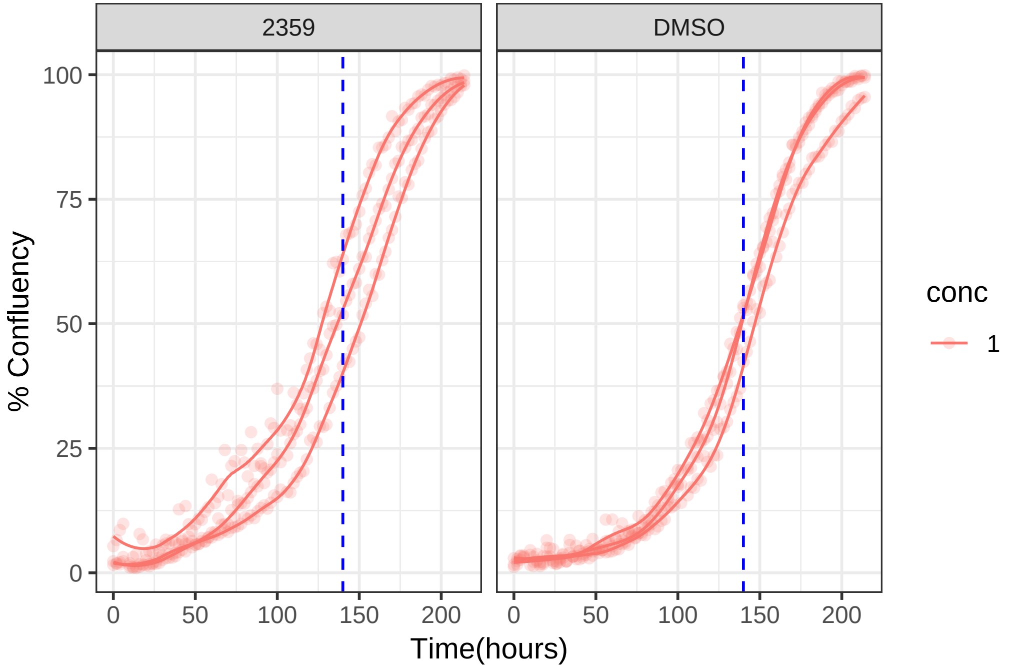

10 uM 2369 RPE1 hTert

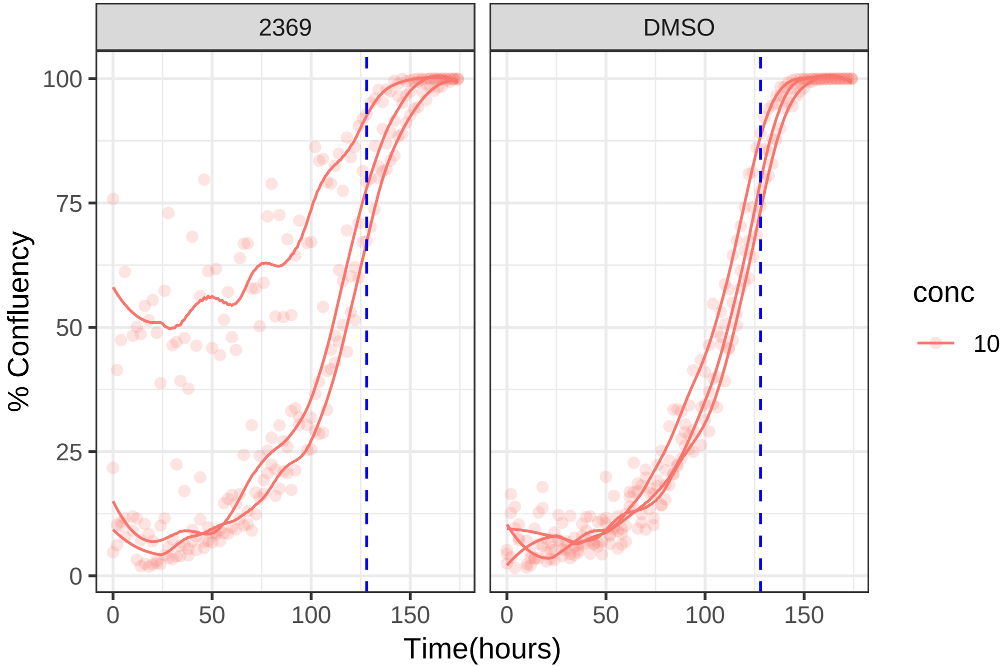

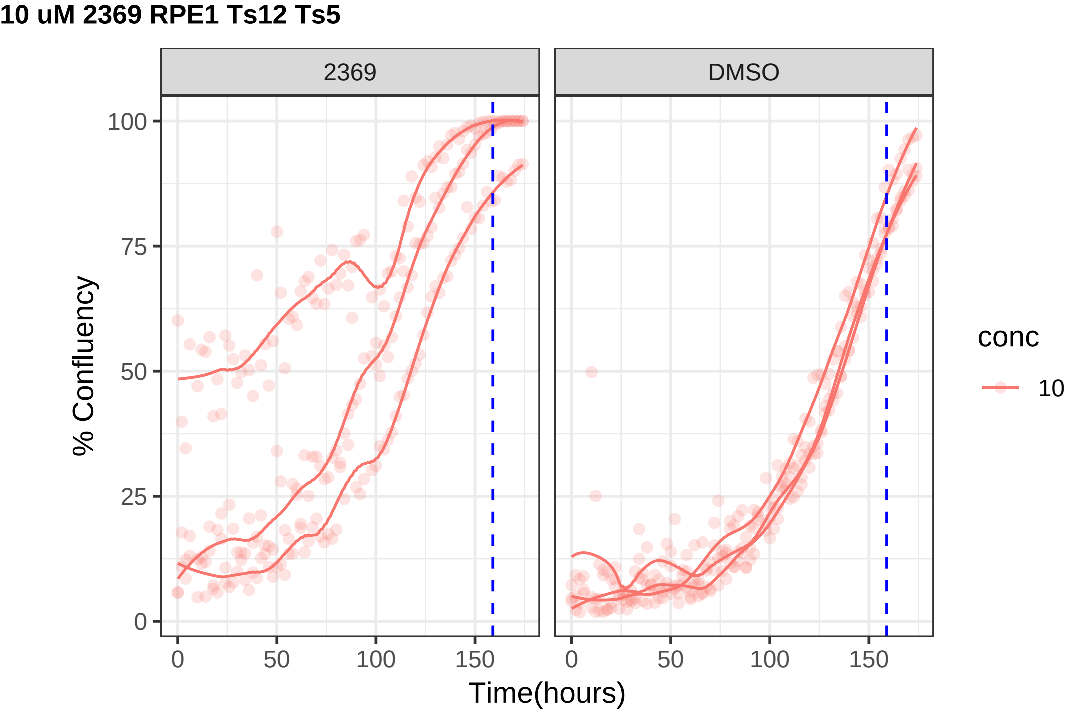

10 uM 2369 RPE1 Ts12 Ts5
2369
DMSO
% Confluency
Time(hours)
0
25
50
75
100
0
50
100
150
conc
10

10 uM 2375 RPE1 hTert

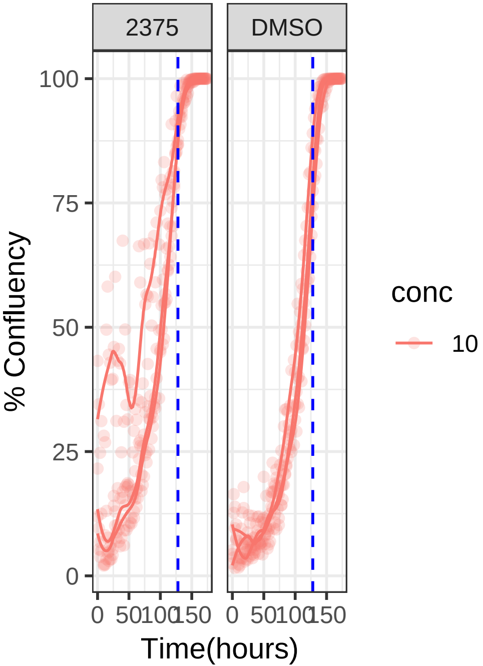

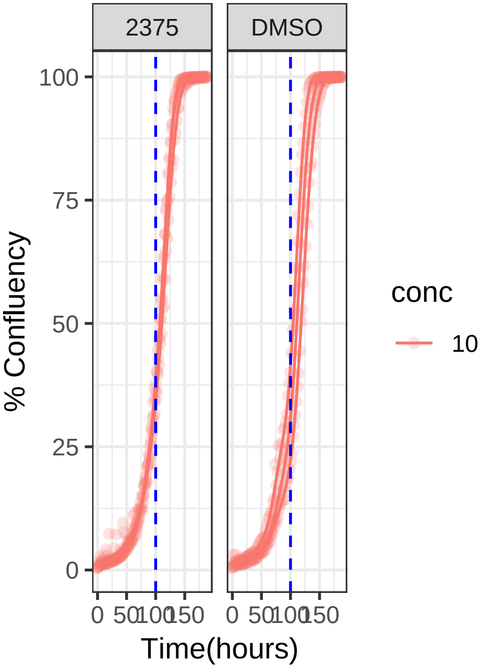

10 uM 2375 RPE1 Ts12 Ts5

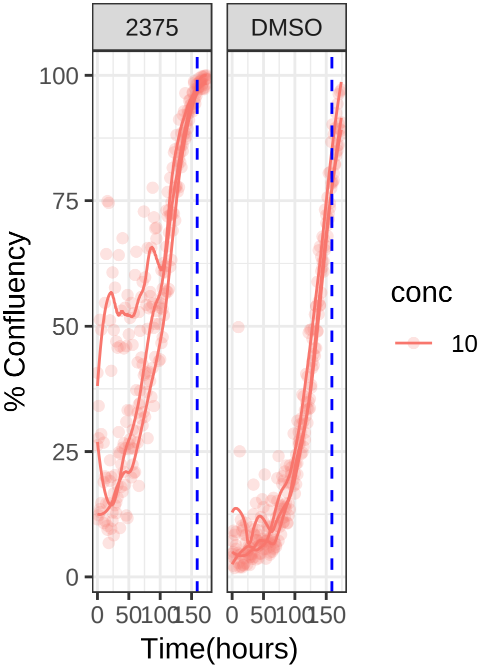

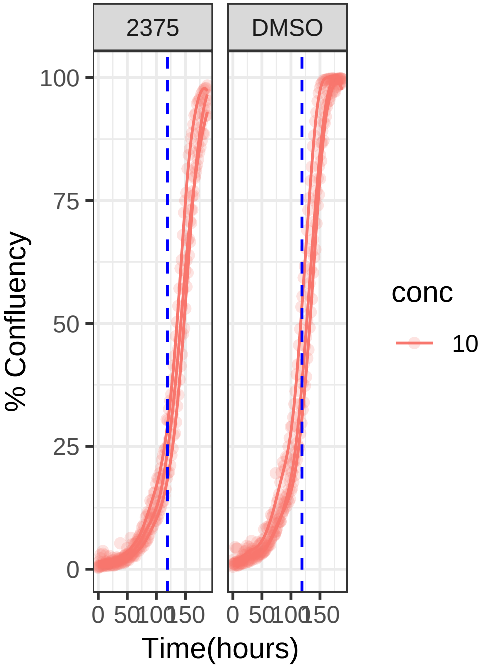

10 uM 2379 RPE1 hTert

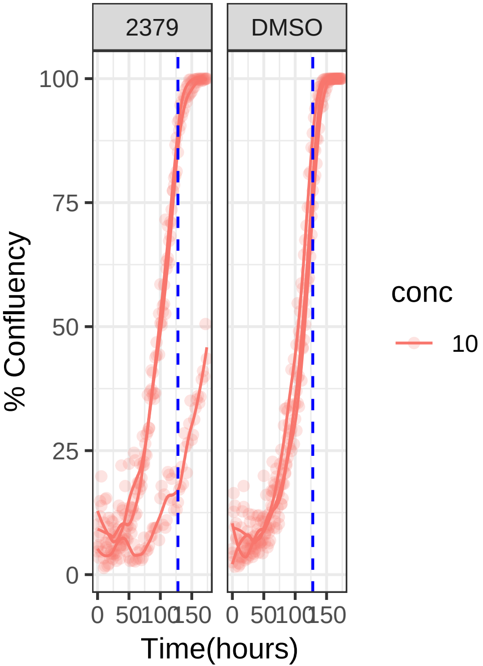

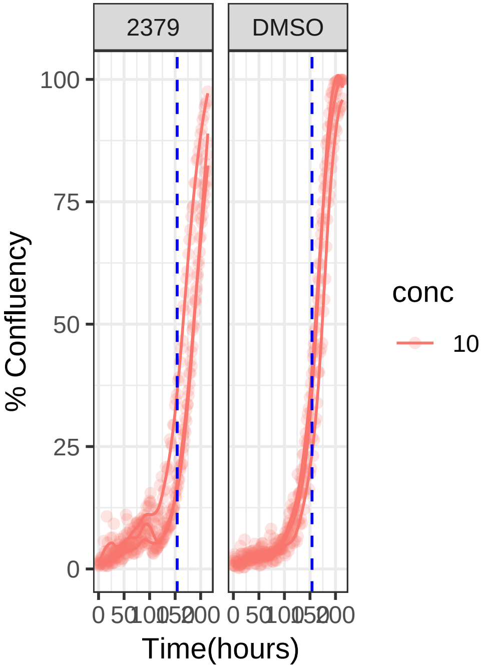

10 uM 2379 RPE1 Ts12 Ts5

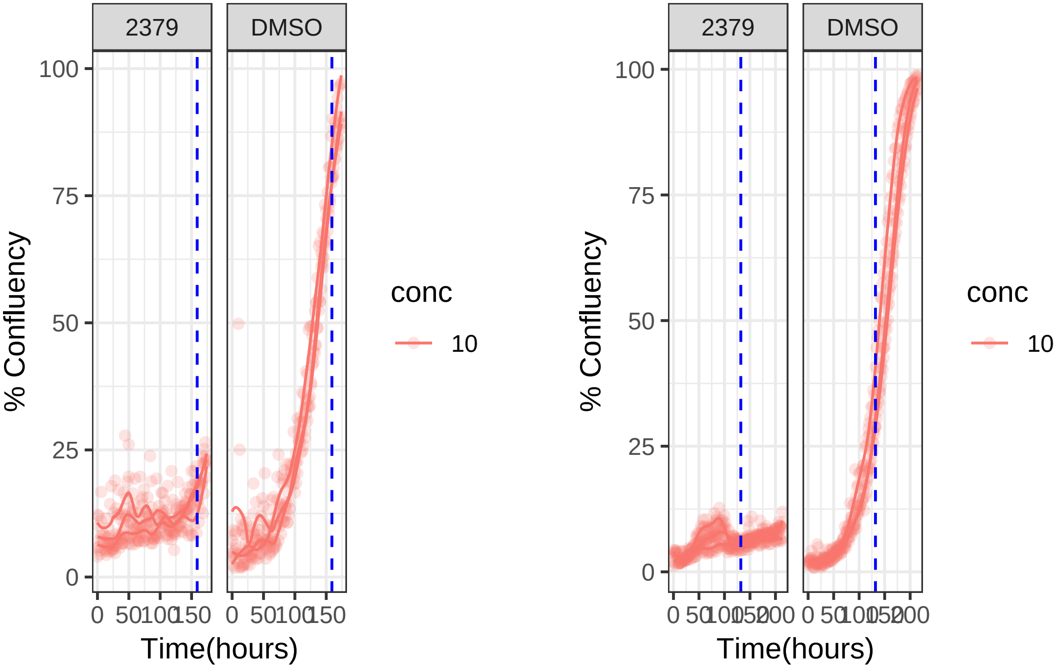

10 uM 2402 RPE1 hTert

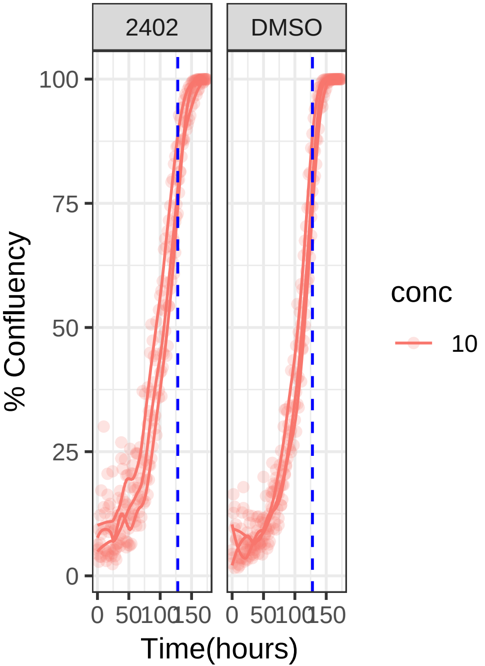

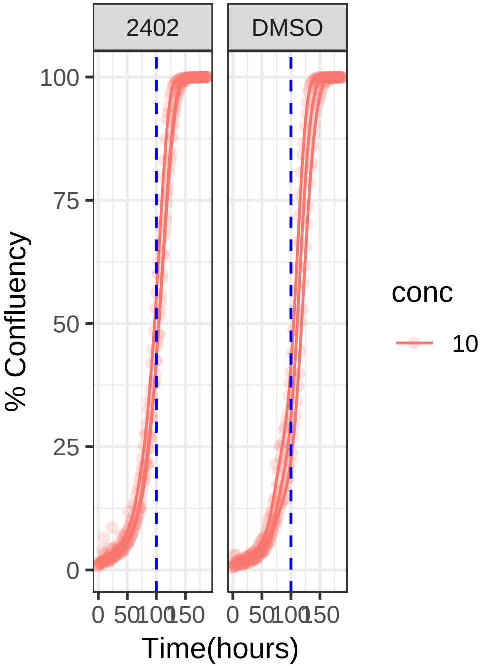

10 uM 2402 RPE1 Ts12 Ts5

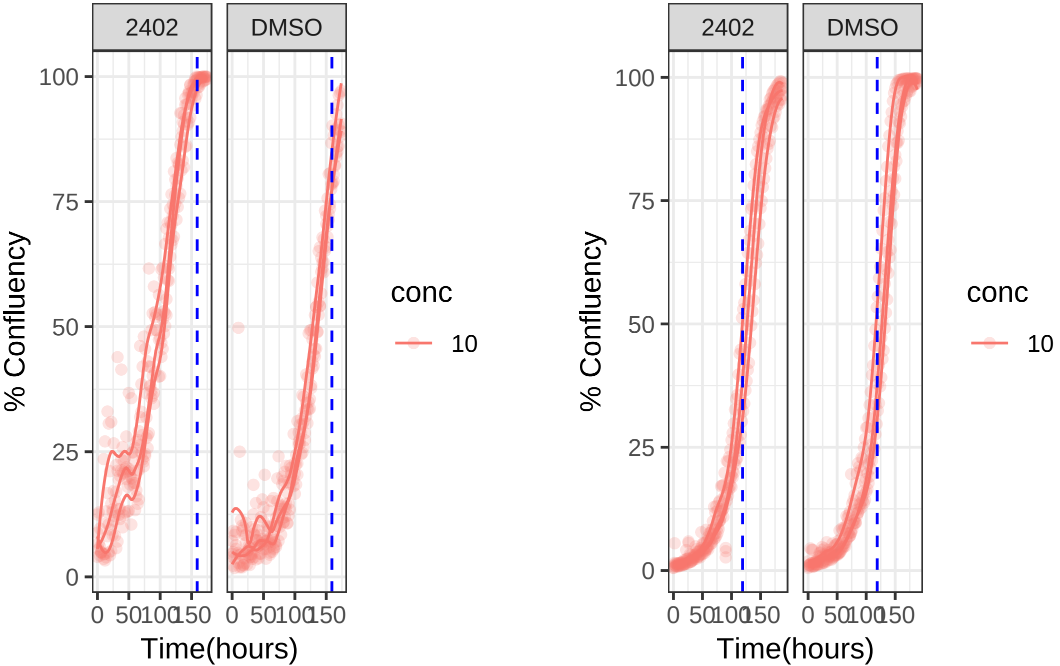

1 uM 2831 RPE1 hTert

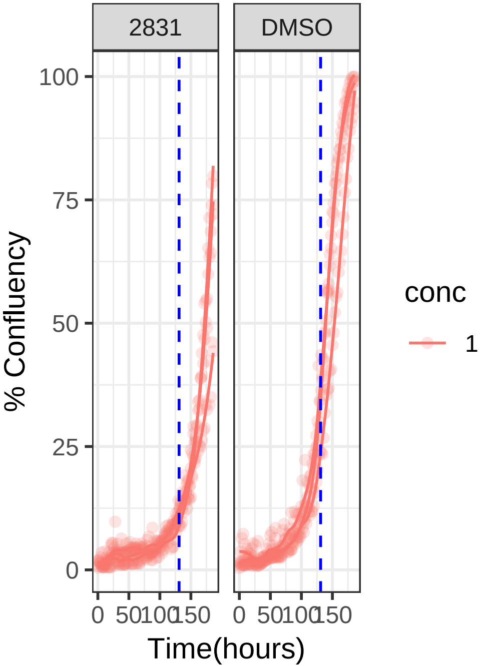

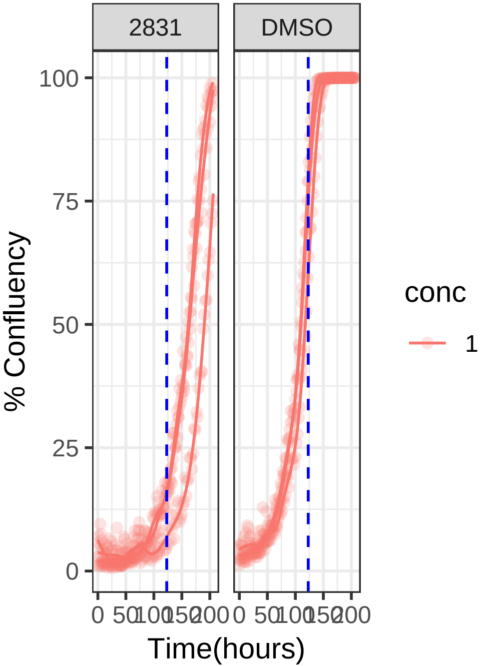

10 uM 2831 RPE1 hTert

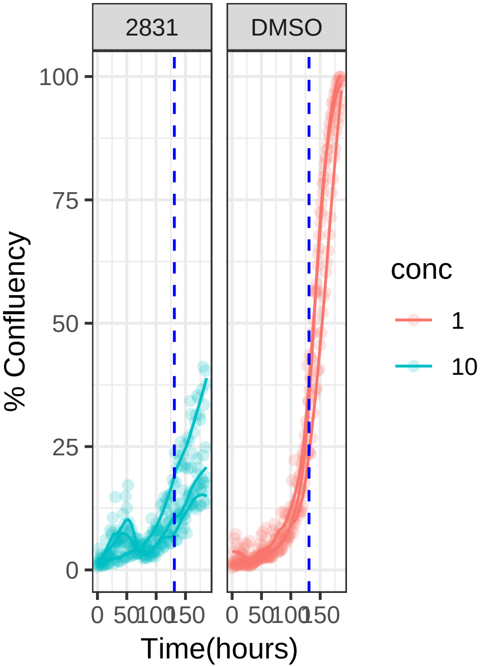

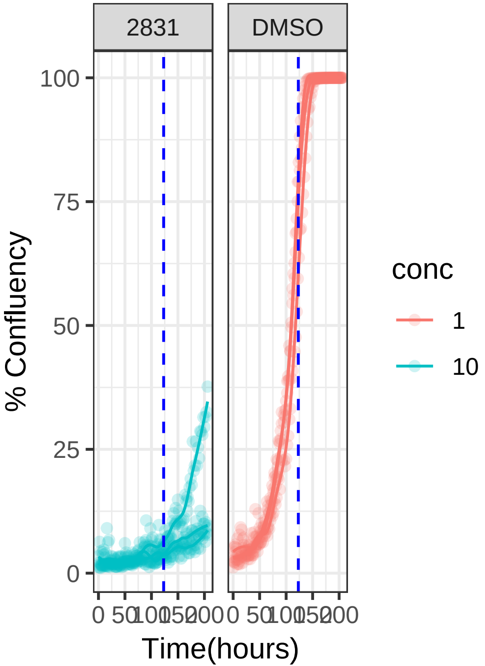

1 uM 2831 RPE1 Ts12 Ts5

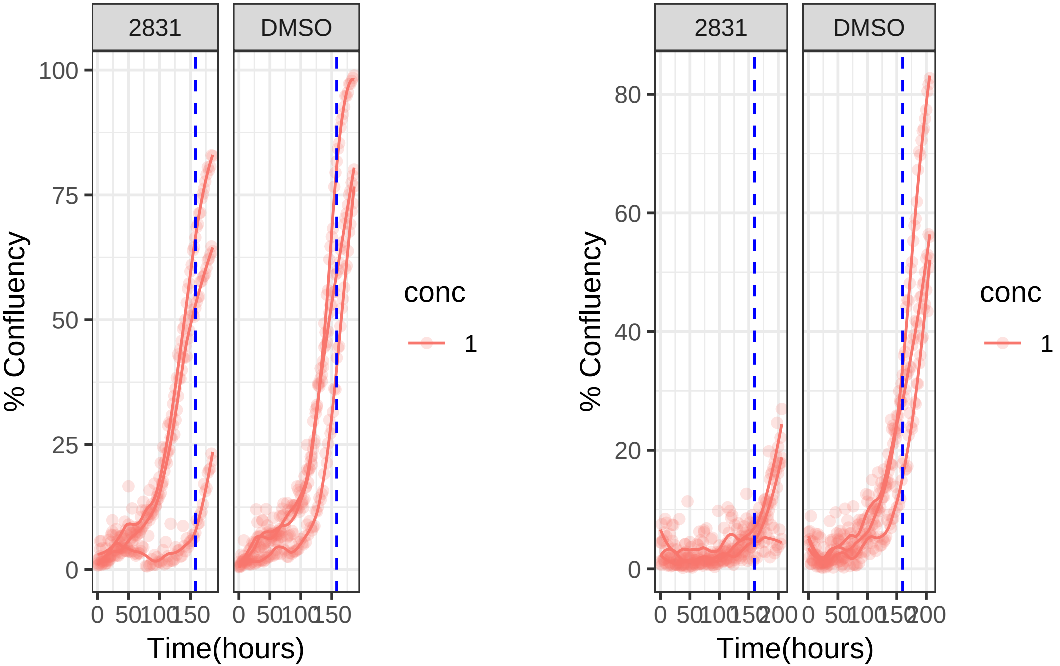

10 uM 2831 RPE1 Ts12 Ts5

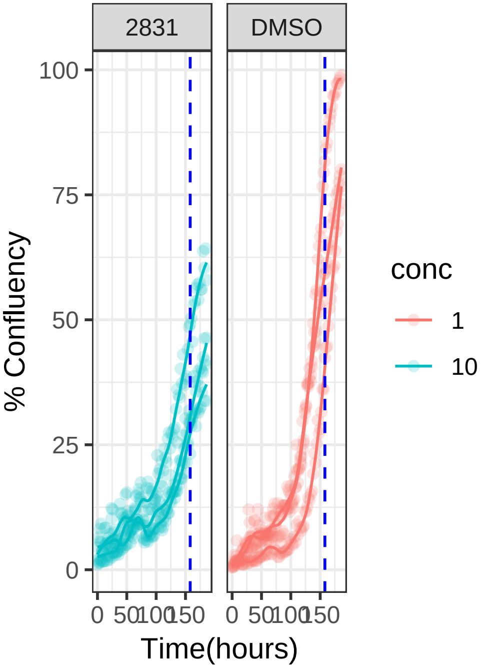

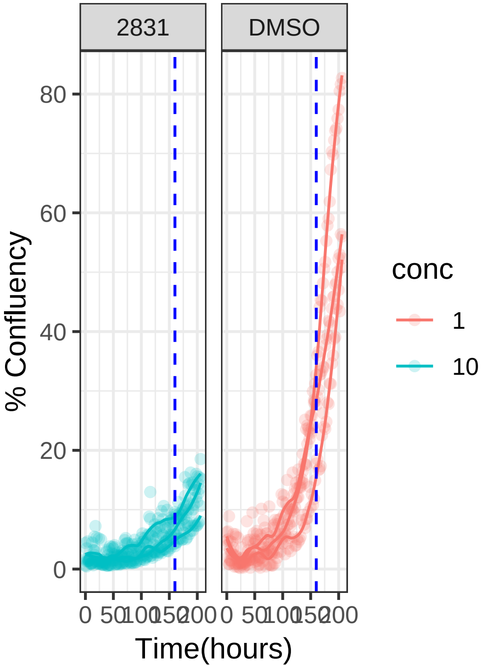

10 uM 2ndcontrol RPE1 hTert

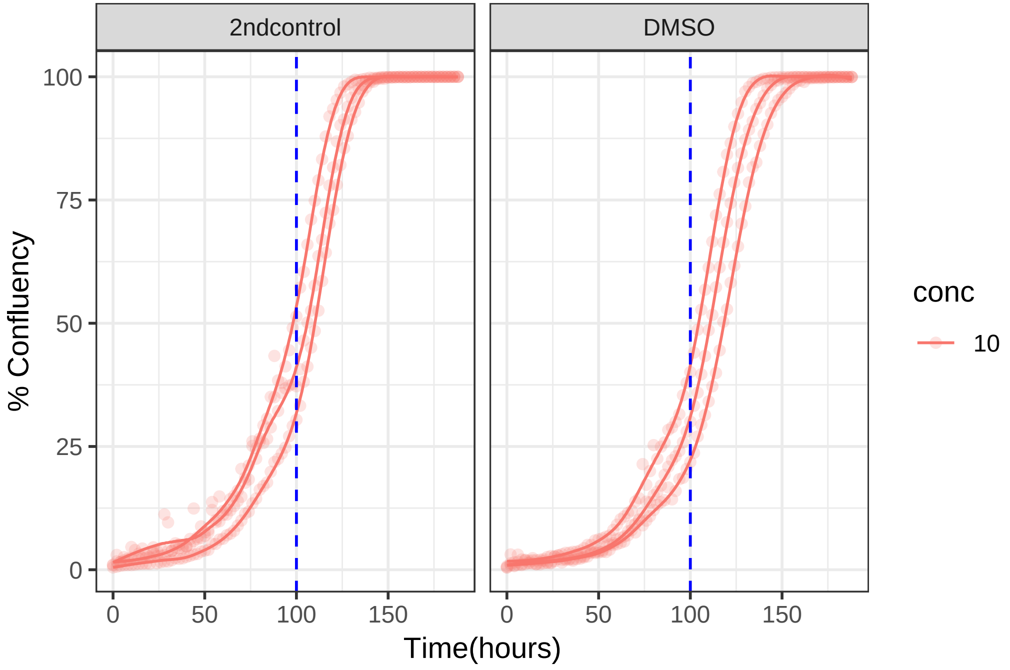

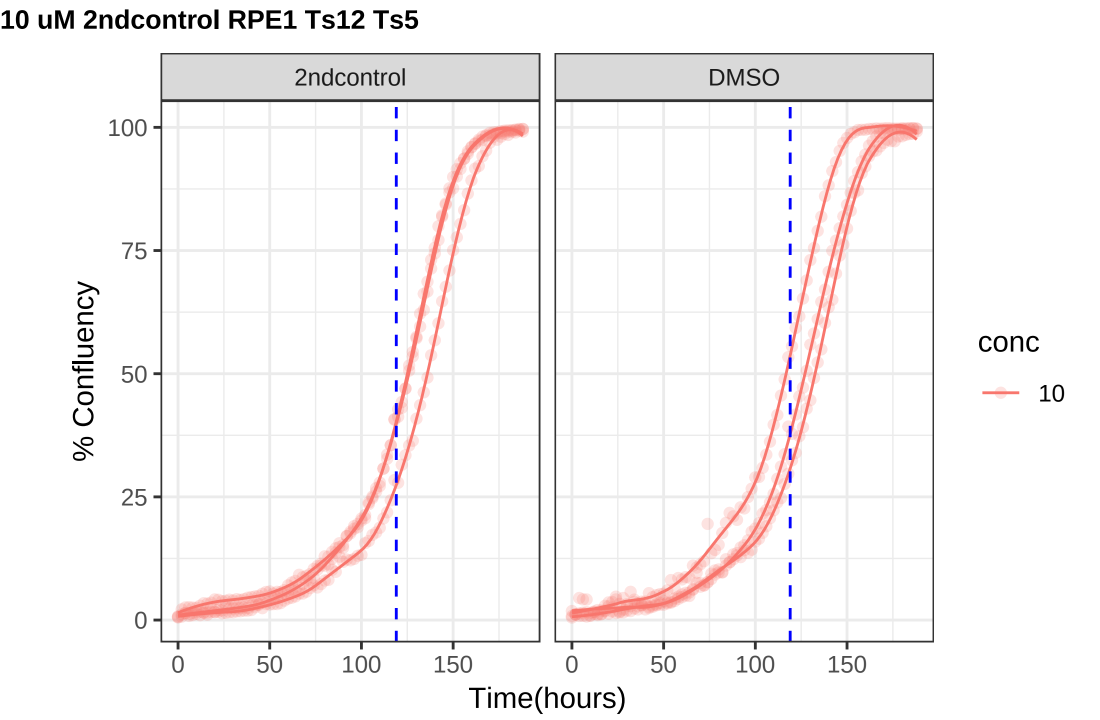

10 uM 2ndcontrol RPE1 Ts12 Ts5
2ndcontrol
DMSO
% Confluency
0
25
50
75
100
0
50
100
150
Time(hours)
conc
10
