# Supplementary material for: Altering microtubule dynamics is synergistically toxic with spindle assembly checkpoint inhibition
Source: Life Sci Alliance. 2020 Jan 24;3(2):e201900499. doi: 10.26508/lsa.201900499 (PMC6985455; doi:10.26508/lsa.201900499)
Supplement: Supplementary file 13 [file LSA-2019-00499_Supplemental_Data_3.pdf]

Supplementary data 3: Drug screen concentration curves

This PDF file contains all growth curves for all 95 drugs used in the aneuploid and CIN screen. One page per drug. Each page contains one or more growth curves comparing RPE1 cells with DMSO and the drug. All drug concentrations start at 10uM. Drugs were screened until they were no longer toxic to RPE1 cells at that concentration, with 1:10 dilution intervals. All concentrations in micromolar (uM). All curves are triplicates.

Drug ID is listed top left corner.

Or

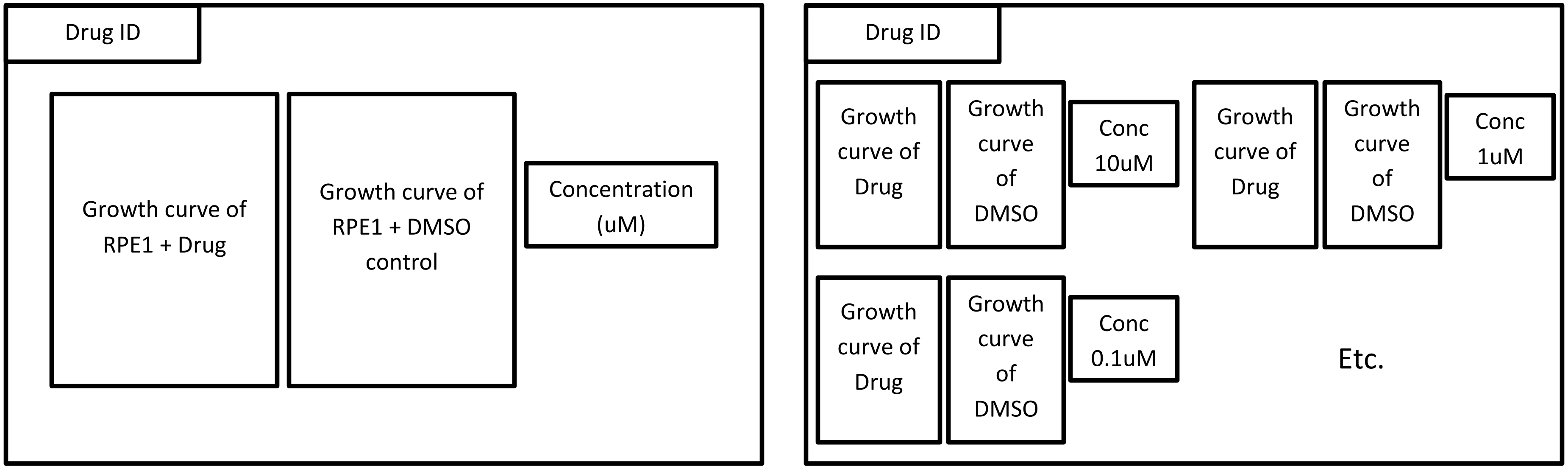

1122

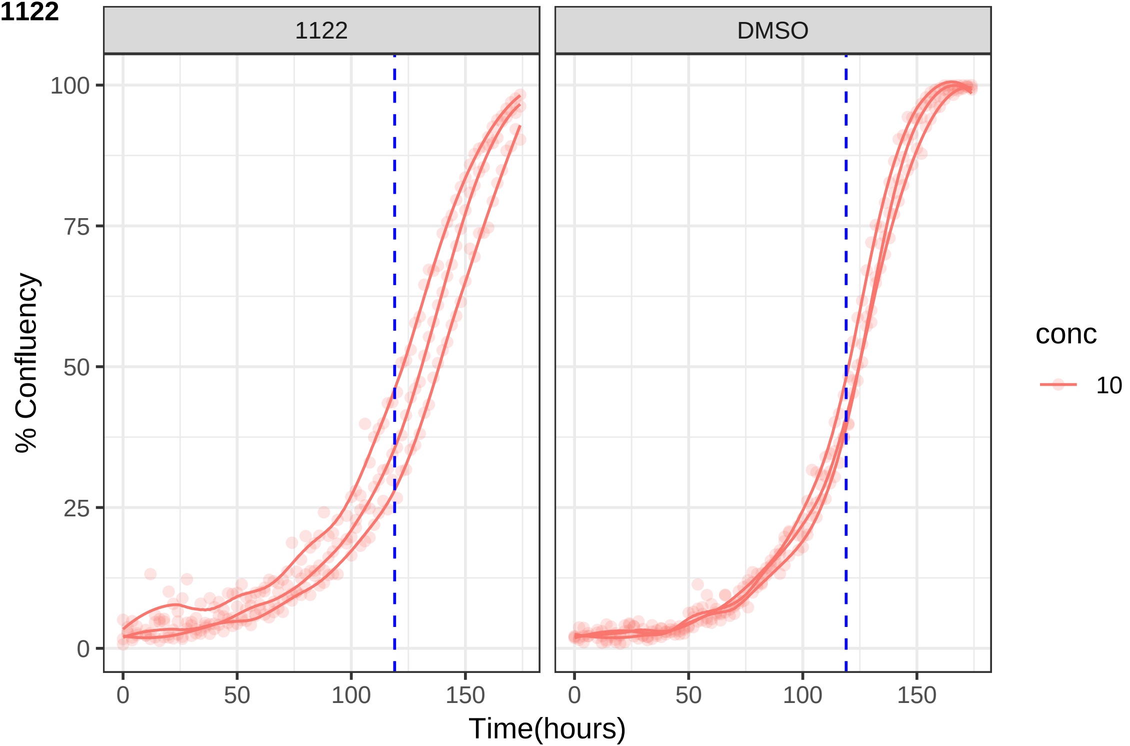
1122
DMSO
100
75
50
25
0
% Confluency
0
50
100
150
Time(hours)
conc
10

1134

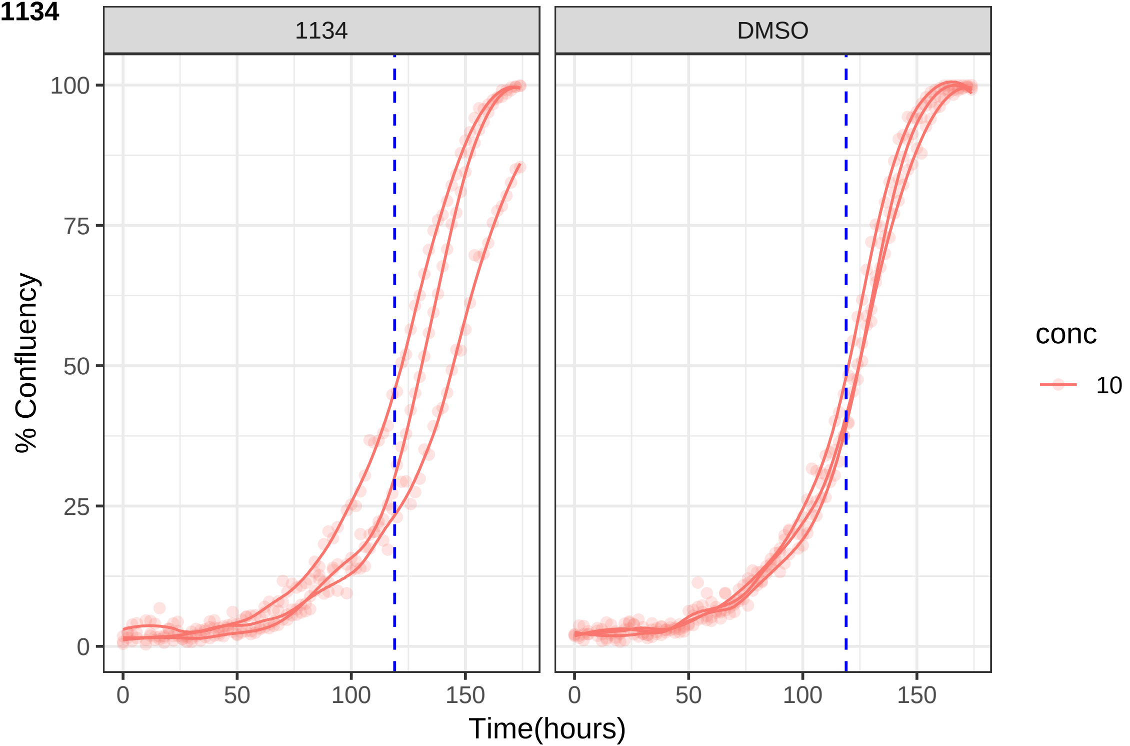
1134
DMSO
% Confluency
100
75
50
25
0
0
50
100
150
Time(hours)
conc
10

1223

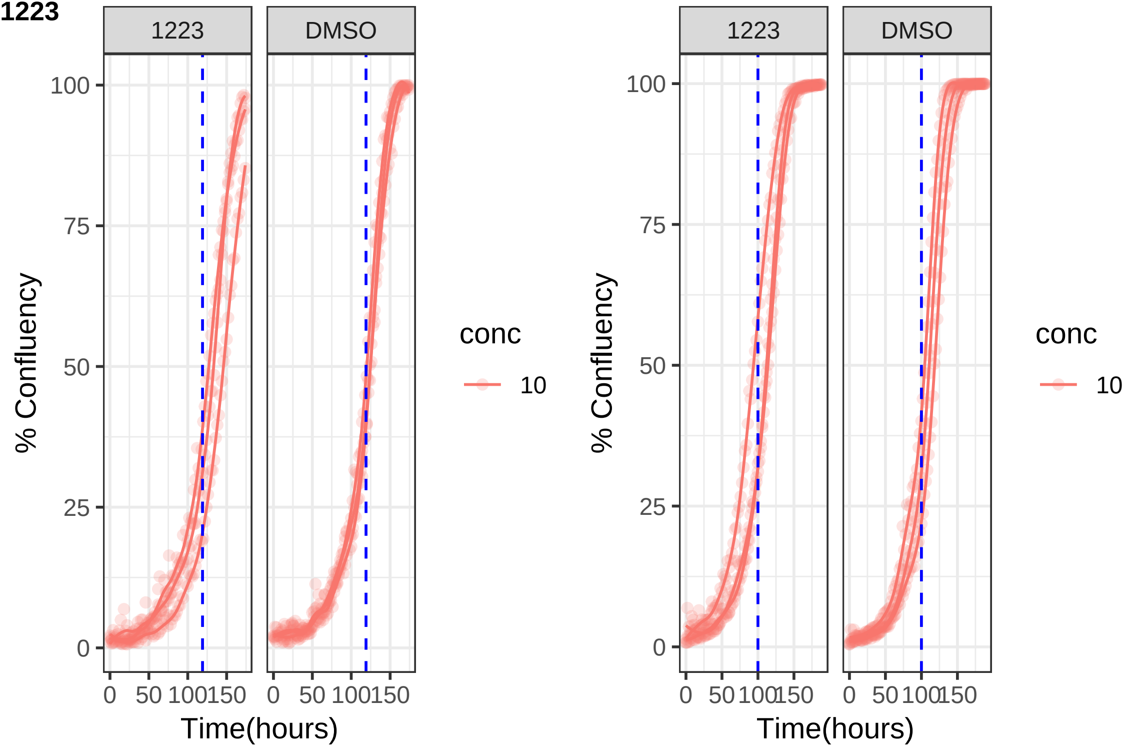

1223
DMSO
100
75
50
25
0
% Confluency
0
50
100
150
Time(hours)
conc
10

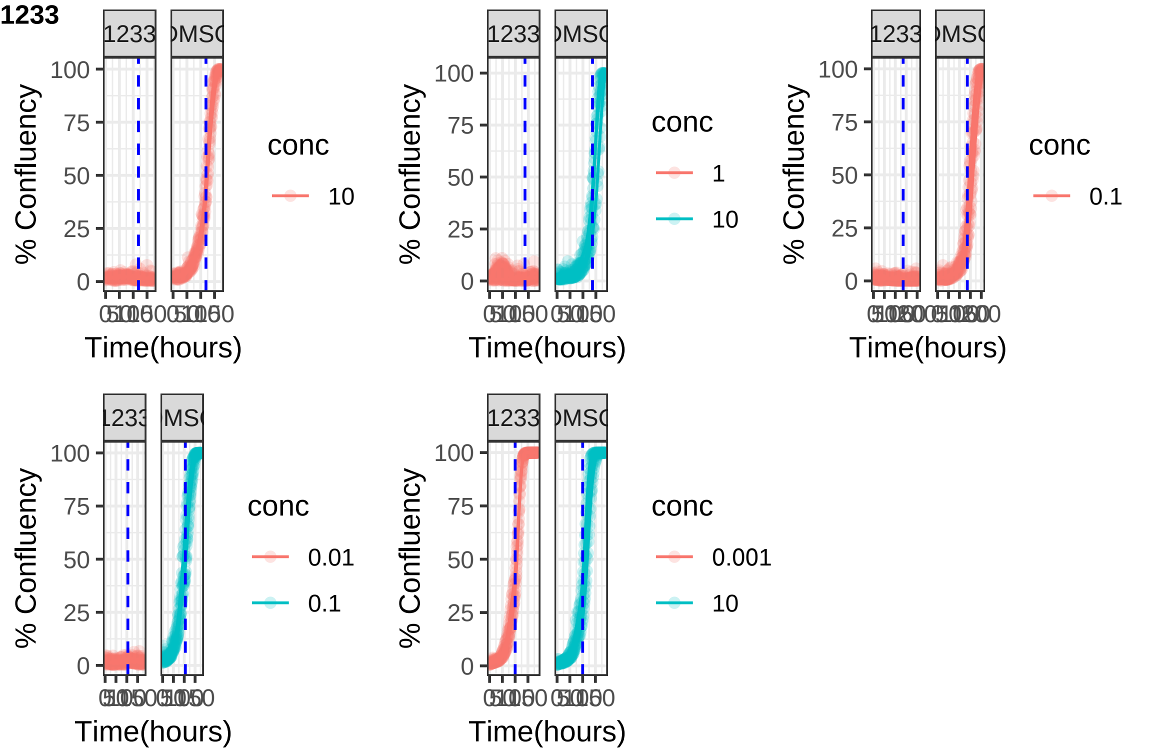
1233
1233
DMSO
100
75
50
25
0
% Confluency
Time(hours)
conc
10
1
0.1
0.01
0.001

1237

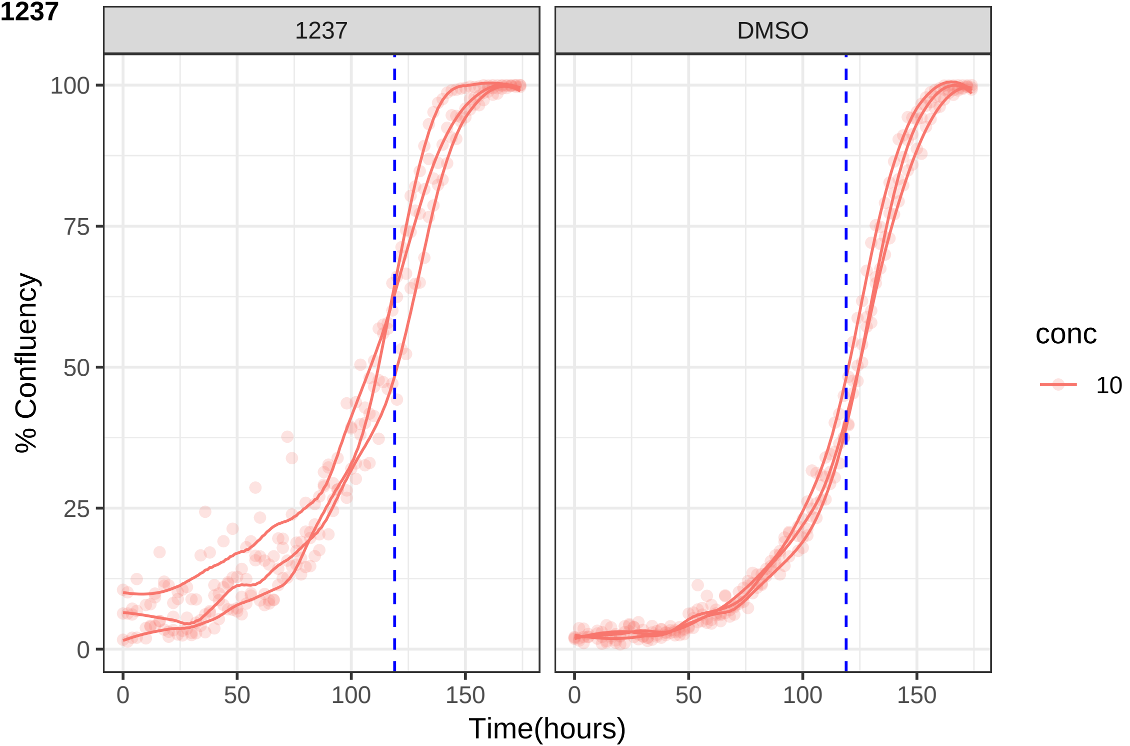

1237
DMSO
% Confluency
100
75
50
25
0
0
50
100
150
Time(hours)
conc
10

1239

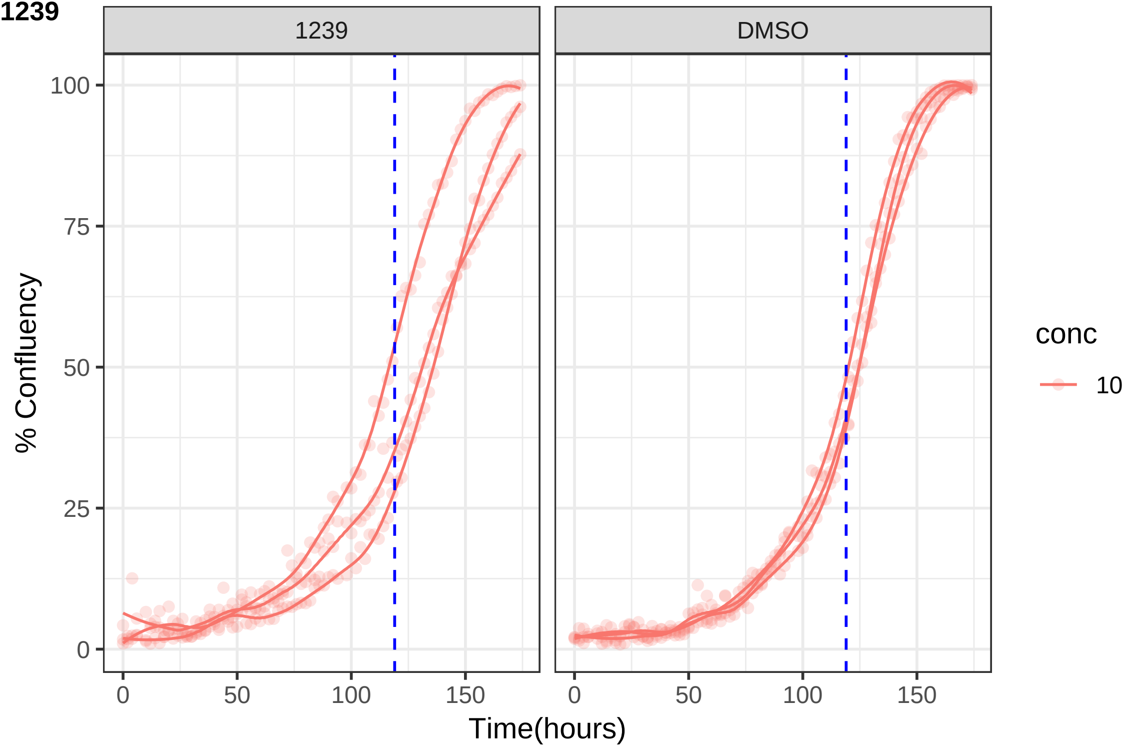

1239
DMSO
100
75
50
25
0
% Confluency
0
50
100
150
Time(hours)
conc
10

1243

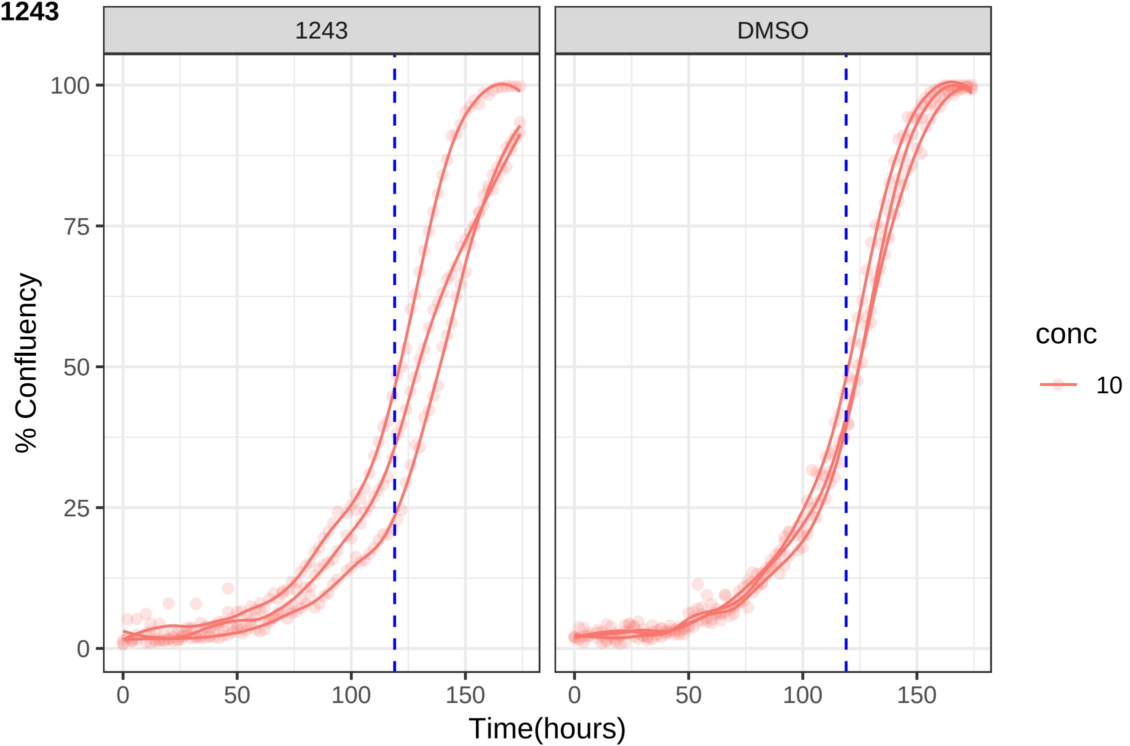
1243
DMSO
100
75
50
25
0
0
50
100
150
% Confluency
Time(hours)
conc
10

1254

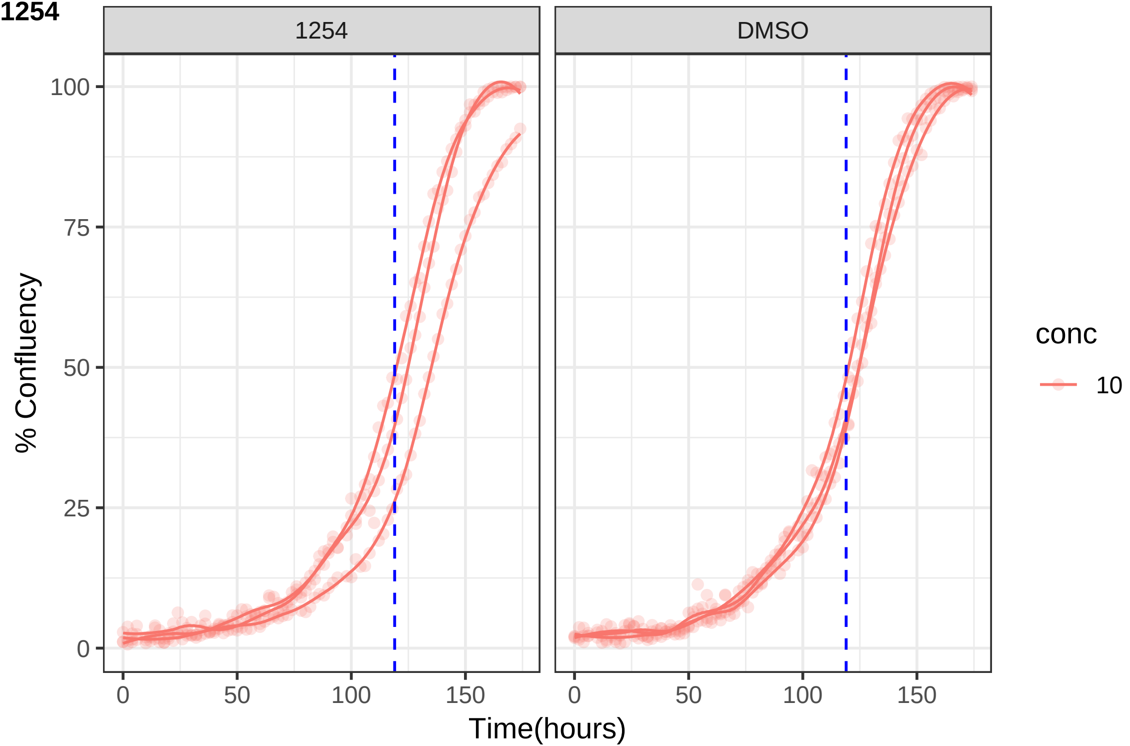
1254
DMSO
% Confluency
0
25
50
75
100
0
50
100
150
Time(hours)
conc
10

1268

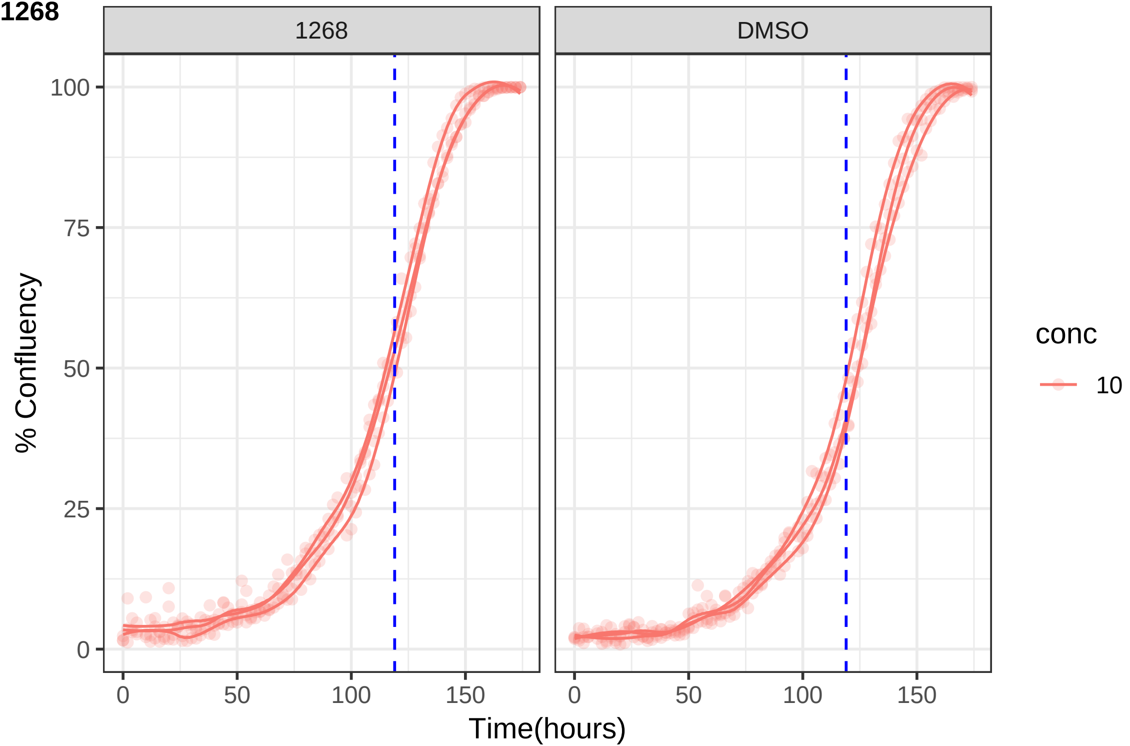

1268
DMSO
% Confluency
Time(hours)
0
25
50
75
100
0
50
100
150
conc
10

1275

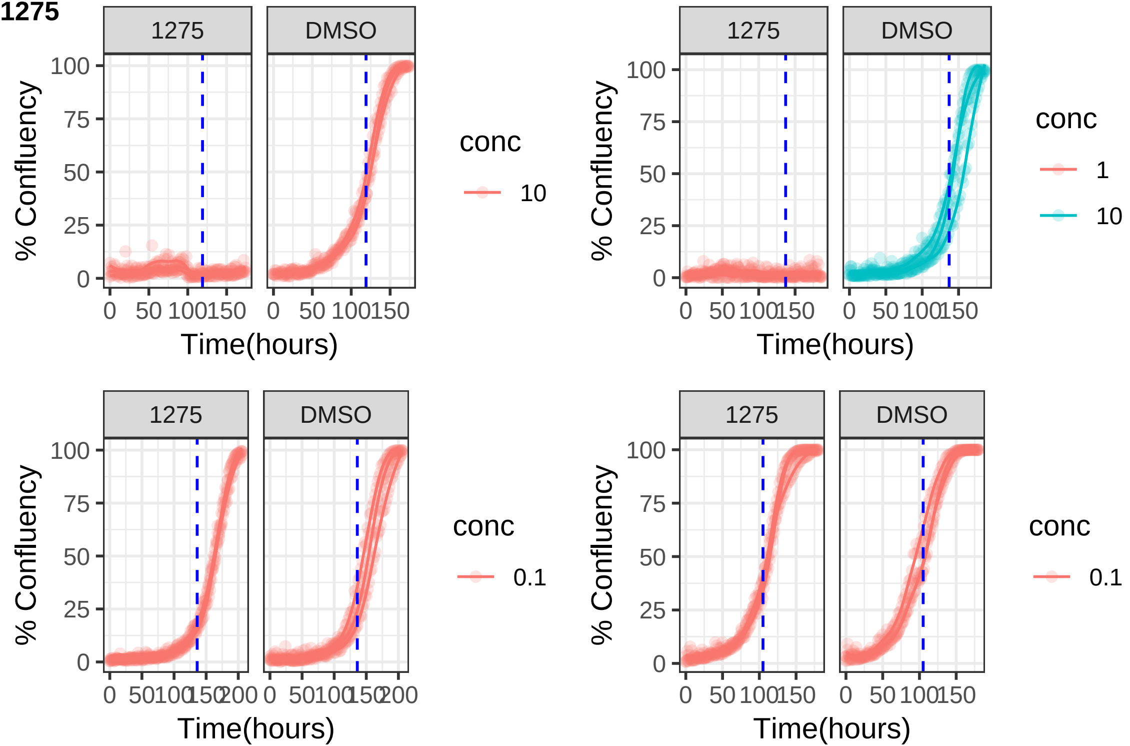

1310

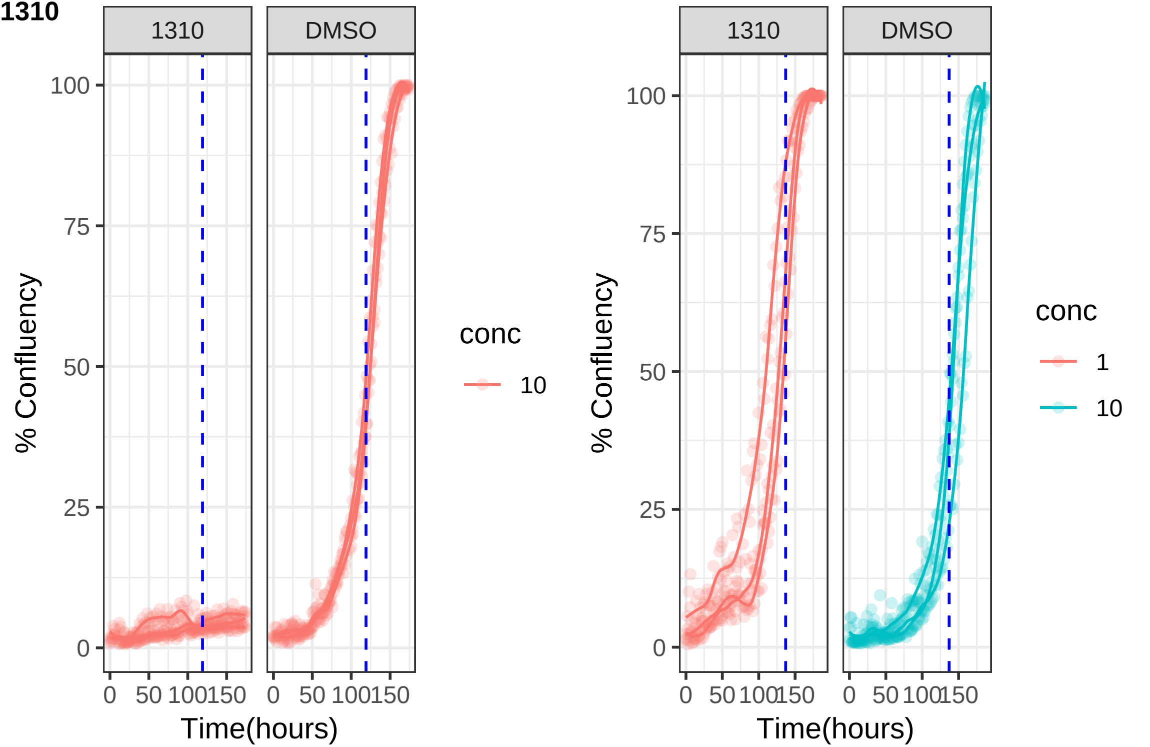
1310
DMSO
% Confluency
100
75
50
25
0
0
50
100
150
Time(hours)
conc
10
1

1334

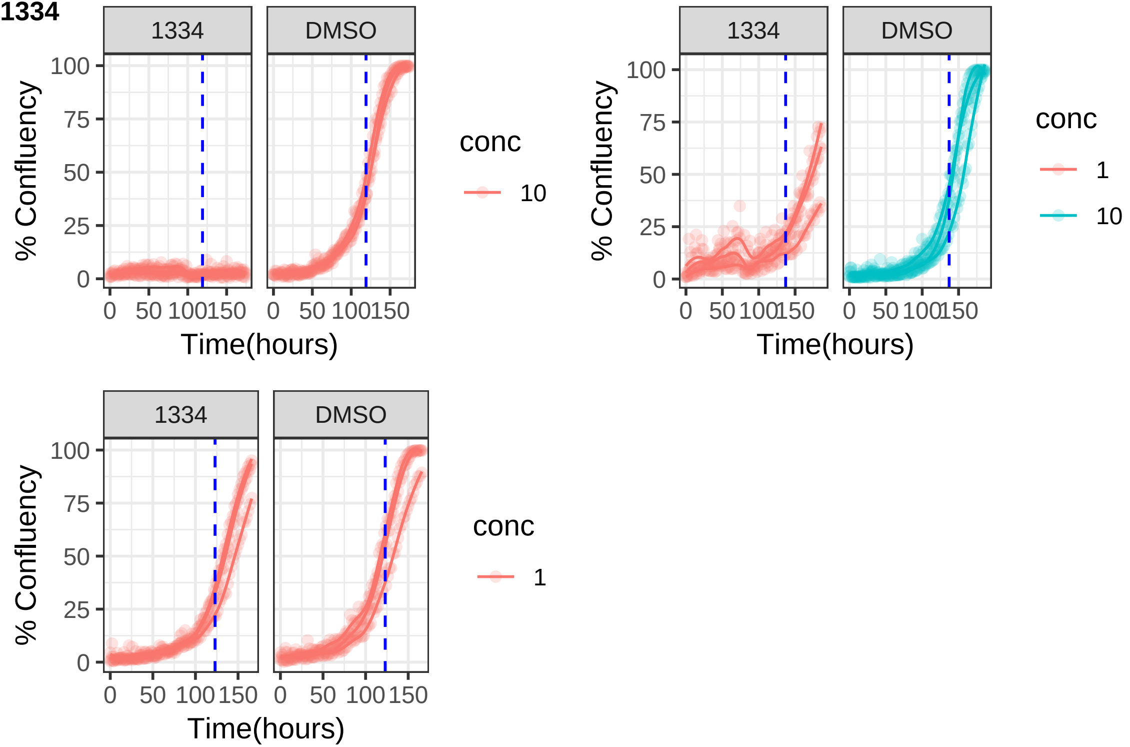

1334
DMSO
100
75
50
25
0
% Confluency
0 50 100 150
Time(hours)
conc
10
1

1355

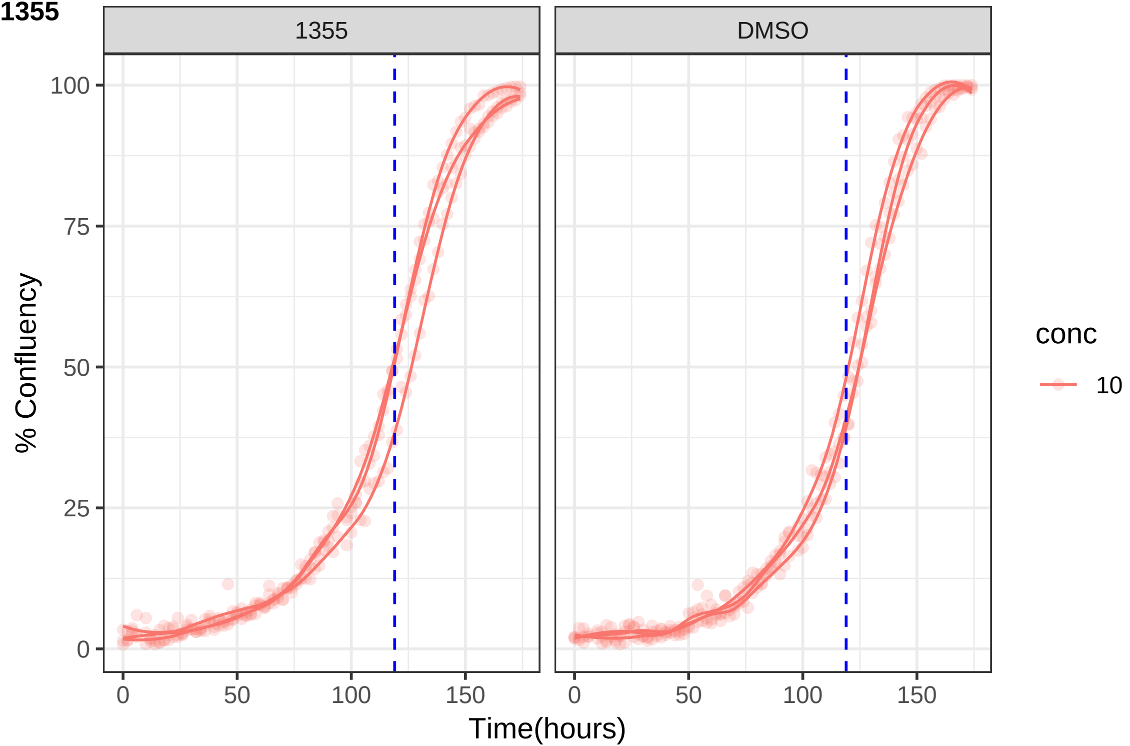

1355
DMSO
% Confluency
Time(hours)
conc
10
100
75
50
25
0
0
50
100
150

1356

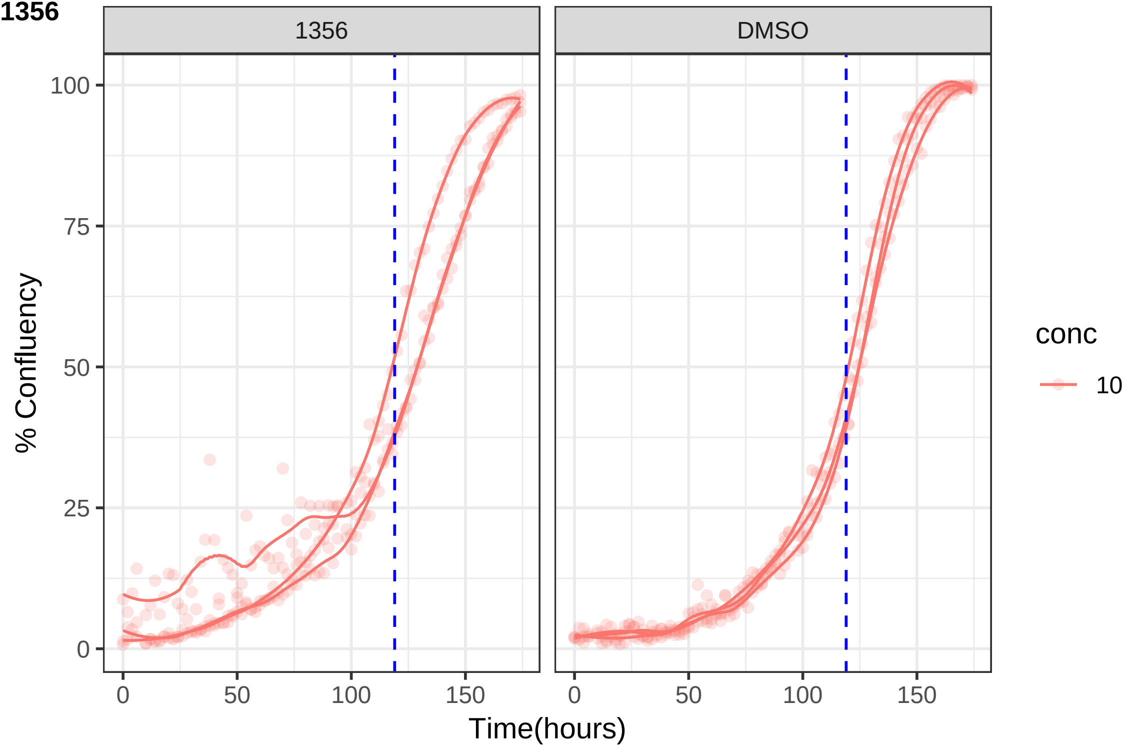

1356
DMSO
100
75
50
25
0
% Confluency
0
50
100
150
Time(hours)
conc
10

1362

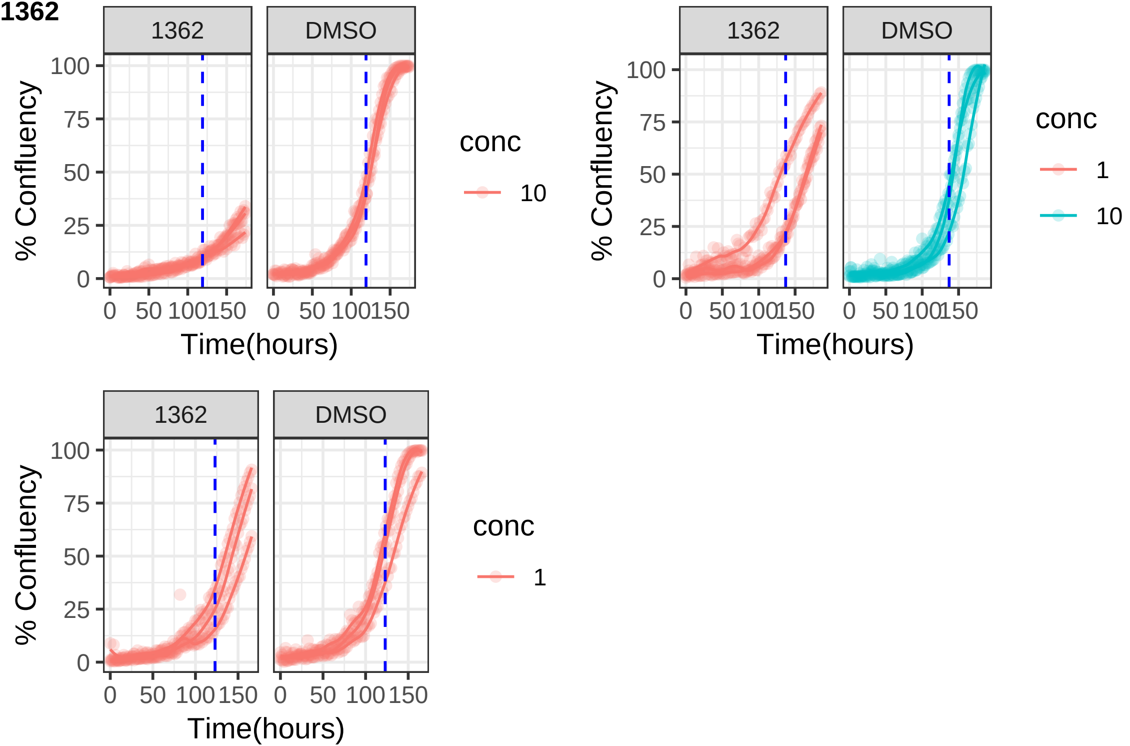

1362
DMSO
100
75
50
25
0
% Confluency
0
50
100
150
Time(hours)
conc
10
1

1375

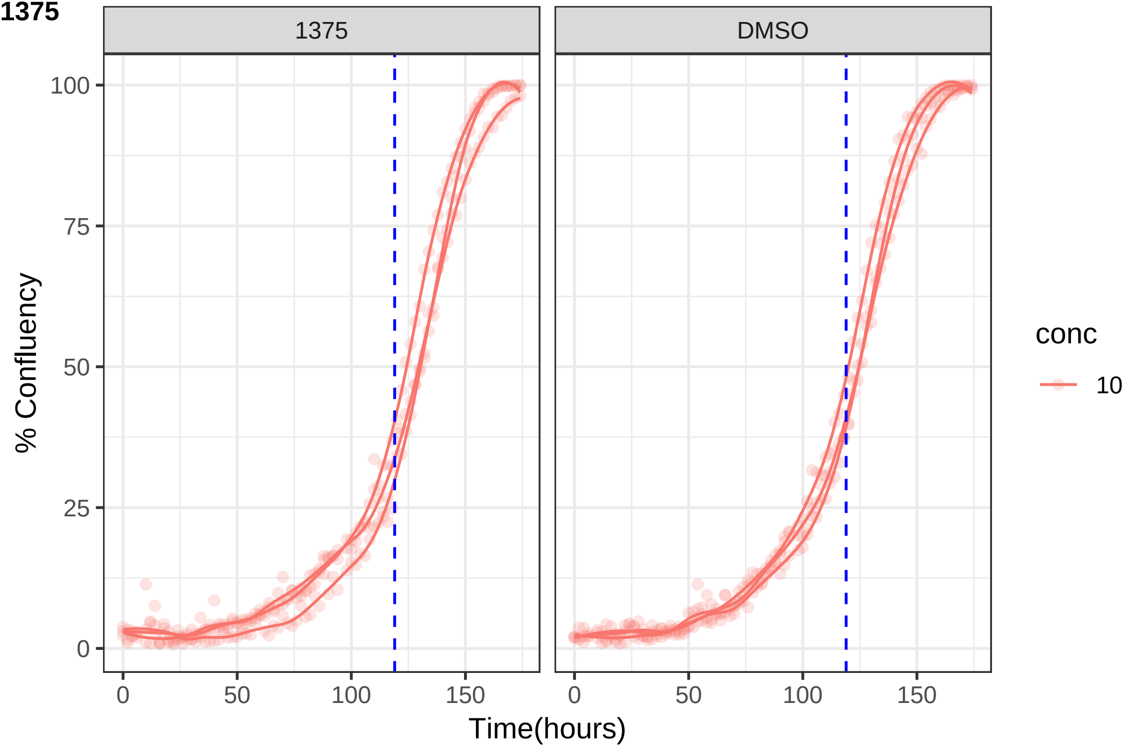
1375
DMSO
% Confluency
Time(hours)
conc
10
0
25
50
75
100
0
50
100
150

1377

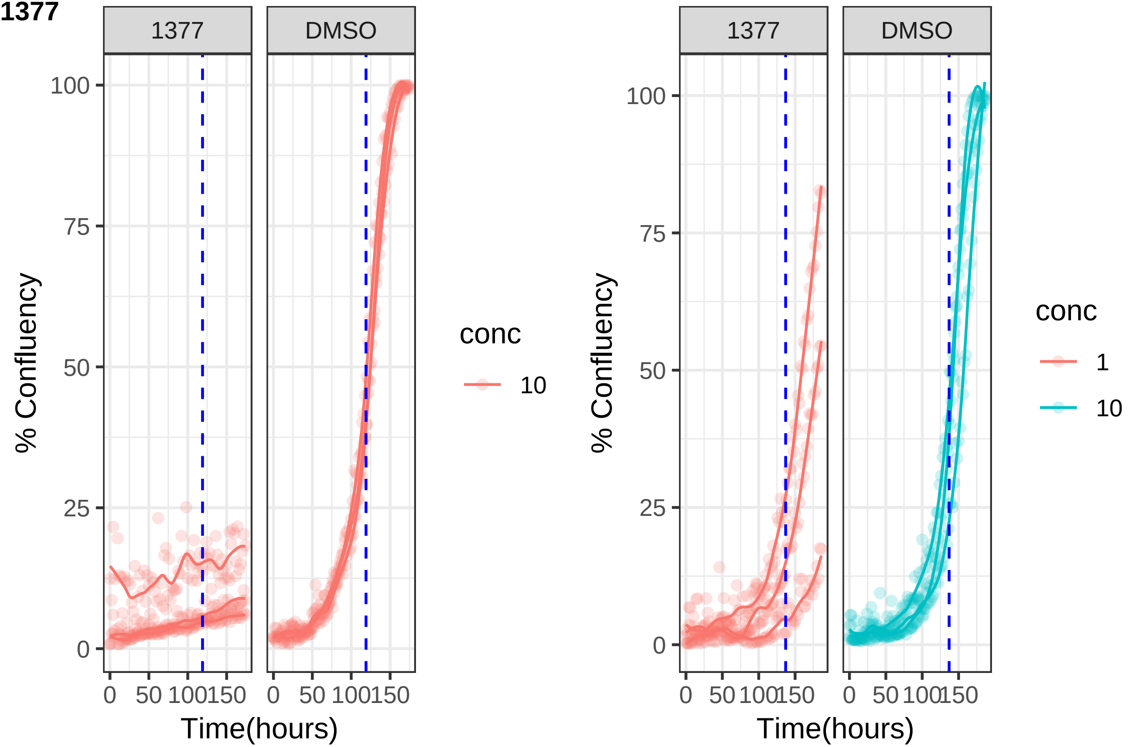
1377
DMSO
% Confluency
100
75
50
25
0
0
50
100
150
Time(hours)
conc
10
1

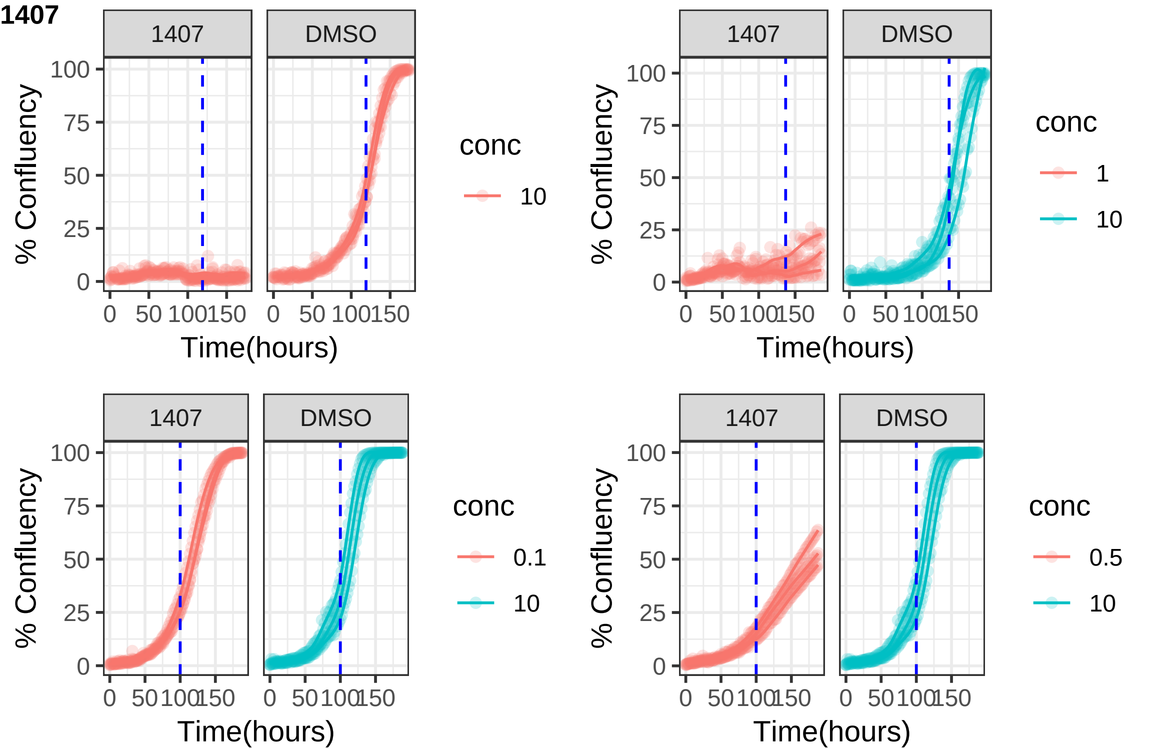
1407
1407
DMSO
% Confluency
100
75
50
25
0
0 50 100 150
Time(hours)
conc
10
1
0.1
0.5

1424

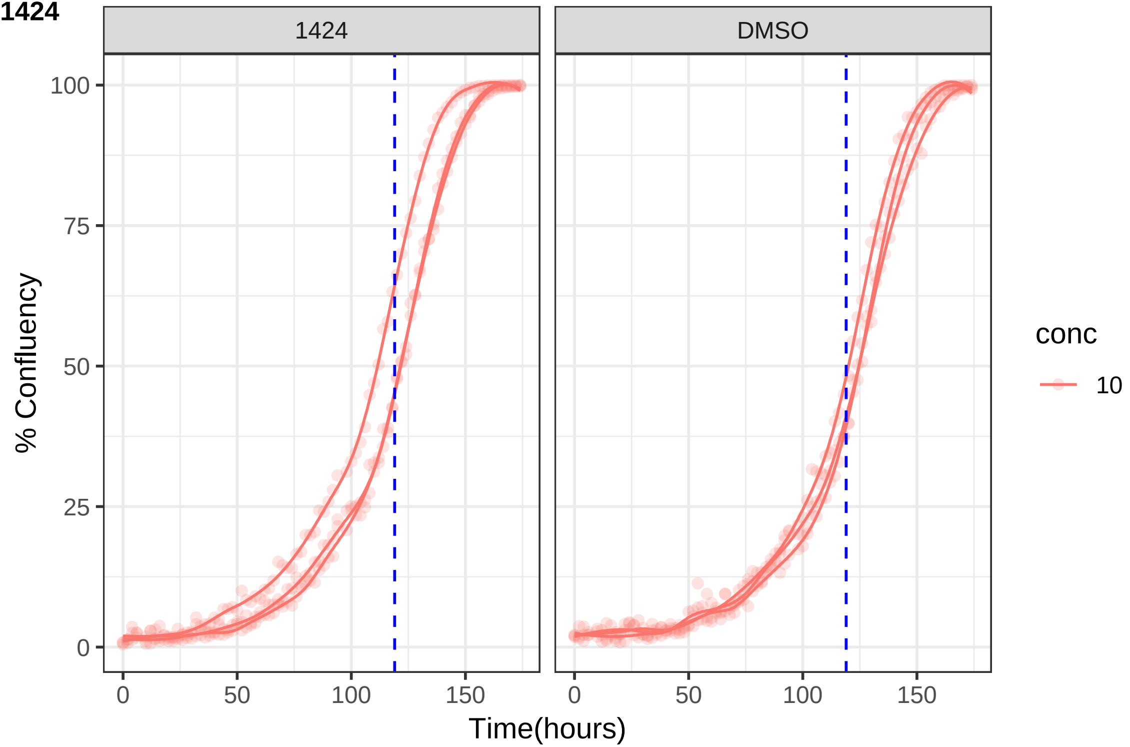

1424
DMSO
100
75
50
25
0
% Confluency
0
50
100
150
Time(hours)
conc
10

1459

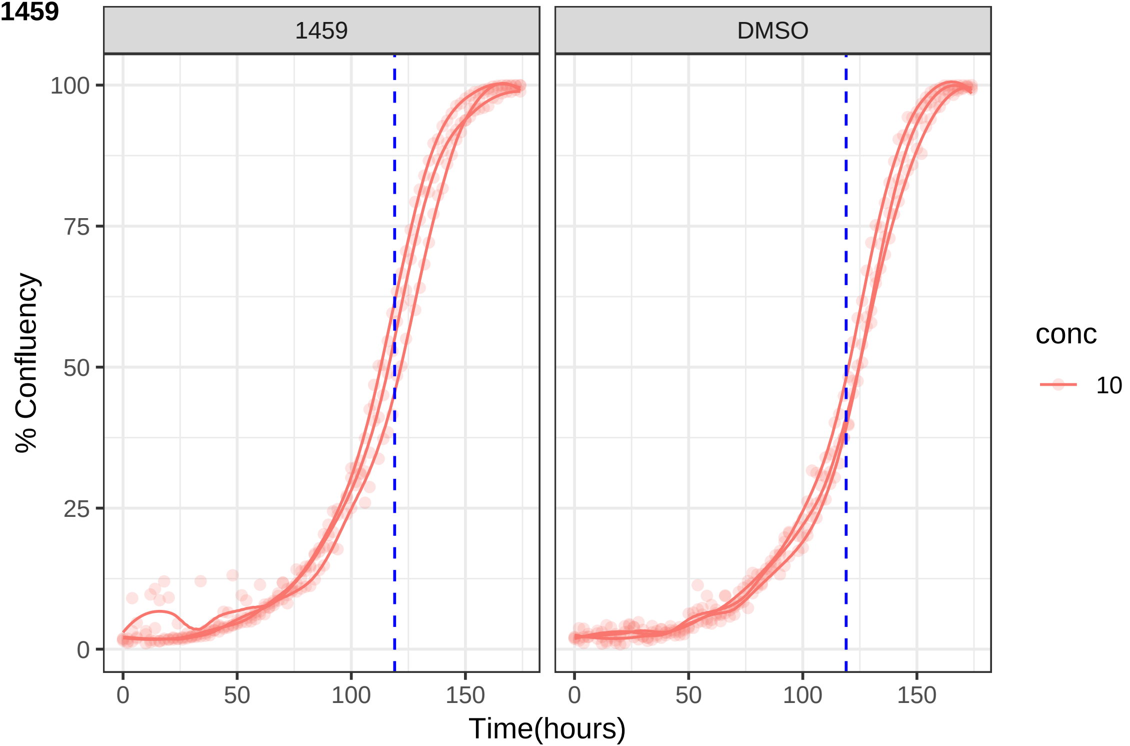

1459
DMSO
100
75
50
25
0
% Confluency
0
50
100
150
Time(hours)
conc
10

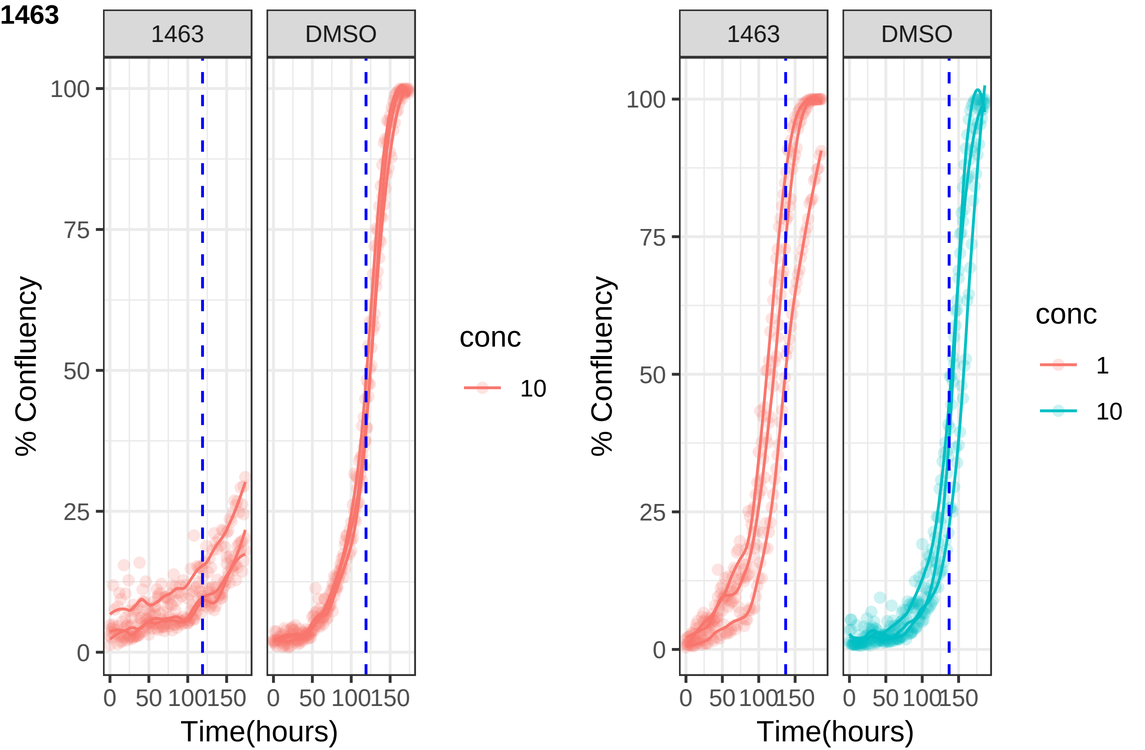
1463
1463
DMSO
100
75
50
25
0
% Confluency
0
50
100
150
Time(hours)
conc
10
1463
DMSO
100
75
50
25
0
% Confluency
0
50
100
150
Time(hours)
conc
1
10

1472

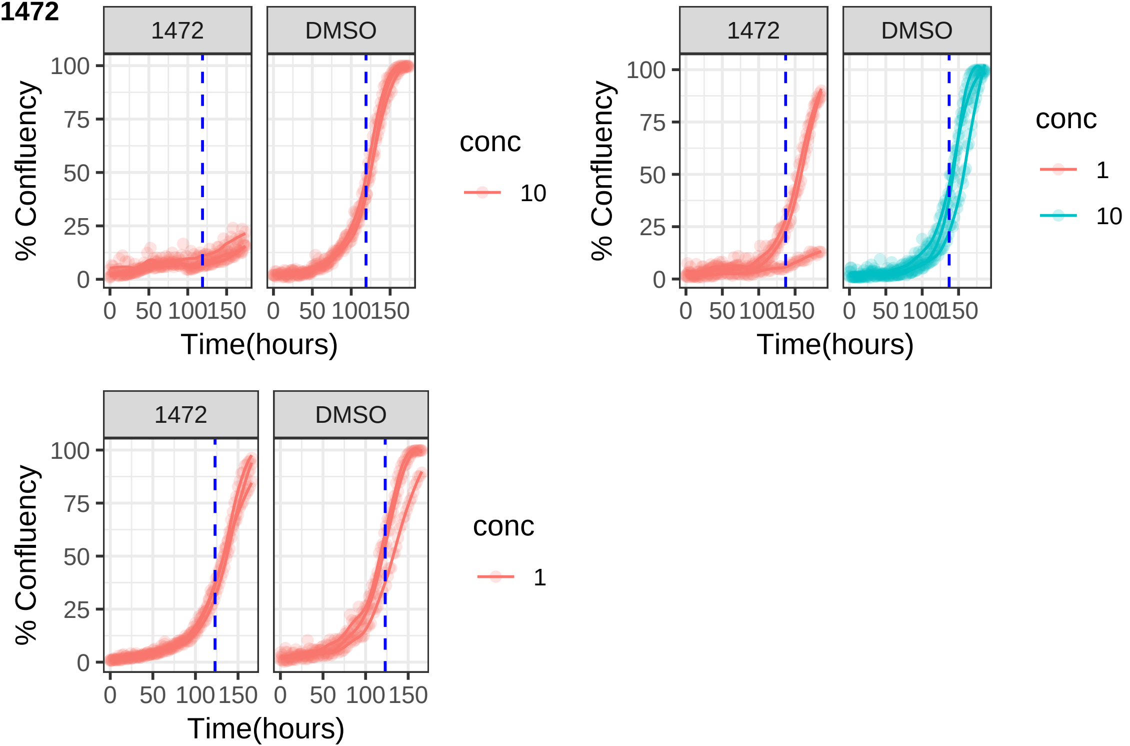
1472
DMSO
% Confluency
0
25
50
75
100
0
50
100
150
Time(hours)
conc
10
1472
DMSO
% Confluency
Time(hours)
conc
1
10
1472
DMSO
% Confluency
Time(hours)
conc
1

1494

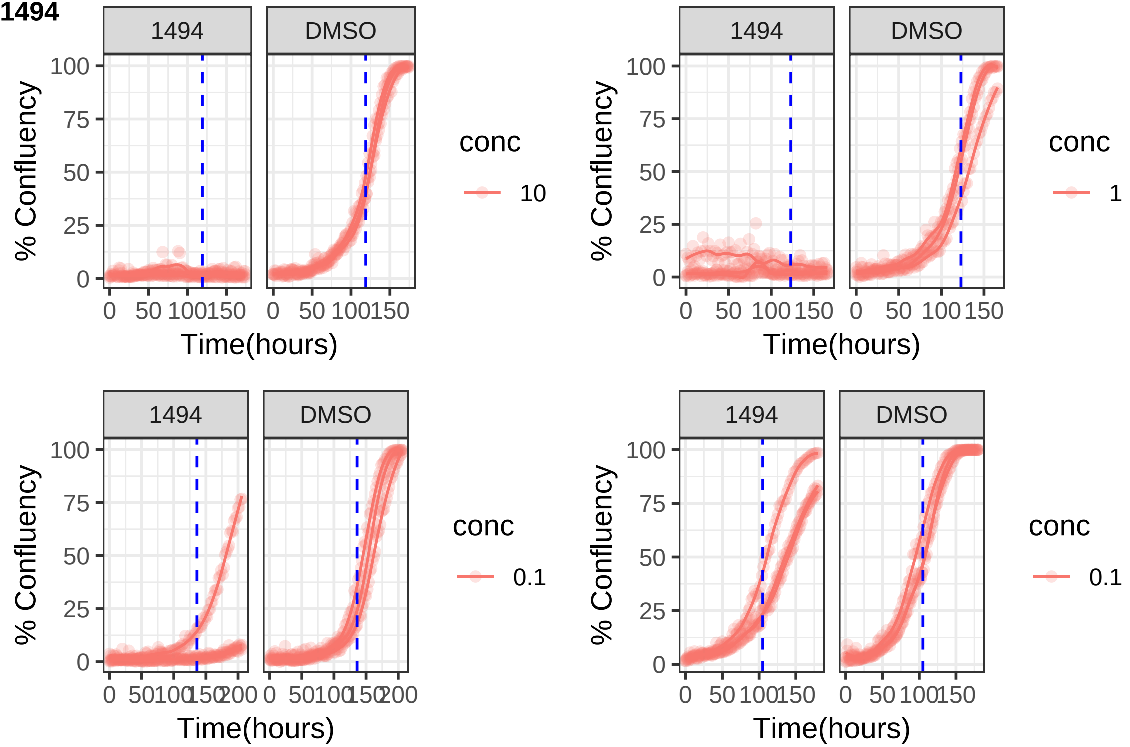

1516

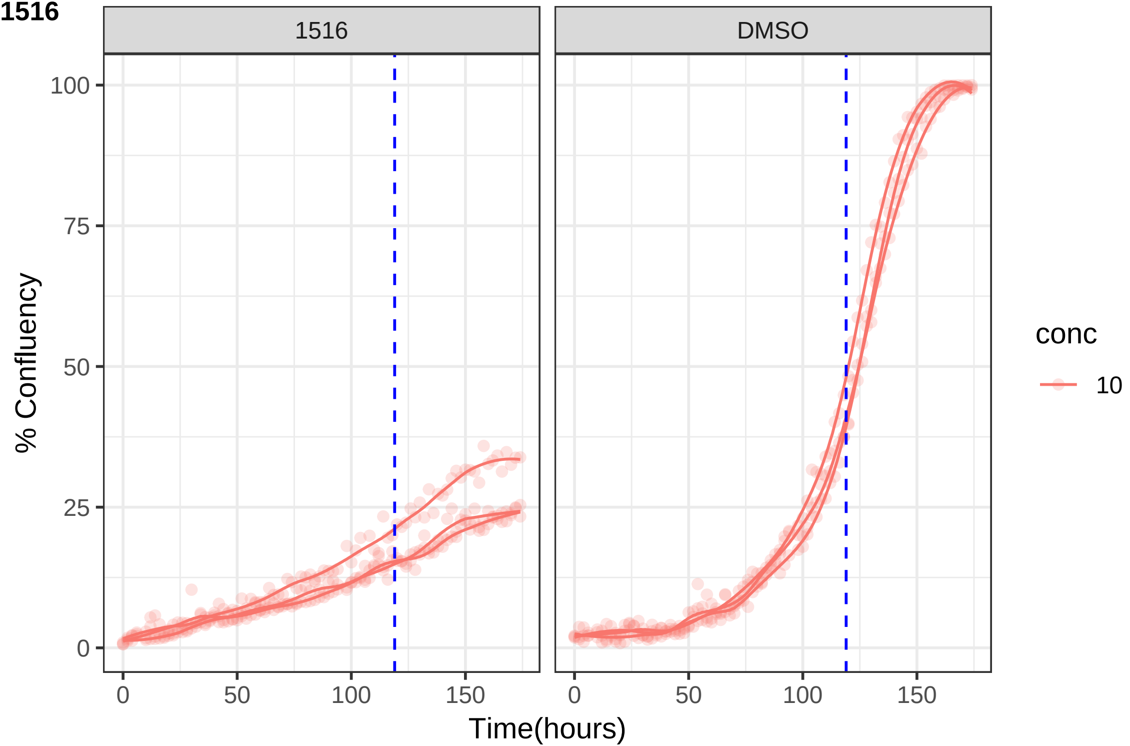
1516
DMSO
100
75
50
25
0
% Confluency
0
50
100
150
Time(hours)
conc
10

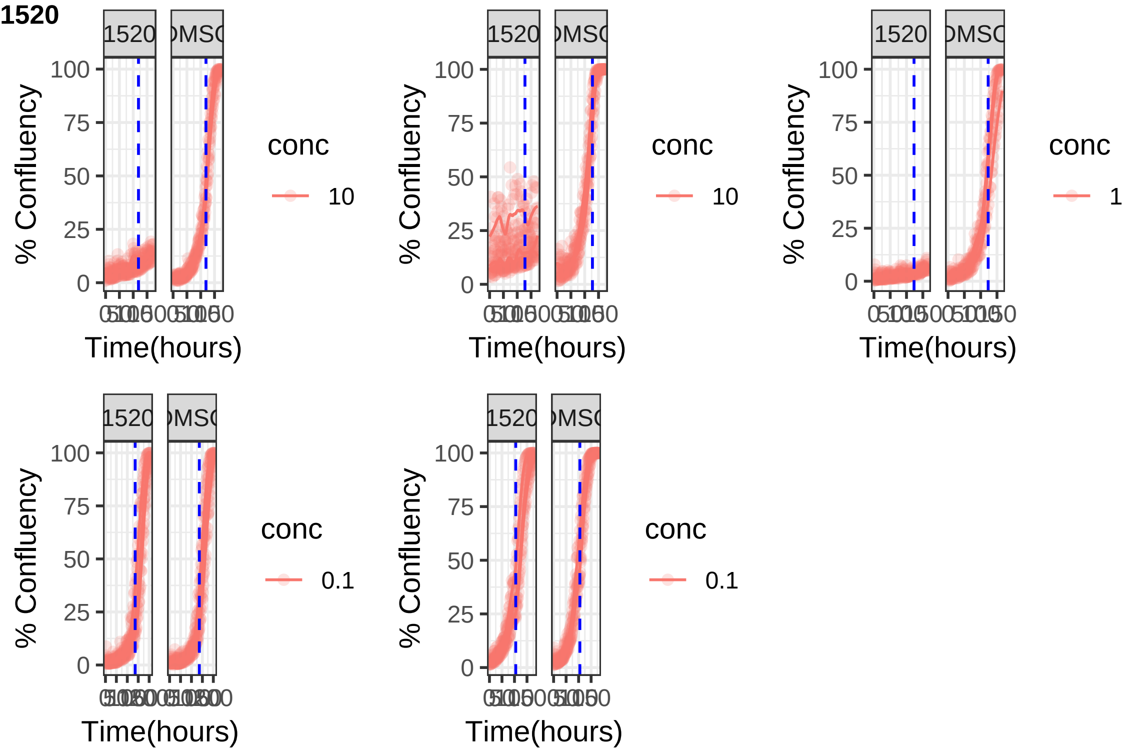
1520
1520
MSO
% Confluency
100
75
50
25
0
Time(hours)
conc
10
1
0.1

1561

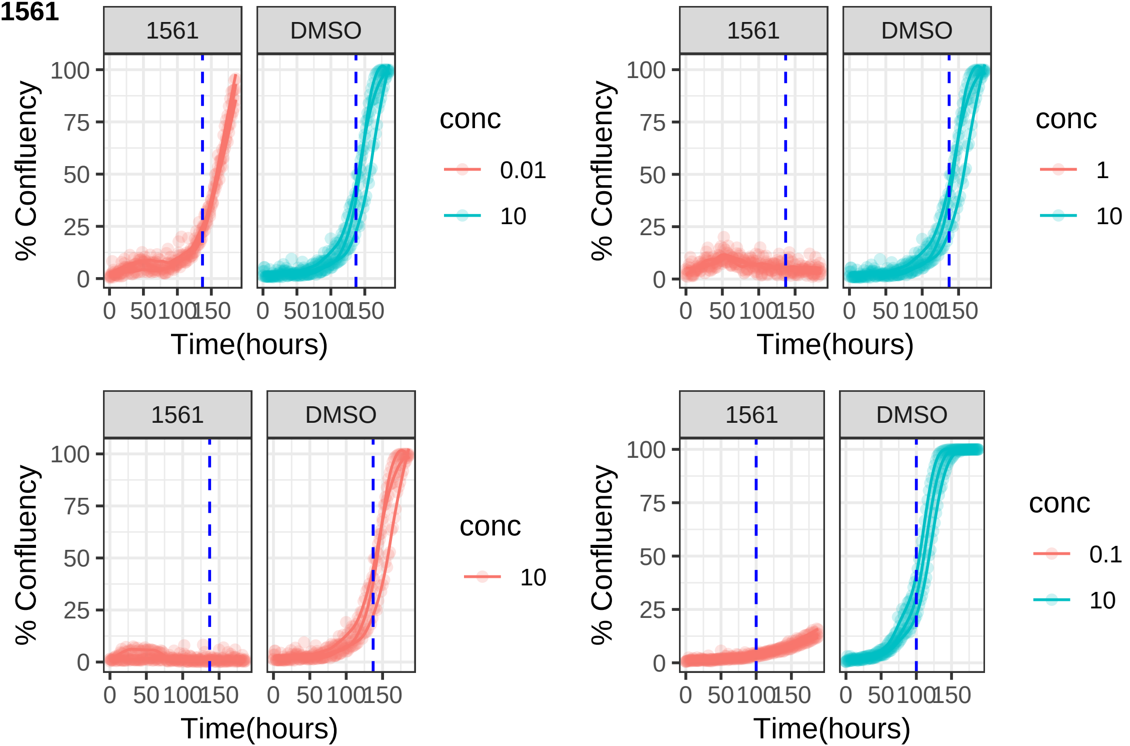

1561
DMSO
% Confluency
100
75
50
25
0
0
50
100
150
Time(hours)
conc
0.01
10
1
10
10
0.1
10

1566

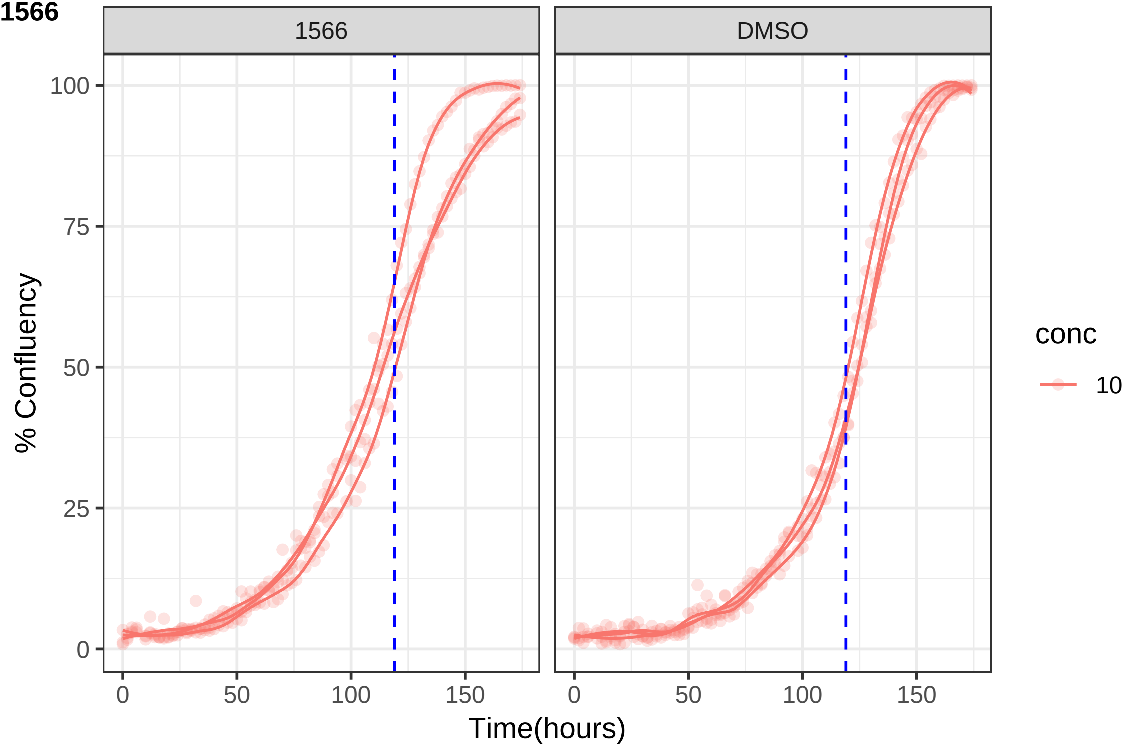

1566
DMSO
100
75
50
25
0
% Confluency
0
50
100
150
Time(hours)
conc
10

1596

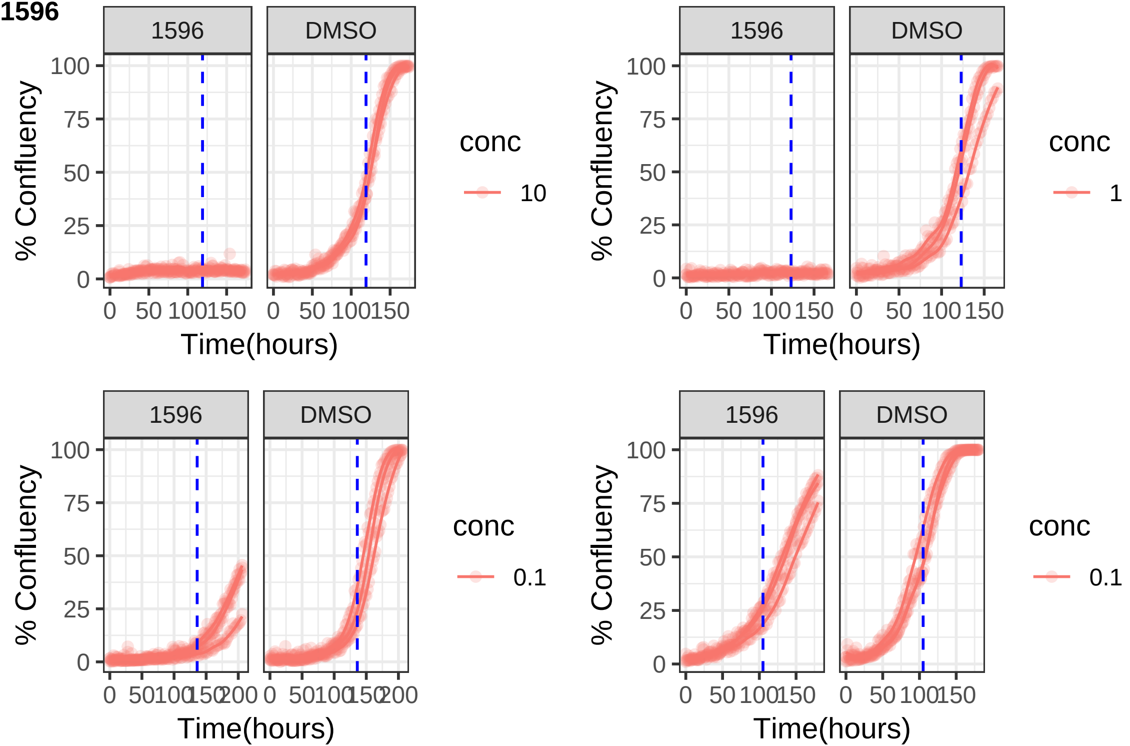
1596
DMSO
% Confluency
100
75
50
25
0
0
50
100
150
200
Time(hours)
conc
10
1
0.1

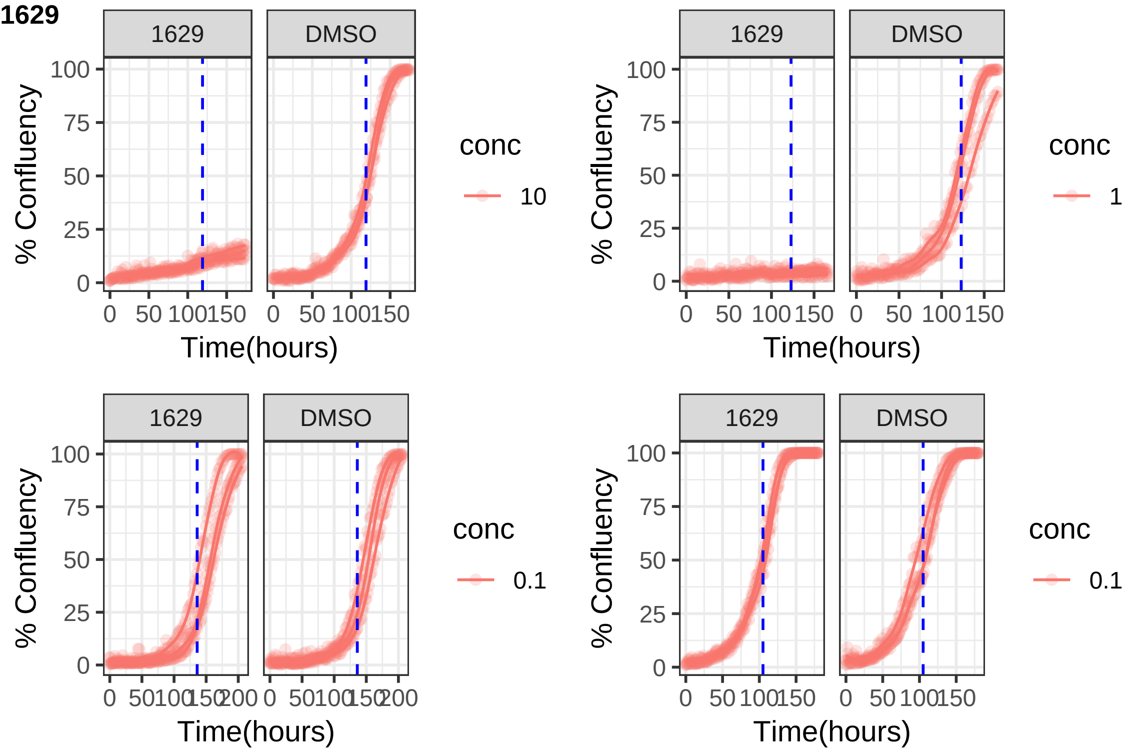
1629
1629
DMSO
% Confluency
Time(hours)
conc
10
1
0.1
0
25
50
75
100
0
50
100
150
200

1645

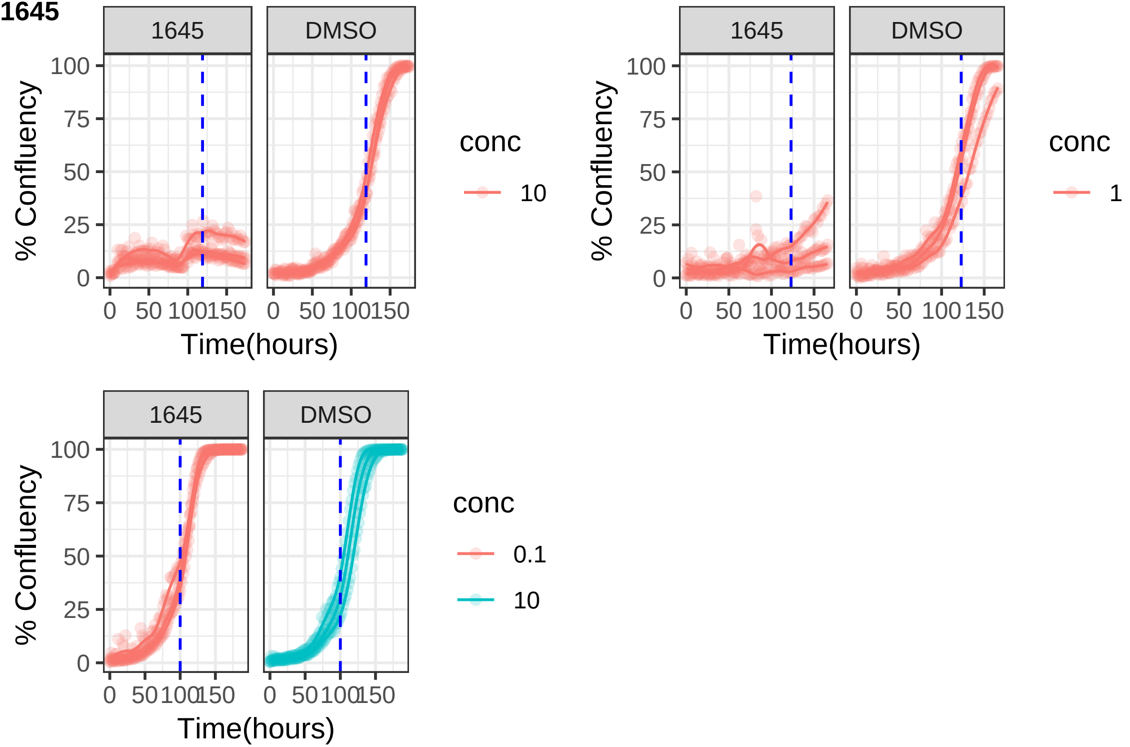
1645
DMSO
% Confluency
Time(hours)
conc
10
1
0.1
0
25
50
75
100
0
50
100
150

1687

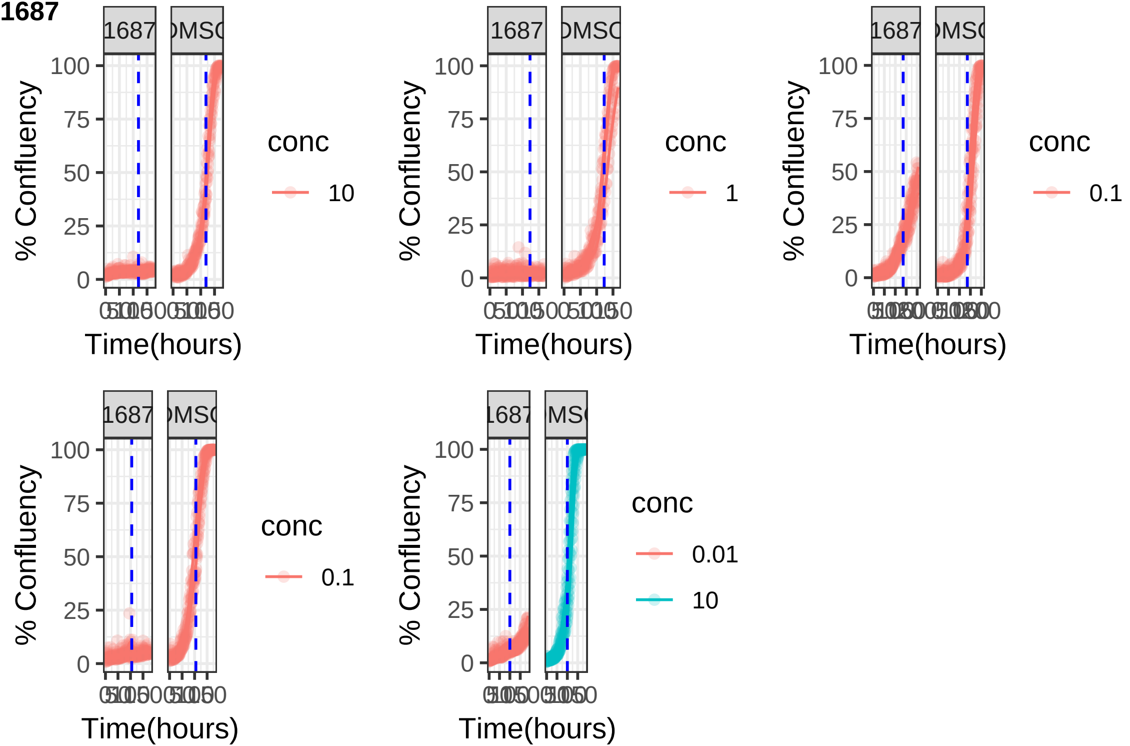

1691

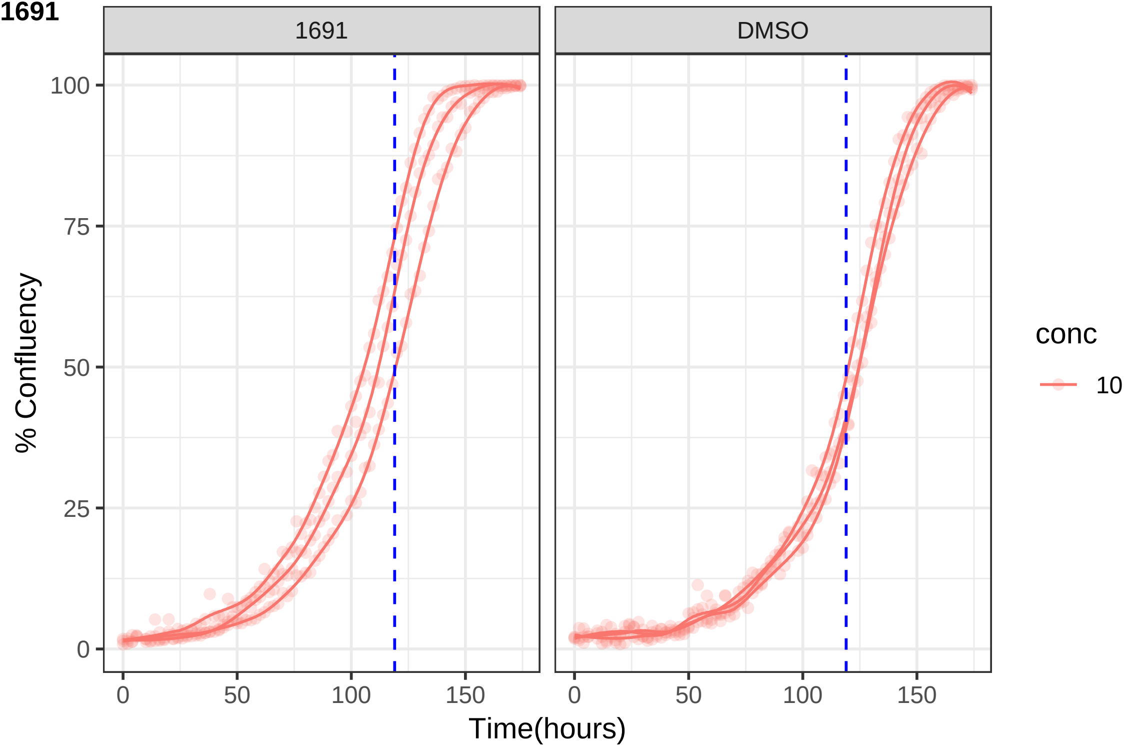
1691
DMSO
% Confluency
100
75
50
25
0
0
50
100
150
Time(hours)
conc
10

1729

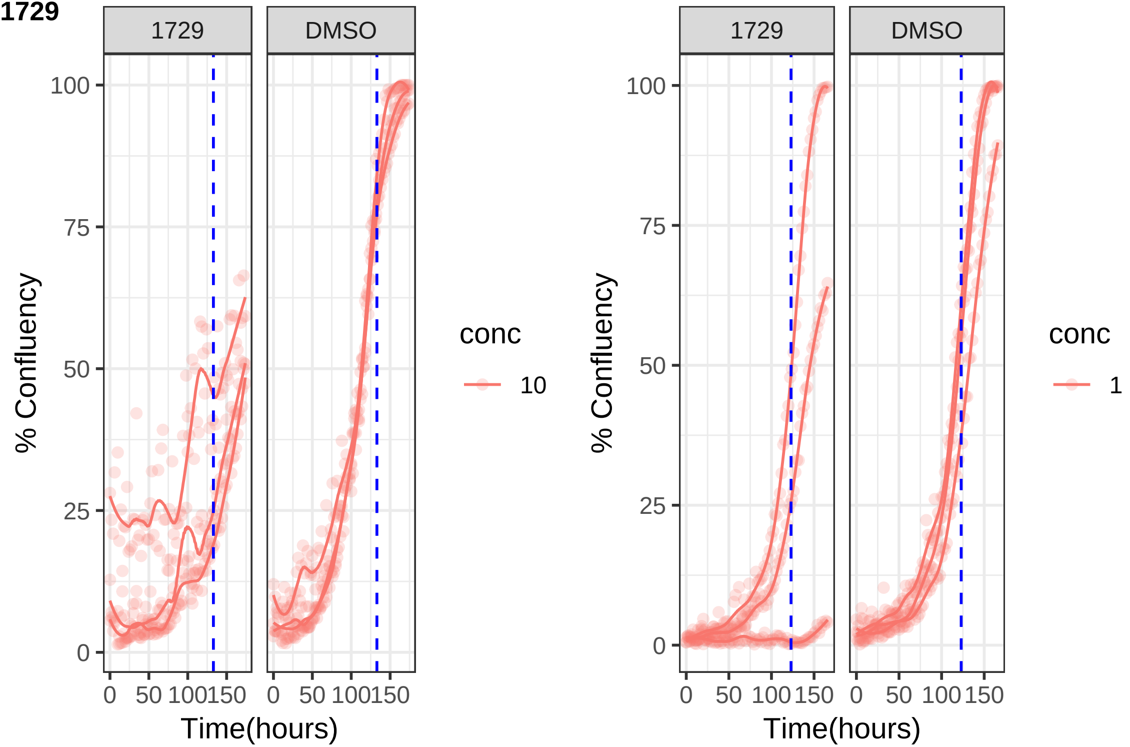

1729
DMSO
100
75
50
25
0
% Confluency
0
50
100
150
Time(hours)
conc
10
1729
DMSO
100
75
50
25
0
% Confluency
0
50
100
150
Time(hours)
conc
1

1743

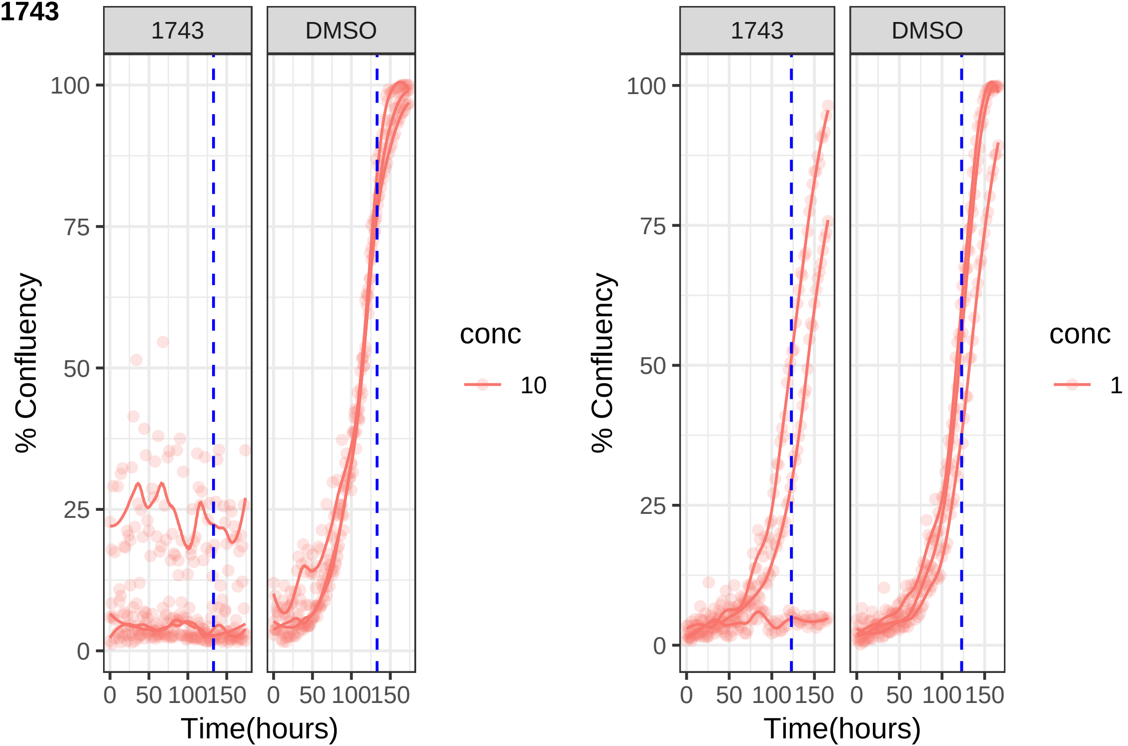

1743
DMSO
% Confluency
Time(hours)
conc
10
1743
DMSO
% Confluency
Time(hours)
conc
1

1765

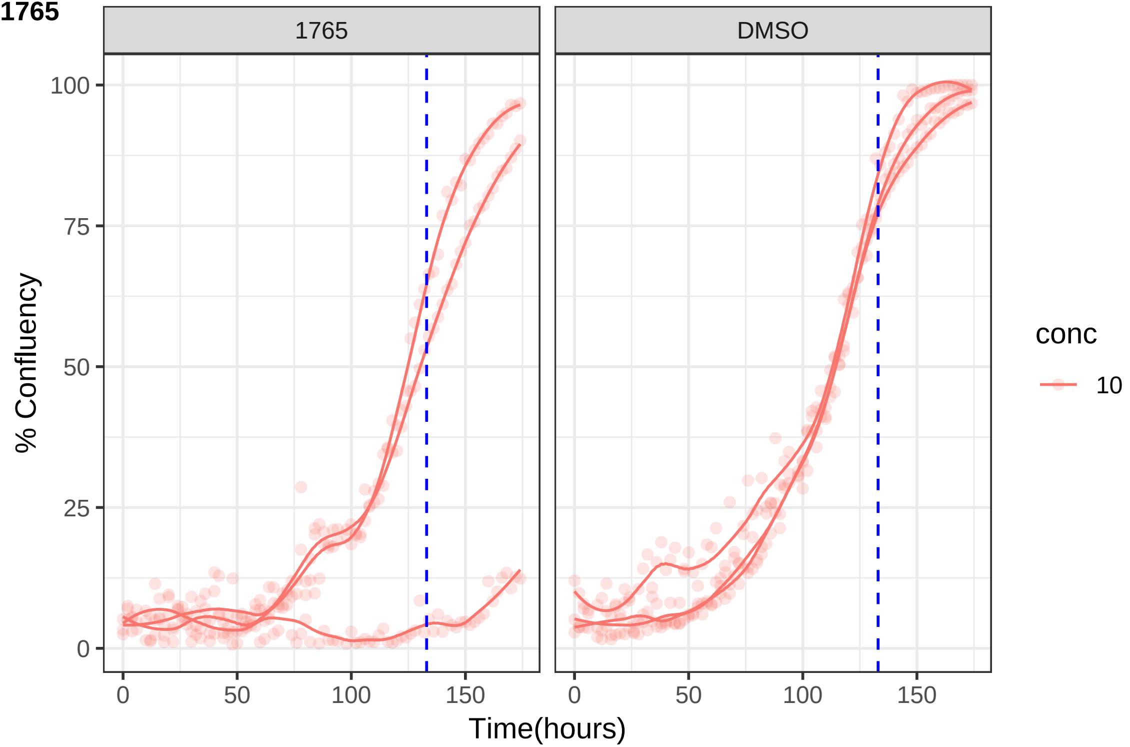

1765
DMSO
% Confluency
100
75
50
25
0
0
50
100
150
0
50
100
150
Time(hours)
conc
10

1789

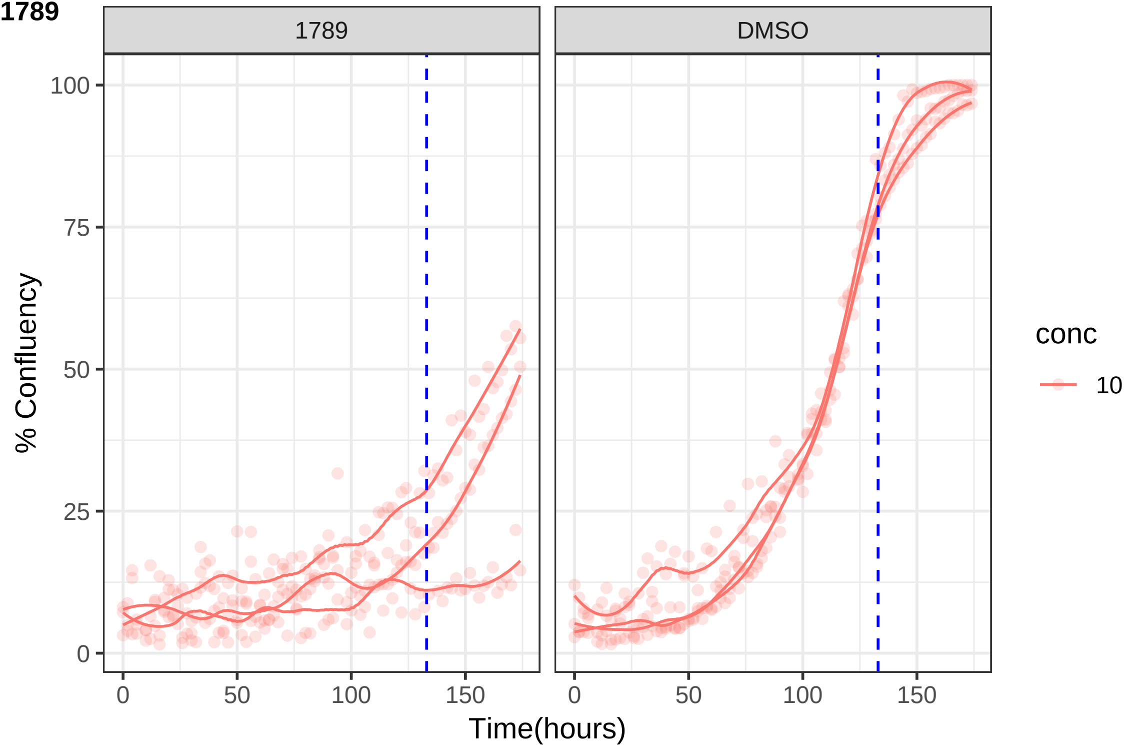

1789
DMSO
% Confluency
100
75
50
25
0
0
50
100
150
Time(hours)
conc
10

1797

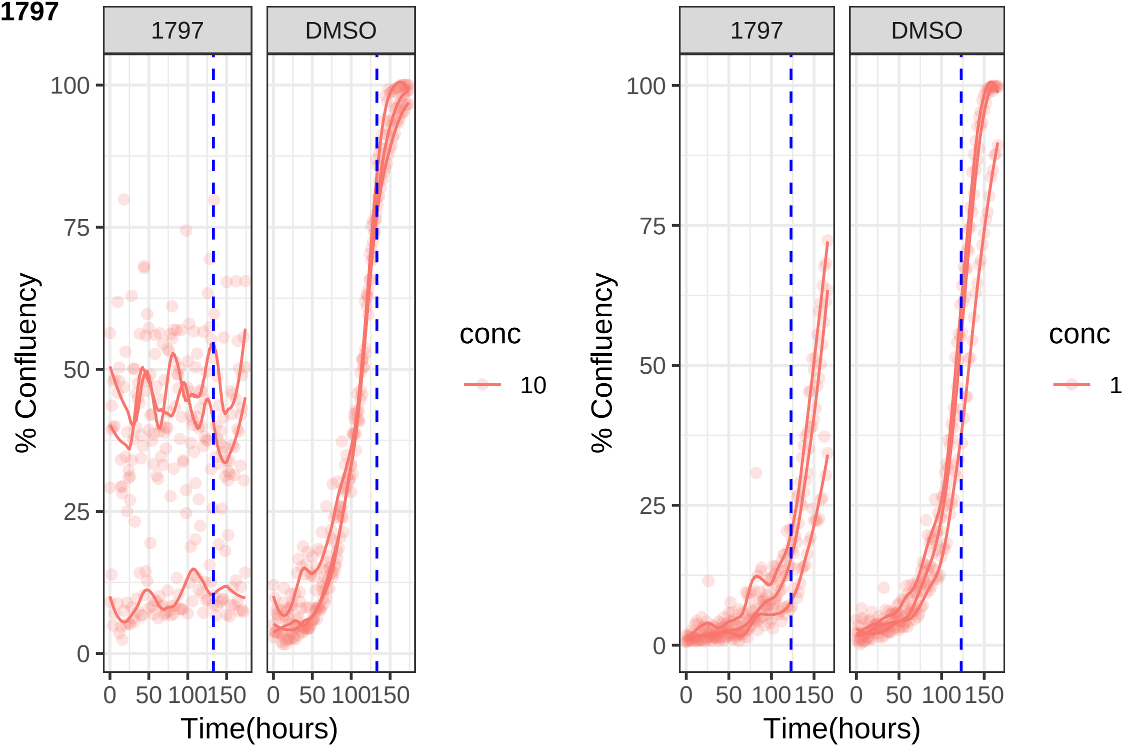

1797
DMSO
% Confluency
100
75
50
25
0
0
50
100
150
Time(hours)
conc
10
1797
DMSO
% Confluency
Time(hours)
conc
1

1801

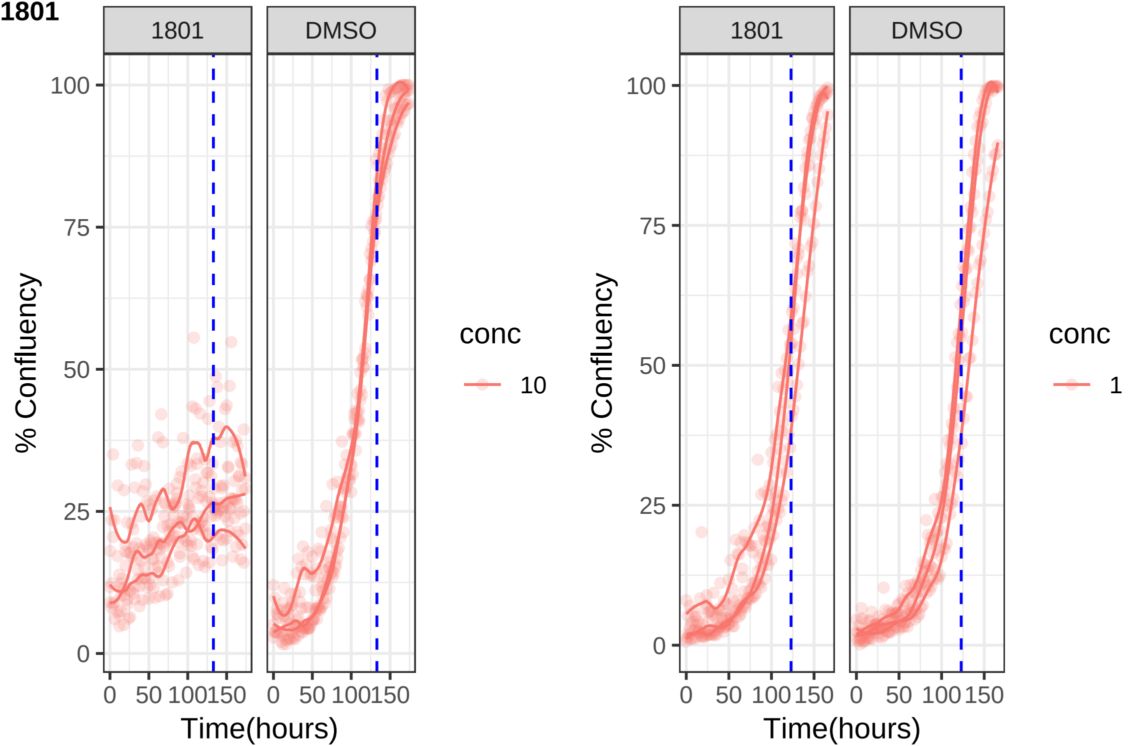

1801
DMSO
100
75
50
25
0
0
50
100
150
% Confluency
Time(hours)
conc
10
1

1803

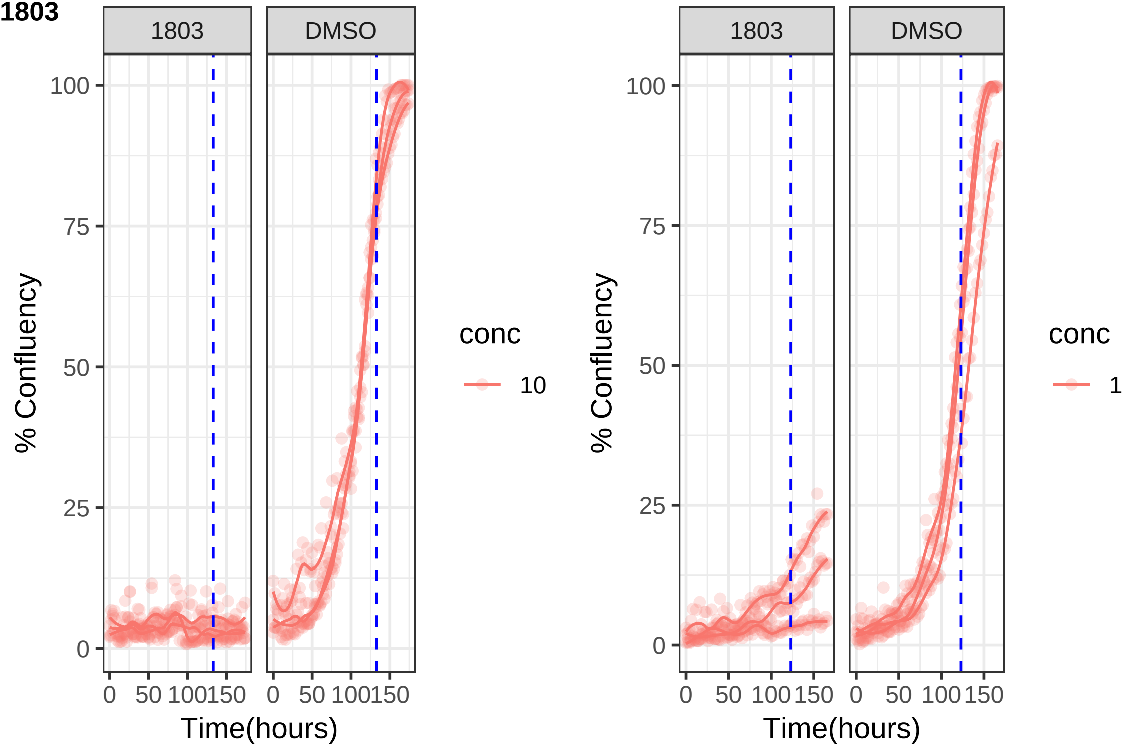
1803
DMSO
% Confluency
100
75
50
25
0
0
50
100
150
Time(hours)
conc
10
1803
DMSO
% Confluency
100
75
50
25
0
0
50
100
150
Time(hours)
conc
1

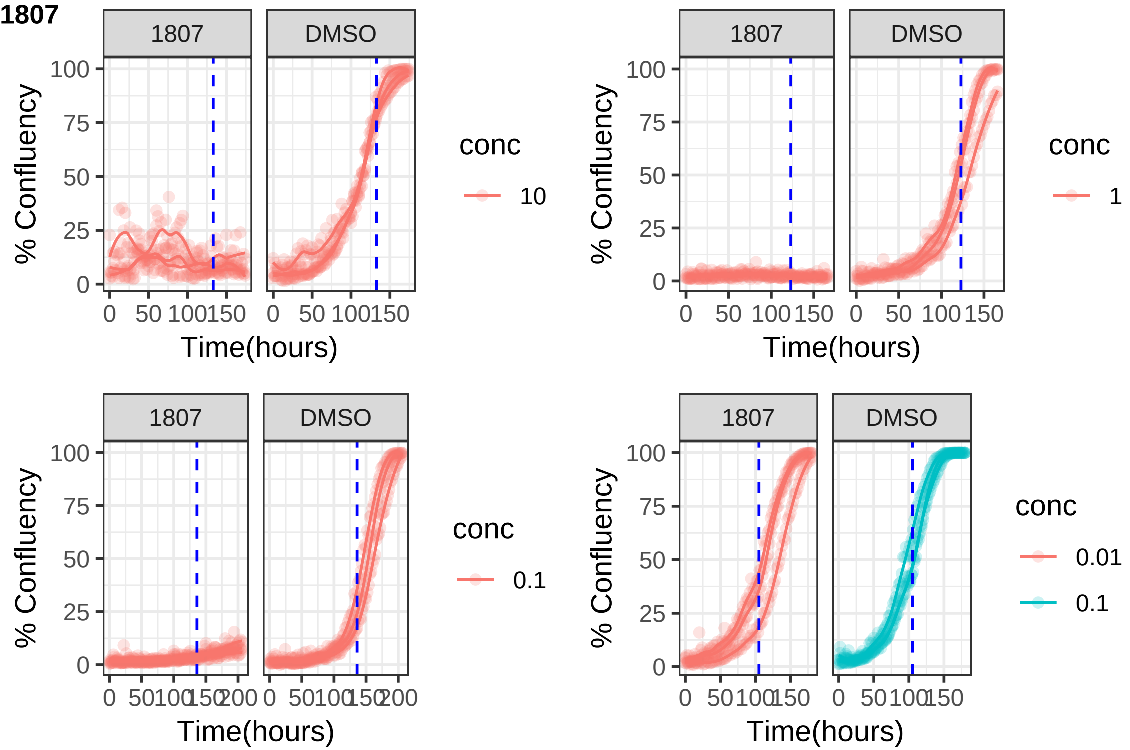

1807
1807
DMSO
% Confluency
Time(hours)
conc
10
1
0.1
0.01
0.1
100
75
50
25
0
0
50
100
150
200

1814

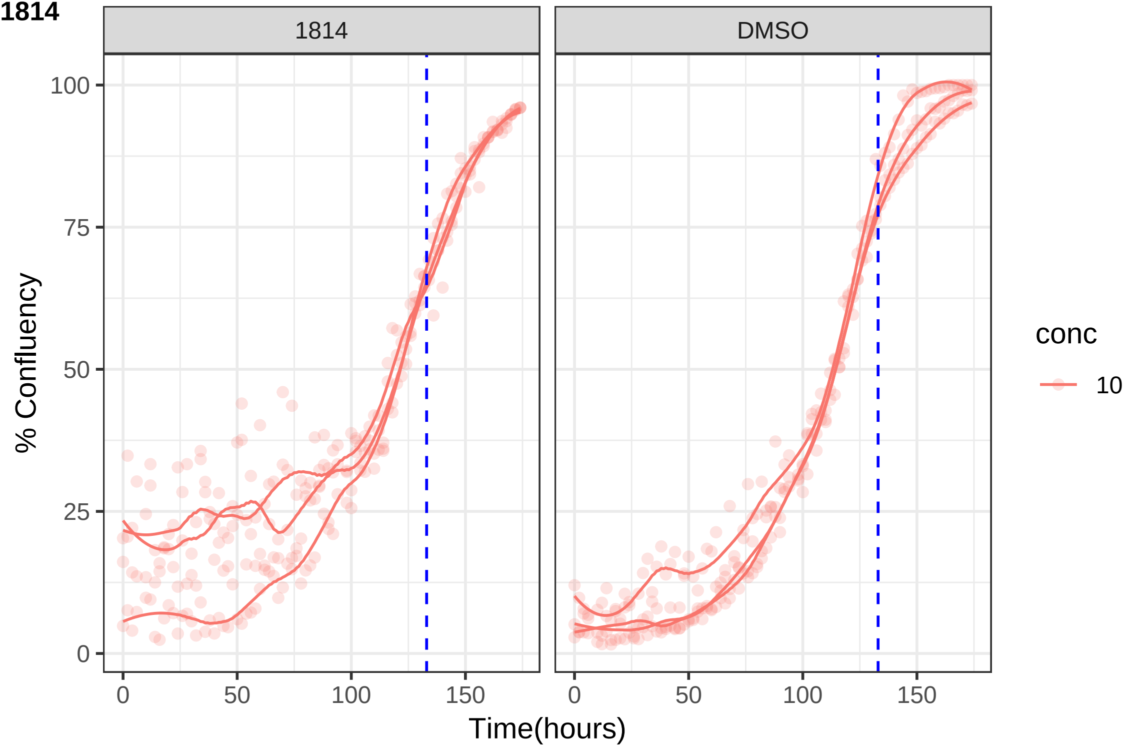
1814
DMSO
% Confluency
0
25
50
75
100
Time(hours)
0
50
100
150
conc
10

1831

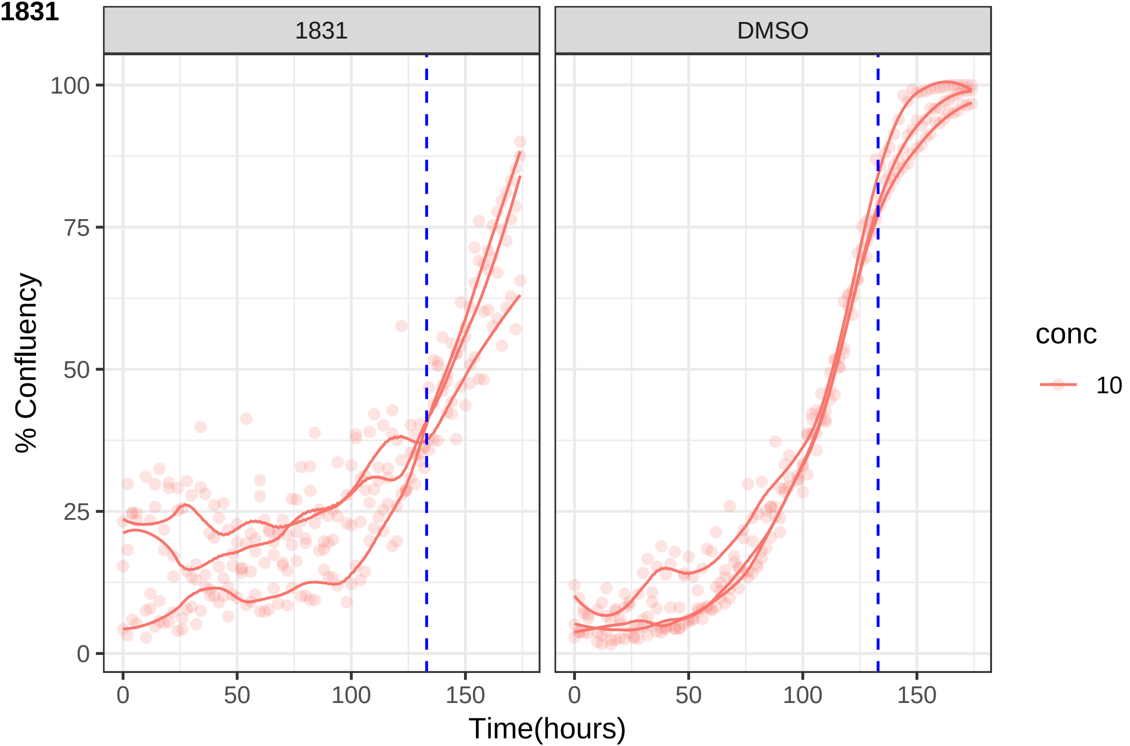

1831
DMSO
% Confluency
100
75
50
25
0
0
50
100
150
Time(hours)
conc
10

1853

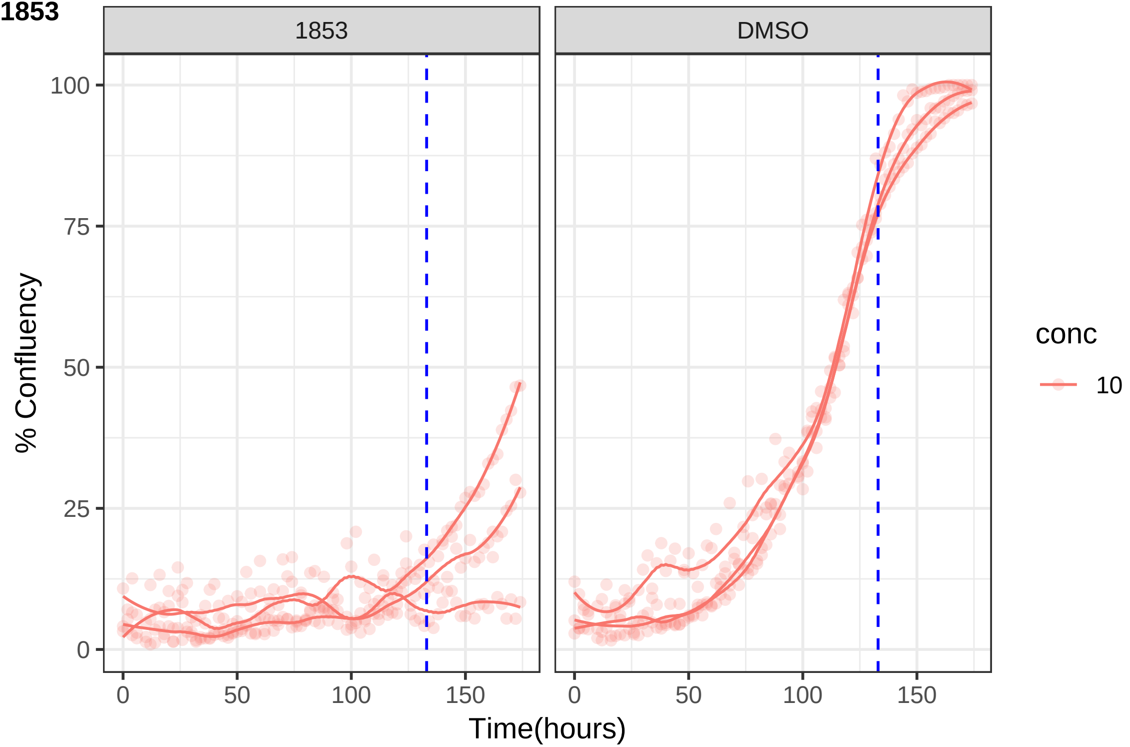
1853
DMSO
% Confluency
Time(hours)
100
75
50
25
0
0
50
100
150
conc
10

1895

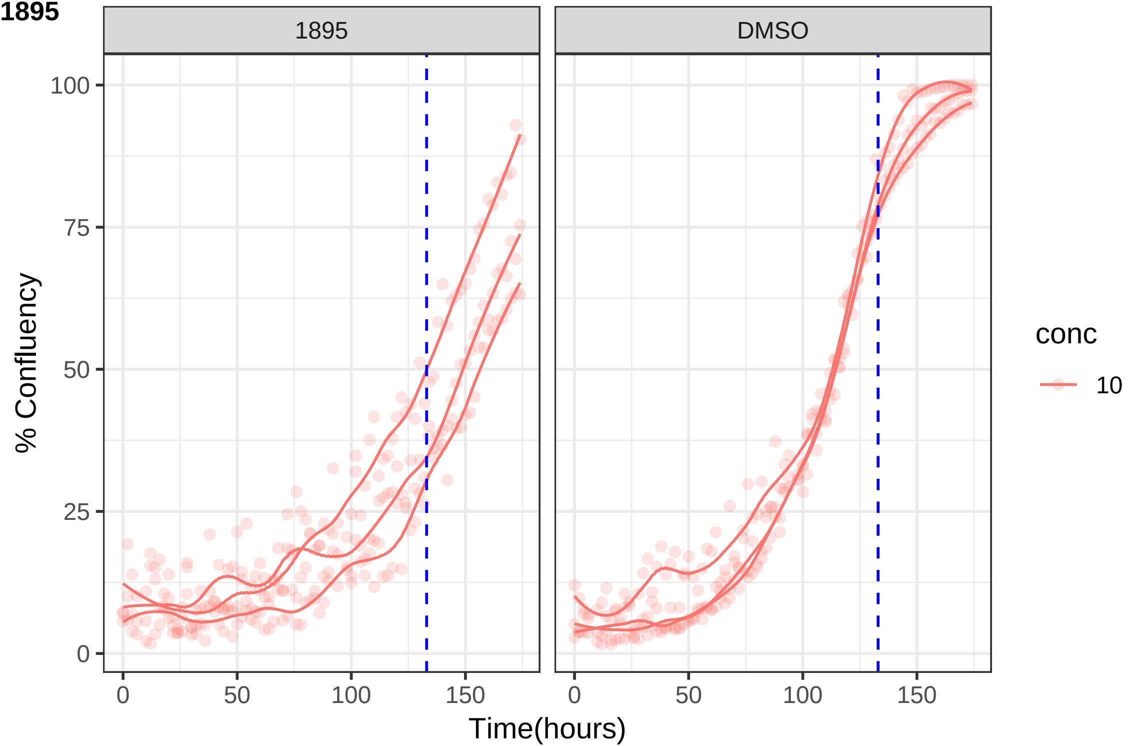
1895
DMSO
% Confluency
0
25
50
75
100
0
50
100
150
Time(hours)
conc
10

1928

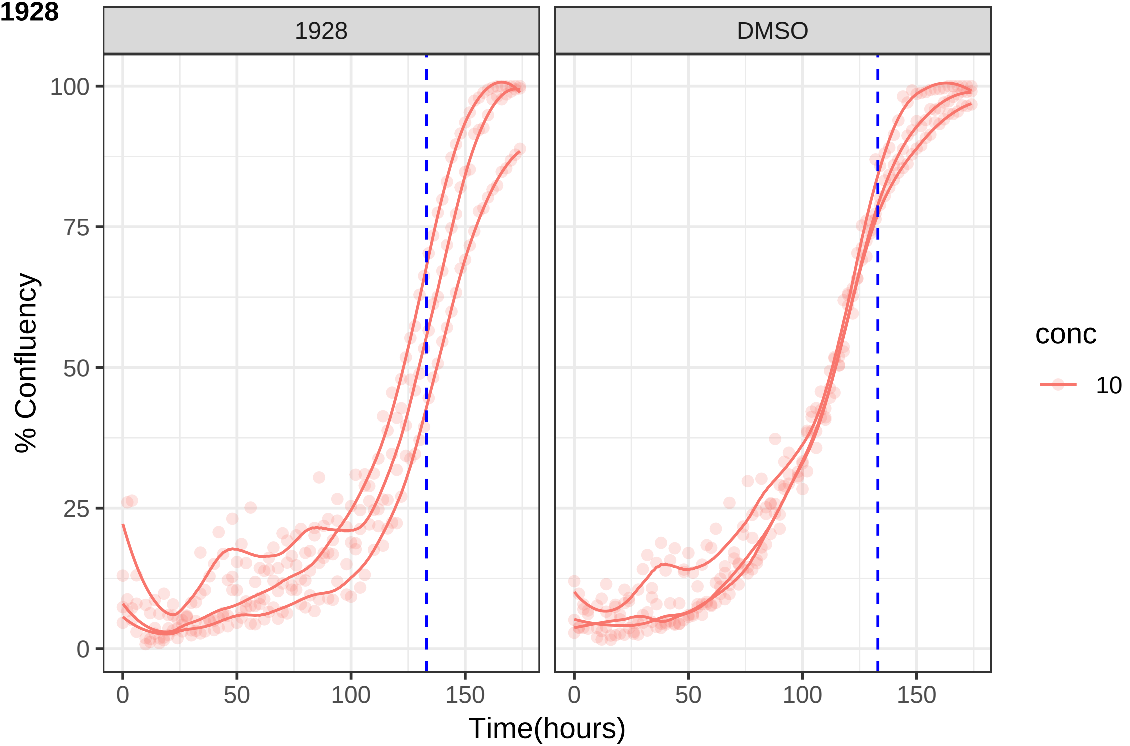
1928
DMSO
100
75
50
25
0
% Confluency
0
50
100
150
Time(hours)
conc
10

1999

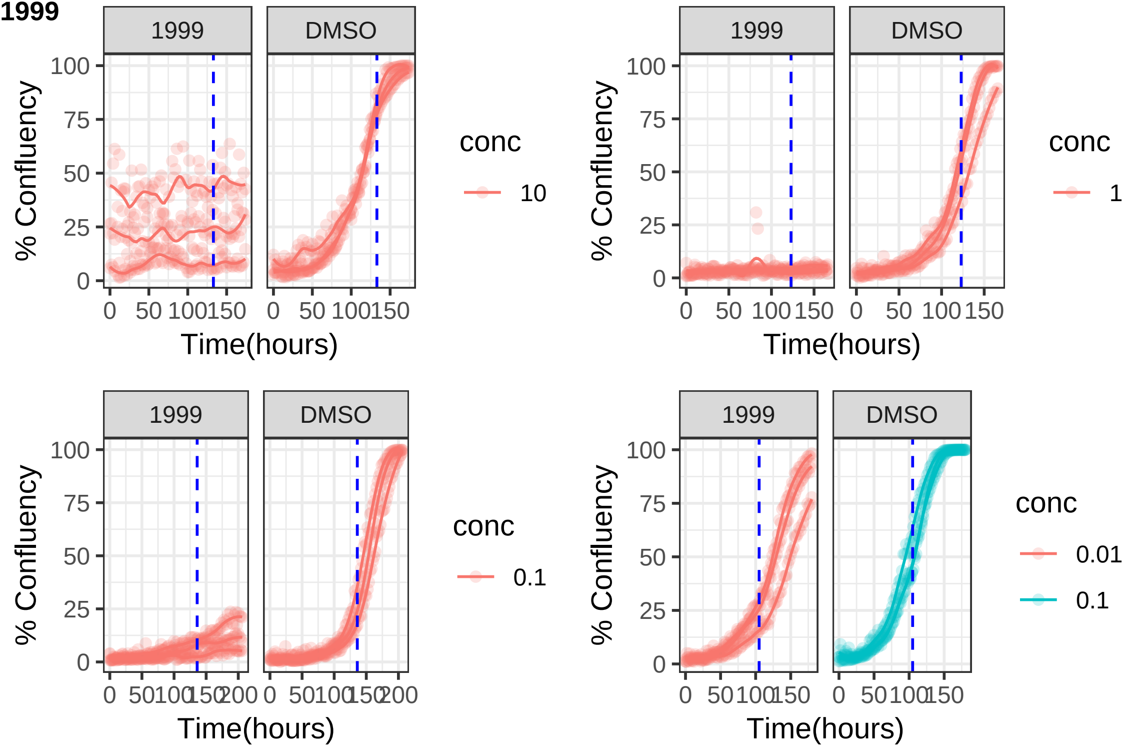

2008

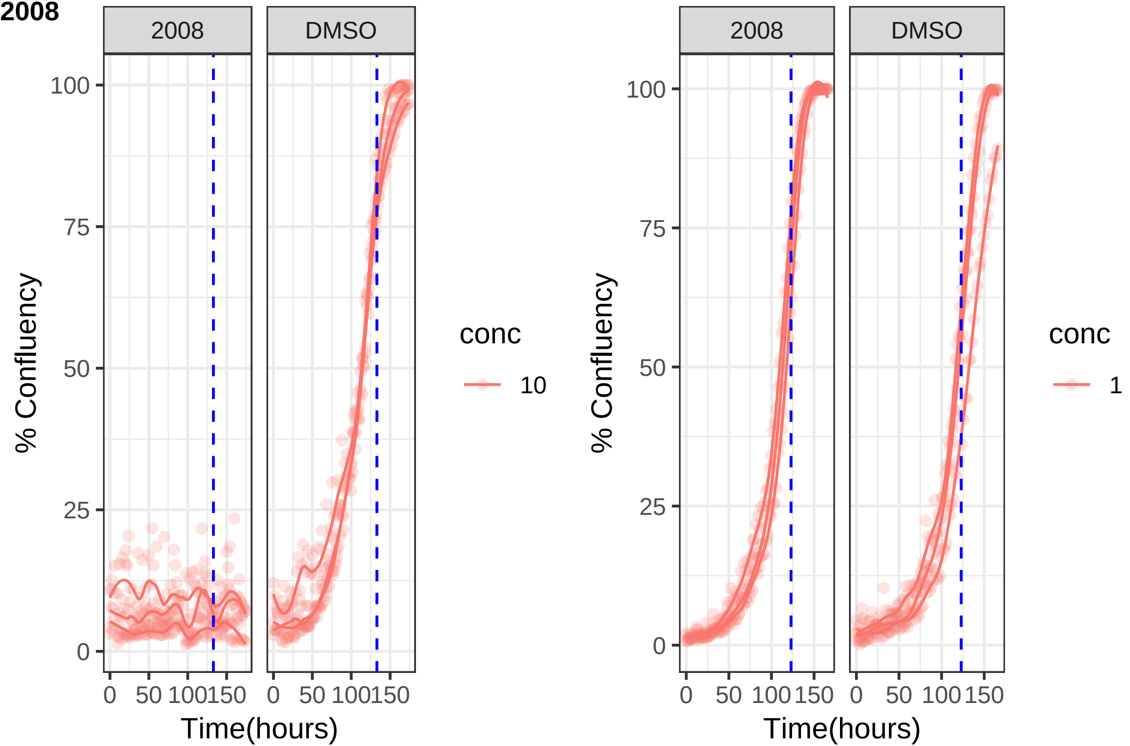

2008
DMSO
100
75
50
25
0
% Confluency
0
50
100
150
Time(hours)
conc
10
2008
DMSO
100
75
50
25
0
% Confluency
0
50
100
150
Time(hours)
conc
1

2014

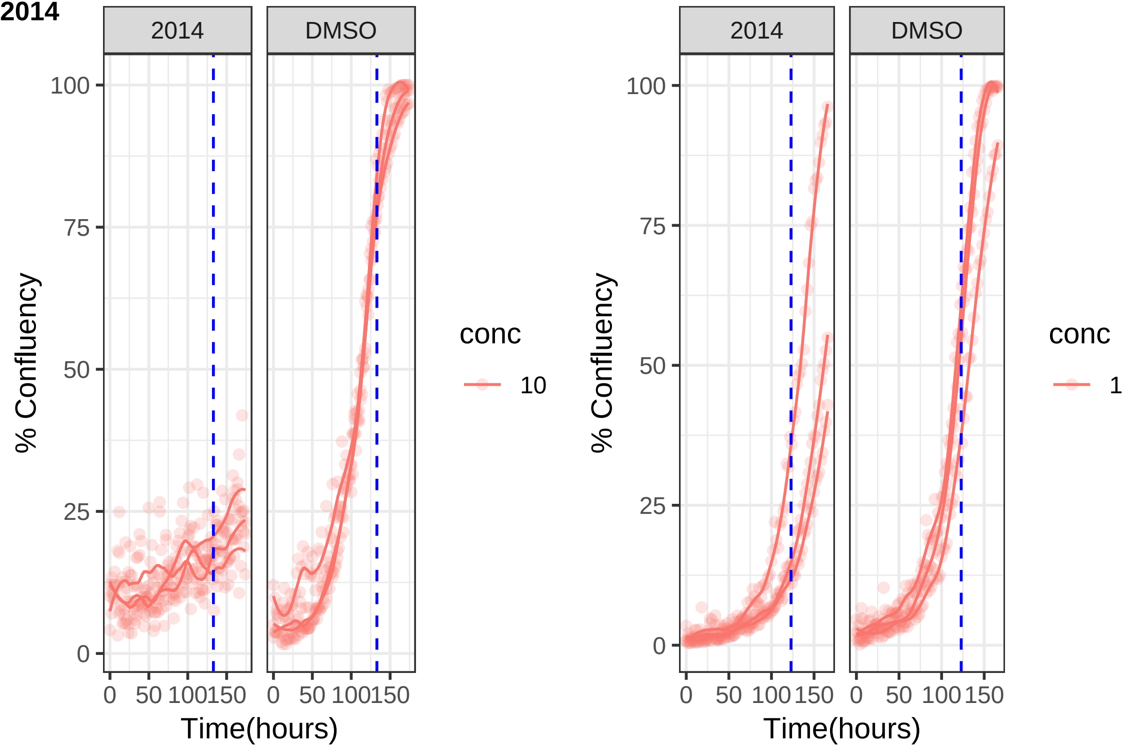

2014
DMSO
100
75
50
25
0
% Confluency
0
50
100
150
Time(hours)
conc
10
2014
DMSO
100
75
50
25
0
% Confluency
0
50
100
150
Time(hours)
conc
1

2084

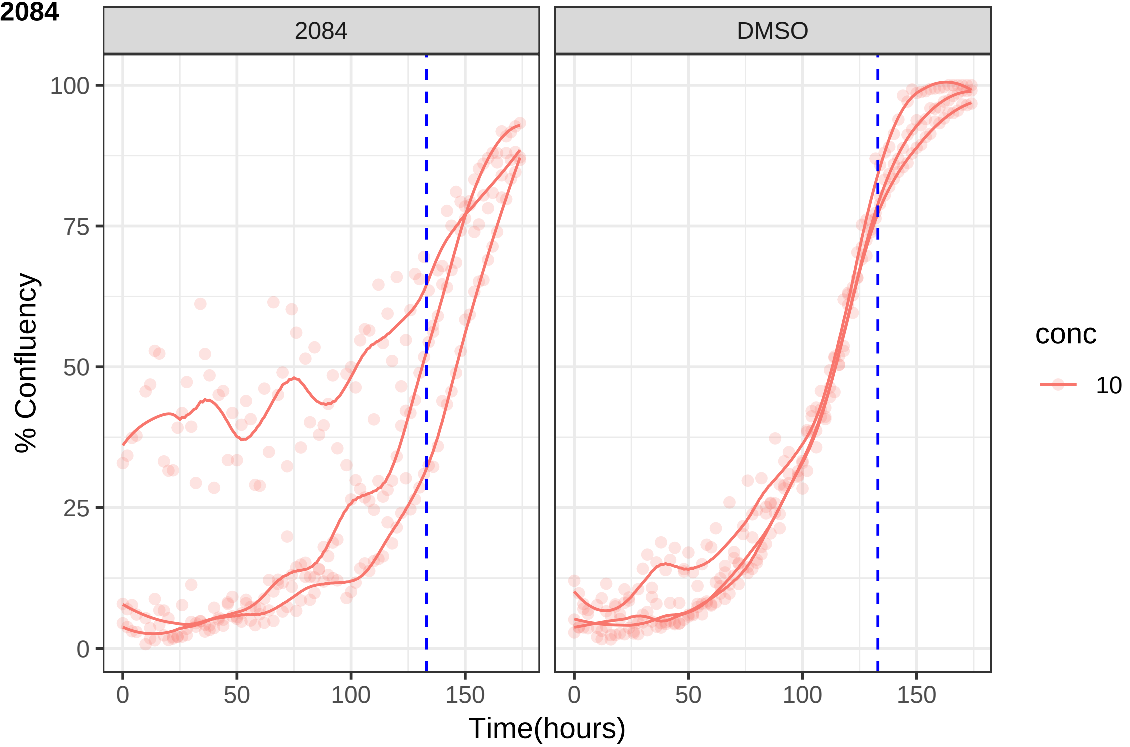
2084
DMSO
100
75
50
25
0
% Confluency
0
50
100
150
Time(hours)
conc
10

2100

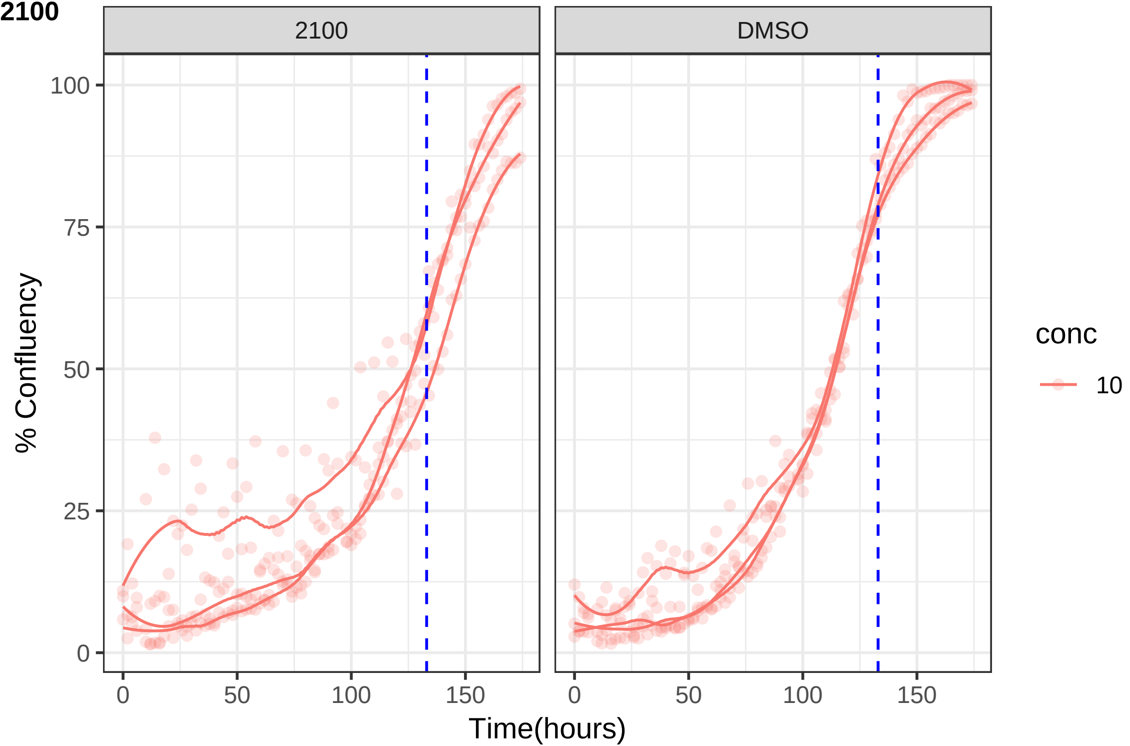

2100
DMSO
% Confluency
100
75
50
25
0
0
50
100
150
Time(hours)
conc
10

2114

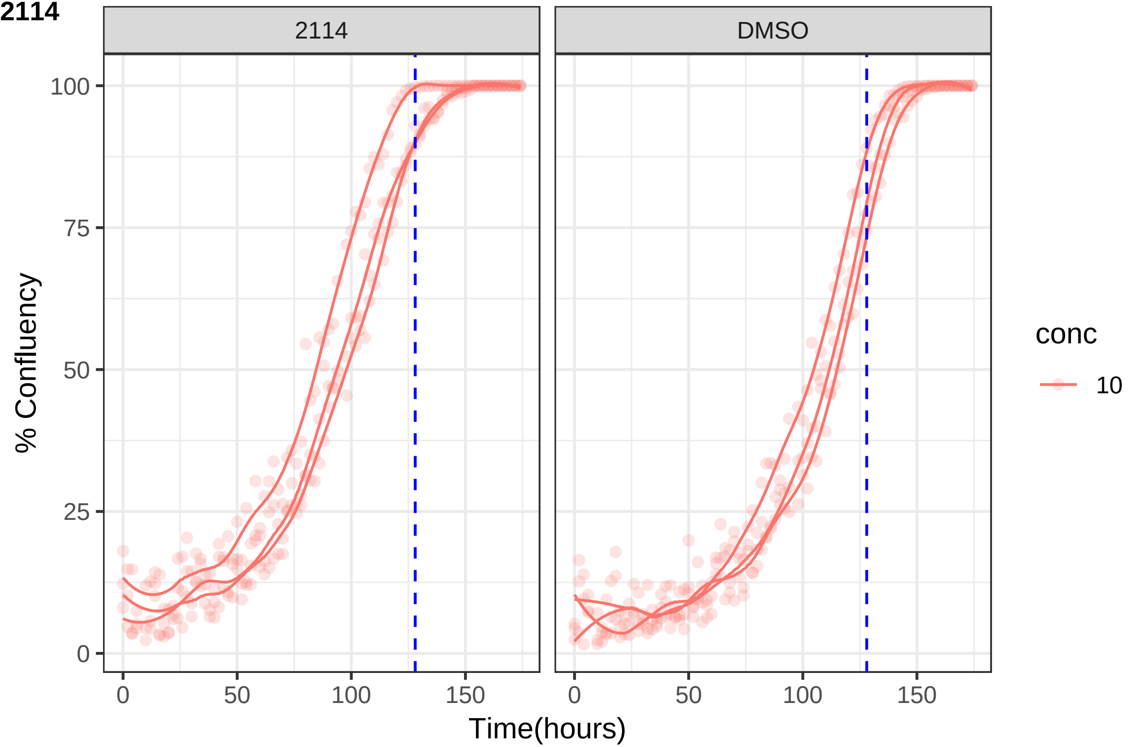
2114
DMSO
100
75
50
25
0
% Confluency
0
50
100
150
Time(hours)
conc
10

2120

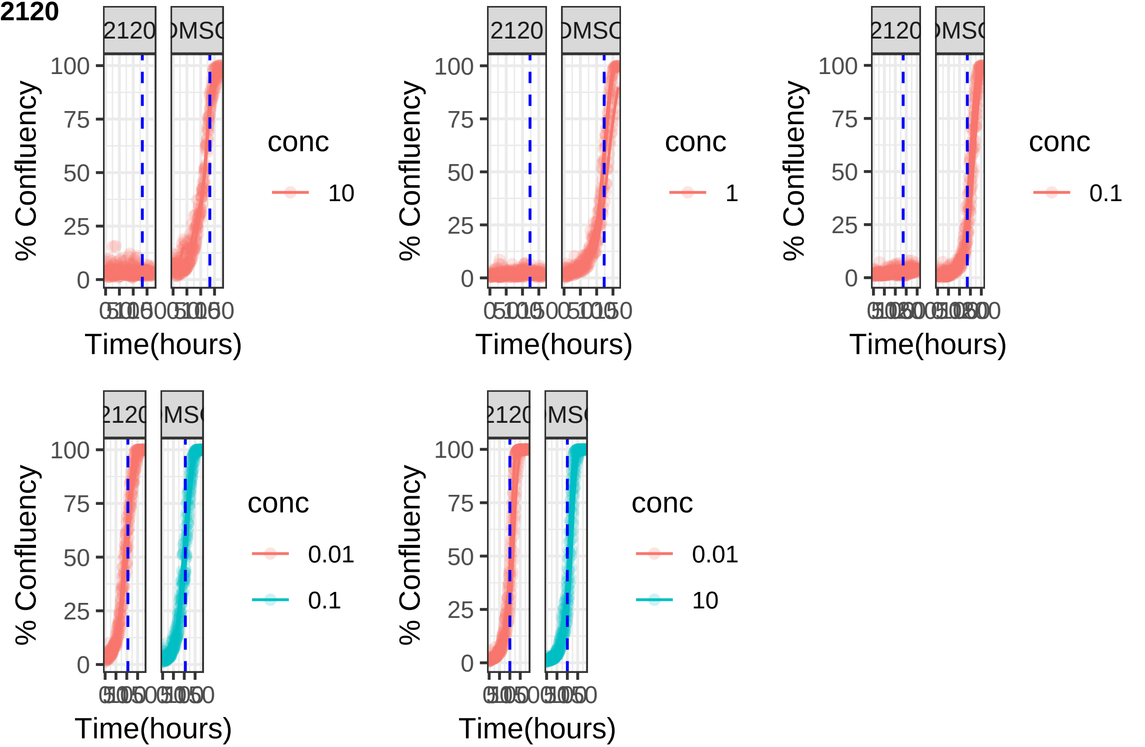

2138

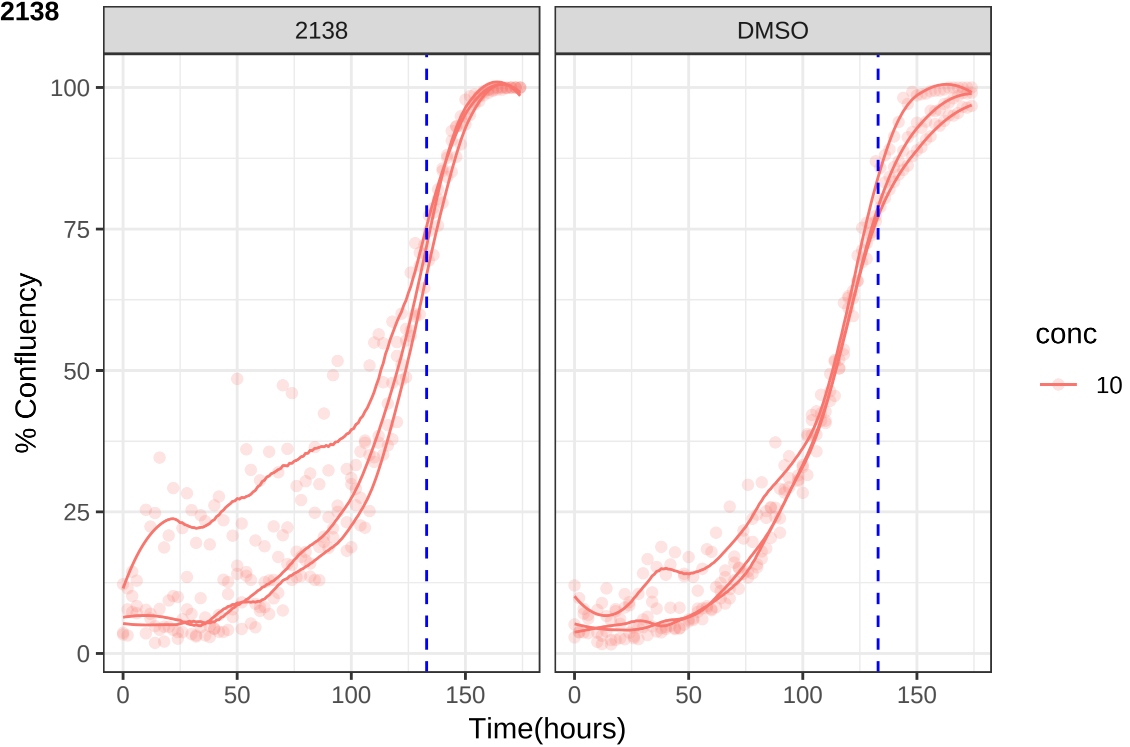
2138
DMSO
% Confluency
100
75
50
25
0
0
50
100
150
Time(hours)
conc
10

2140

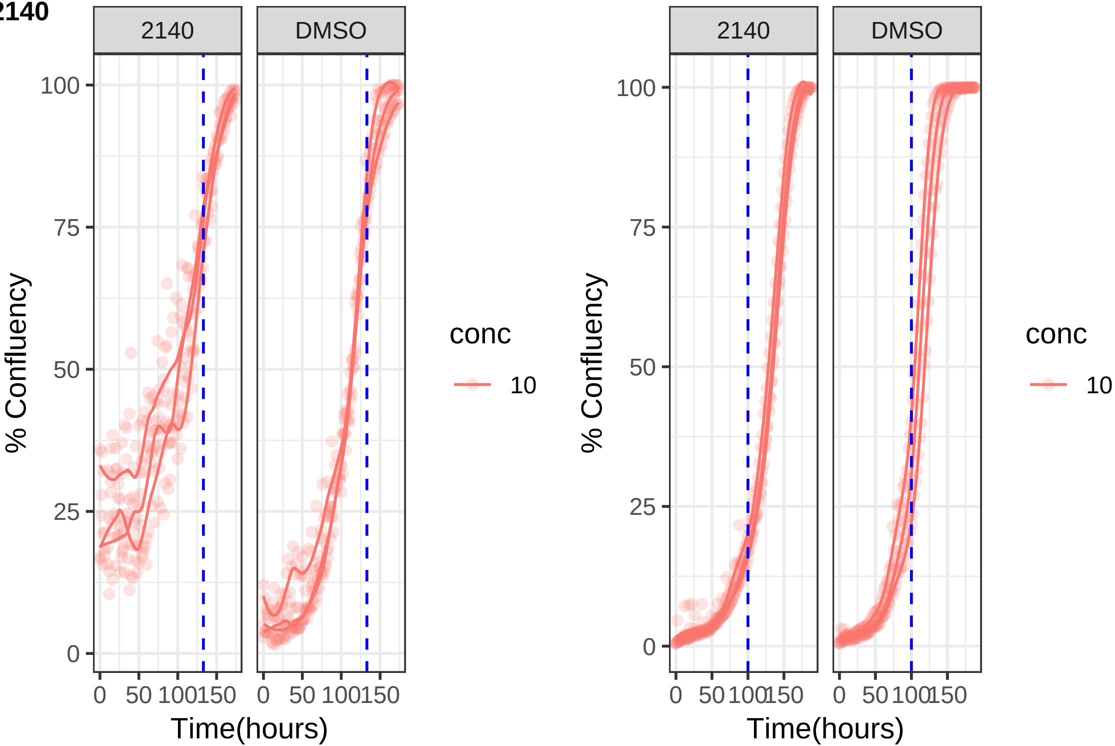
2140
DMSO
% Confluency
0
25
50
75
100
0
50
100
150
Time(hours)
conc
10
2140
DMSO
% Confluency
0
25
50
75
100
0
50
100
150
Time(hours)
conc
10

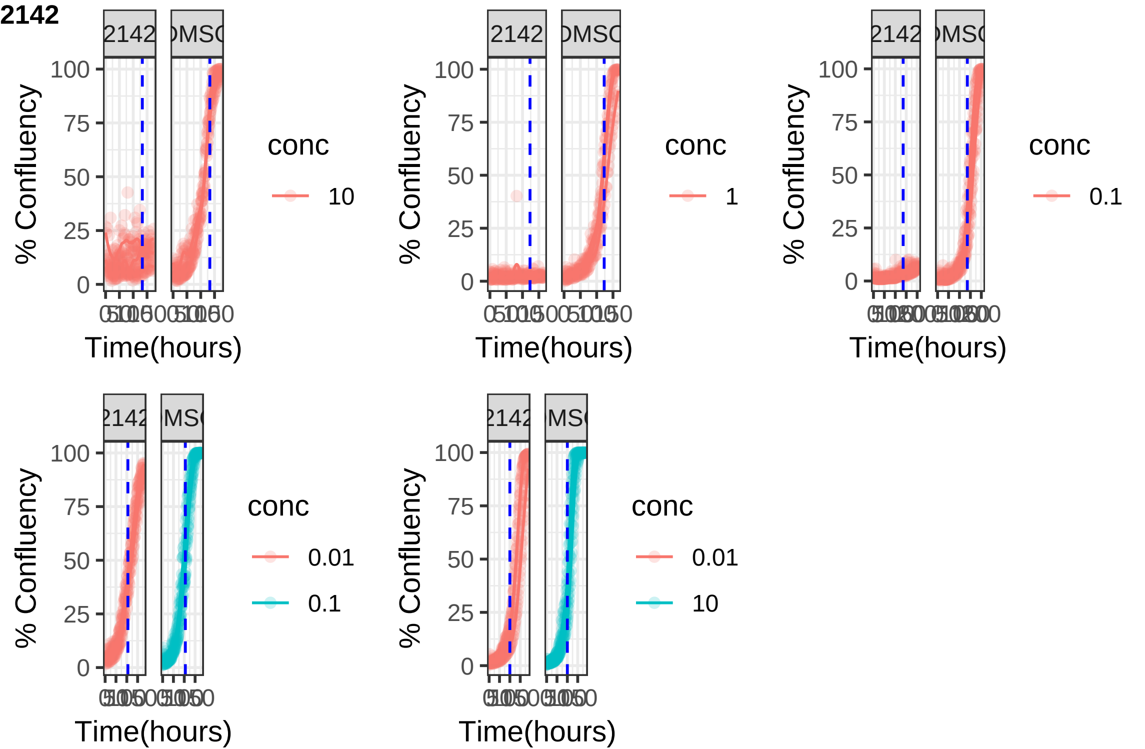
2142
% Confluency
Time(hours)
conc
10
1
0.1
0.01
0.1
0.01
10
0
25
50
75
100

2158

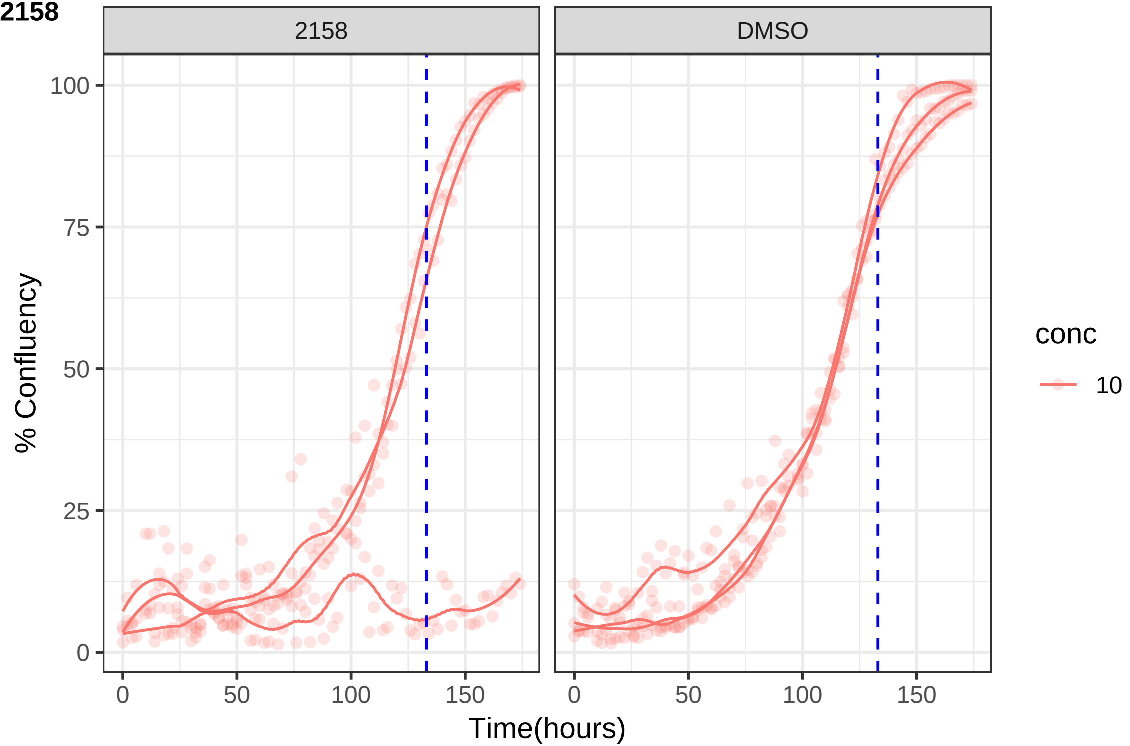

2158
DMSO
% Confluency
100
75
50
25
0
0
50
100
150
Time(hours)
conc
10

2159

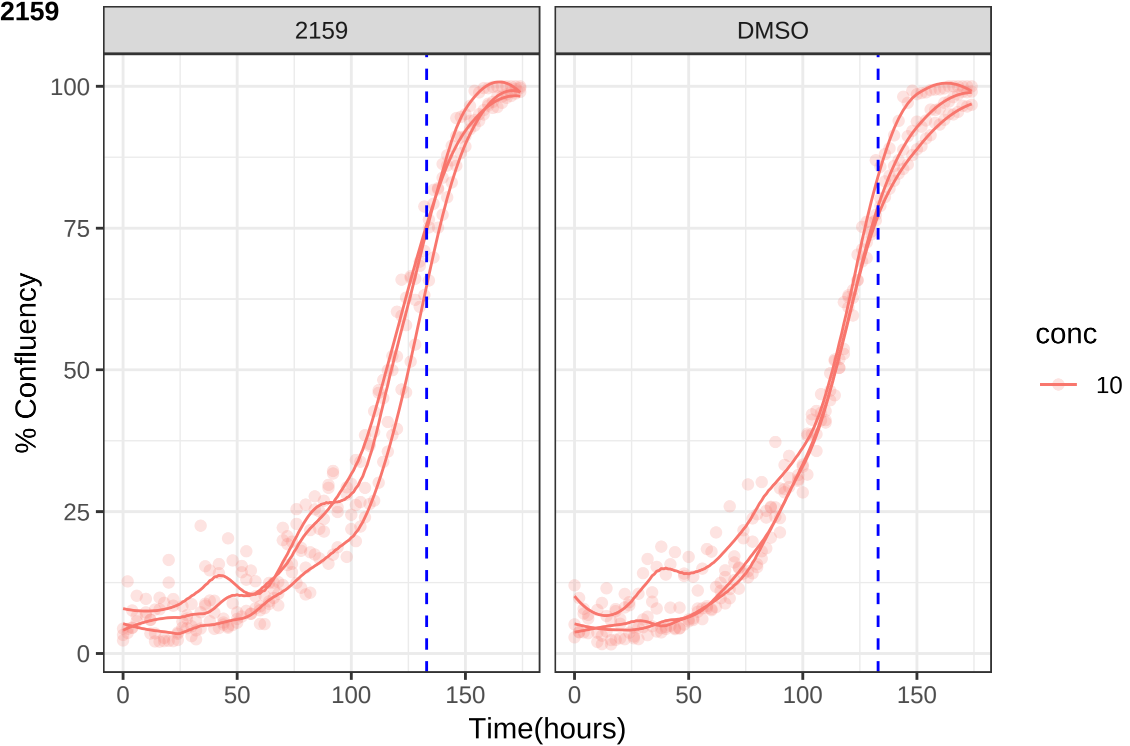
2159
DMSO
100
75
50
25
0
% Confluency
0
50
100
150
Time(hours)
conc
10

2160

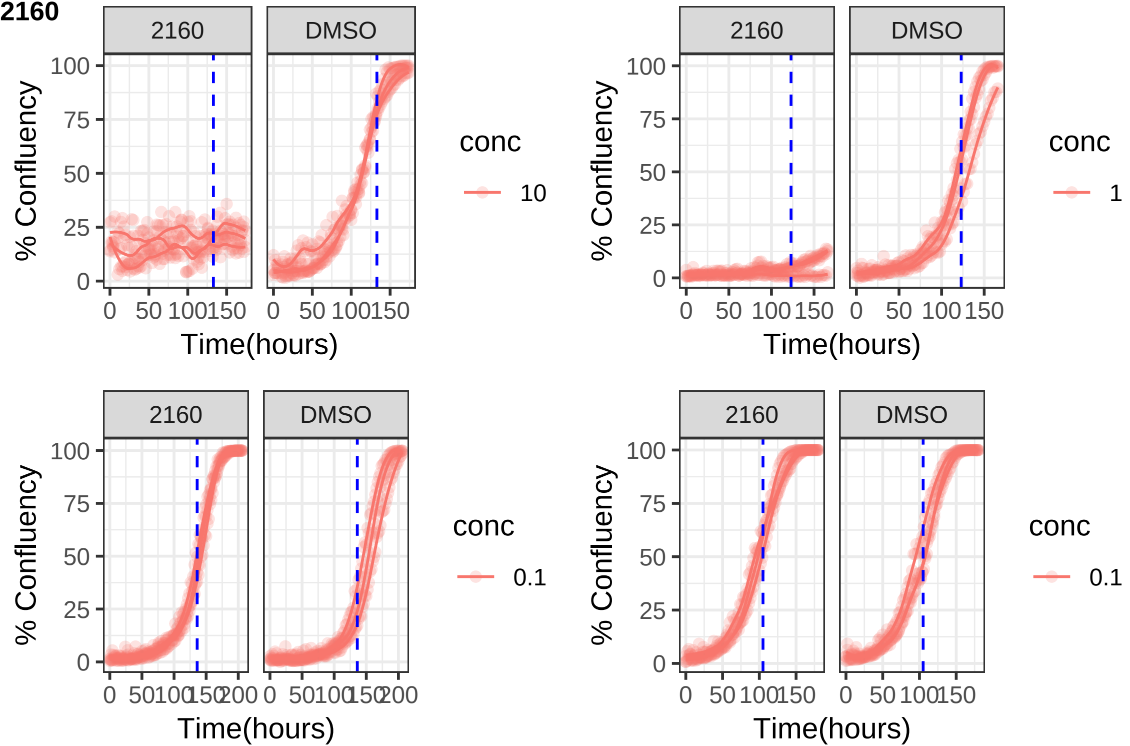

2160
DMSO
% Confluency
Time(hours)
conc
10
1
0.1
0
25
50
75
100
0
50
100
150
200

2165

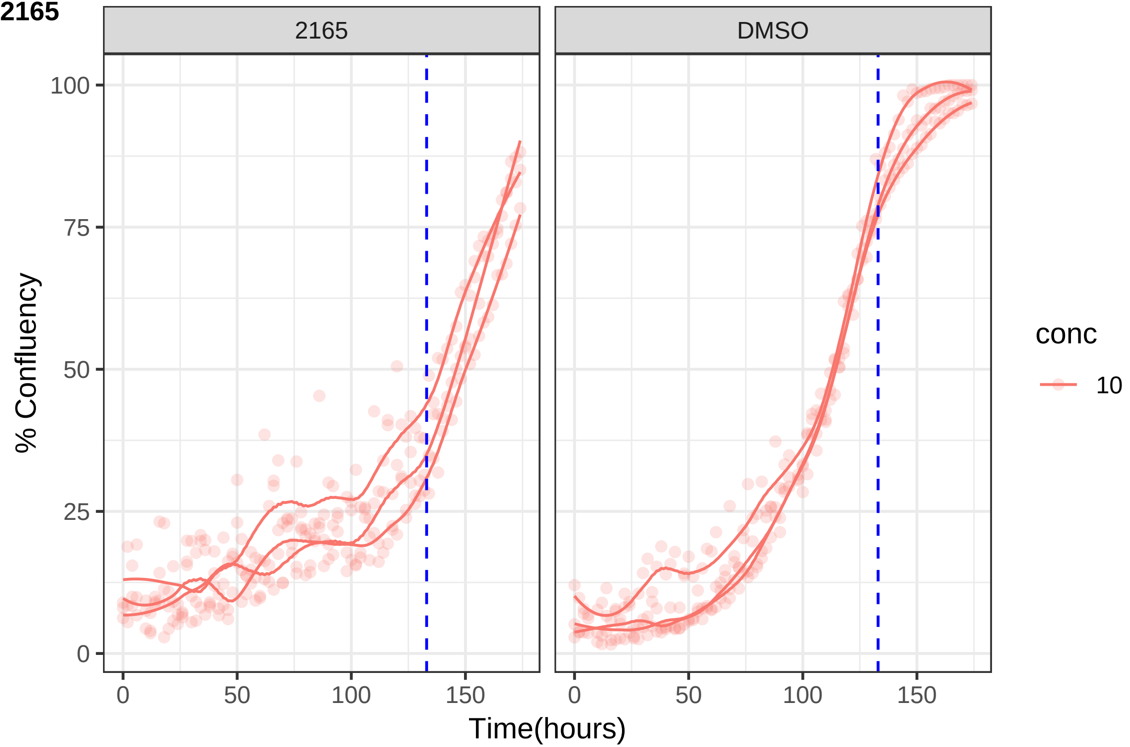

2165
DMSO
% Confluency
100
75
50
25
0
0
50
100
150
Time(hours)
conc
10

2168

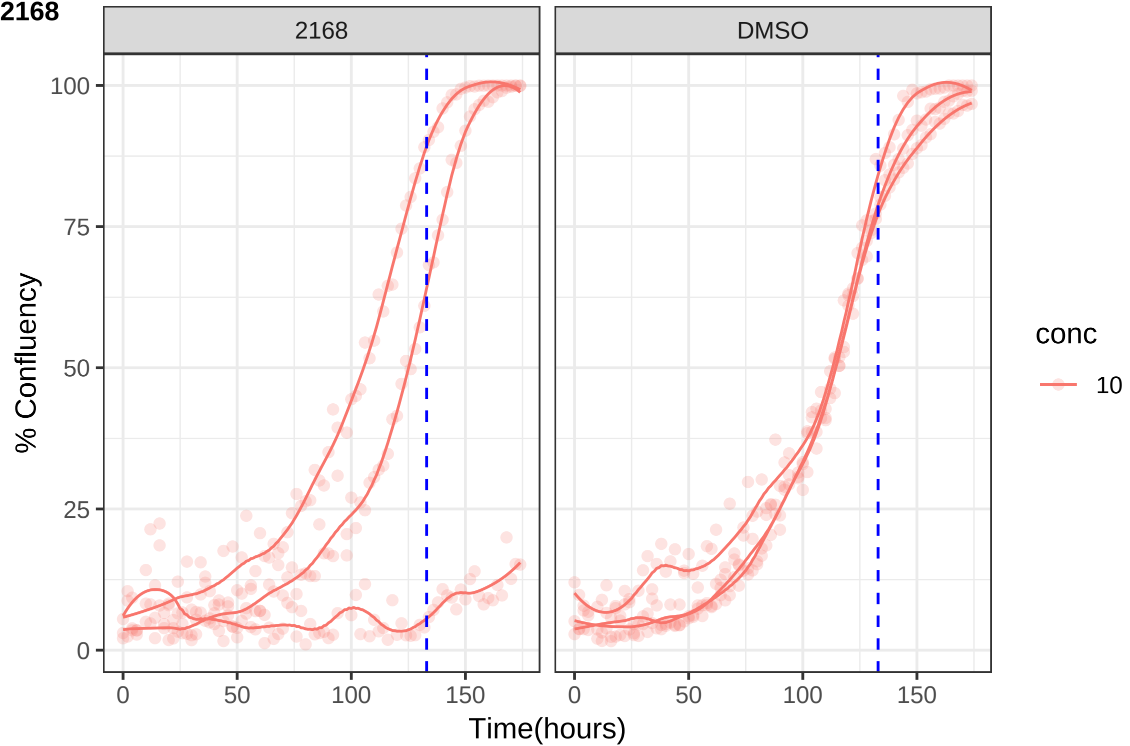

2168
DMSO
100
75
50
25
0
% Confluency
0
50
100
150
Time(hours)
conc
10

2170

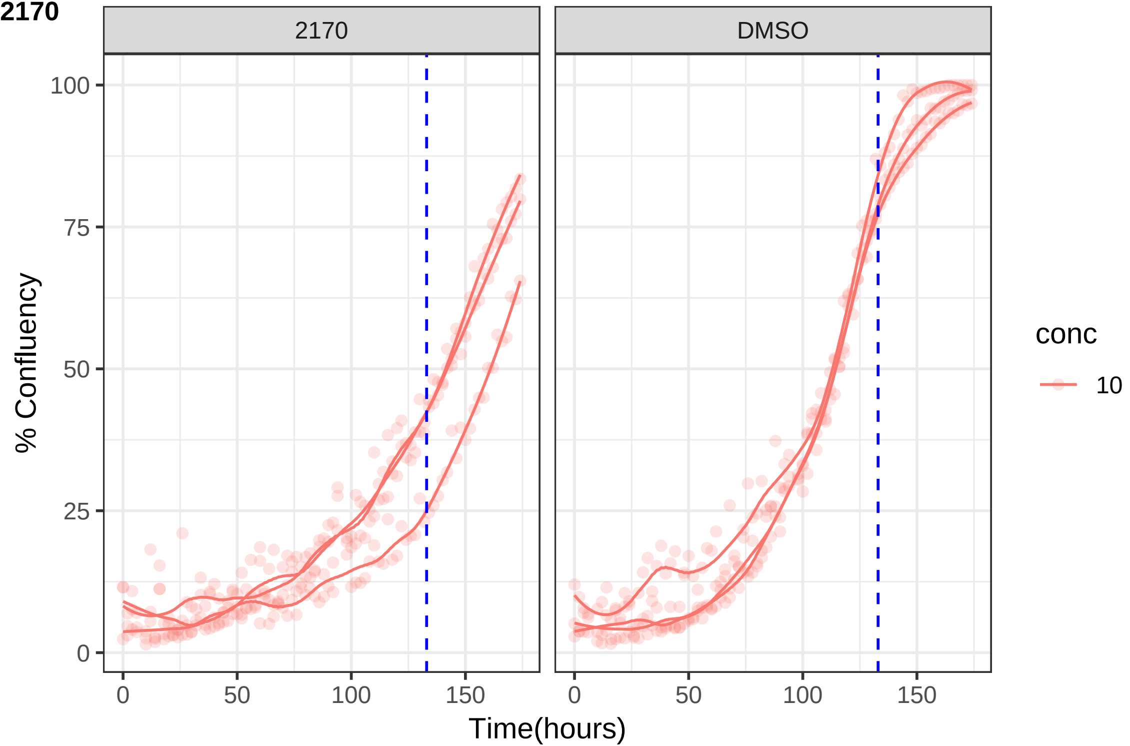
2170
DMSO
% Confluency
Time(hours)
0
25
50
75
100
0
50
100
150
conc
10

2180

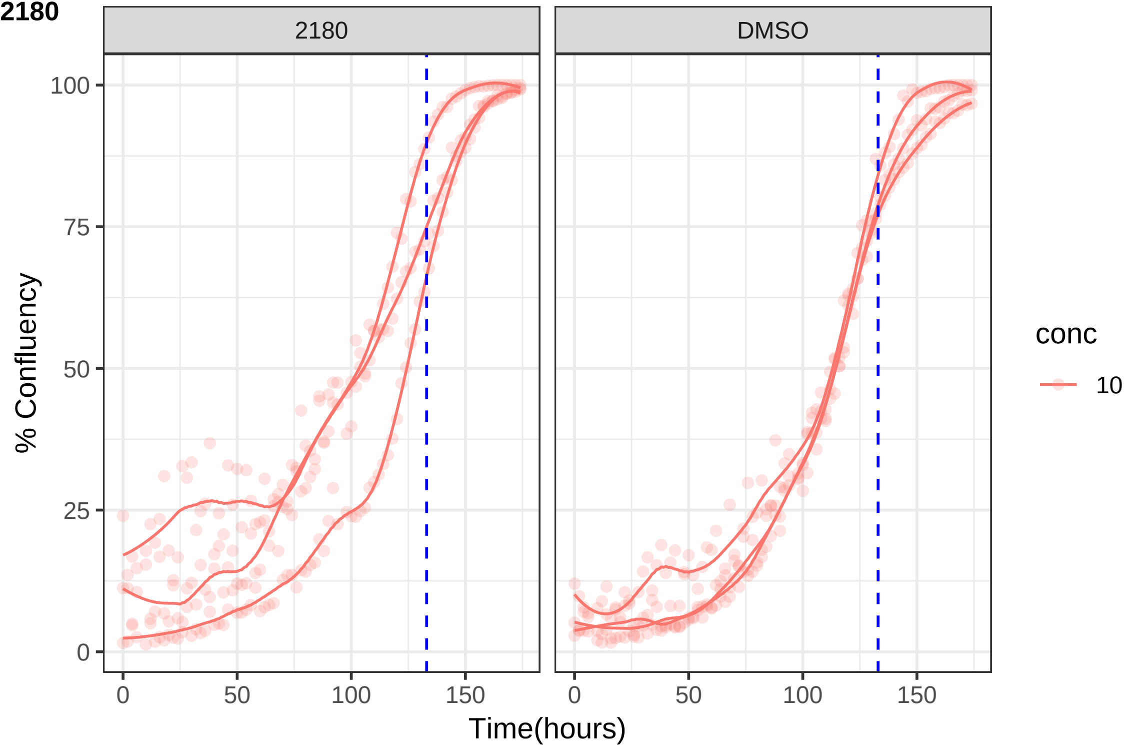

2180
DMSO
% Confluency
100
75
50
25
0
0
50
100
150
0
50
100
150
Time(hours)
conc
10

2185

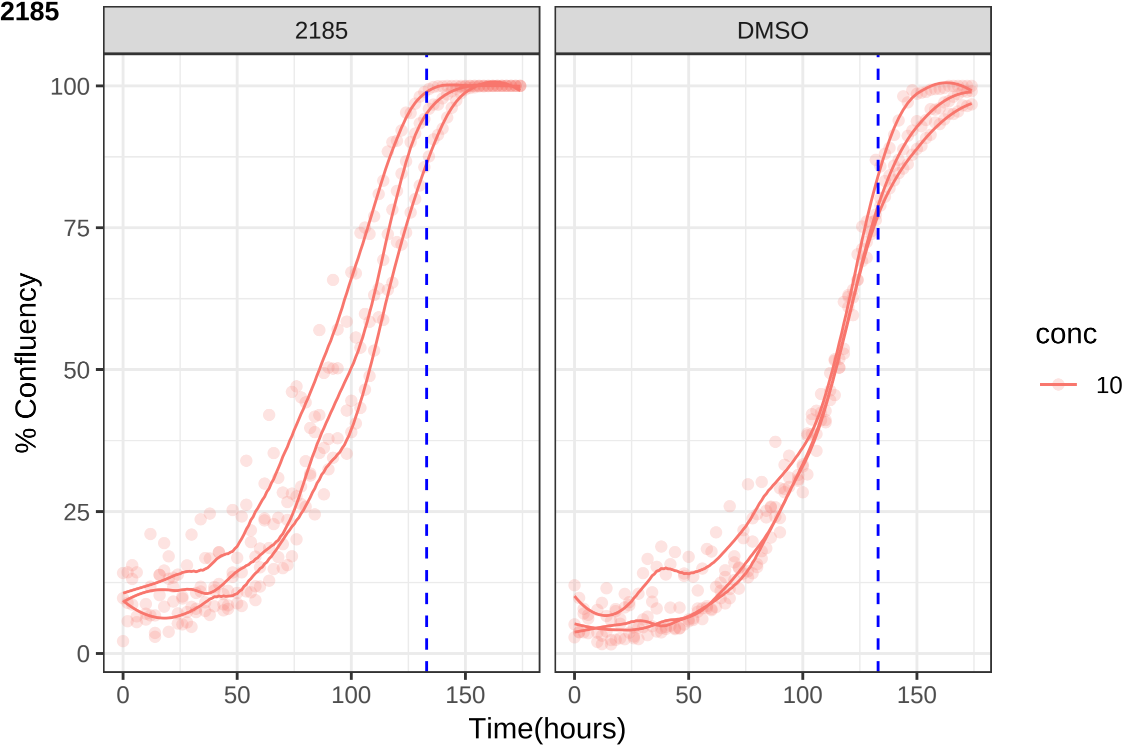
2185
DMSO
100
75
50
25
0
% Confluency
0
50
100
150
Time(hours)
conc
10

2188

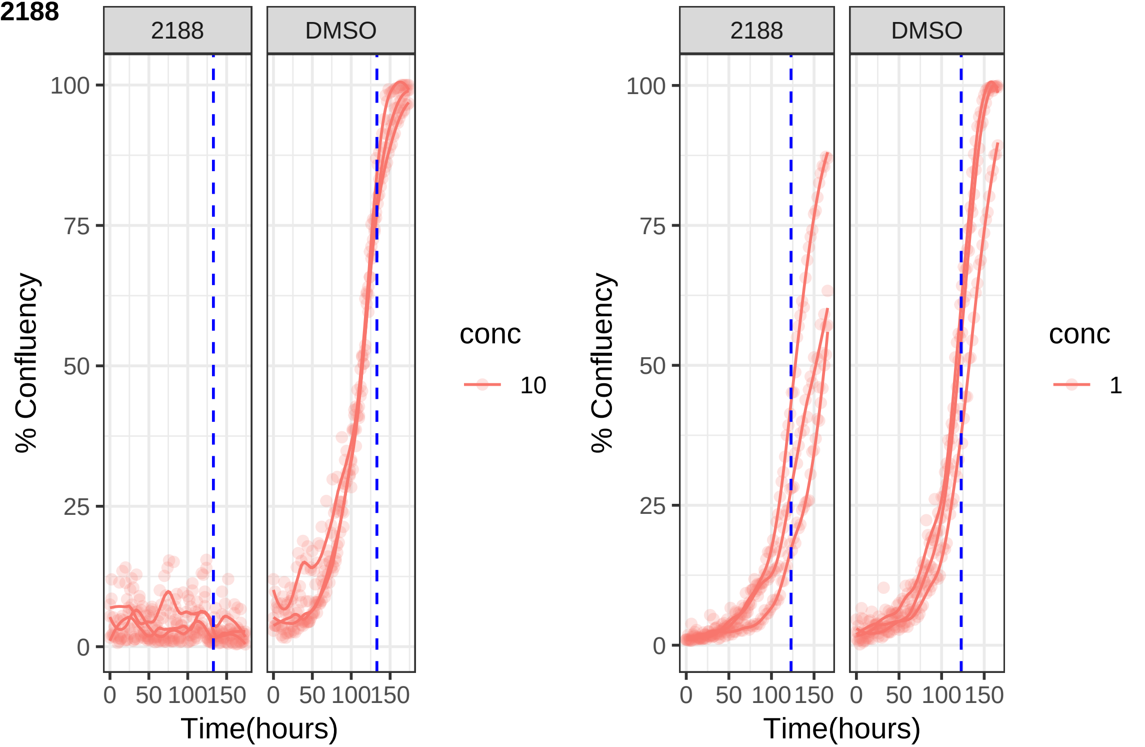

2188
DMSO
100
75
50
25
0
% Confluency
0
50
100
150
Time(hours)
conc
10
2188
DMSO
100
75
50
25
0
% Confluency
0
50
100
150
Time(hours)
conc
1

2195

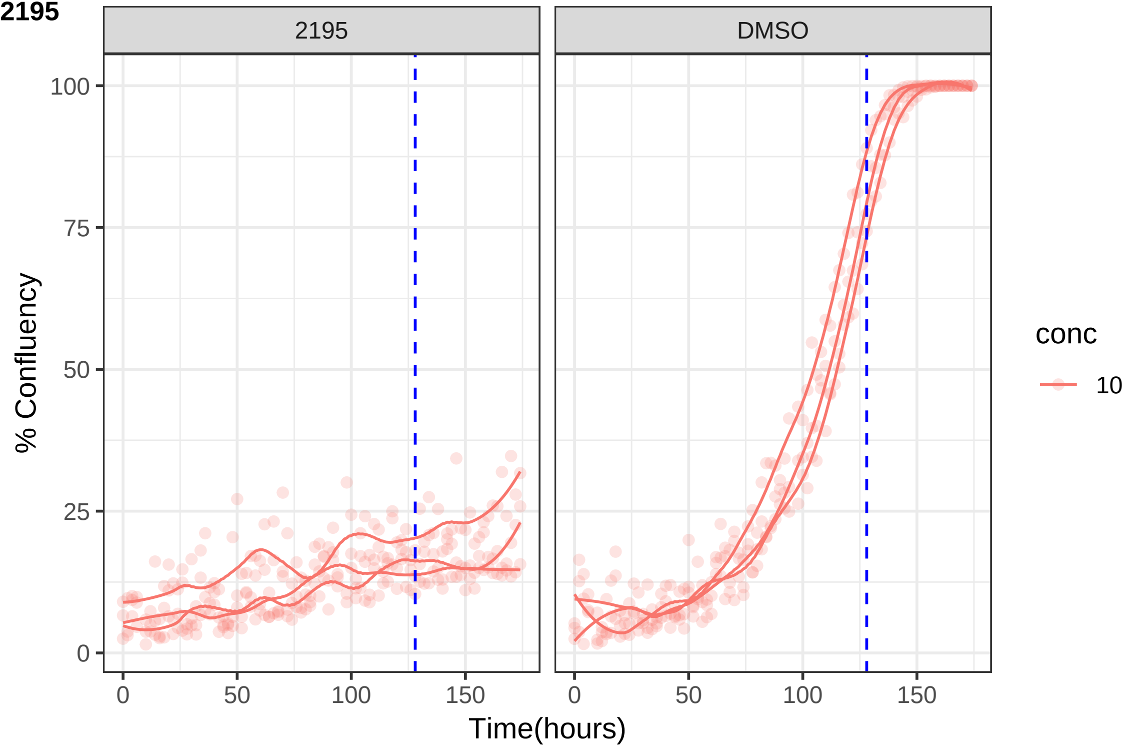

2195
DMSO
% Confluency
0
25
50
75
100
0
50
100
150
Time(hours)
conc
10

2198

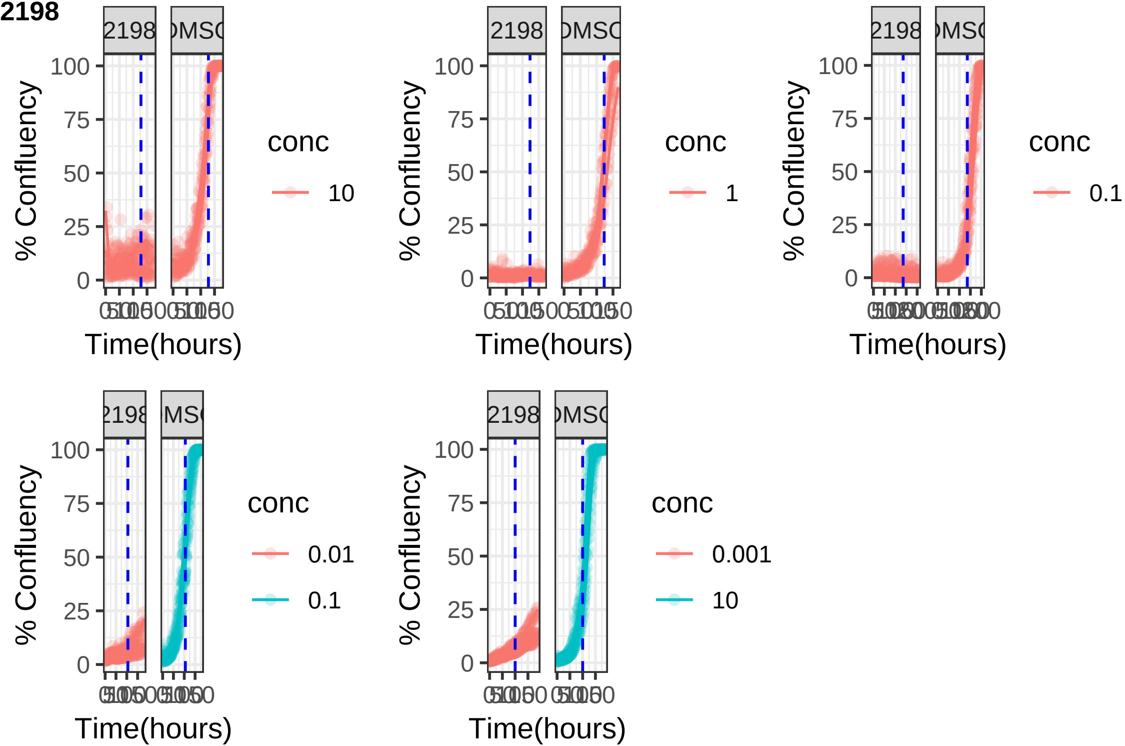
2198
DMSO
% Confluency
Time(hours)
conc
10
1
0.1
0.01
0.1
0.001
10
100
75
50
25
0

2209

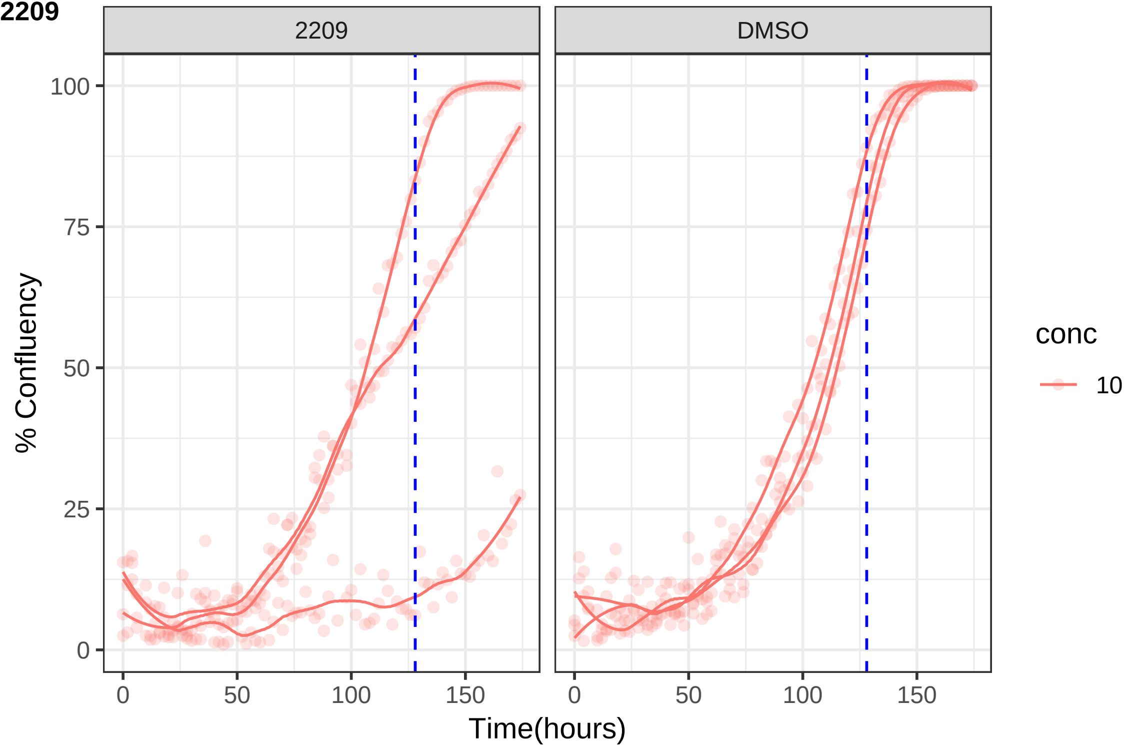
2209
DMSO
100
75
50
25
0
% Confluency
0
50
100
150
Time(hours)
conc
10

2210

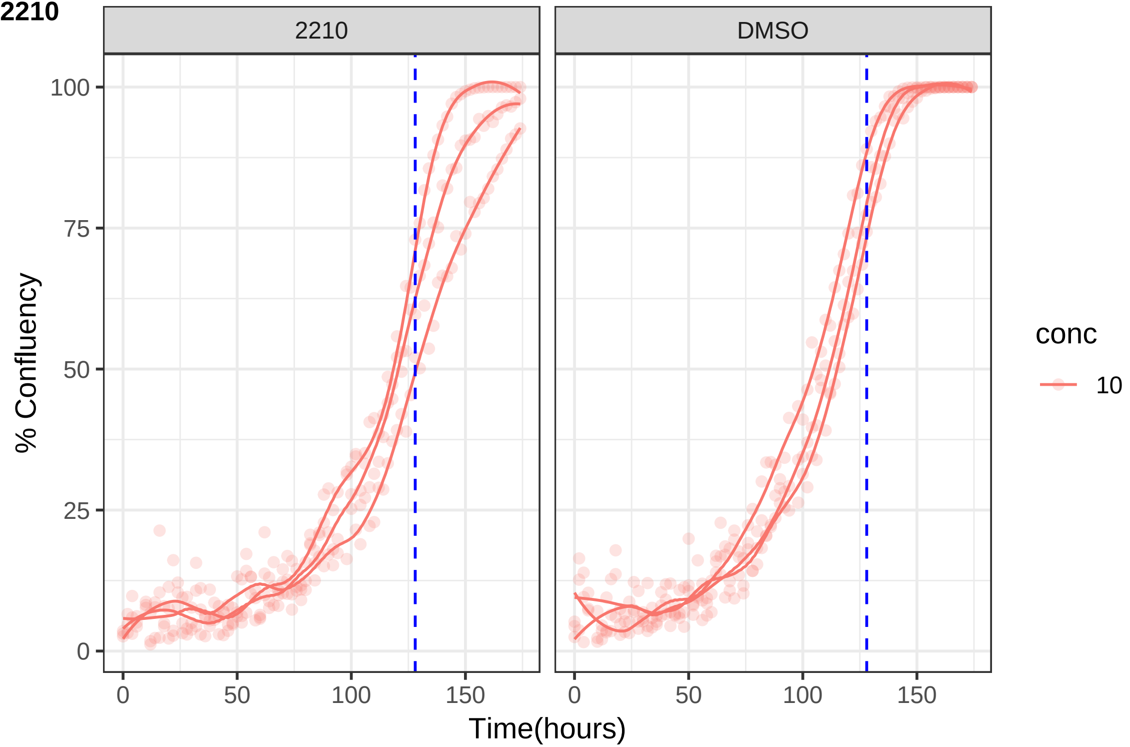
2210
DMSO
100
75
50
25
0
0
50
100
150
% Confluency
Time(hours)
conc
10

2223

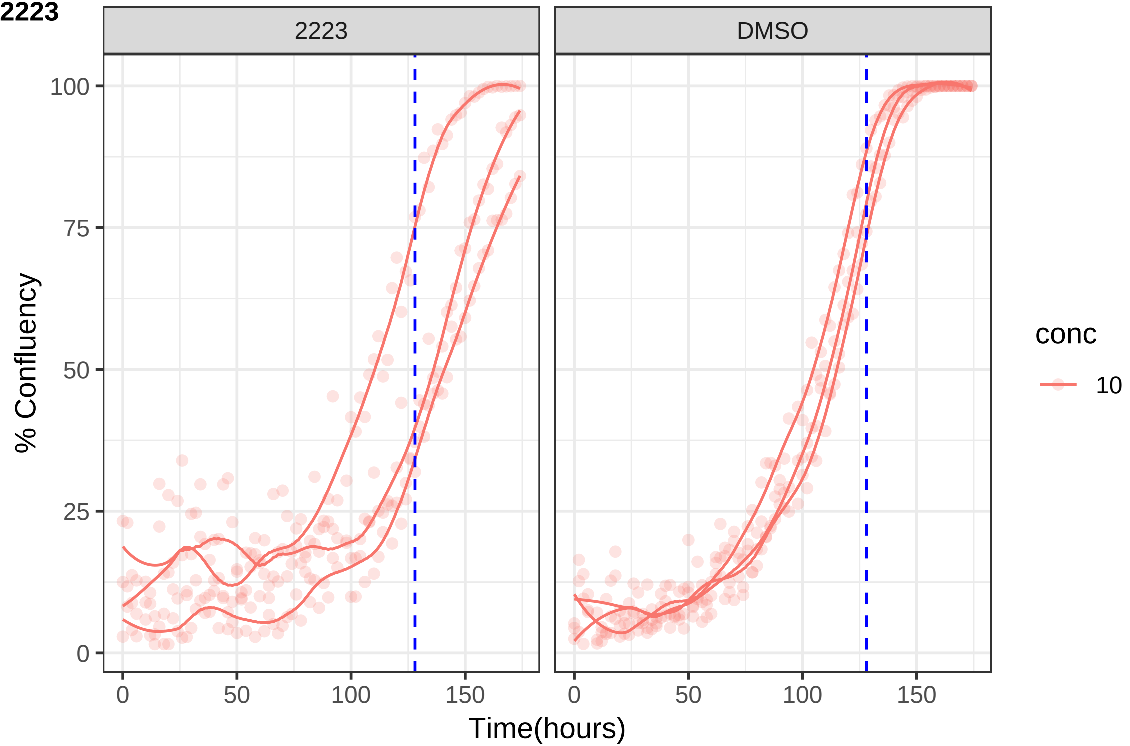
2223
DMSO
% Confluency
0
25
50
75
100
0
50
100
150
0
50
100
150
Time(hours)
conc
10

2227

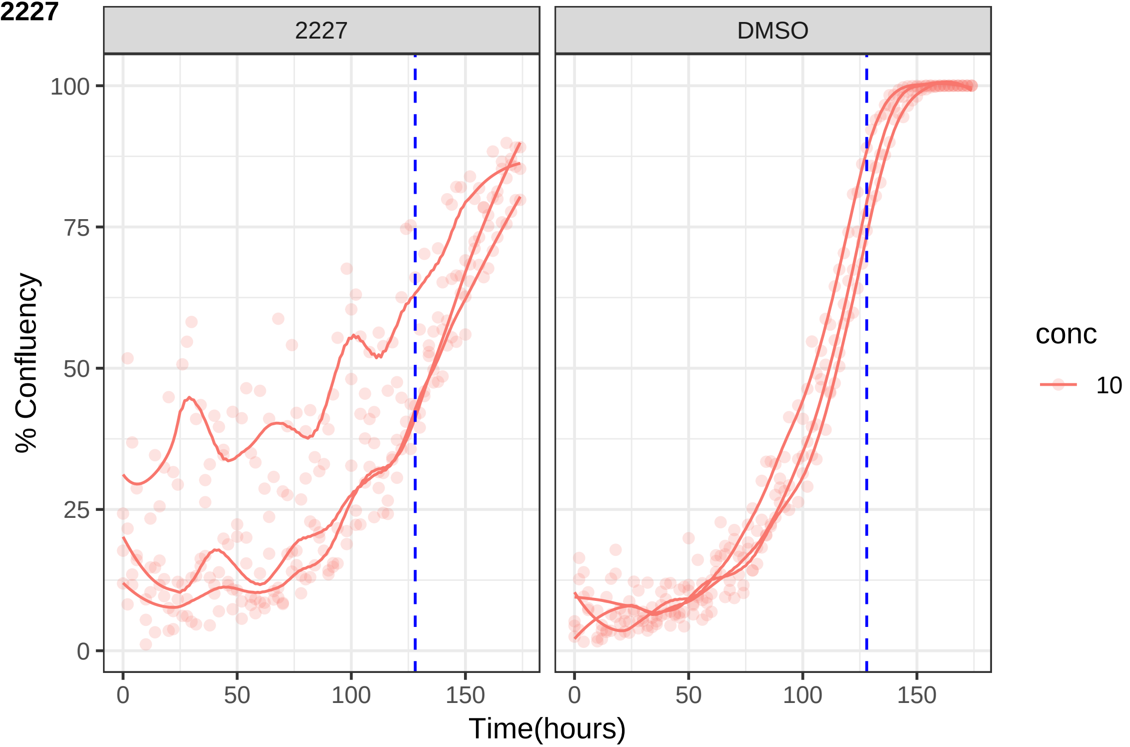

2227
DMSO
100
75
50
25
0
% Confluency
0
50
100
150
Time(hours)
conc
10

2241

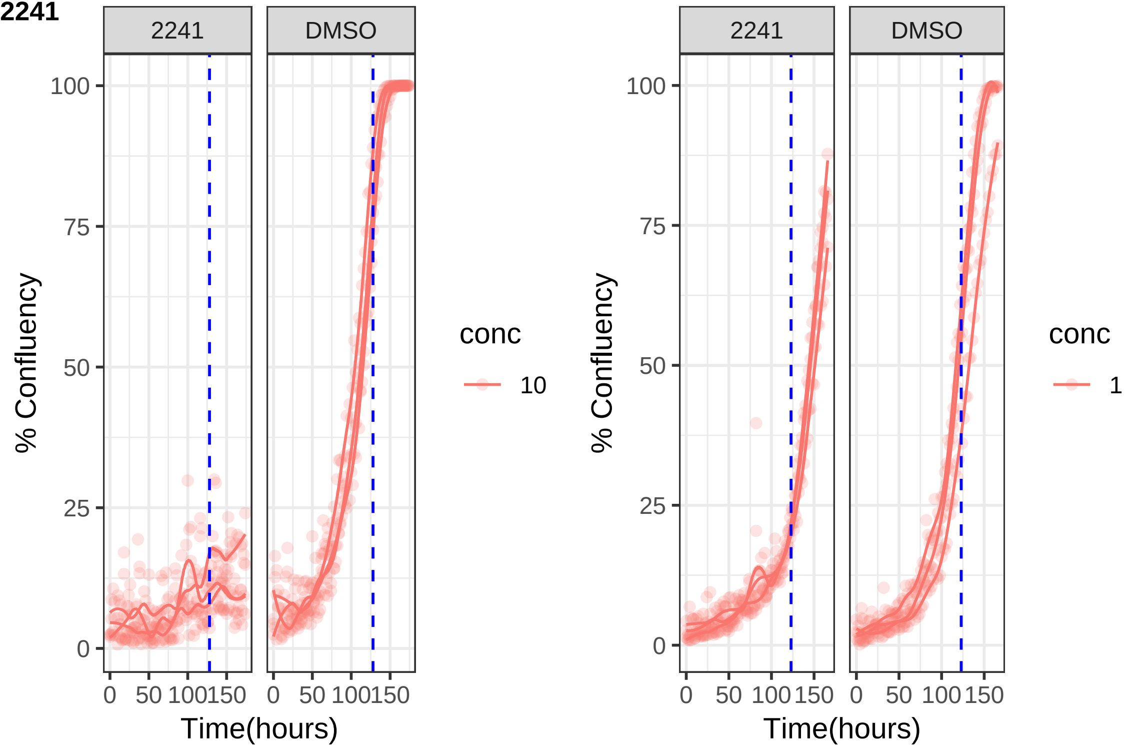

2241
DMSO
% Confluency
100
75
50
25
0
0
50
100
150
Time(hours)
conc
10
2241
DMSO
% Confluency
100
75
50
25
0
0
50
100
150
Time(hours)
conc
1

2242

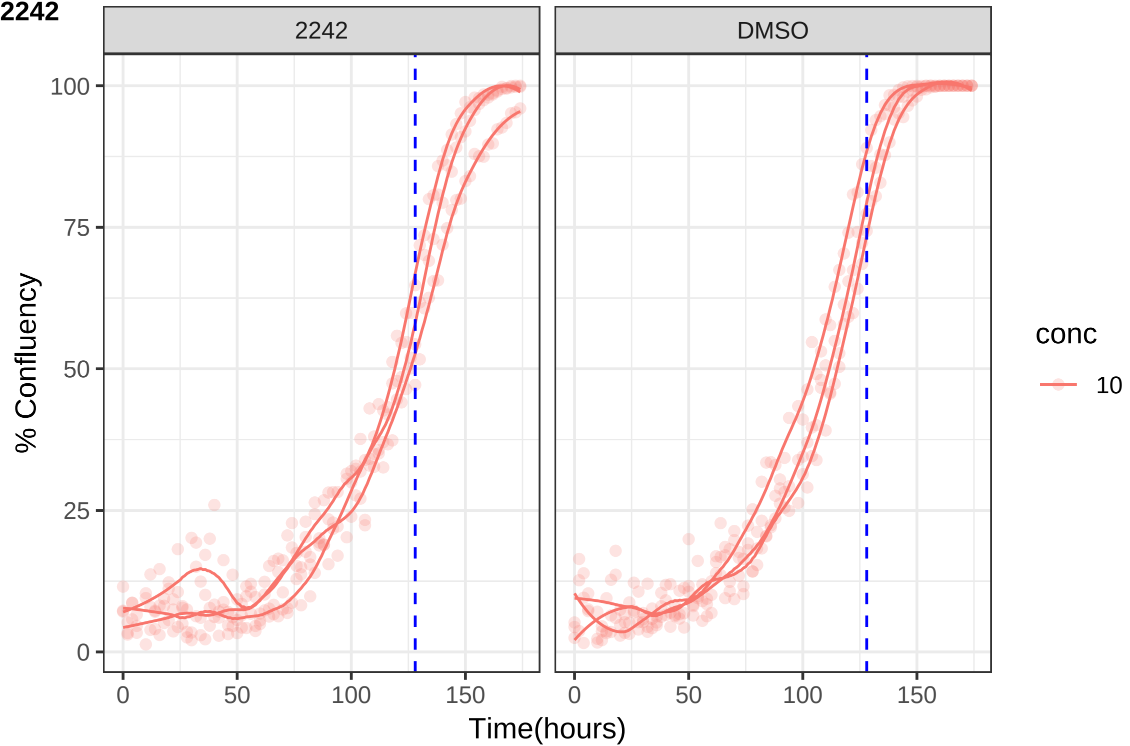

2242
DMSO
100
75
50
25
0
% Confluency
0
50
100
150
Time(hours)
conc
10

2244

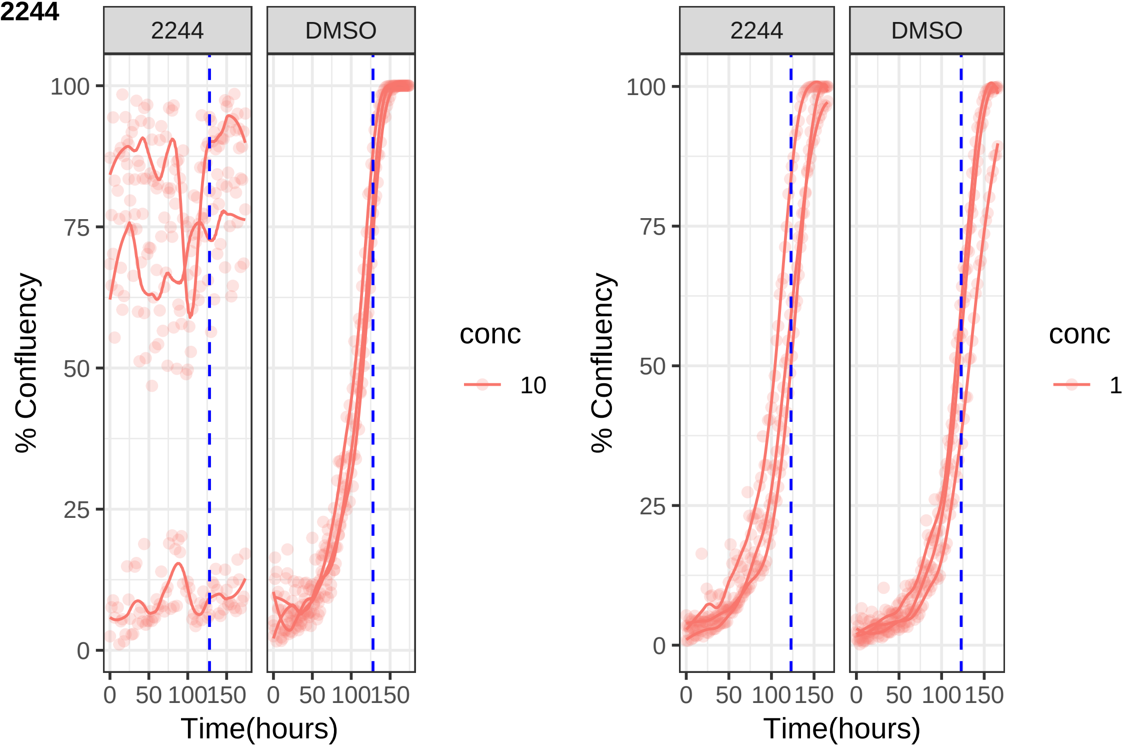
2244
DMSO
100
75
50
25
0
% Confluency
0
50
100
150
Time(hours)
conc
10
2244
DMSO
100
75
50
25
0
% Confluency
0
50
100
150
Time(hours)
conc
1

2249

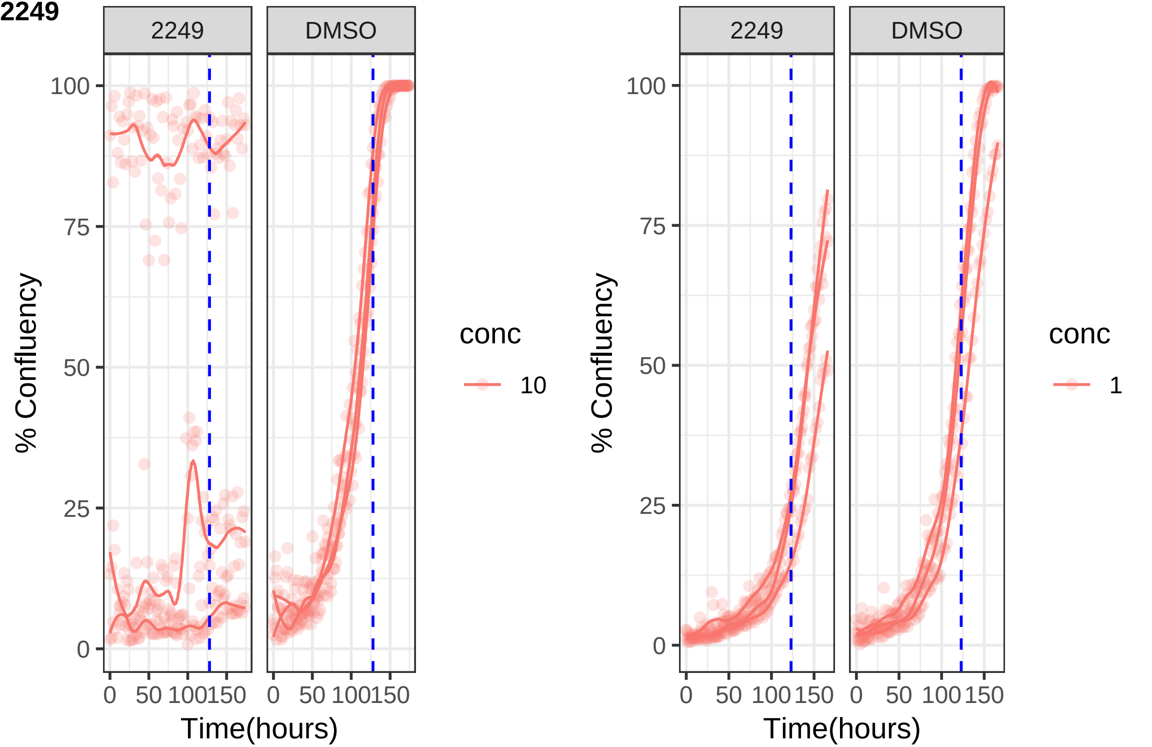
2249
DMSO
100
75
50
25
0
% Confluency
0 50 100 150
Time(hours)
conc
10
2249
DMSO
100
75
50
25
0
% Confluency
0 50 100 150
Time(hours)
conc
1

2250

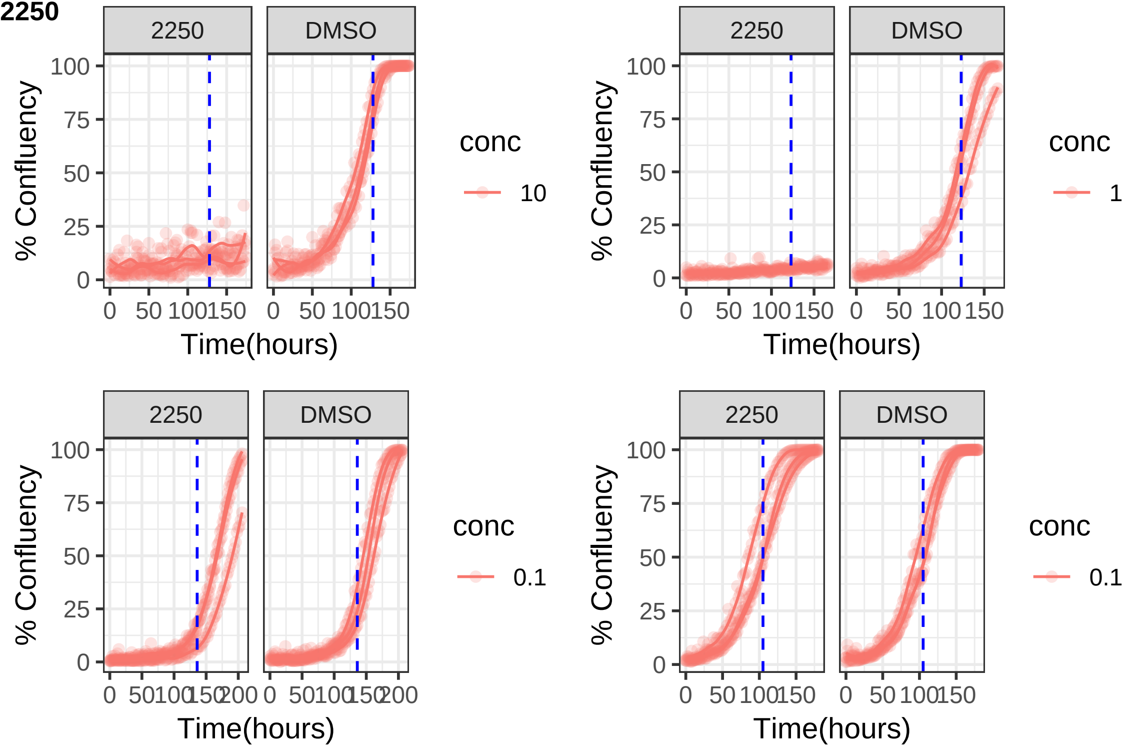
2250
DMSO
% Confluency
Time(hours)
conc
10
1
0.1
0
25
50
75
100
0
50
100
150
200

2262

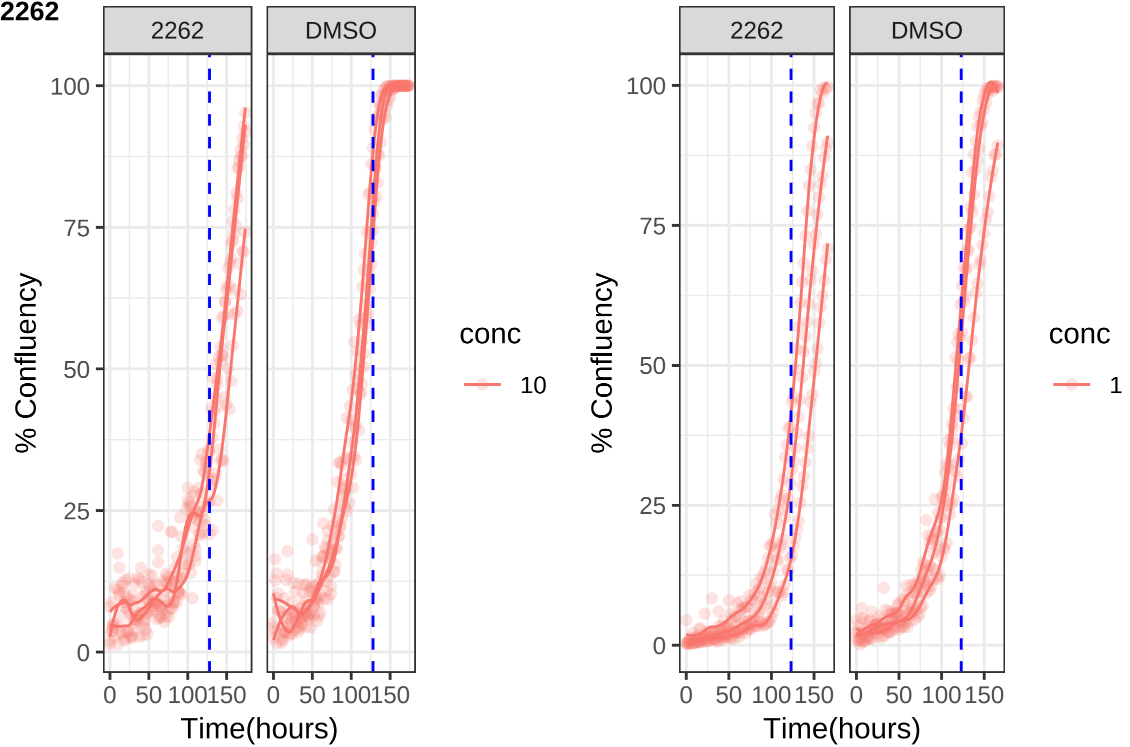

2262
DMSO
% Confluency
Time(hours)
0
25
50
75
100
0
50
100
150
conc
10
1

2268

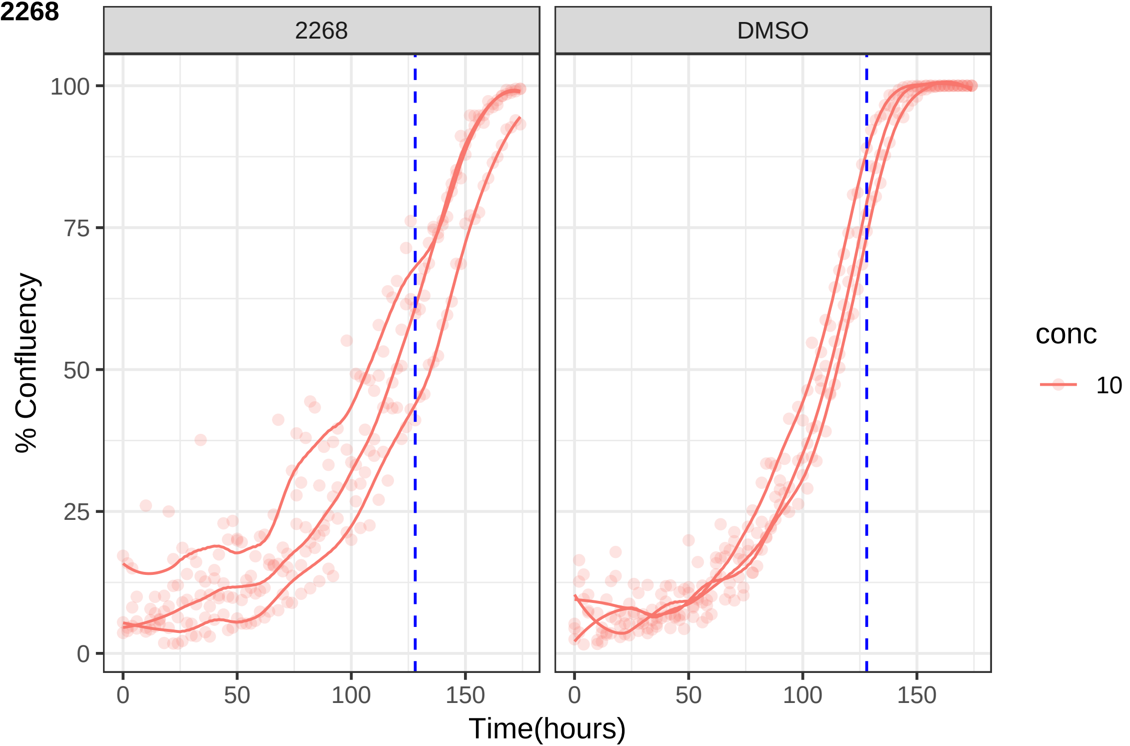
2268
DMSO
% Confluency
0
25
50
75
100
0
50
100
150
Time(hours)
conc
10

2278

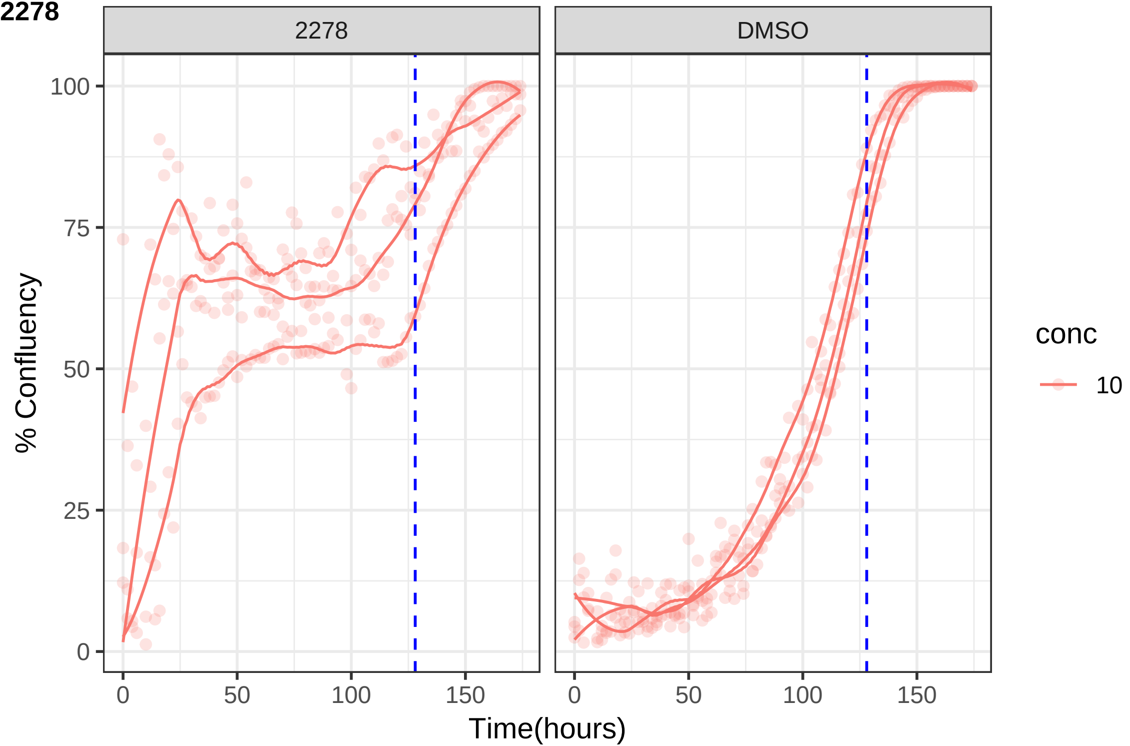
2278
DMSO
100
75
50
25
0
0
50
100
150
% Confluency
Time(hours)
conc
10

2293

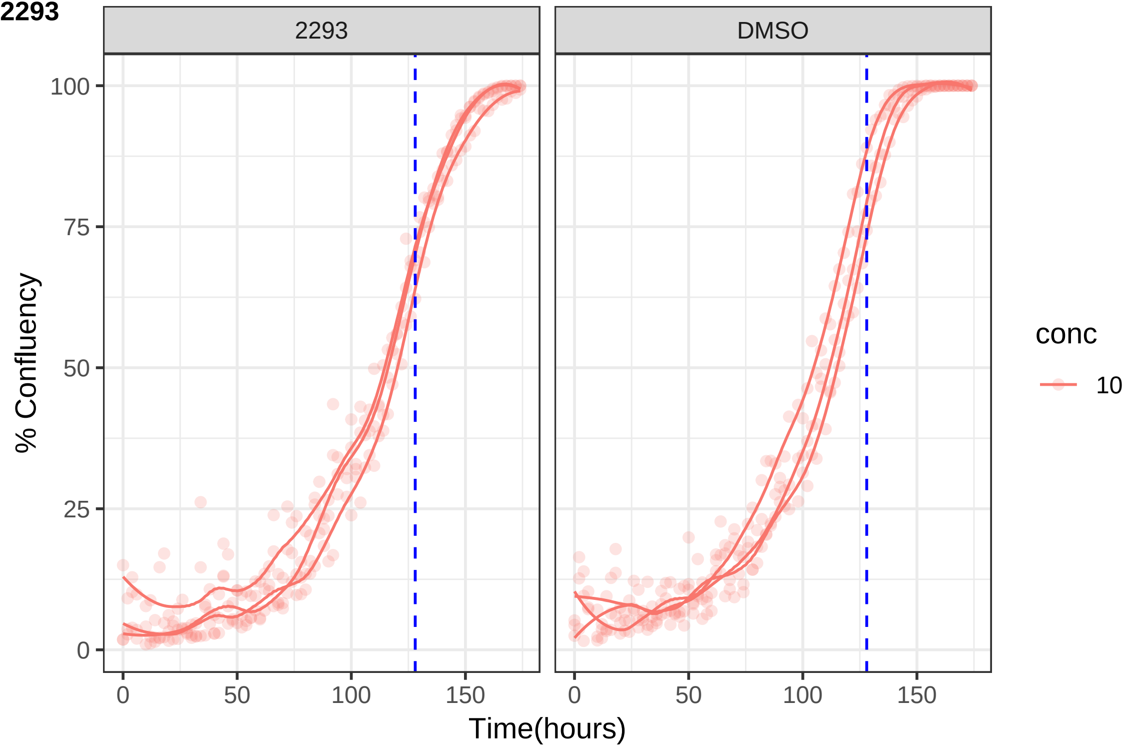
2293
DMSO
% Confluency
100
75
50
25
0
0
50
100
150
Time(hours)
conc
10

2300

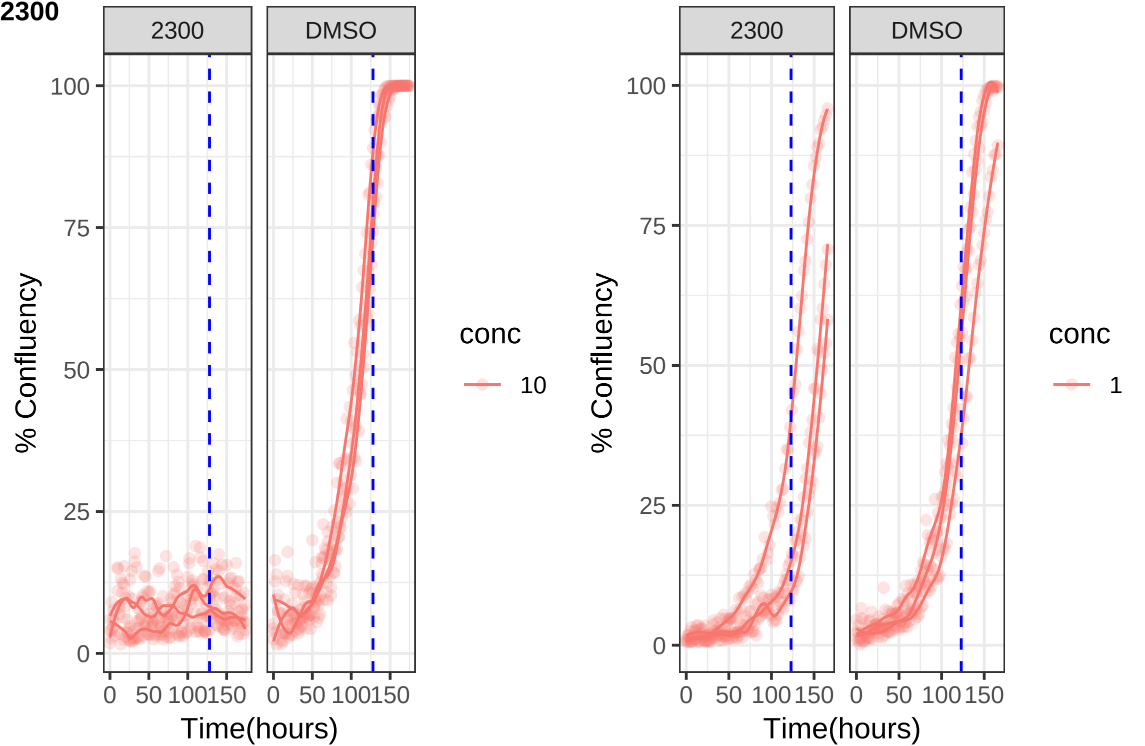
2300
DMSO
100
75
50
25
0
% Confluency
0
50
100
150
Time(hours)
conc
10
2300
DMSO
100
75
50
25
0
% Confluency
0
50
100
150
Time(hours)
conc
1

2319

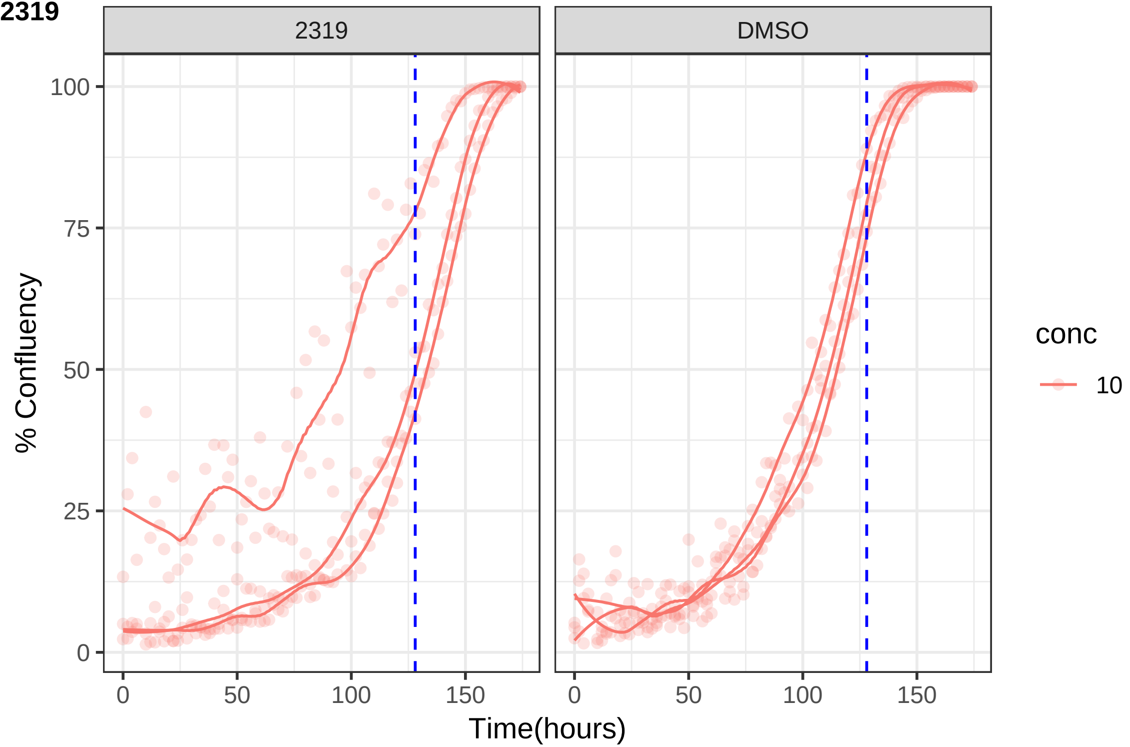

2319
DMSO
100
75
50
25
0
% Confluency
0
50
100
150
Time(hours)
conc
10

2323

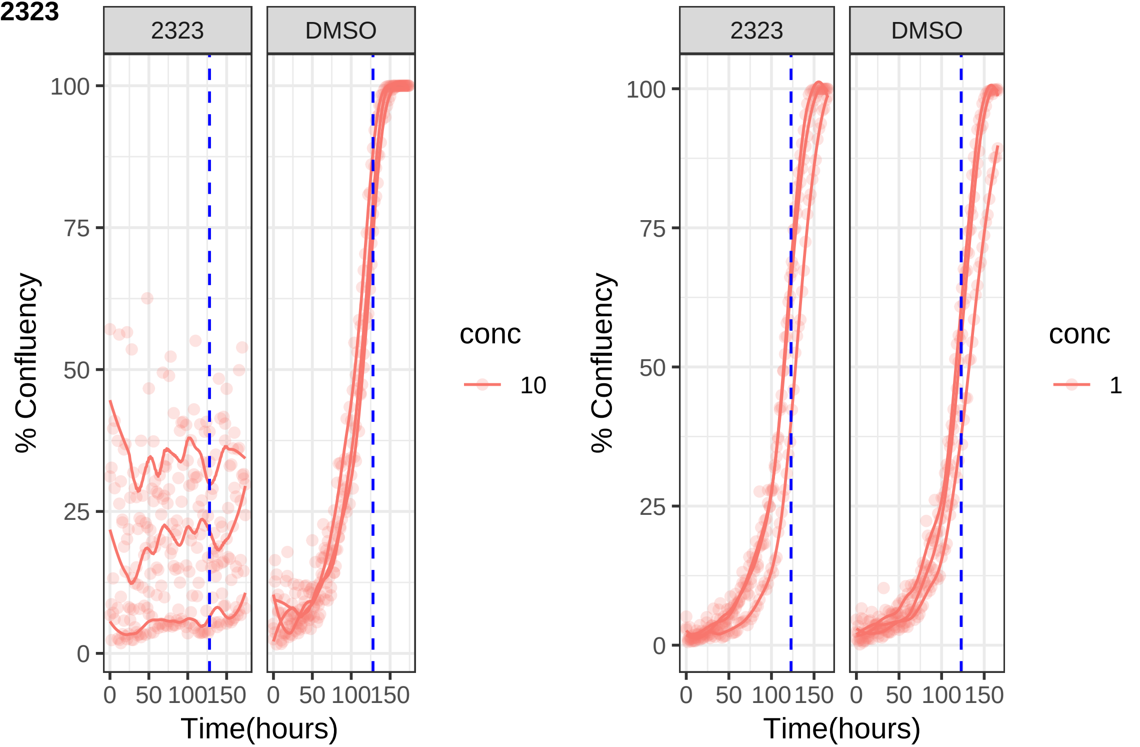

2323
DMSO
100
75
50
25
0
% Confluency
0
50
100
150
Time(hours)
conc
10
2323
DMSO
100
75
50
25
0
% Confluency
0
50
100
150
Time(hours)
conc
1

2325

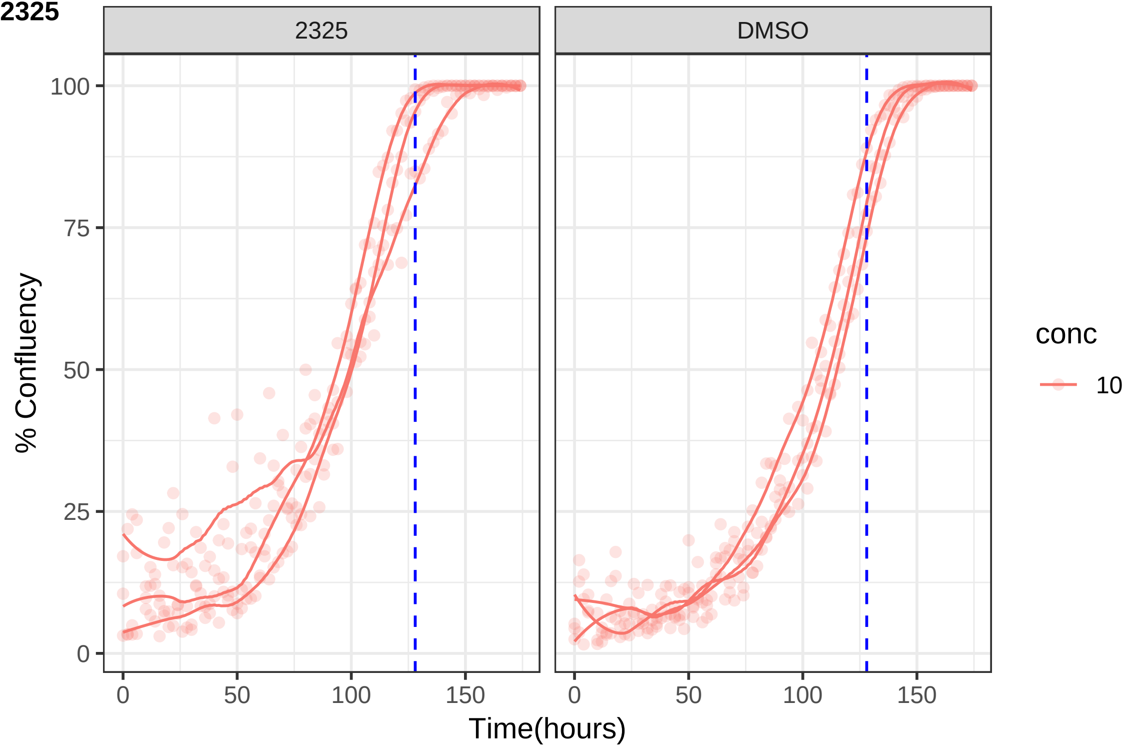

2325
DMSO
100
75
50
25
0
0
50
100
150
0
50
100
150
% Confluency
Time(hours)
conc
10

2326

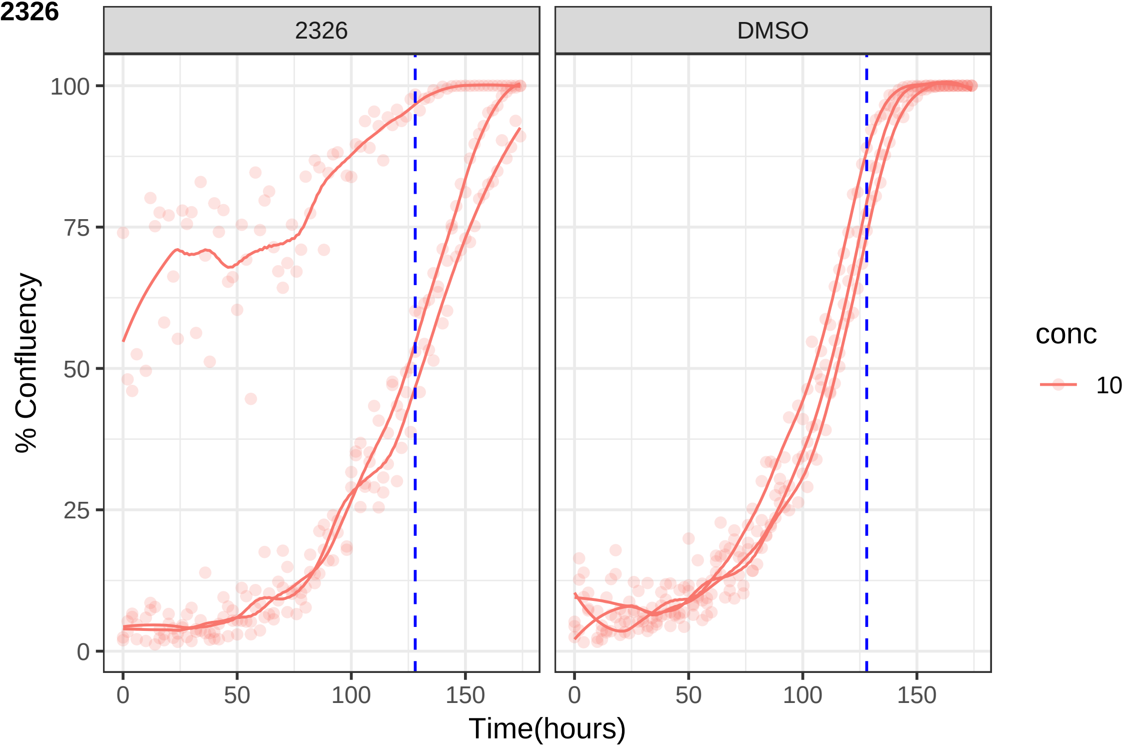
2326
DMSO
100
75
50
25
0
% Confluency
0
50
100
150
Time(hours)
conc
10

2327

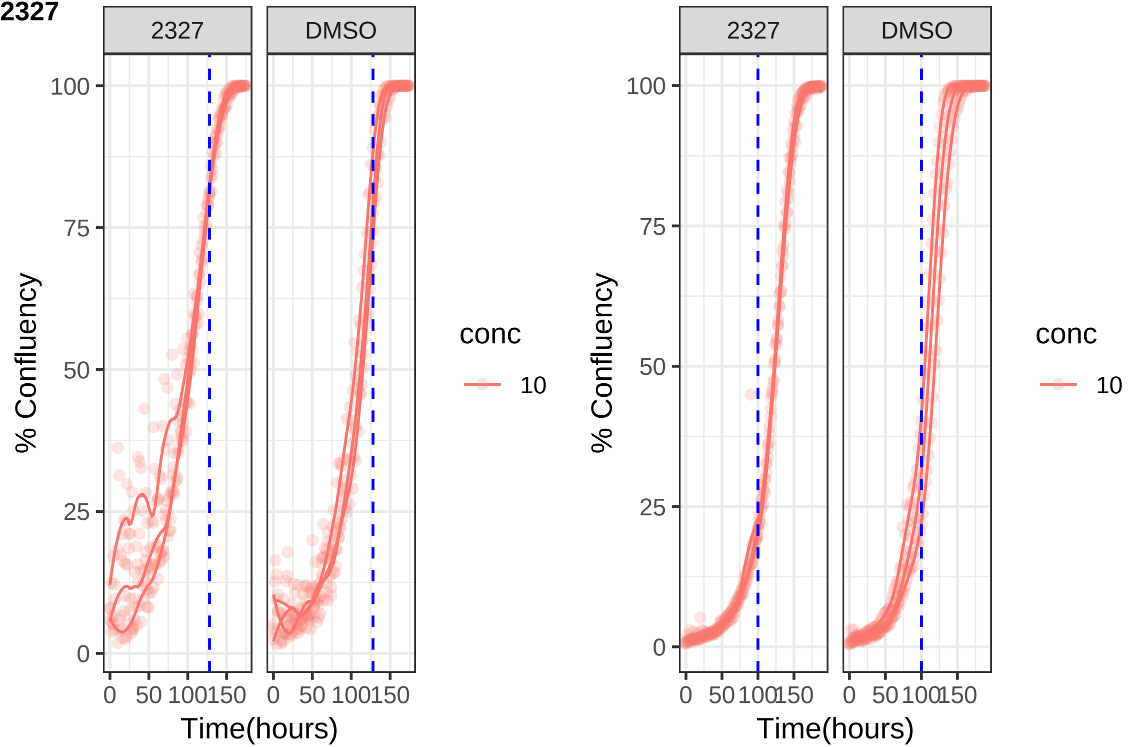

2327
DMSO
100
75
50
25
0
% Confluency
0
50
100
150
Time(hours)
conc
10
2327
DMSO
100
75
50
25
0
% Confluency
0
50
100
150
Time(hours)
conc
10

2339

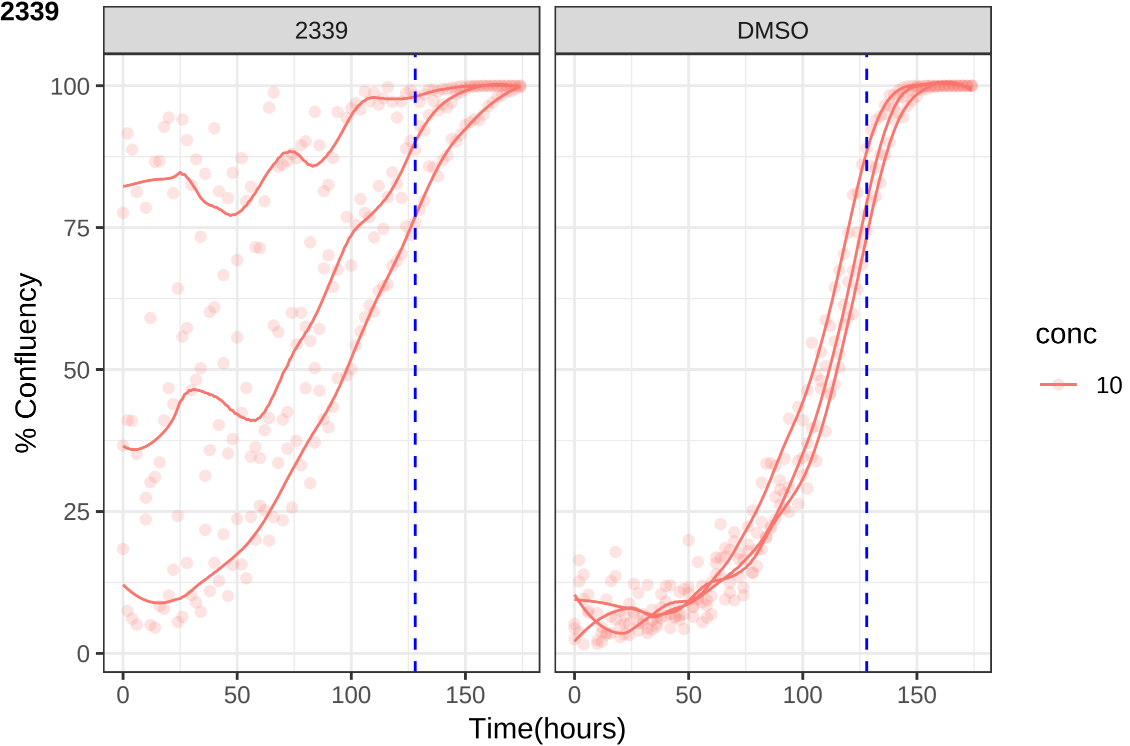

2339
DMSO
% Confluency
100
75
50
25
0
0
50
100
150
Time(hours)
conc
10

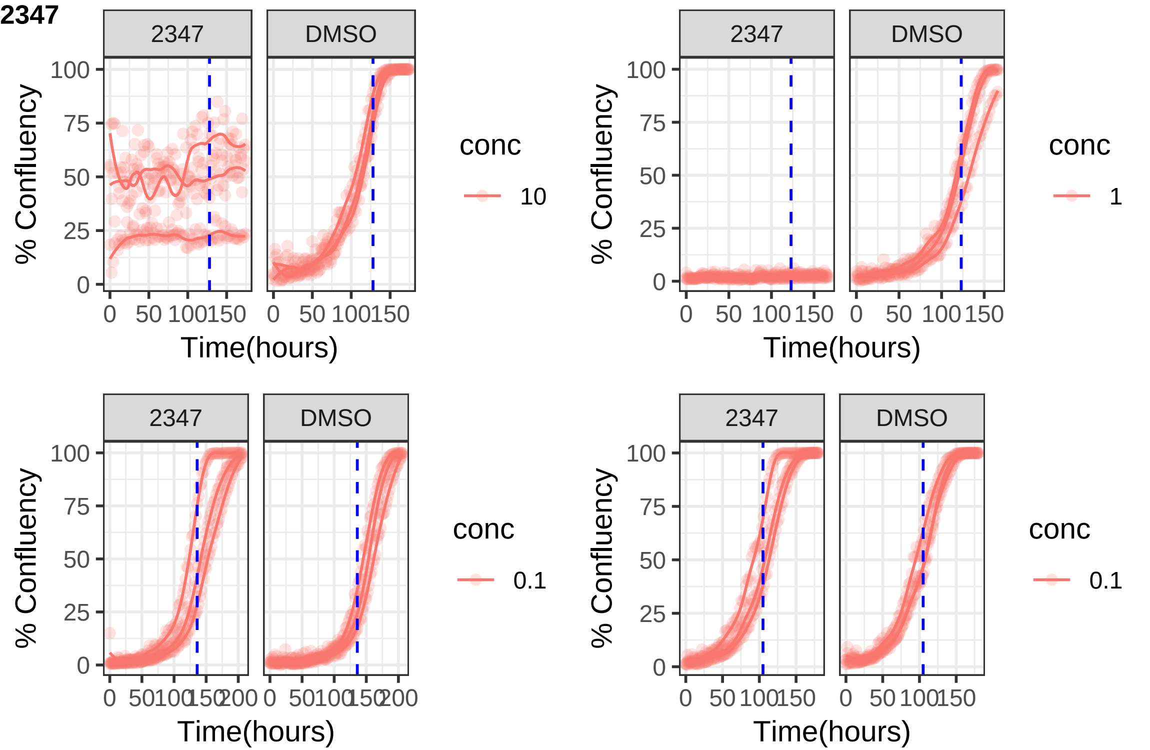
2347
2347
DMSO
% Confluency
100
75
50
25
0
0
50
100
150
Time(hours)
conc
10
1
0.1
200

2358

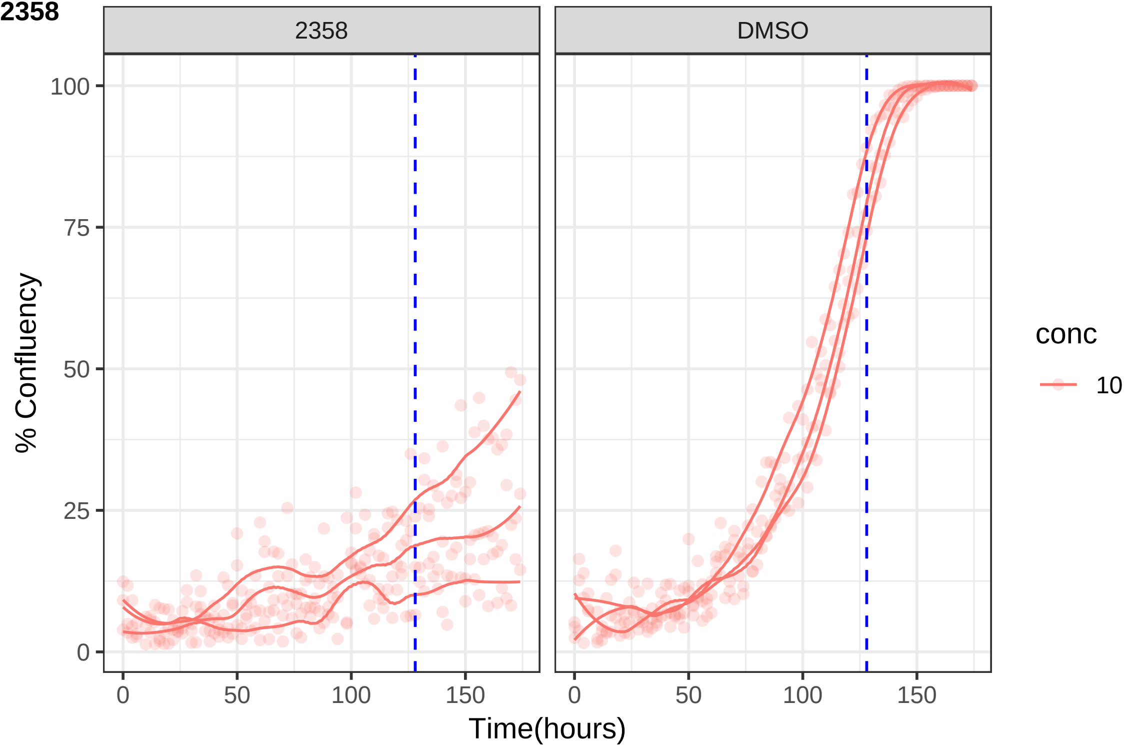

2358
DMSO
100
75
50
25
0
% Confluency
0
50
100
150
Time(hours)
conc
10

2359

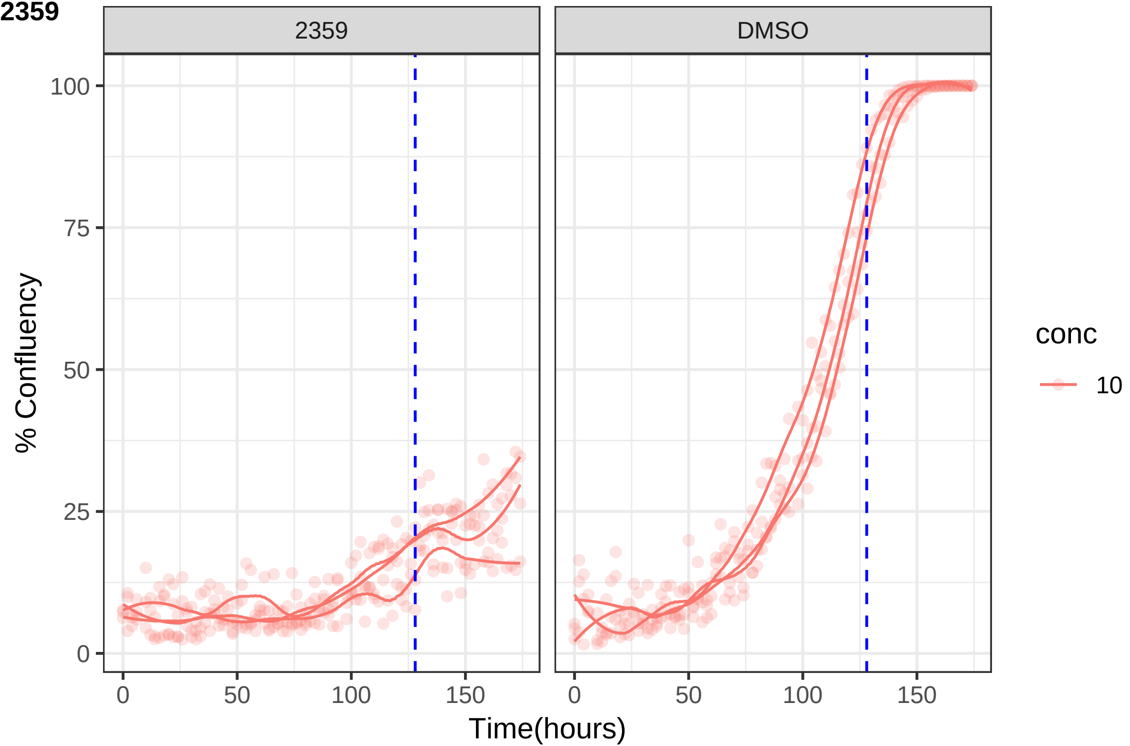
2359
DMSO
100
75
50
25
0
% Confluency
0
50
100
150
Time(hours)
conc
10

2369

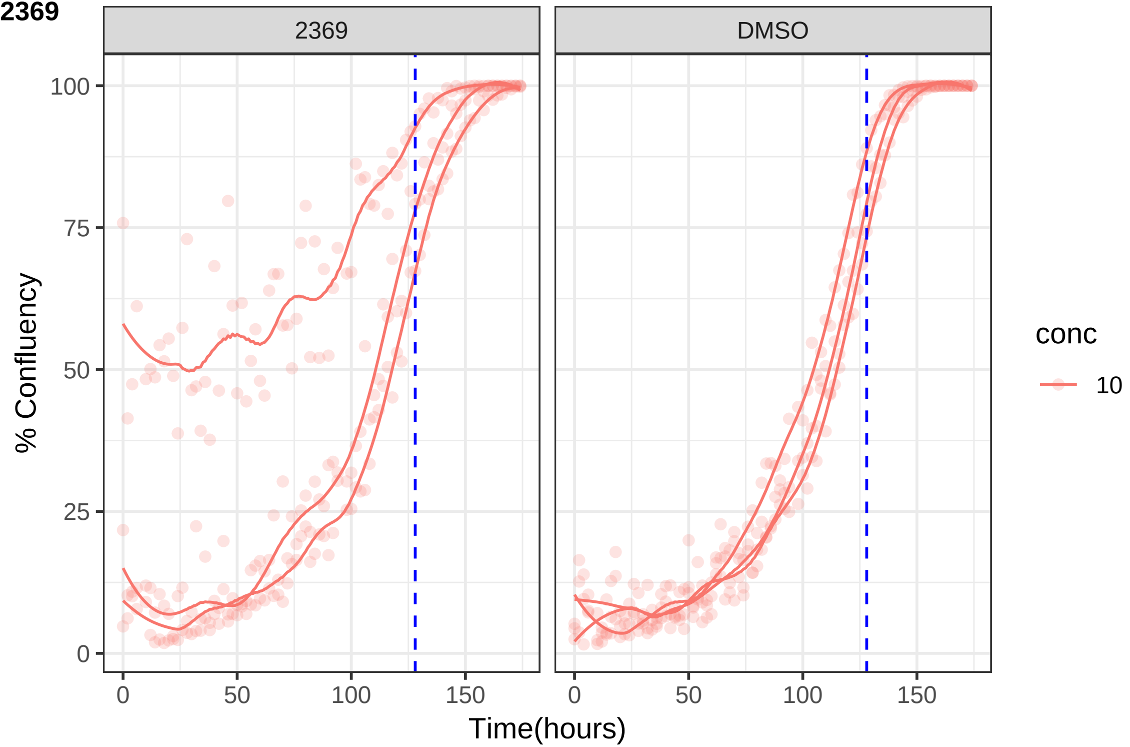
2369
DMSO
% Confluency
0
25
50
75
100
0
50
100
150
Time(hours)
conc
10

2375

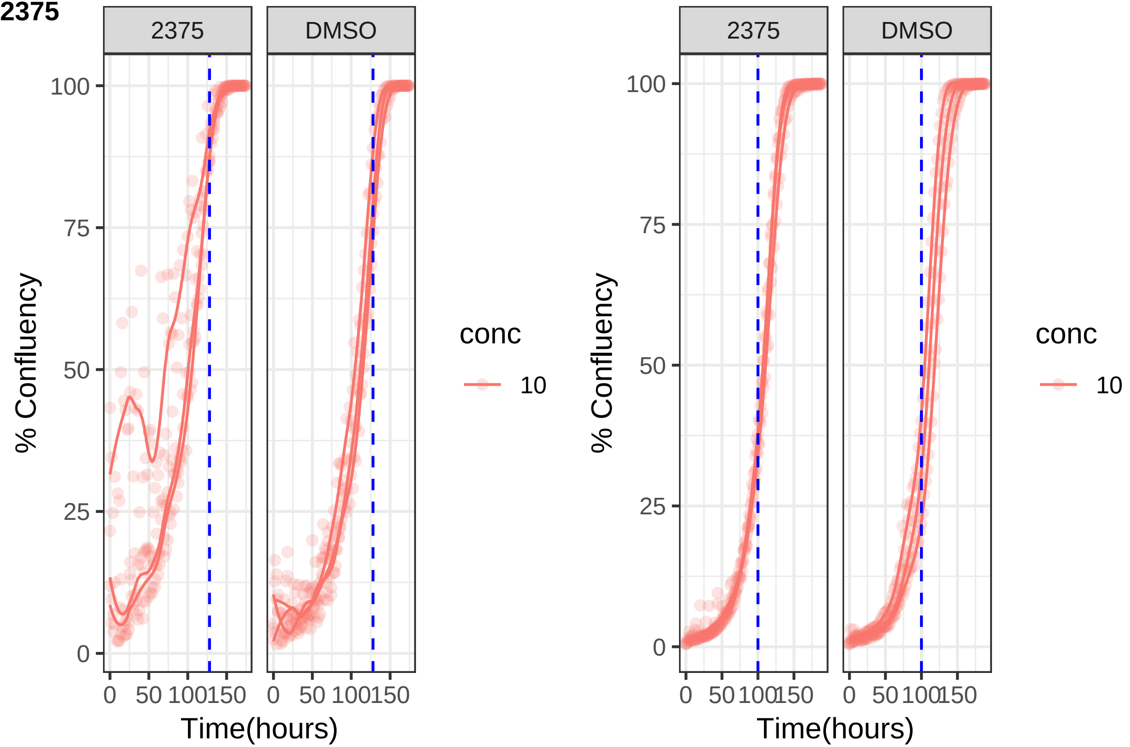

2375
DMSO
% Confluency
100
75
50
25
0
0
50
100
150
Time(hours)
conc
10
2375
DMSO
% Confluency
Time(hours)
conc
10

2379

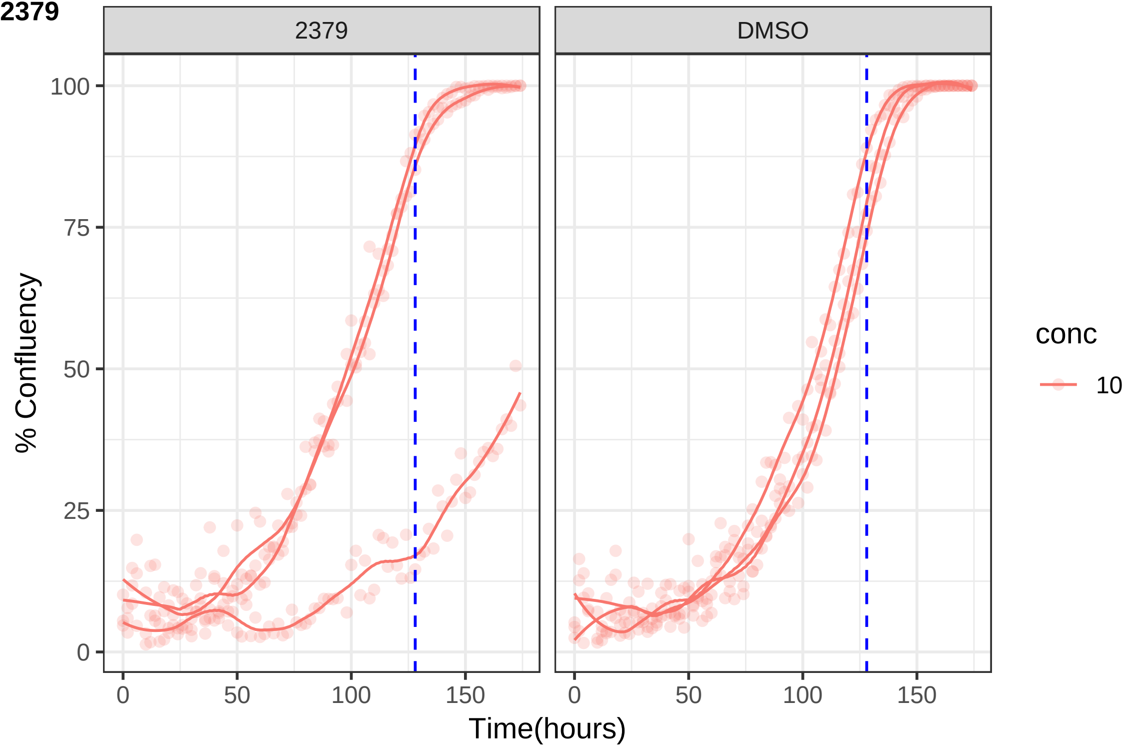

2379
DMSO
100
75
50
25
0
% Confluency
0
50
100
150
Time(hours)
conc
10

2402

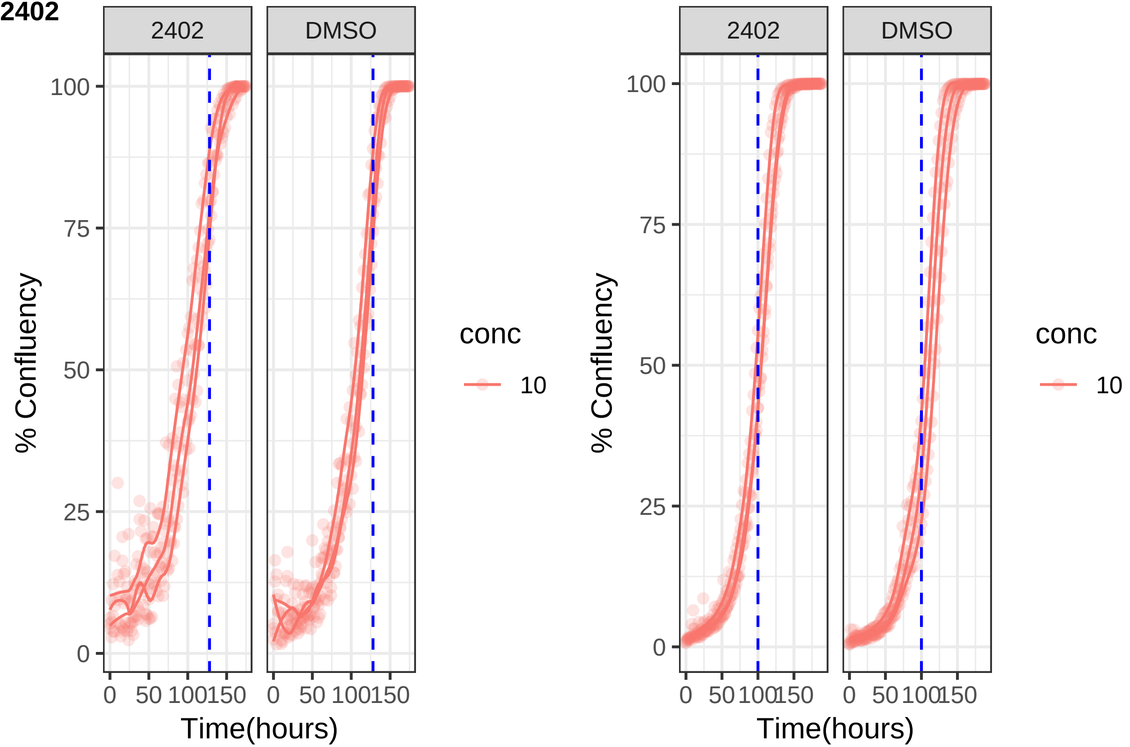

2402
DMSO
% Confluency
100
75
50
25
0
0
50
100
150
Time(hours)
conc
10

2831

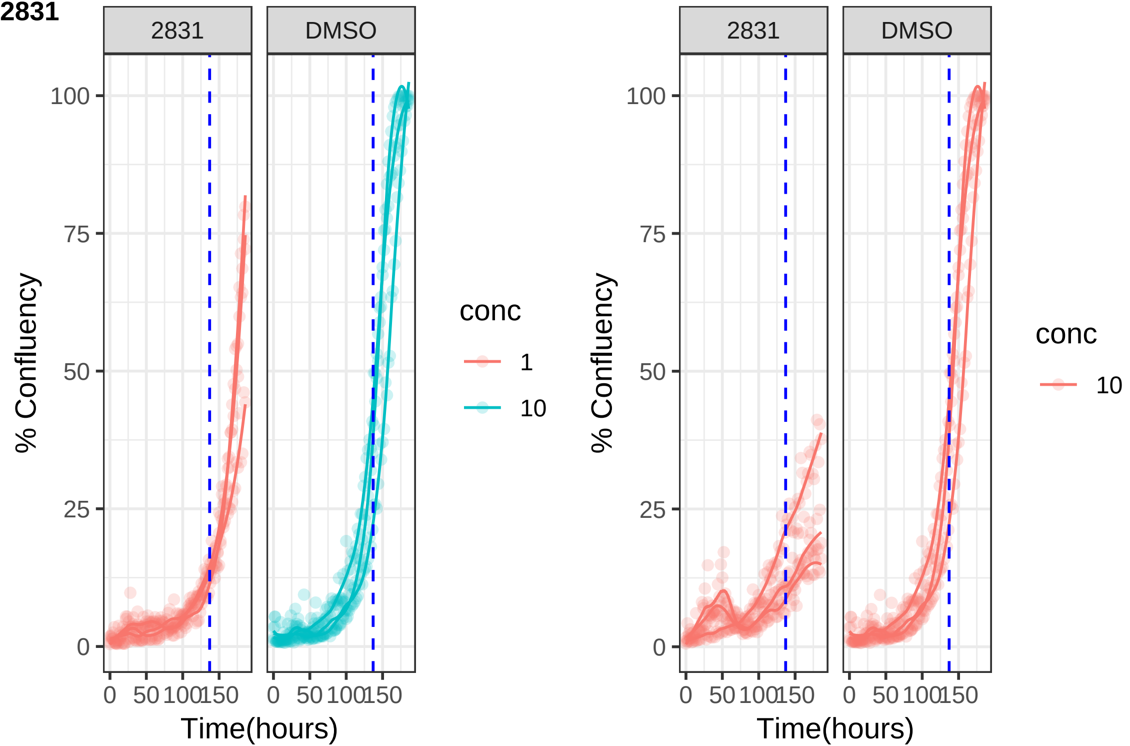
2831
DMSO
100
75
50
25
0
% Confluency
0 50 100 150
Time(hours)
conc
1
10
2831
DMSO
% Confluency
Time(hours)
conc
10

2ndcontrol

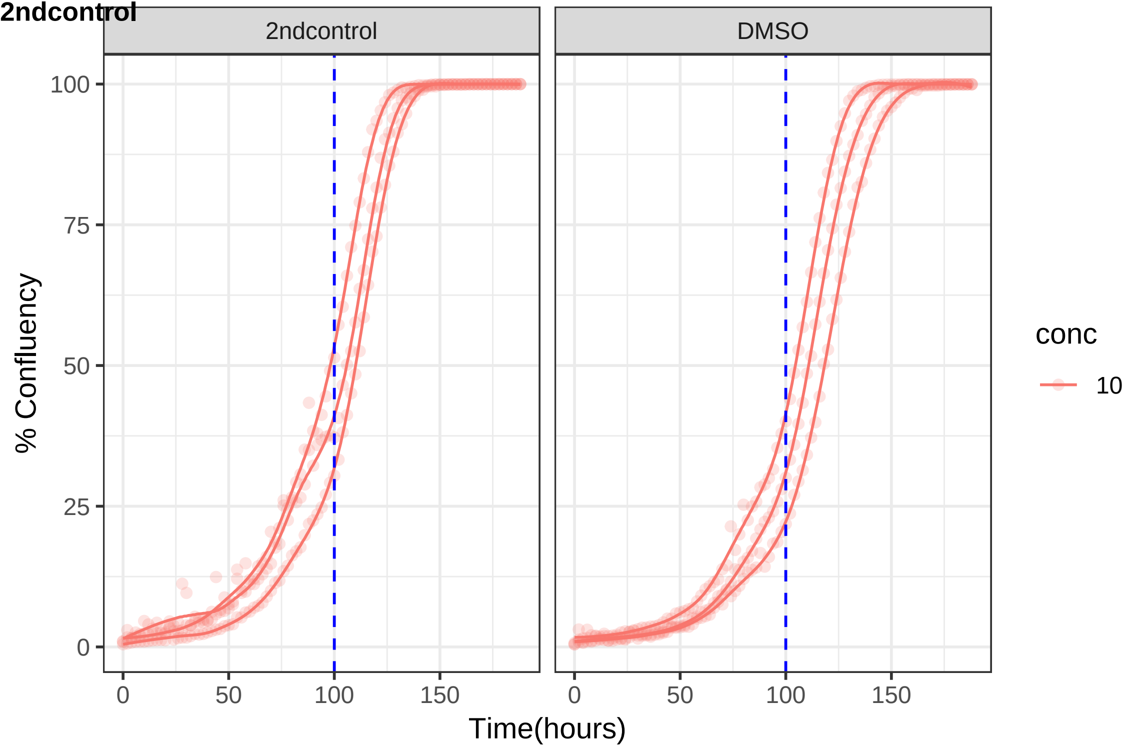
2ndcontrol
DMSO
% Confluency
100
75
50
25
0
0
50
100
150
0
50
100
150
Time(hours)
conc
10

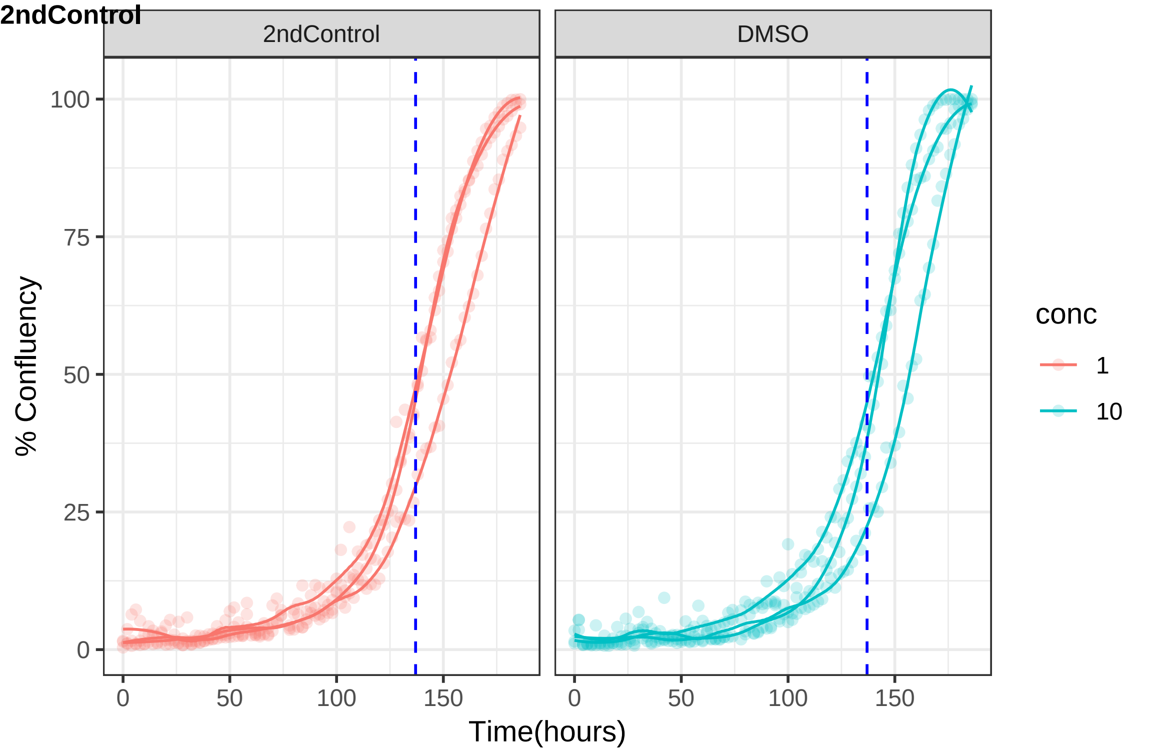
2ndControl
2ndControl
DMSO
100
75
50
25
0
% Confluency
0
50
100
150
0
50
100
150
Time(hours)
conc
1
10
